# Mining the contribution of intensive care clinical course to outcome after traumatic brain injury

**DOI:** 10.1038/s41746-023-00895-8

**Published:** 2023-08-21

**Authors:** Shubhayu Bhattacharyay, Pier Francesco Caruso, Cecilia Åkerlund, Lindsay Wilson, Robert D. Stevens, David K. Menon, Ewout W. Steyerberg, David W. Nelson, Ari Ercole, the CENTER-TBI investigators and participants

**Affiliations:** 1 Division of Anaesthesia, University of Cambridge, Cambridge, UK; 2 Department of Clinical Neurosciences, University of Cambridge, Cambridge, UK; 3 Laboratory of Computational Intensive Care Medicine, Johns Hopkins University, Baltimore, MD USA; 4 Department of Biomedical Sciences, Humanitas University, Via Rita Levi Montalcini 4, Pieve Emanuele, Milan, 20072 Italy; 5 Department of Physiology and Pharmacology, Section for Perioperative Medicine and Intensive Care, Karolinska Institutet, Stockholm, Sweden; 6 Division of Psychology, University of Stirling, Stirling, UK; 7 Department of Anesthesiology and Critical Care Medicine, Johns Hopkins University, Baltimore, MD USA; 8 Department of Biomedical Data Sciences, Leiden University Medical Center, Leiden, The Netherlands; 9 Cambridge Centre for Artificial Intelligence in Medicine, Cambridge, UK

**Keywords:** Brain injuries, Computational science, Prognostic markers, Data mining

## Abstract

Existing methods to characterise the evolving condition of traumatic brain injury (TBI) patients in the intensive care unit (ICU) do not capture the context necessary for individualising treatment. Here, we integrate all heterogenous data stored in medical records (1166 pre-ICU and ICU variables) to model the individualised contribution of clinical course to 6-month functional outcome on the Glasgow Outcome Scale -Extended (GOSE). On a prospective cohort (*n* = 1550, 65 centres) of TBI patients, we train recurrent neural network models to map a token-embedded time series representation of all variables (including missing values) to an ordinal GOSE prognosis every 2 h. The full range of variables explains up to 52% (95% CI: 50–54%) of the ordinal variance in functional outcome. Up to 91% (95% CI: 90–91%) of this explanation is derived from pre-ICU and admission information (i.e., static variables). Information collected in the ICU (i.e., dynamic variables) increases explanation (by up to 5% [95% CI: 4–6%]), though not enough to counter poorer overall performance in longer-stay (>5.75 days) patients. Highest-contributing variables include physician-based prognoses, CT features, and markers of neurological function. Whilst static information currently accounts for the majority of functional outcome explanation after TBI, data-driven analysis highlights investigative avenues to improve the dynamic characterisation of longer-stay patients. Moreover, our modelling strategy proves useful for converting large patient records into interpretable time series with missing data integration and minimal processing.

## Introduction

Traumatic brain injury (TBI) is the most frequently occurring neurological disorder and imposes a substantial public health burden^1,2^. Whilst TBI is increasingly appreciated as a progressive condition rather than a single event, the disease course of TBI patients in the intensive care unit (ICU) has not been well-characterised. As a result, existing ICU treatments are based on limited evidence and do not target the heterogeneous mechanisms of an individual’s TBI^3^. Answering the call for patient-tailored treatments (i.e., precision medicine), issued by *The Lancet Neurology* Commissions on TBI^1,2^, must start with an evidence-based understanding of individual patient trajectories in the ICU.

The main instrument for characterising TBI severity in the ICU is the Glasgow Coma Scale (GCS), for which a patient’s best motor, verbal, and eye responses are assessed^4^. The GCS, however, is not sufficient for precision medicine as it does not capture a patient’s pathophysiological profile and is confounded by external factors (e.g., drug use, medications, and tracheal intubation)^5^. An alternative approach is to characterise severity through functional outcome prognosis. Functional outcome after TBI is typically evaluated on the ordinal, eight-point Glasgow Outcome Scale-Extended (GOSE)^6^, and currently, the best validated prognostic tools for moderate-to-severe TBI (GCS ≤ 12) are the International Mission for Prognosis and Analysis of Clinical Trials in TBI (IMPACT) models^7^. The IMPACT extended model estimates the probability of either survival (GOSE > 1) or functional independence (GOSE > 4) at 6 months post-injury from ten static predictors collected from the first 24 h of ICU stay and explains approximately 35% of the pseudo-variance in dichotomised GOSE^7^. Considering a patient’s full, dynamic clinical course and increasing model output granularity (i.e., ordinal GOSE prognosis^8^) would further enable clinical characterisation through prognosis whilst uncovering the outcome contribution of ICU events and treatments.

In this work, we take a full-context, data-driven approach to assess the limits of dynamic ICU characterisation after TBI. The Collaborative European NeuroTrauma Effectiveness Research in TBI (CENTER-TBI) project represents the most comprehensive set of pre-ICU and ICU data for TBI patients across Europe^5,9^. Mining clinical trajectories from this data—which comprises a complex combination of modalities with varying structure, sampling, and missingness—is not straightforward. We therefore develop a regularised disease course modelling strategy which integrates all this heterogenous information and returns an interpretable, detailed proxy for severity over each patient’s ICU stay.

Upon developing our TBI modelling strategy, our central aims were to: (1) evaluate the additive prognostic significance of incorporating the most complete description of ICU stay available and (2) uncover clinical events most strongly associated with transitions in an individual’s trajectory. We also assess the reliability (i.e., calibration) and information content of our explanatory modelling approach to validate its application in deriving insight from medical data.

## Results

### Study population

Of the 2138 CENTER-TBI patients available for analysis in the ICU stratum of the core study, 1550 met the additional inclusion criteria of this work (Supplementary Fig. 1). Since the regularity of bihourly assessments collected for CENTER-TBI decreased after a week (Supplementary Fig. 2), and since over half the population remained at this point (Supplementary Fig. 3), we focused our analysis on the first week after ICU admission and the last week before ICU discharge. The summary characteristics of our study population are detailed in Table 1. Additional characteristics (e.g., race, comorbidities, and vitals) of our study population have been previously published^5^, and distributions of all study variables are available online (https://www.center-tbi.eu/data/dictionary).

**Table 1:** Summary characteristics of the study population at ICU admission stratified by ordinal 6-month outcomes.

Summary characteristics	Overall	Glasgow Outcome Scale-Extended (GOSE) at 6 months post-injury							*P* value
		(1) Death	(2 or 3) Vegetative or lower severe disability	(4) Upper severe disability	(5) Lower moderate disability	(6) Upper moderate disability	(7) Lower good recovery	(8) Upper good recovery	
*n*	1550	318 (20.5%)	262 (16.9%)	120 (7.7%)	227 (14.6%)	200 (12.9%)	206 (13.3%)	217 (14.0%)	
Age (years)	51 (31–66)	66 (50–76)	55 (36–68)	48 (29–61)	44 (31–56)	41 (27–53)	48 (31–65)	41 (24–61)	<0.0001
Sex									0.94
Female	409 (26.4%)	78 (24.5%)	71 (27.1%)	43 (35.8%)	64 (28.2%)	49 (24.5%)	59 (28.6%)	45 (20.7%)	
Baseline GCS (*n* = 1465)	8 (4–14)	5 (3–10)	6 (3–10)	8 (4–13)	8 (5–13)	9 (6–14)	13 (7–15)	13 (8–15)	<0.0001
Mild (13–15)	390 (26.6%)	30 (10.3%)	38 (15.3%)	26 (23.4%)	42 (19.5%)	66 (34.9%)	91 (45.3%)	97 (46.4%)	
Moderate (9–12)	331 (22.6%)	65 (22.3%)	41 (16.5%)	28 (25.2%)	65 (30.2%)	36 (19.0%)	40 (19.9%)	56 (26.8%)	
Severe (3–8)	744 (50.8%)	196 (67.4%)	170 (68.3%)	57 (51.4%)	108 (50.2%)	87 (46.0%)	70 (34.8%)	56 (26.8%)	
Marshall CT (*n* = 1255)	VI (II–VI)	III (II–VI)	II (II–VI)	II (II–VI)	II (II–II)	II (II–III)	II (II–II)	VI (II–VI)	0.02
No visible pathology (I)	118 (9.4%)	8 (3.3%)	11 (5.3%)	5 (5.2%)	17 (8.7%)	25 (15.2%)	24 (13.6%)	28 (16.5%)	
Diffuse injury II	592 (47.2%)	56 (22.8%)	84 (40.6%)	54 (56.2%)	92 (47.2%)	100 (60.6%)	103 (58.5%)	103 (60.6%)	
Diffuse injury III	108 (8.6%)	42 (17.1%)	17 (8.2%)	10 (10.4%)	14 (7.2%)	9 (5.5%)	6 (3.4%)	10 (5.9%)	
Diffuse injury IV	16 (1.3%)	7 (2.8%)	1 (0.5%)	1 (1.0%)	4 (2.1%)	1 (0.6%)	1 (0.6%)	1 (0.6%)	
Mass lesion (V & VI)	421 (33.5%)	133 (54.0%)	94 (45.4%)	26 (27.1%)	68 (34.9%)	30 (18.2%)	42 (23.9%)	28 (16.5%)	
tSAH (*n* = 1254)	957 (76.3%)	221 (90.2%)	176 (84.2%)	73 (76.0%)	150 (76.9%)	106 (63.9%)	125 (71.4%)	106 (63.1%)	0.12
EDH (*n* = 1257)	244 (19.4%)	31 (12.7%)	32 (15.3%)	21 (21.9%)	46 (23.6%)	32 (19.3%)	42 (23.9%)	40 (23.5%)	0.01
Retired (*n* = 1312)	353 (26.9%)	136 (61.3%)	74 (33.6%)	23 (22.1%)	12 (5.9%)	13 (7.3%)	52 (28.1%)	43 (21.8%)	0.02
Length of ICU stay (days)	8.3 (3.0–16.9)	7.0 (2.9–14.2)	17.5 (10.1–24.9)	13.1 (4.6–21.7)	9.1 (3.7–16.3)	6.9 (2.8–14.9)	3.7 (2.1–9.7)	3.9 (1.8–9.1)	<0.0001

Data are counts (% of total study) for sample size (*n*), median (IQR) for continuous characteristics, and *n* (% of column group) for categorical characteristics. Units or numerical definitions of characteristics are provided in square brackets. If a characteristic had missing values for some patients in the population, the non-missing sample size was provided in parentheses—e.g., Marshall CT (*n* = 1255). Conventionally, TBI severity is categorically defined by baseline GCS scores as indicated in square brackets. Incidence of epidural haematoma (EDH) or traumatic subarachnoid haemorrhage (tSAH) was assessed from CT scans at ICU admission. *P* values are determined from proportional odds logistic regression (POLR) coefficient analysis trained on all summary characteristics concurrently. For categorical variables with *k* > 2 categories (e.g., Baseline GCS), *P* values were calculated with a likelihood ratio test (with *k*-1 degrees of freedom) on POLR.

### Disease course modelling

**Fig. 1:**
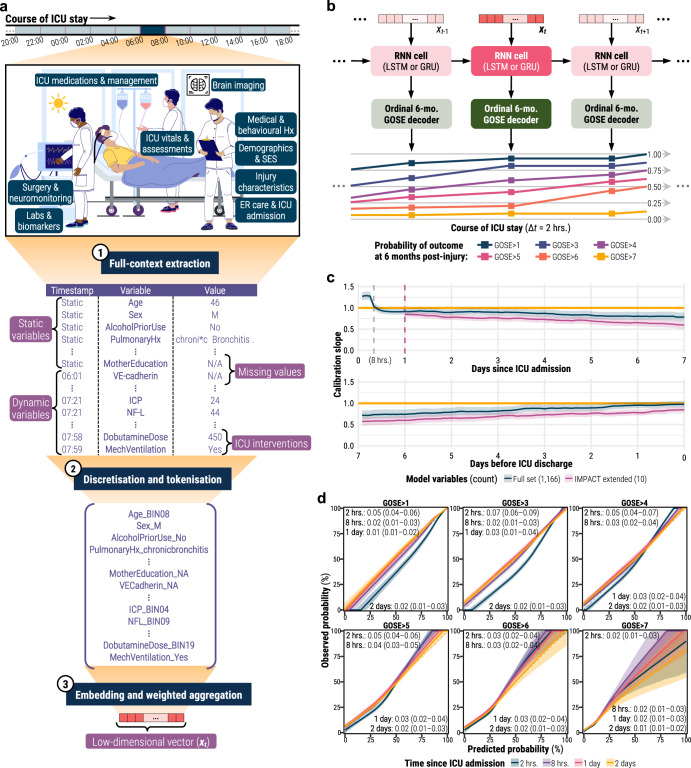
Illustration and reliability of disease course modelling strategy. Unless otherwise specified, all shaded regions surrounding curves are 95% confidence intervals derived using bias-corrected bootstrapping (1000 resamples) to represent the variation across 20 repeated fivefold cross-validation partitions. **a** Tokenisation and embedding of the variables in a sample patient’s ICU stay into a single, low-dimensional vector (*x*_*t*_) per time window. The patient’s ICU stay (sample timeline) was first discretised into non-overlapping, 2-h time windows. From each time window, values for up to 428 dynamic variables were combined with values for up to 738 static variables to form the variable set (Supplementary Note 1). The variable values were converted to tokens by discretising numerical values (e.g., intracranial pressure [ICP] and neurofilament light chain [NF-L]) into 20-quantile bins from the training set and removing special formatting from text-based entries. Through an embedding layer, a vector was learned for each token encountered in the training set, and tokens were replaced with these vectors. Finally, a positive relevance weight, also learned for each token, was used to weight-average the vectors of a time window into a single, low-dimensional vector. The patient stock image is accredited to iStock.com/SiberianArt and was purchased under Standard License. **b** The sequence of low-dimensional vectors (*x*_*t*_) representing a patient’s ICU stay were fed into a recurrent neural network (RNN) with either long short-term memory (LSTM) or gated recurrent unit (GRU) cells. The RNN outputs were then decoded at each time window into an ordinal prognosis of 6-month functional outcome. The level of recovery associated with each threshold of 6-month GOSE is decoded in the heading of Table 1 (e.g., GOSE > 1 represents survival at 6 months post-injury). **c** Probability calibration slope, averaged across the six functional outcome thresholds, in the first (top) and last (bottom) week of ICU stay for models trained on the full variable set (blue) and on the static IMPACT extended set from the first 24 h of ICU stay (red). The ideal calibration slope of one is marked with a horizontal orange line. **d** Ordinal probability calibration curves at four different timepoints after ICU admission. The diagonal dashed line represents the line of perfect calibration. The values in each panel correspond to the mean absolute error (95% confidence interval) between the curve and the perfect calibration line.

**Fig. 2:**
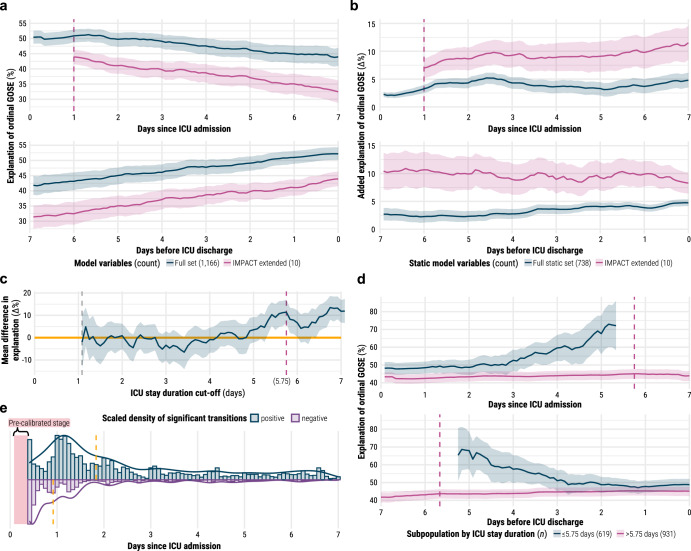
Explanation of outcome by modelling strategy and distribution of high-magnitude transitions. Unless otherwise specified, all shaded regions surrounding curves are 95% confidence intervals derived using bias-corrected bootstrapping (1000 resamples) to represent the variation across 20 repeated fivefold cross-validation partitions. **a** Explanation of ordinal 6-month functional outcome—measured by Somers’ *D*_*xy*_ —in the first (top) and last (bottom) week of ICU stay by models trained on the full variable set (blue) and on the static IMPACT extended set from the first 24 h of ICU stay (red). **b** Added explanation of ordinal 6-month functional outcome—measured by the difference in Somers’ *D*_*xy*_—in the first (top) and last (bottom) week of ICU stay achieved by the full variable model over baseline models trained on all static variables (blue) and on the static IMPACT extended set from the first 24 h of ICU stay (red). **c** Mean difference in full variable model explanation—measured by difference in Somers’ *D*_*xy*_—between subpopulation with ICU stay less than or equal to cut-off and subpopulation with ICU stay greater than the same cut-off. Positive values designate greater explanation in shorter-stay subpopulation, and the horizontal orange line designates no difference. **d** Explanation of ordinal 6-month functional outcome —measured by Somers’ *D*_*xy*_—in the first (top) and last (bottom) week of ICU stay by the full variable model on the subpopulation with ICU stay less than or equal to 5.75 days (blue) and on the subpopulation with ICU greater than 5.75 days (red). **e** Scaled histograms (bin width equals a 2-h time window) and density curves of high-magnitude transitions identified by model trajectories. A high-magnitude transition is defined as a change of output probability that is in the 99th percentile of changes for a specific threshold of 6-month functional outcome. The blue histogram/density represents positive (i.e., improvement) transitions whilst the purple histogram/density represents negative (i.e., worsening) transitions. The dashed orange lines mark the median time since ICU admission for each type of transition.

We developed a modelling strategy to map all 738 static (i.e., fixed at ICU admission) and 428 dynamic (i.e., collected during ICU stay) variables in CENTER-TBI (Supplementary Note 1) to a multidimensional, evolving prognostic trajectory over each patient’s ICU stay. Through supervised learning, our optimised models were trained with three main components: (1) a token-embedding encoder to integrate all variable types and missing values (Fig. 1a), (2) a recurrent neural network (RNN), and (3) an ordinal outcome decoder (Fig. 1b). Since model performance was independent of time window length (Supplementary Fig. 4), we focused on models with 2-h time windows to offer the greatest possible trajectory resolution. With both calibration slope (averaged across the GOSE thresholds, Fig. 1c) and smoothed calibration curves (Fig. 1d), we observed that our modelling strategy required 8 h of information to achieve sufficient calibration for analysis (Fig. 1c). However, after three days post-admission, the calibration slope of both the dynamic and baseline comparison models began decreasing, indicating a slight overfitting for TBI patients with longer ICU stays.

### Explanation of functional outcome

At best, the entire set of 1166 core CENTER-TBI variables combined with our modelling strategy explained 51.3% (95% CI: 49.1–53.3%) of the ordinal variance in 6-month GOSE at 32 h post-admission and 52.2% (95% CI: 50.2–54.3%) at discharge (Fig. 2a). Whilst overall explanation performance consistently decreased after approximately three days post-admission, the added explanation over both baseline comparison models increased over time (Fig. 2b). The additional explanation of the full CENTER-TBI set over the ten IMPACT variables (Supplementary Table 1) increased from 7.0% (95% CI: 5.2–8.7%) at 24 h to 11.5% (95% CI: 8.5–14.5%) at 1 week, and the additional explanation of ICU information increased from 2.1% (95% CI: 1.6–2.5%) at admission to 5.2% (95% CI: 4.2–6.2%) at 1 week. Therefore, at 1 week after admission, the ten IMPACT variables accounted for 73.9% of the explanation of functional outcome achieved by all 1166 CENTER-TBI variables and 82.9% of that achieved by the 738 static variables.

In addition, functional outcome explanation was, on average, 11.4% (95% CI: 6.6–16.9%) greater in patients who stayed in the ICU for 5.75 days or less (*n* = 619) than in those who stayed longer (*n* = 931) (Fig. 2c). Explanation performance was significantly better in shorter-stay patients from ICU admission, but the difference was not significant closer to discharge (Fig. 2d). Longer-stay patients were more likely to have presented with severe TBI, received more intense treatment, and remained alive but severely disabled at 6 months post-injury (Supplementary Fig. 5). Patients who died in the ICU were significantly more likely to have shorter stays (Supplementary Fig. 6).

### Contributions of clinical events to transitions in outcome

In the calibrated analysis region (10 h to 1 week after admission), we found a median of one (IQR: zero–three) high-magnitude transition (as defined in “Methods” and Supplementary Table 2) per patient’s ICU stay. The majority of identified transitions occurred within two days of ICU admission, but clinical worsening transitions occurred earlier than improvement transitions, on average (Fig. 2e).

**Fig. 3:**
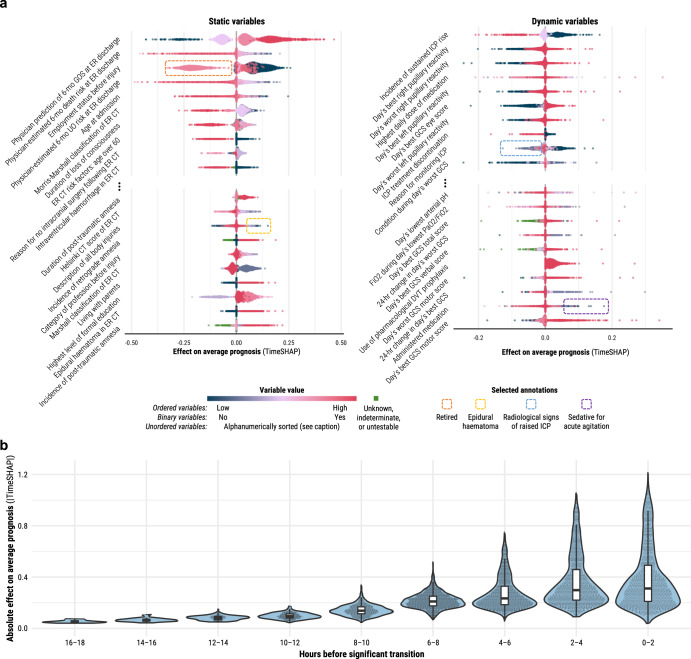
Population-level variable and time window contributions to expected 6-month functional outcome output at high-magnitude transitions. TimeSHAP values are interpreted as contributions of variables or time windows towards the difference in a patient’s expected 6-month functional outcome output from that of the average patient (Supplementary Fig. 13). **a** TimeSHAP values of the 20 highest-contribution static (left) and 20 highest-contribution dynamic (right) variables. The variables were selected by first identifying the ten variables with non-missing value tokens with the most negative median TimeSHAP values across the population (above the ellipses) and then, amongst the remaining variables, selecting the ten with non-missing value tokens with the most positive median TimeSHAP values (below the ellipses). Each point represents the mean TimeSHAP value for a token across an individual patient’s high-magnitude transitions. The colour of the point represents the relative ordered value of a token within a variable, and for unordered variables (e.g., employment status before injury), tokens were sorted alphanumerically (the sort index per possible unordered variable token is provided in the Supplementary Note 1). Green points represent variable tokens that are not missing but explicitly encode an unknown value (i.e., GCS motor score untestable due to sedation). New variable abbreviations include deep vein thrombosis (DVT), the fraction of inspired oxygen (FiO_2_), partial pressure of oxygen (PaO_2_), and unfavourable outcome (UO) as defined by GOSE ≤4 at 6 months post-injury. **b** TimeSHAP amplitude distributions of 2-h time windows leading up to high-magnitude transitions. The width of violin plots is scaled for each time window, but the width of the points inside them demonstrates relative frequency across the windows.

According to the TimeSHAP values^10^ associated with high-magnitude transitions across the population (Fig. 3a), physician-based prognostic estimates at emergency room (ER) discharge had the highest contribution to model trajectories. However, when we retrained the dynamic models without physician-based impressions (Supplementary Note 2), we found no statistically significant drop (95% confidence) in explanation percentage until 1 week after admission: −4.7% (95% CI: −0.2 to −9.2%) (Supplementary Fig. 7). Of the remaining static variables, certain demographic (i.e., employment, age, education, and living situation), CT (i.e., subarachnoid haemorrhage, intraventricular haemorrhage, and epidural haematoma), and clinical presentation (i.e., loss of consciousness and amnesia) variables ranked highest in terms of contribution to model output. For dynamic variables, markers of raised intracranial pressure, neurological function (i.e., pupillary, motor, and verbal reactivity), and administered medication contributed the most. The highest-contributing variables were largely consistent across the 6-month GOSE thresholds (Supplementary Fig. 8), but TimeSHAP amplitudes generally decreased at higher thresholds, and the incidence of mechanical ventilation had a strong negative association with achieving full functional recovery (GOSE > 7). Observing the TimeSHAP values of top-contributing variables per category (Supplementary Fig. 9), we found that whilst certain ER lab measurements (e.g., glucose) had significant contributions, the same lab measurements taken in the ICU did not. When comparing patients receiving withdrawal of life-sustaining treatment (WLST, *n* = 203) with those who did not, TimeSHAP values for models trained with and without physician-based impressions did not reveal a significant difference in clinical events associated with high-magnitude transitions (Supplementary Fig. 10). The TimeSHAP values of missing variables (Supplementary Fig. 11) demonstrated that missingness of a variable could have a significant negative (e.g., missing level of education) or positive (e.g., missing heart rate value at ER admission) effect on model output. Absolute TimeSHAP values of timepoints leading up to a high-magnitude transition (Fig. 3b) suggested that only ICU events that occurred within 10 h before a high-magnitude transition offered considerable contribution to the change in model output.

### Individualised trajectories

**Fig. 4:**
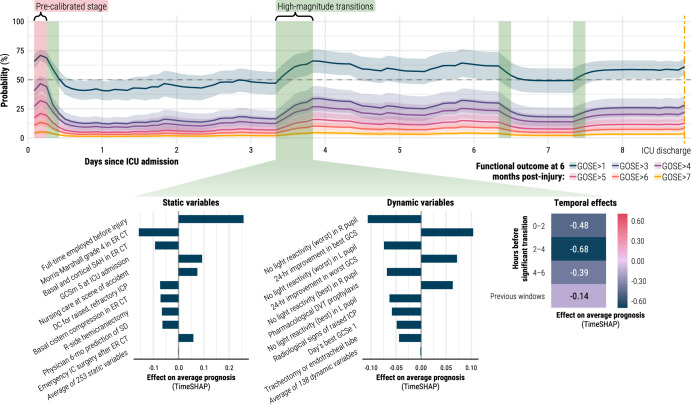
Example of individual ICU disease course with explanations for high-magnitude transition. TimeSHAP values are interpreted as contributions of variables or time windows towards the difference in this patient’s expected 6-month functional outcome output from that of the average patient (Supplementary Fig. 13). The patient was an approximately 50-year-old male, admitted to the ICU after a moderate traumatic brain injury (GCS 10), who became severely disabled (SD) with full functional dependency by 6 months post-injury (GOSE 2 or 3). The patient presented with a subarachnoid haemorrhage (SAH) and received emergency intracranial surgery (IC) and a decompressive craniectomy (DC). New variable abbreviations include deep vein thrombosis (DVT), eye component score of GCS (GCSe), left (L), and right (R).

In Fig. 4, we show the prognostic trajectories for a typical individual in our population. The patient, an approximately 50-year-old male, was admitted to the ICU after a moderate TBI (GCS 10) caused by a traffic collision. The patient survived for at least 6 months after the injury but became severely disabled and completely functionally dependent (GOSE 2 or 3). The models correctly returned low probabilities for all 6-month GOSE thresholds but GOSE > 1, for which the prognostic trajectory oscillates above and below 50% with high-magnitude transitions. In the highlighted positive high-magnitude transition (centre of Fig. 4), we found improvements from the last day’s GCS and the start of pharmacological thromboprophylaxis to be most strongly associated with the improvement in the patient’s condition. The penultimate time window before the transition contributed the most towards the model output. In Supplementary Fig. 12, we also show similarly dynamic individual trajectories for patient cases at each remaining 6-month GOSE score.

## Discussion

In this work, we develop a dynamic, data-driven approach to exploit the full clinical context available in the CENTER-TBI dataset and produce individual trajectories of TBI disease course. Notably, our modelling strategy required minimal data processing for a large set of variables and imposed no constraints on the number or type of variables per patients (Fig. 1a)^11^. Moreover, by including missing value tokens, models discovered meaningful patterns of missingness (Supplementary Fig. 11)^12^. Finally, our approach detailed clinical events in terms of prognostic transitions on ordinal levels of functional recovery (Fig. 1b), which is an improvement in statistical power and clinical information over using a dichotomised outcome (e.g., mortality)^8^. Our modelling strategy can potentially be valuable in other heterogenous data-intensive domains in medicine to: (1) qualify information in a dataset, (2) explore high-magnitude transitions in individuals, or (3) automate state-space characterisation for applications in reinforcement learning^13^ and individualised treatment effect estimation^14^.

Our principal finding is that the full range of 1,166 core CENTER-TBI variables explained up to 52.2% (95% CI: 50.2–54.3%) of the variance in ordinal, 6-month functional outcome (Fig. 2a). Up to 90.9% (95% CI: 90.3–91.3%) of this explanation was derived from static (i.e., pre-ICU or admission) information, which constituted ~80% of the variables in the average patient time window (Supplementary Fig. 2). Over time, the dynamic (i.e., collected during ICU stay) information increased explanation over the baseline (Fig. 2b), though not enough to compensate for poorer overall performance in longer-stay (>5.75 days in ICU) patients. These patients more likely experienced severe TBI and received intense treatment without early discharge (Supplementary Figs. 5 and 6 and as described previously^15^), and our results suggest greater, unexplained variation in recovery for this subpopulation. Remarkably, the ten IMPACT variables covered 86.3% of the explanation achieved by all 1166 CENTER-TBI variables at 24 h post-admission.

The greater outcome influence of static over dynamic information has several plausible explanations which could guide future TBI research and clinical management. Amongst high-resource ICUs, the variation in TBI treatment strategies has previously been shown not to result in a commensurate variation in functional outcome^15^. Since ICU treatments for TBI are mostly effective in mitigating secondary insults^3^, primary injury severity and pre-ICU circumstances may account for greater outcome differences amongst ICU patients. These explanations support both the continued use of baseline prognosis to guide treatment planning in existing practice^8^ and a paradigm shift in future practice towards targeted treatment of primary injury mechanisms^2^. Moreover, the information currently collected in the ICU may not sufficiently capture pathophysiological changes that take place from the acute stage of TBI^1^. This motivates the development of more precise ICU metrics inspired by scientific discoveries of longitudinal TBI effects. Furthermore, 6-month GOSE may not reflect the full contribution of ICU clinical course towards functional recovery. Upper levels of GOSE have been shown not to discriminate cognitive function well^16^, and GOSE may require repeated measurements to account adequately for day-to-day variation in questionnaire responses during recovery^6^. A multidimensional measure of TBI outcome, which integrates assessments of mental, cognitive, and physical health over time, may be preferable to 6-month GOSE in revealing specific contributions of ICU events^17^. Finally, a fraction of functional outcome is likely to be explained by variations in rehabilitative care and longer-term sequalae of TBI^18^. Therefore, extending data collection past ICU discharge may reveal outcome contributions of dynamic information overlooked in our study.

The data-driven results also highlight several avenues to help account for the remaining half of functional outcome explanation. Amongst potential static variables for inclusion, genetic factors (not available for this study) are the most promising as they have been shown to explain up to 26% of the variation in dichotomised GOSE^19^. Whilst the exclusion of physician-based impressions did not significantly worsen GOSE explanation until 1 week post-admission (Supplementary Fig. 7), the relatively high contribution of these impressions (Fig. 3a) merits an investigation into the extent to which they affect self-fulfilling prophecies^20^ or simply summarise other clinical variables^21^. For dynamic variables, the logical first step would be to test the inclusion of high-resolution time series—both routinely collected (e.g., cerebral perfusion pressure^22^) and experimental (e.g., accelerometry^23^)—that have been shown to correlate with pathophysiological changes after TBI. The fact that clinical assessments of pupillary reactivity and the GCS rank amongst the highest-contributing dynamic variables (Fig. 3a) may encourage the development of methods that more precisely characterise neurological mechanisms underpinning reactivity. At the same time, the relatively high information coverage of the ten IMPACT variables may suggest that the existing CENTER-TBI set could be made more concise for prognosis-based characterisation.

There are two important considerations when understanding the results of this study. First, TimeSHAP values on observational data are merely associative and cannot be interpreted for causal inference. For example, the consistently positive contribution of pharmacological thromboprophylaxis (Fig. 3a) is likely explained more by the clinical selection of patients with reduced risk of intracranial bleeding for such treatment than by the effect of the treatment itself^24^. Moreover, TimeSHAP is not sufficient for developing clinical rules as it does not reveal important variable interactions^25^. We used TimeSHAP in this work not to derive claims but rather to highlight potential areas of investigation from a wider, data-driven approach, even if many of these associations may be confounded. For instance, employment status had a strong model contribution in this (Fig. 3a) and prior work;^8^ whether that is due to an indication of frailty (shown to be associated with lower GOSE, regardless of age^26^) or a spurious association may be worth exploring. Second, we strongly advise against using our models for clinical outcome prediction. Our explanatory modelling strategy was designed for mining patient trajectories from observational datasets and is not deployable for real-time prediction due to concerns of self-fulfilling prophecies, generalisability, and variable robustness. We refer readers interested in dynamic TBI prediction model development to the following studies^22,27,28^.

We recognise several additional limitations in this study. Our modelling strategy discretised both numerical variables into binned tokens and time into windows, which caused some loss of information. To bypass the discretisation of time, neural differential equations^29^ may be a suitable alternative to RNNs but still require greater validation in medical problems. In addition, our definition of high-magnitude transitions based on a percentile cut-off of model outputs was ultimately arbitrary. We encourage investigators either to try other percentiles or assess TimeSHAP values at known clinical events and transitions. Finally, our results may encode recruitment, collection, and clinical biases native to our European patient set and may not generalise to other populations^30^. We encourage investigators to apply our modelling strategy to other longitudinal, granular datasets of critically ill TBI patients—particularly in low- and middle-income countries where the burden of TBI is disproportionately higher^31^—and compare their results.

## Methods

### Study design and participants

CENTER-TBI is a longitudinal, observational cohort study (NCT02210221) involving 65 medical centres across 19 European countries^5,9^. TBI patients were prospectively recruited between December 19, 2014 and December 17, 2017 if they met the following criteria: (1) presentation within 24 h of a TBI, (2) clinical indication for a CT scan, and (3) no severe pre-existing neurological disorder. In accordance with relevant laws of the European Union and the local country, ethical approval was obtained for each site, and written informed consent by the patient or legal representative was documented electronically. The study sites, ethical committees, approval numbers, and approval dates are listed in Supplementary Table 3. The project objectives and design of CENTER-TBI have been described in detail previously^5,9^.

In this work, we apply the following inclusion criteria in addition to those of CENTER-TBI: (1) primary admission to the ICU for at least 24 h, (2) at least 16 years old and (3) availability of functional outcome assessment at 6 months post-injury.

### Variables and functional outcome

We extracted all variables collected before and during ICU stays for the CENTER-TBI core study^9^ (v3.0, ICU stratum) using Opal database software^32^. These variables were sourced from medical records and online test results and include structured (i.e., numerical, binary, or categorical), unstructured (i.e., free text), and missing values. We manually excluded variables which explicitly indicate death or withdrawal of life-sustaining treatment (Supplementary Note 3). In total, we included 1166 variables (Supplementary Note 1): 738 static (i.e., fixed at ICU admission) variables and 428 dynamic variables (i.e., collected during ICU stay). We organised the variables into the nine categories listed in Table 2 and further indicated whether the variables represented an ICU intervention or a physician-based impression. The highest resolution amongst regularly collected variables was once every 2 h.

**Table 2:** Variable count per category and subtype.

Category	Example variable	Count by subtypes				
		All	Static	Dynamic	Interventions	Physician impressions
Demographics and socioeconomic status	Lives with parents	22	22	0	0	0
Medical and behavioural history	Takes beta blockers	188	188	0	0	0
Injury characteristics and severity	Helmet on during accident	84	84	0	0	0
Emergency care and ICU admission	Physician prognosis at ER discharge	246	246	0	0	16
Brain imaging reports	Midline shift	186	108	78	0	25
Laboratory measurements	Glial fibrillary acidic protein	228	81	147	0	1
ICU medications and management	Fluid loading	108	3	105	75	17
ICU vitals and assessments	Bihourly systolic blood pressure	67	0	67	0	0
Surgery and neuromonitoring	Decompressive craniectomy	37	6	31	7	18
Total		1166	738	428	82	77

Data represent the number of subtype (column) variables per category (row).

In addition, we extracted the eight-point, ordinal GOSE functional outcome score at 6 months post-injury (heading of Table 1). Since CENTER-TBI does not distinguish vegetative patients (GOSE = 2) into a separate category, GOSE scores 2 and 3 (lower severe disability) were combined to one category (GOSE∈{2,3}). For 12.8% of our study patients, 6-month GOSE scores were previously imputed by CENTER-TBI using a Markov multi-state model based on the observed GOSE scores recorded at different timepoints between 2 weeks to 1 year post-injury^33^.

### Modelling strategy

We created 100 partitions of our patient population for repeated *k*-fold cross-validation (20 repeats, fivefold), stratified by 6-month GOSE, with validation sets nested within training sets.

Our explanatory modelling strategy is outlined in Fig. 1 and builds upon our previous work^8,11^. We started by partitioning ICU stays into non-overlapping time windows of either 2, 8, 12 or 24 h. Static variables were carried forward across all windows (Fig. 1a). All variables were tokenised through one of the following methods: (1) for categorical variables, appending the value to the variable name, (2) for numerical variables, learning the training set distribution and discretising into either 3, 4, 5, 7, 10 or 20-quantile bins, (3) for text-based entries, removing all special characters, spaces, and capitalisation from the text and appending to the variable name, and (4) for missing values, creating a separate token to designate missingness (Fig. 1a). By labelling missing values with separate tokens instead of imputing them, the models could learn potentially significant patterns of missingness and integrate a diverse range of missing data without needing to validate the assumptions of imputation methods on each variable. During training, the models learned a low-dimensional vector (of either 16, 32, 64 or 128 units) and a “relevance” weight for each token in the training set. Therefore, models would take the unique tokens from each time window of a patient, replace them with the corresponding vectors, and average the vectors—weighted by relevance—into a single vector per time window (Fig. 1a).

Each patient’s sequence of low-dimensional vectors then fed into a RNN—either long short-term memory (LSTM) or gated recurrent unit (GRU)—to output another vector per time window. In this manner, the models learned temporal patterns of variable interactions from training set ICU records and updated outputs with each new time window of data. Finally, each RNN output vector was decoded—either with a multinomial (i.e., softmax) or ordinal (i.e., constrained sigmoid) output layer—to return a probability at each threshold of 6-month GOSE over time (Fig. 1b).

The combinations of hyperparameters—in addition to those already mentioned (time window length, quantile bin count, embedding vector dimension, RNN type, and output layer)—and their optimisation results are reported in the Supplementary Methods.

### Model and information evaluation

All metrics, curves, and associated confidence intervals (CIs) were calculated on the testing sets using the repeated Bootstrap Bias Corrected Cross-Validation (BBC-CV) method^34^. We calculated metrics and CIs at each timepoint after ICU admission as well as at each timepoint leading up to ICU discharge.

The reliability of model-generated trajectories was assessed through the calibration of output probabilities at each threshold of 6-month GOSE. Using the logistic recalibration framework^35^, we first measured calibration slope. Calibration slope less(/greater) than one indicates overfitting(/underfitting)^35^. In addition, we examined smoothed probability calibration curves to detect miscalibrations that might have been overlooked by the logistic recalibration framework^35^.

We also assessed the information quality achieved by the combination of our modelling strategy and the CENTER-TBI variables by calculating Somers’ *D*_*xy*_^36^. In our context, Somers’ *D*_*xy*_ is interpreted as the proportion of ordinal variation in 6-month GOSE that is explained by the variation in model output^37^. The calculation of Somers’ *D*_*xy*_ is detailed in the Supplementary Methods.

We compared the performance of our modelling strategy with that of two baseline models on the same remaining patients over time. The first was a multinomial logistic regression model trained on the ten, static IMPACT extended variables (Supplementary Table 1) from the first 24 h of ICU stay (i.e., the validated standard for static prognosis)^8^. The second was our developed modelling strategy but trained only on the 738 static variables in CENTER-TBI to measure added explanation by ICU information.

### High-magnitude transition identification and explanation

Within the calibrated region of testing set model outputs, we found high-magnitude transitions, both negative (i.e., worsening) and positive (i.e., improvement), of model-generated probabilities at each threshold of 6-month GOSE. High-magnitude transitions were arbitrarily defined by a consecutive time window difference in probability that:

1. for negative transitions, was less than or equal to the 1st percentile of negative differences for a given GOSE threshold across the population,
2. for positive transitions, was greater than or equal to the 99th percentile of positive differences for a given GOSE threshold across the population.

The cut-offs for high-magnitude transitions are listed in Supplementary Table 2.

To uncover the variables associated with high-magnitude transitions, we applied the TimeSHAP algorithm^10^. TimeSHAP estimates the relative contribution of both tokens and time windows towards an individual’s model output by perturbing the clinical events leading up to a high-magnitude transition. TimeSHAP applies a temporal coalition pruning algorithm which groups low-contributing time windows in the distant past together as a single feature (otherwise, the calculation would be computationally intractable given the number of tokens).

At the timepoints of high-magnitude transition, we calculated TimeSHAP for contributions towards both the threshold probability of 6-month GOSE and the expected 6-month GOSE index (Supplementary Fig. 13 and the Supplementary Methods). For the TimeSHAP baseline, we defined an “average patient” to be one with tokens that are in 50^+^% of training set time windows. Therefore, TimeSHAP values were interpreted as associative contributions of tokens or timesteps towards the difference in a patient’s model output from that of the average patient.

### Reporting summary

Further information on research design is available in the Nature Research Reporting Summary linked to this article.

### Supplementary information

Supplementary Information

Reporting Summary

## Data Availability

Individual participant data, including data dictionary, the study protocol and analysis scripts are available online, conditional to the approved study proposal, with no end date. Interested investigators must submit a study proposal to the management committee at https://www.center-tbi.eu/data. Signed confirmation of a data access agreement is required, and all access must comply with regulatory restrictions imposed on the original study.

## Code availability

All code used in this project can be found at the following online repository: https://github.com/sbhattacharyay/dynamic_GOSE_model (10.5281/zenodo.7668551).
