# Supplementary material for: Mining the contribution of intensive care clinical course to outcome after traumatic brain injury
Source: NPJ Digit Med. 2023 Aug 21;6:154. doi: 10.1038/s41746-023-00895-8 (PMC10442346; doi:10.1038/s41746-023-00895-8)
Supplement: Supplementary file 1 — Supplementary Information [file 41746_2023_895_MOESM1_ESM.pdf]

Supplementary Information

TABLE OF CONTENTS

Supplementary Figures **2**

Supplementary Figure 1. Flow diagram for patient enrolment and follow-up. *2*

Supplementary Figure 2. Per-patient token information over the first month of ICU stay. *2*

Supplementary Figure 3. Proportion of study population remaining over the first month of ICU stay. *3*

Supplementary Figure 4. Effect of time-window length on explanation of ordinal six-month functional outcome. *3*

Supplementary Figure 5. Characteristics of study population remaining over first week of ICU stay. *3*

Supplementary Figure 6. Proportion of study population, stratified by ICU survival, remaining over the first month of ICU stay. *4*

Supplementary Figure 7. Effect of physician-based impressions on explanation of ordinal six-month functional outcome *4*

Supplementary Figure 8. Population-level TimeSHAP values across levels of six-month functional recovery. *5*

Supplementary Figure 9. Population-level TimeSHAP values for each category of variables. *9*

Supplementary Figure 10. TimeSHAP values stratified by WLST and inclusion of physician impressions. *14*

Supplementary Figure 11. Population-level TimeSHAP values for missing value tokens. *16*

Supplementary Figure 12. Example of individual ICU disease course for each level of six-month functional outcome. *17*

Supplementary Figure 13. Expected six-month functional outcome *20*

Supplementary Tables **21**

Supplementary Table 1. IMPACT extended model variables stratified by six-month functional outcome. *21*

Supplementary Table 2. Calculated cut-offs to define high-magnitude transitions. *21*

Supplementary Table 3. CENTER-TBI study sites, ethical committees, approval numbers, and approval dates. *22*

Supplementary Notes **25**

Supplementary Note 1. Variables by category. *25*
Demographics and socioeconomic status 25
Medical and behavioural history 26
Injury characteristics and severity 41
Emergency care and ICU admission 46
Brain imaging reports 64
Laboratory measurements 83
ICU medications and management 98
ICU vitals and assessments 105
Surgery and neuromonitoring 108

Supplementary Note 2. Physician-based impression variables. *111*

Supplementary Note 3. Manually excluded variables indicating death or withdrawal of life-sustaining treatment. *115*

Supplementary Note 4. The CENTER-TBI investigators and participants. *118*

Supplementary Methods **122**

Hyperparameter optimisation report *122*
Summary 122
Overview of tested hyperparameters 122
Tested hyperparameters per model version 123

Calculation of Somers' D_{xy} *129*

Supplementary References **130**

SUPPLEMENTARY FIGURES

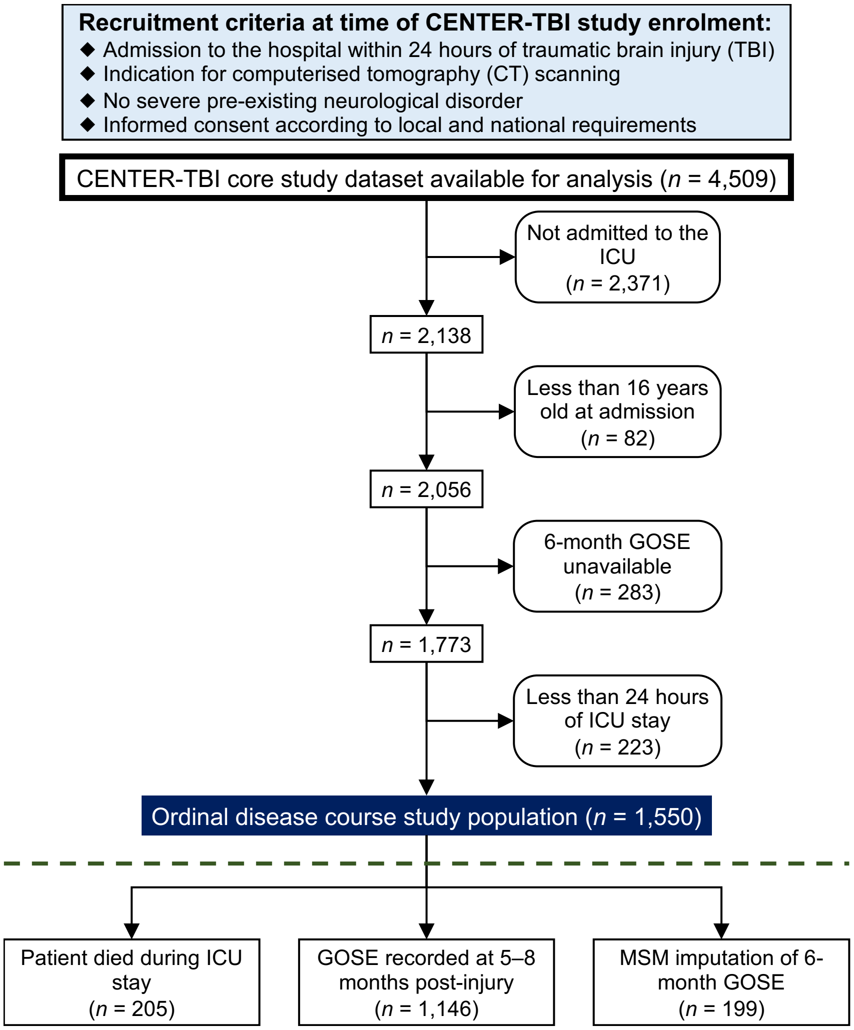

Supplementary Figure 1. Flow diagram for patient enrolment and follow-up. The dashed, olive-green line in the lower-middle of the diagram divides the enrolment flow diagram (above) and the follow-up breakdown (below). Imputation of missing six-month GOSE values with a Markov multi-state model (MSM) is described in the Methods.

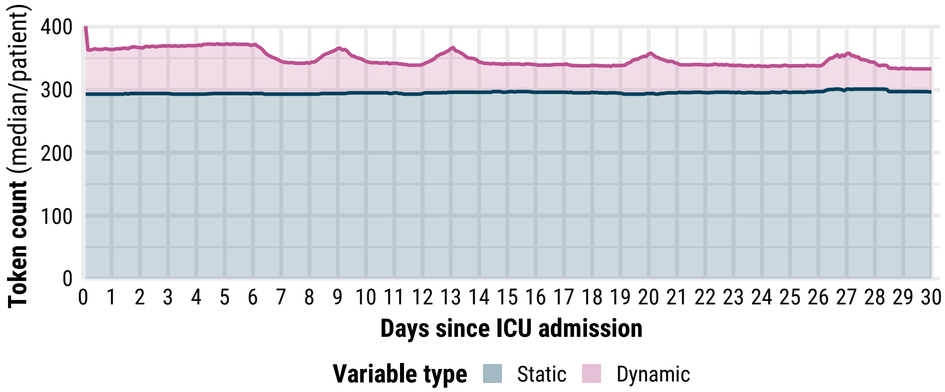

Supplementary Figure 2. Per-patient token information over the first month of ICU stay. The blue and purple lines represent the median number of non-missing value tokens of static and dynamic variables, respectively, per patient's time window over the first month of ICU stay.

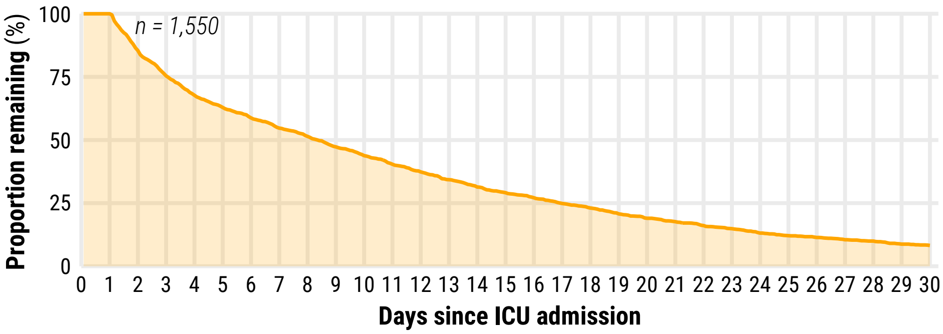

Supplementary Figure 3. Proportion of study population remaining over the first month of ICU stay.

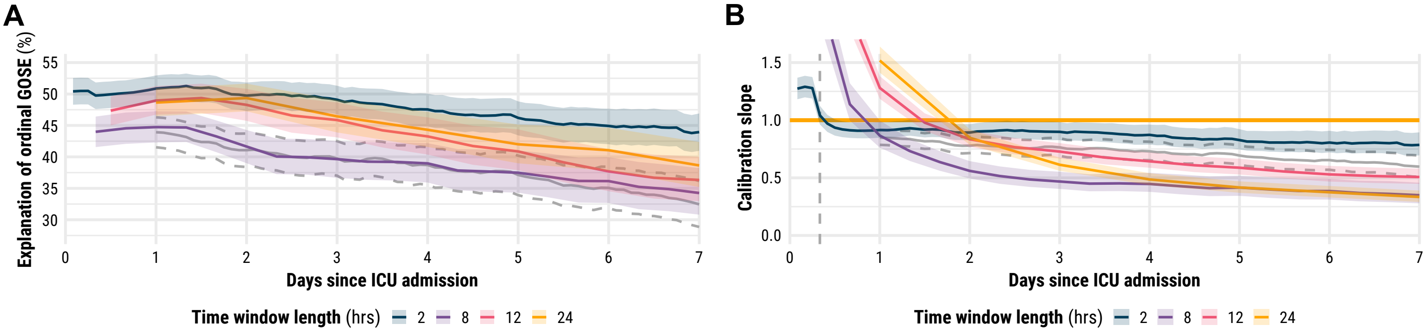

Supplementary Figure 4. Effect of time-window length on explanation of ordinal six-month functional outcome. The shaded regions surrounding curves are 95% confidence intervals derived using bias-corrected bootstrapping (1,000 resamples) to represent the variation across 20 repeated five-fold cross-validation partitions. (**A**) Model explanation of ordinal six-month functional outcome, measured by Somers' D_{xy}, in the first week of ICU stay. The grey line (with 95% confidence interval in dashed lines) is the explanation achieved by a static model trained on ten validated variables from the first 24 hours of ICU stay. (**B**) Model probability calibration slope, averaged across the six functional outcome thresholds, in the first week of ICU stay. The ideal calibration slope of one is marked with a horizontal orange line. The grey line (with 95% confidence interval in dashed lines) is the average calibration slope of a static model trained on ten validated predictors from the first 24 hours of ICU stay.

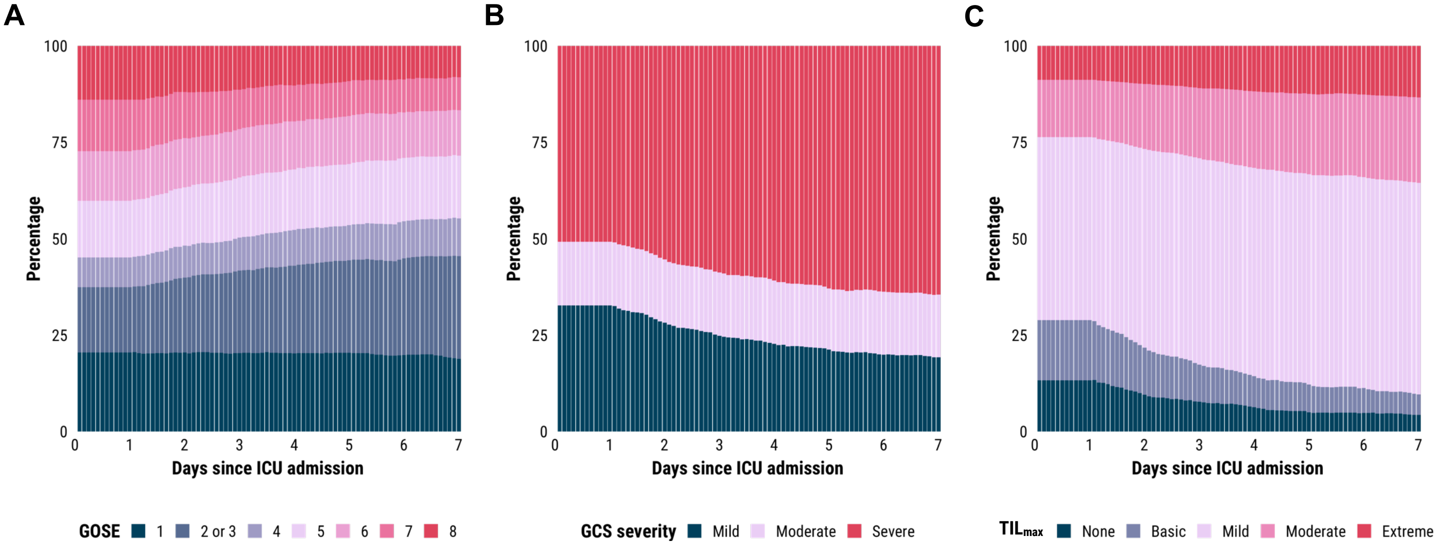

Supplementary Figure 5. Characteristics of study population remaining over first week of ICU stay. (**A**) The eight, ordinal scores of GOSE are decoded in the heading of Table 2. (**B**) GCS is categorised into severity as follows: Mild=13–15, Moderate=9–12, and Severe=3–8. (**C**) Maximum therapy intensity level over ICU stay (TIL_{max}) is categorised into severity as follows: None=0, Basic=1–2, Mild=3–11, Moderate=12–18, and Extreme=19–38.

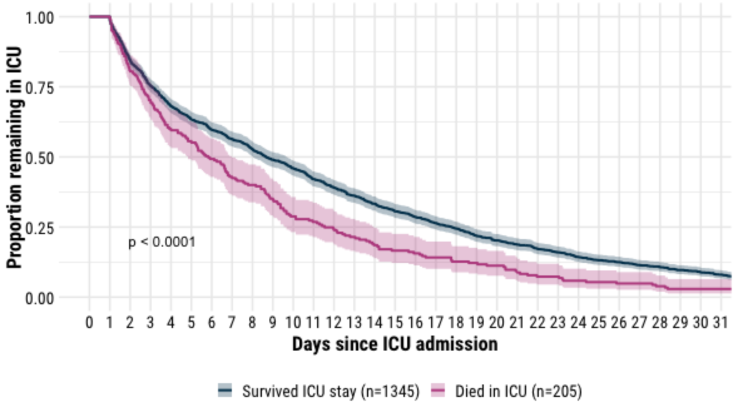

Supplementary Figure 6. Proportion of study population, stratified by ICU survival, remaining over the first month of ICU stay. The shaded regions surrounding curves are 95% confidence intervals and *p*-values are derived from a log-rank test.

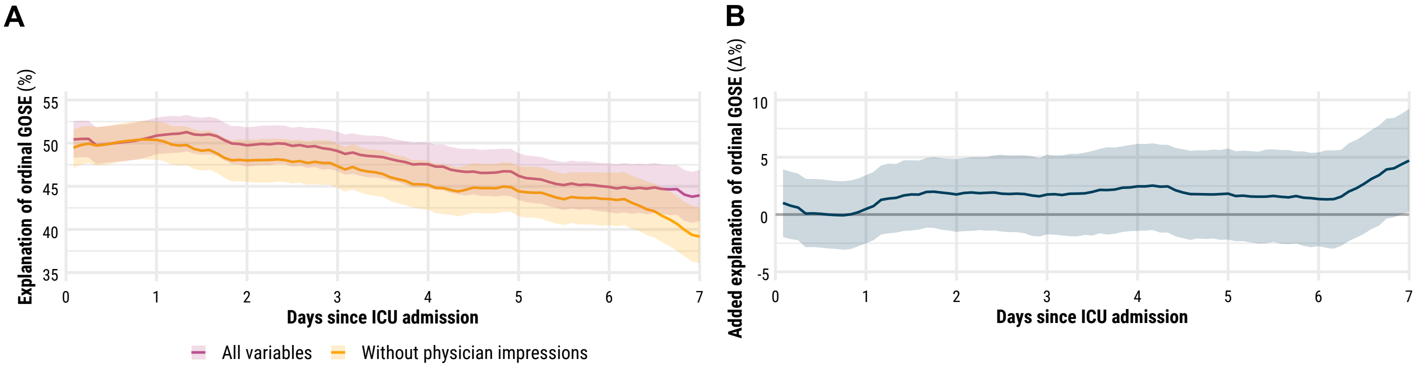

Supplementary Figure 7. Effect of physician-based impressions on explanation of ordinal six-month functional outcome. The shaded regions surrounding curves are 95% confidence intervals derived using bias-corrected bootstrapping (1,000 resamples) to represent the variation across 20 repeated five-fold cross-validation partitions. All physician-based impression variables are listed in Supplementary Note 2. (**A**) Model explanation of ordinal six-month functional outcome, measured by Somers' D_{xy}, in the first week of ICU stay. (**B**) Added model explanation of ordinal six-month functional outcome is measured by difference in Somers' D_{xy} in the first week of ICU stay.

Supplementary Figure 8. Population-level TimeSHAP values across levels of six-month functional recovery. Legend provided at end of figure (pp 8).

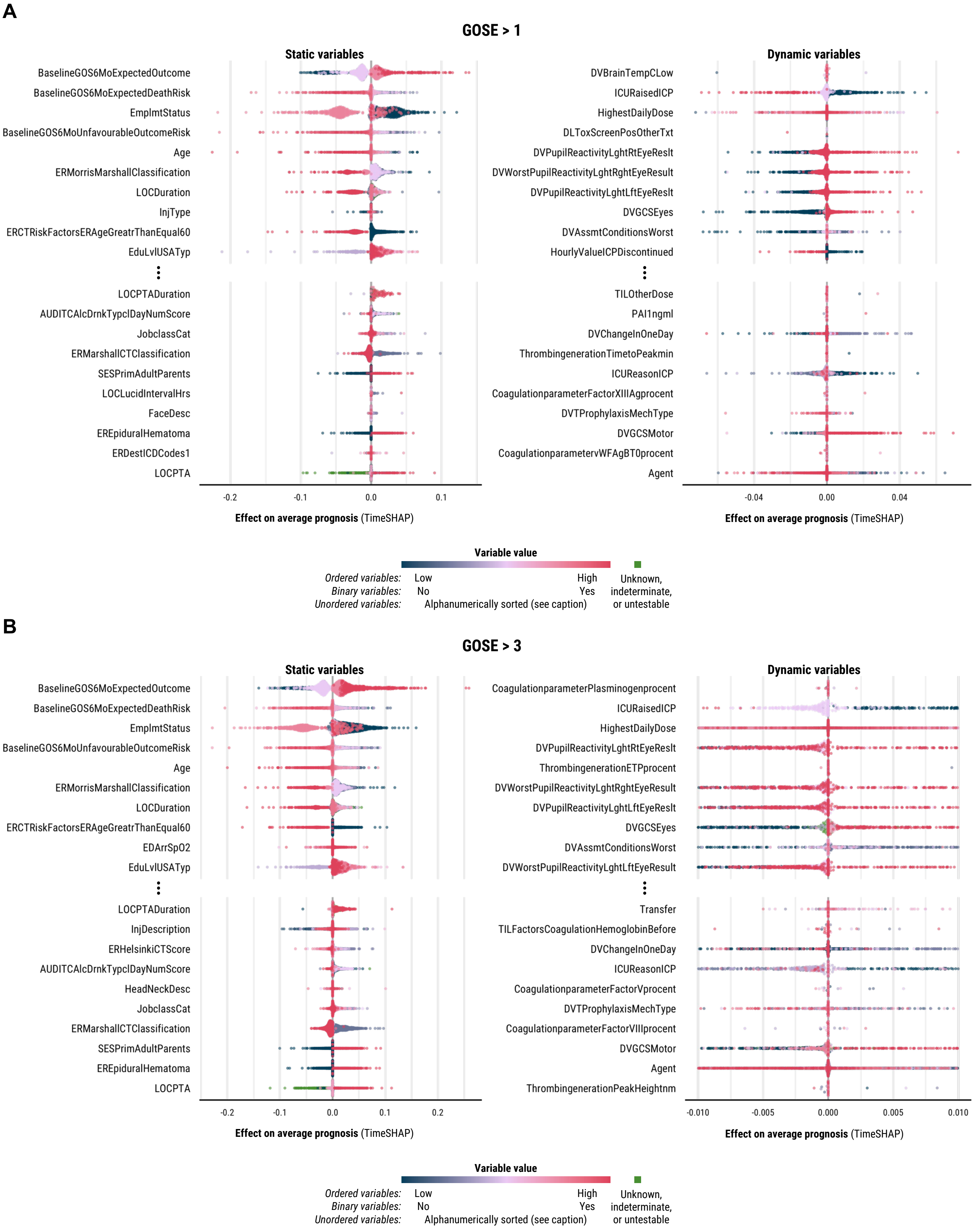

Supplementary Figure 8 (*continued*).

C

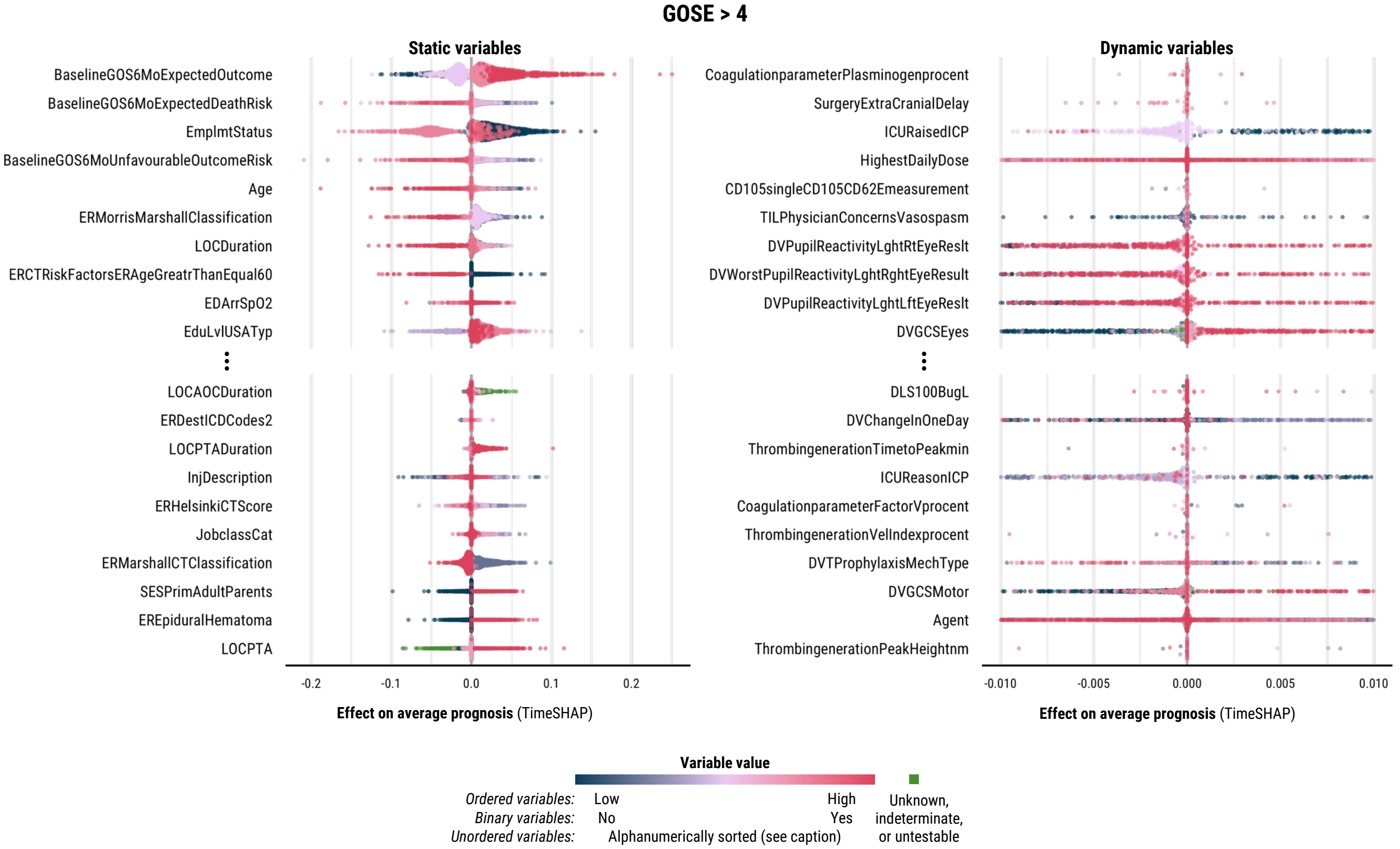

D

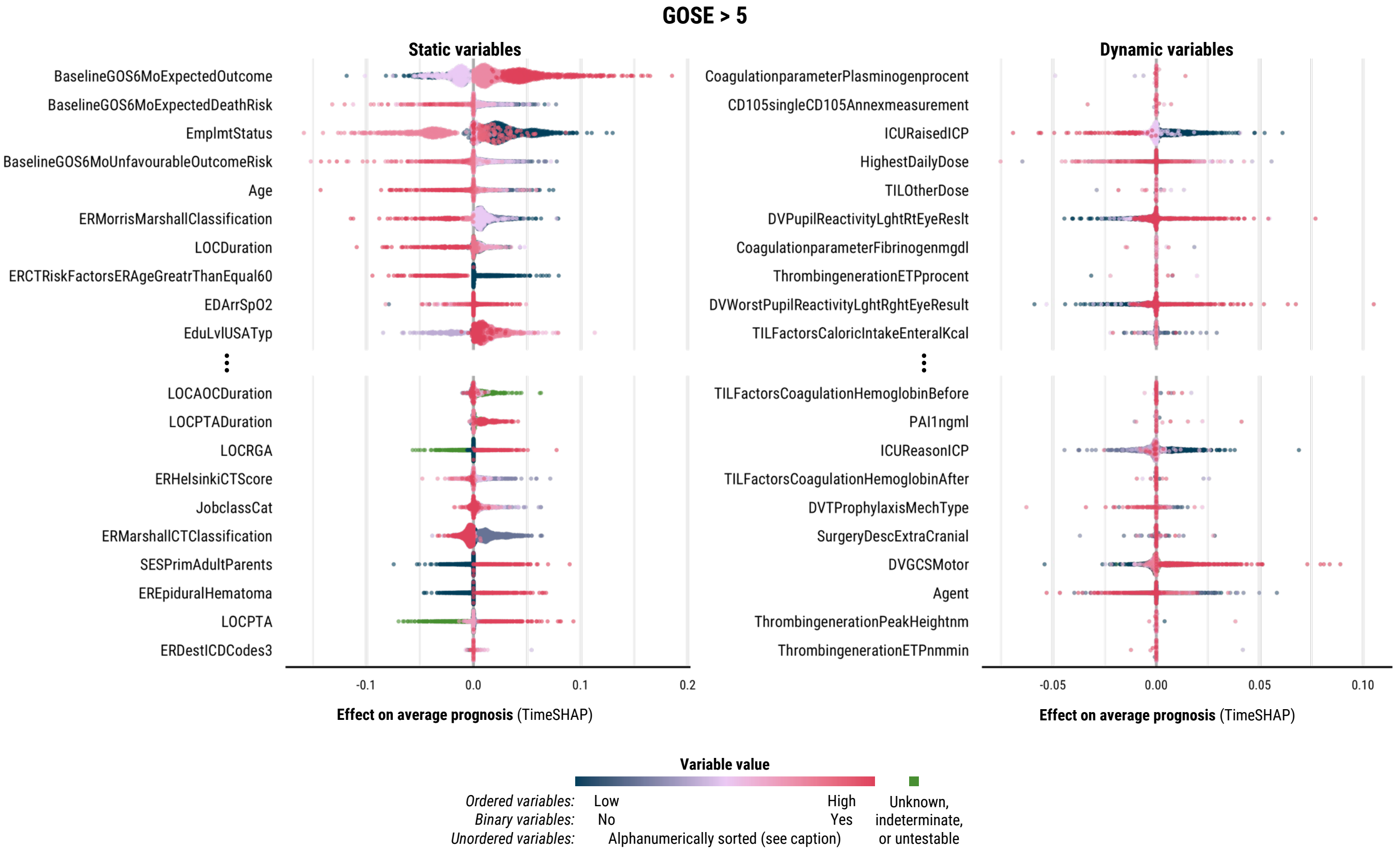

Supplementary Figure 8 (*continued*).

E

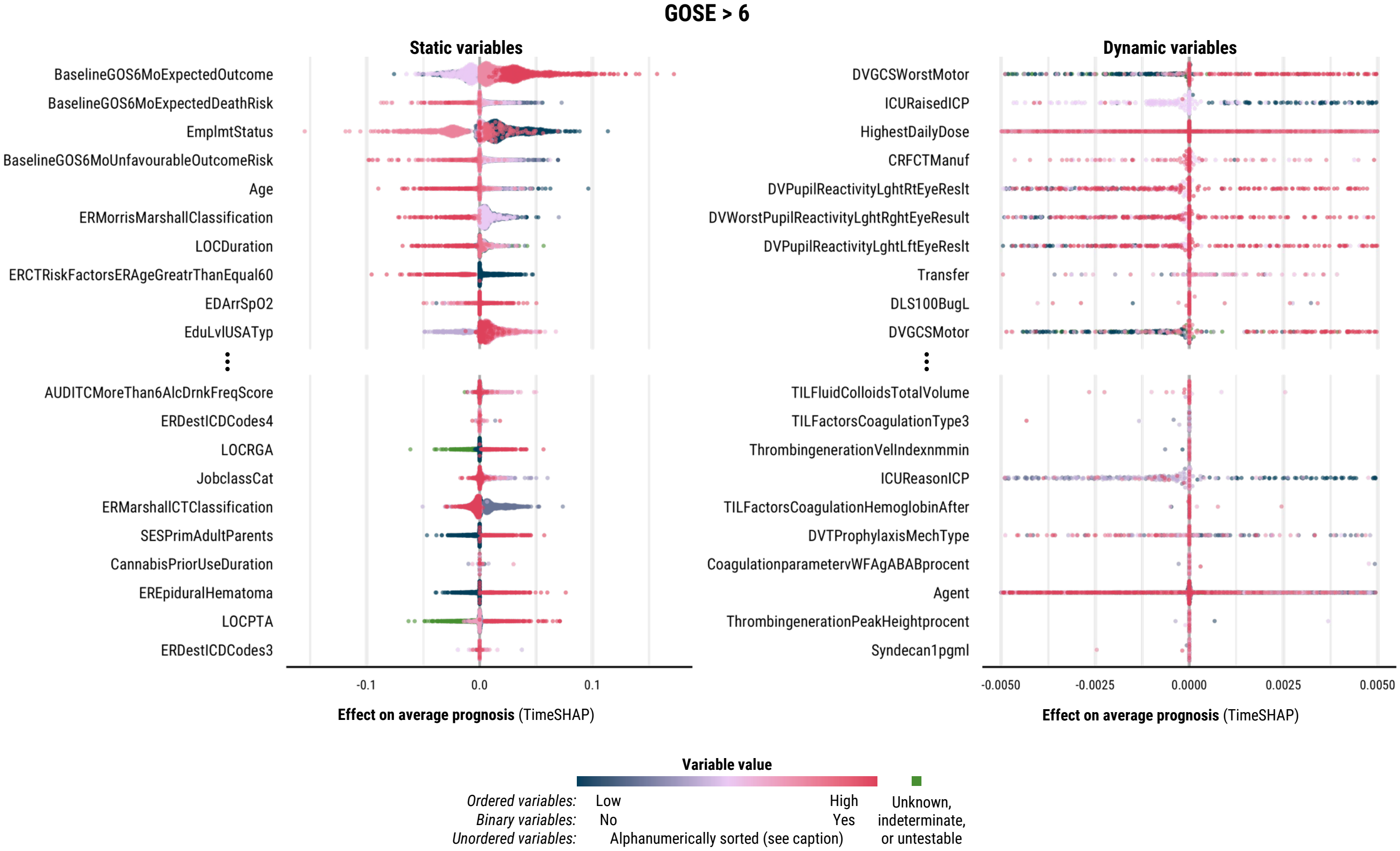

F

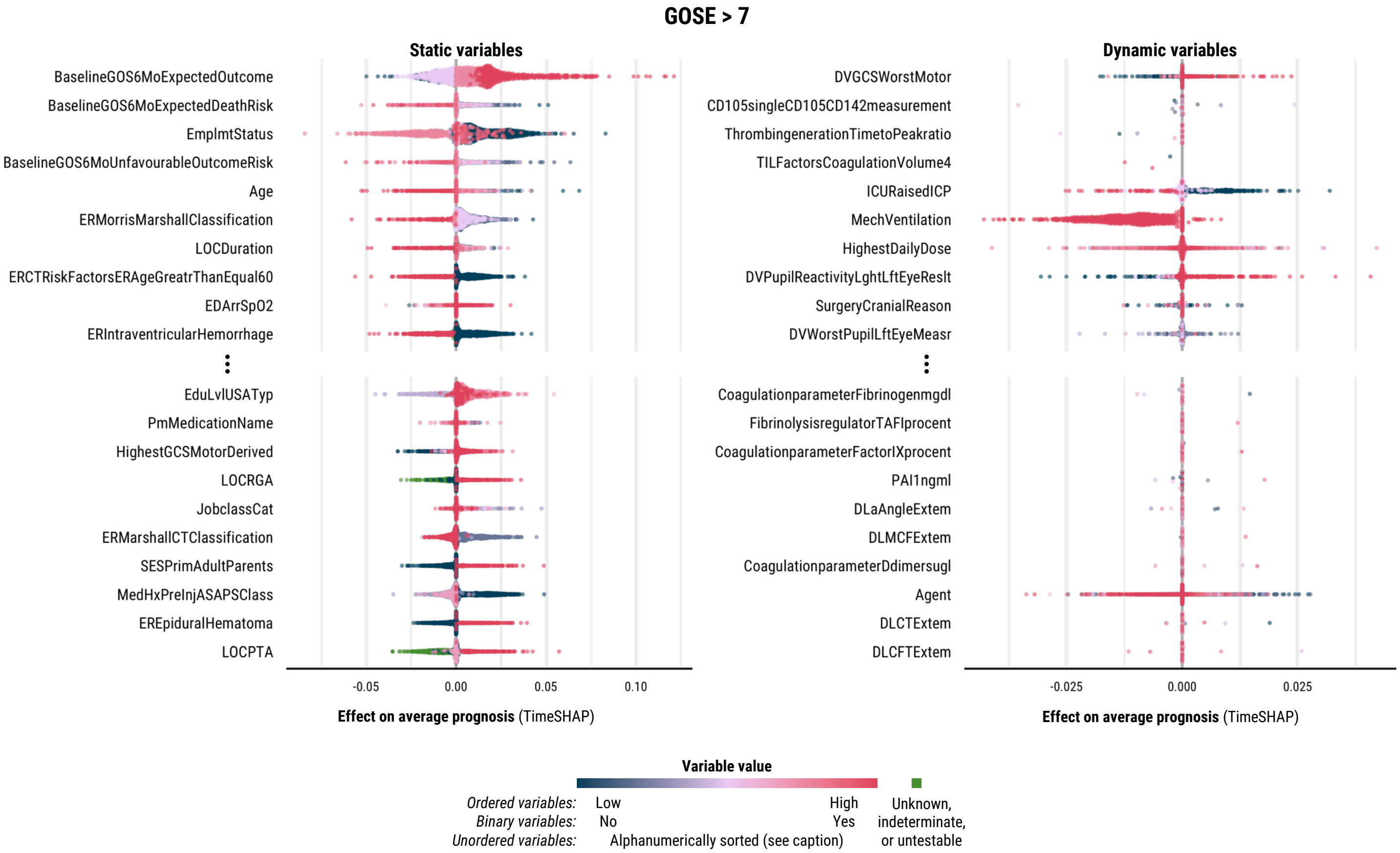

Supplementary Figure 8. Population-level TimeSHAP values across levels of six-month functional recovery. All abbreviated variable names are decoded in Supplementary Note 1. TimeSHAP values are interpreted as contributions of variables or time windows towards the difference in a patient's probability at each threshold of six-month GOSE from that of the average patient. The variables were selected – for each threshold of GOSE – by first identifying the ten variables with non-missing value tokens with the most negative median TimeSHAP values across the population (above the ellipses) and then, among the remaining variables, selecting the ten with non-missing value tokens with the most positive median TimeSHAP values (below the ellipses). Each point represents the mean TimeSHAP value for a token across an individual patient's high-magnitude transitions. The colour of the point represents the relative ordered value of a token within a variable, and for unordered variables (e.g., employment status before injury), tokens were sorted alphanumerically (the sort index per possible unordered variable token is provided in Supplementary Note 1). Green points represent variable tokens that are not missing but explicitly encode an unknown value. The TimeSHAP values are provided for each threshold of GOSE: (**A**) GOSE>1, i.e., survival, (**B**) GOSE>3, i.e., regaining consciousness, (**C**) GOSE>4, i.e., regaining independence, (**D**) GOSE>5, i.e., regaining ability to participate in one or more life roles, (**E**) GOSE>6, i.e., symptomatic return to normal life, and (**F**) GOSE>7, i.e., full return to normal life.

Supplementary Figure 9. Population-level TimeSHAP values for each category of variables. Legend provided at end of figure (pp 13).

A

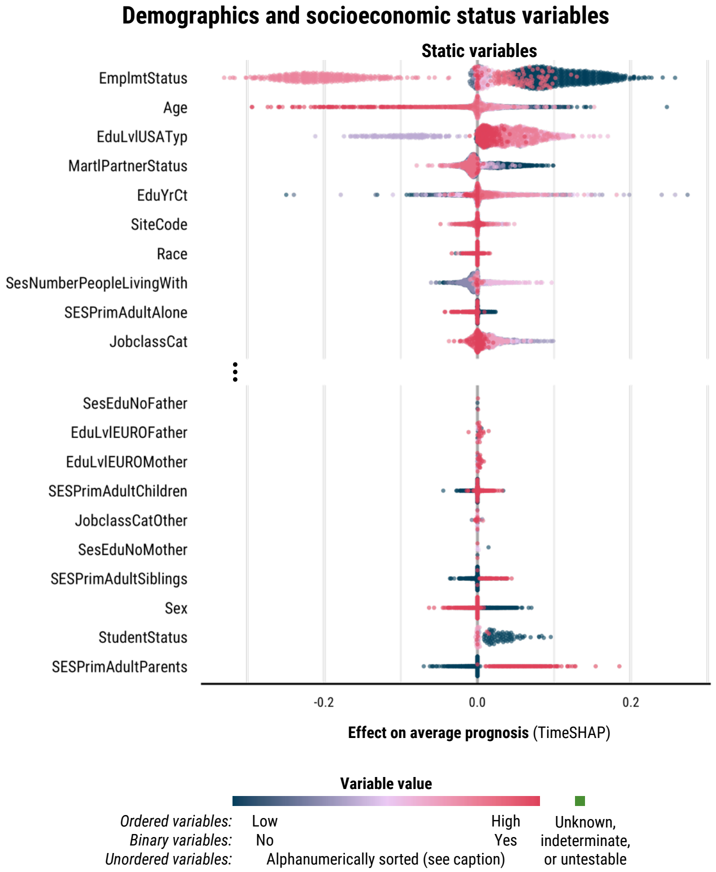

B

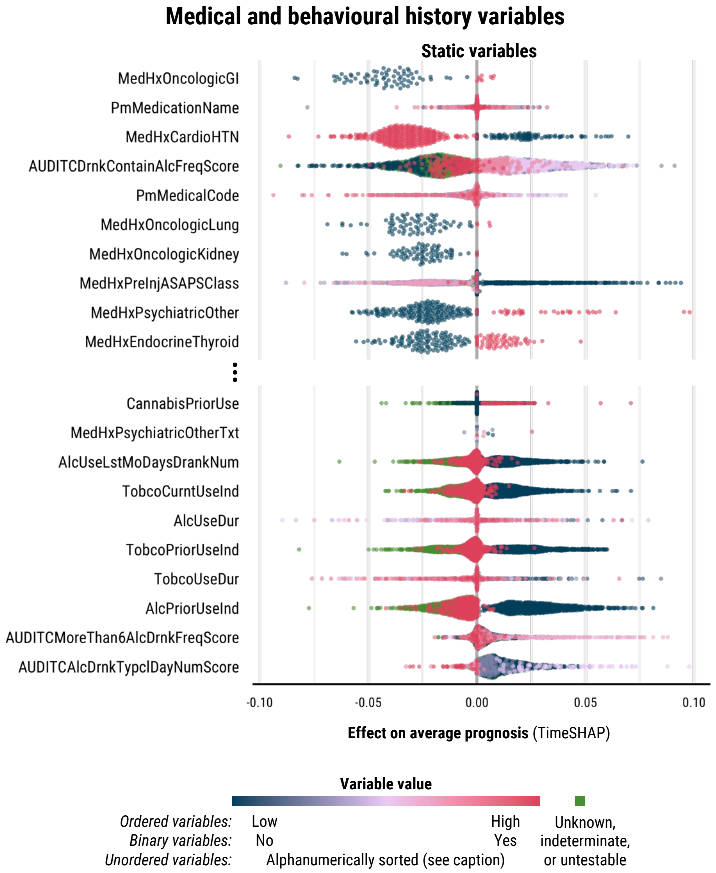

Supplementary Figure 9 (*continued*).

C

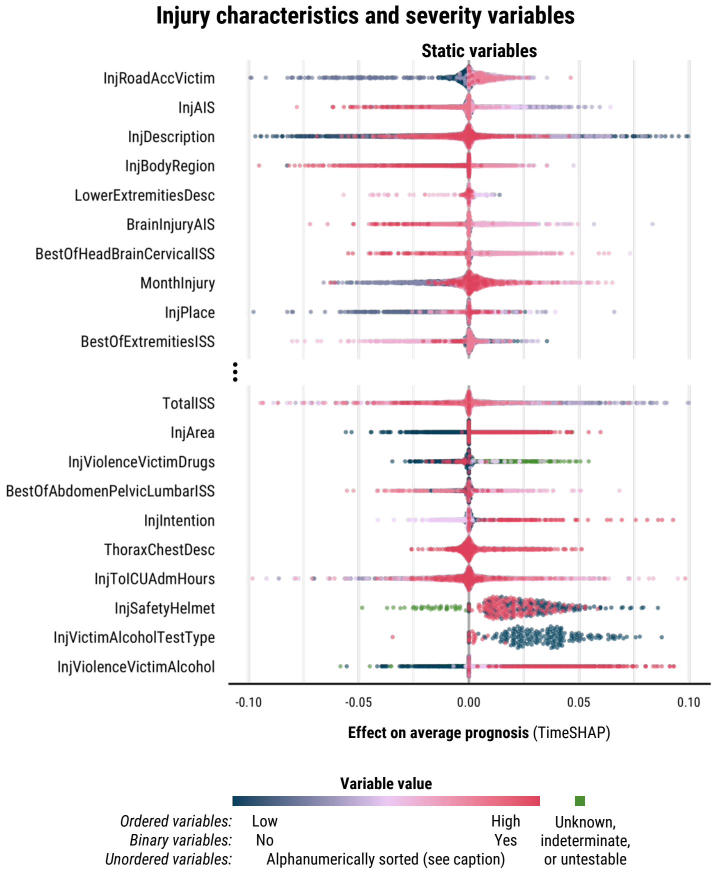

D

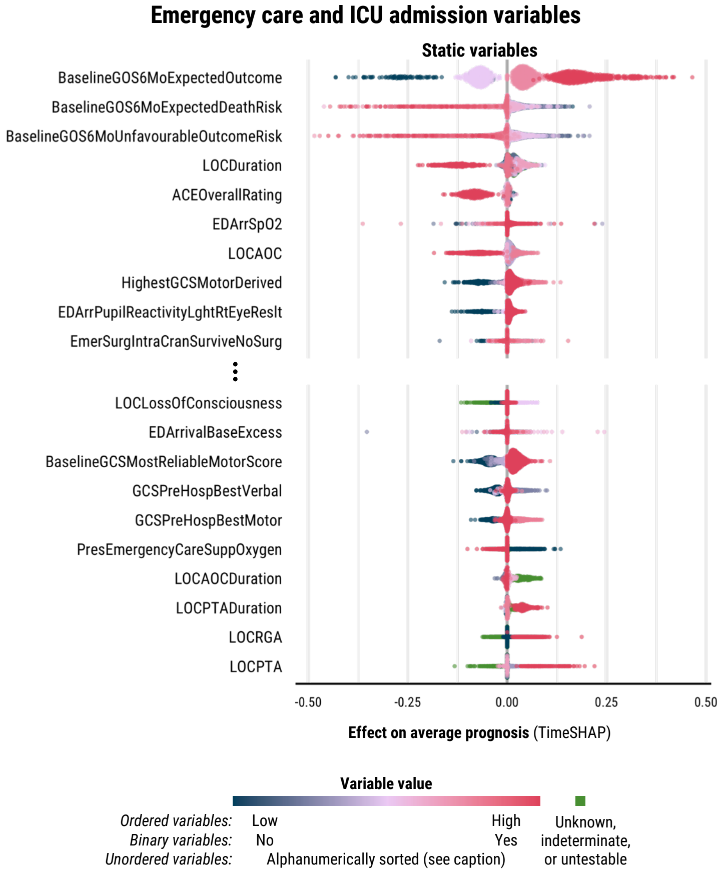

Supplementary Figure 9 (*continued*).

E

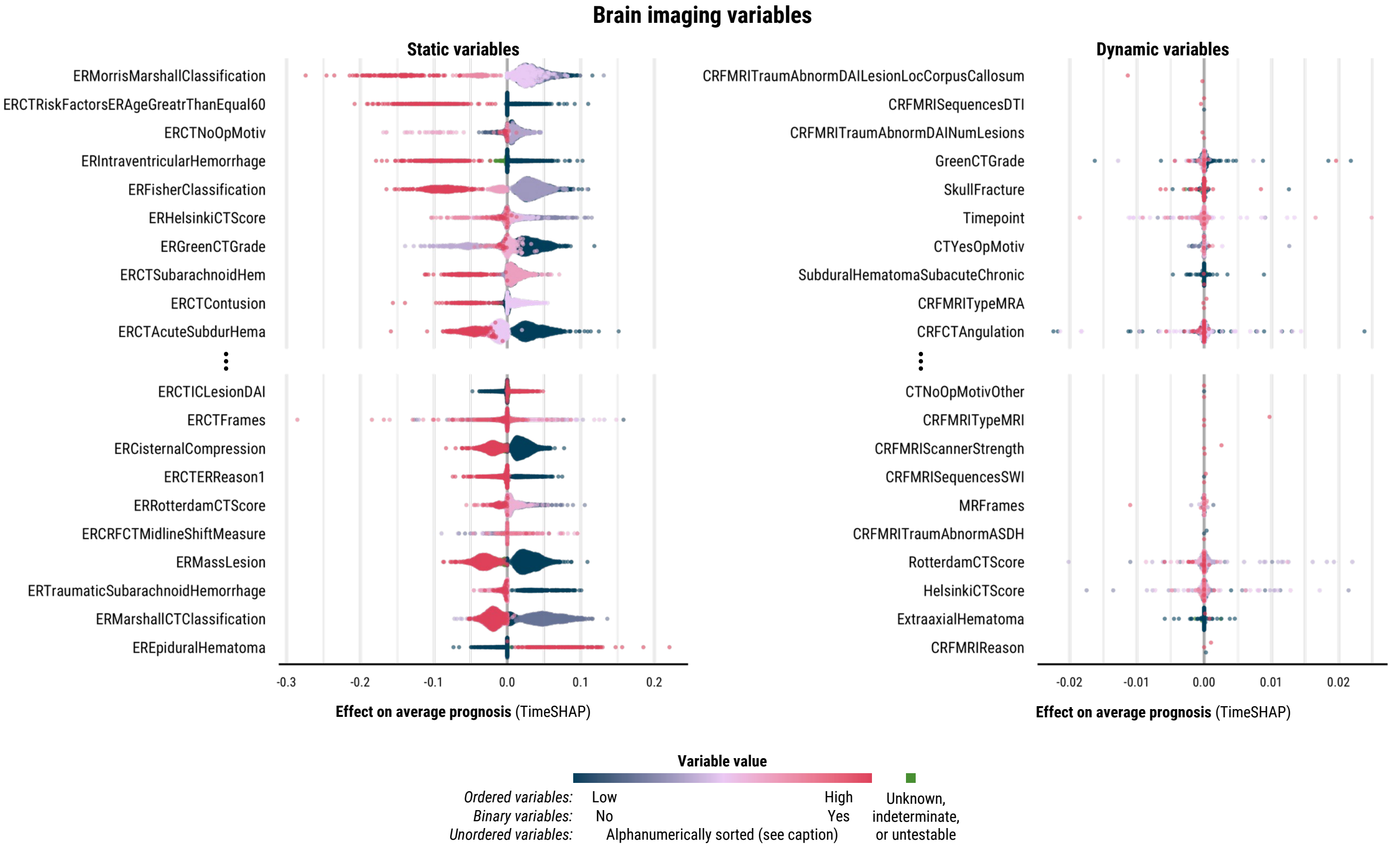

F

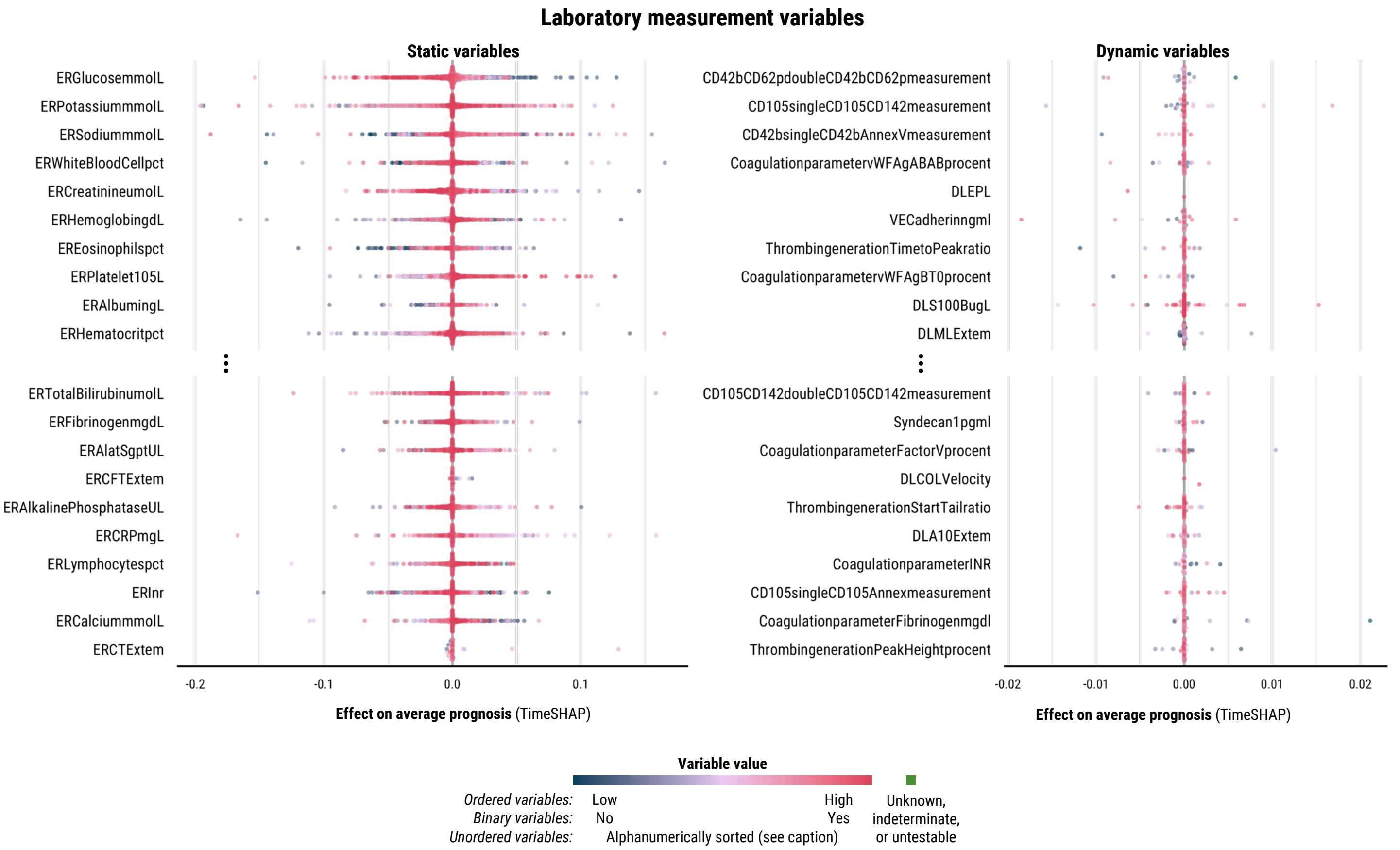

Supplementary Figure 9 (*continued*).

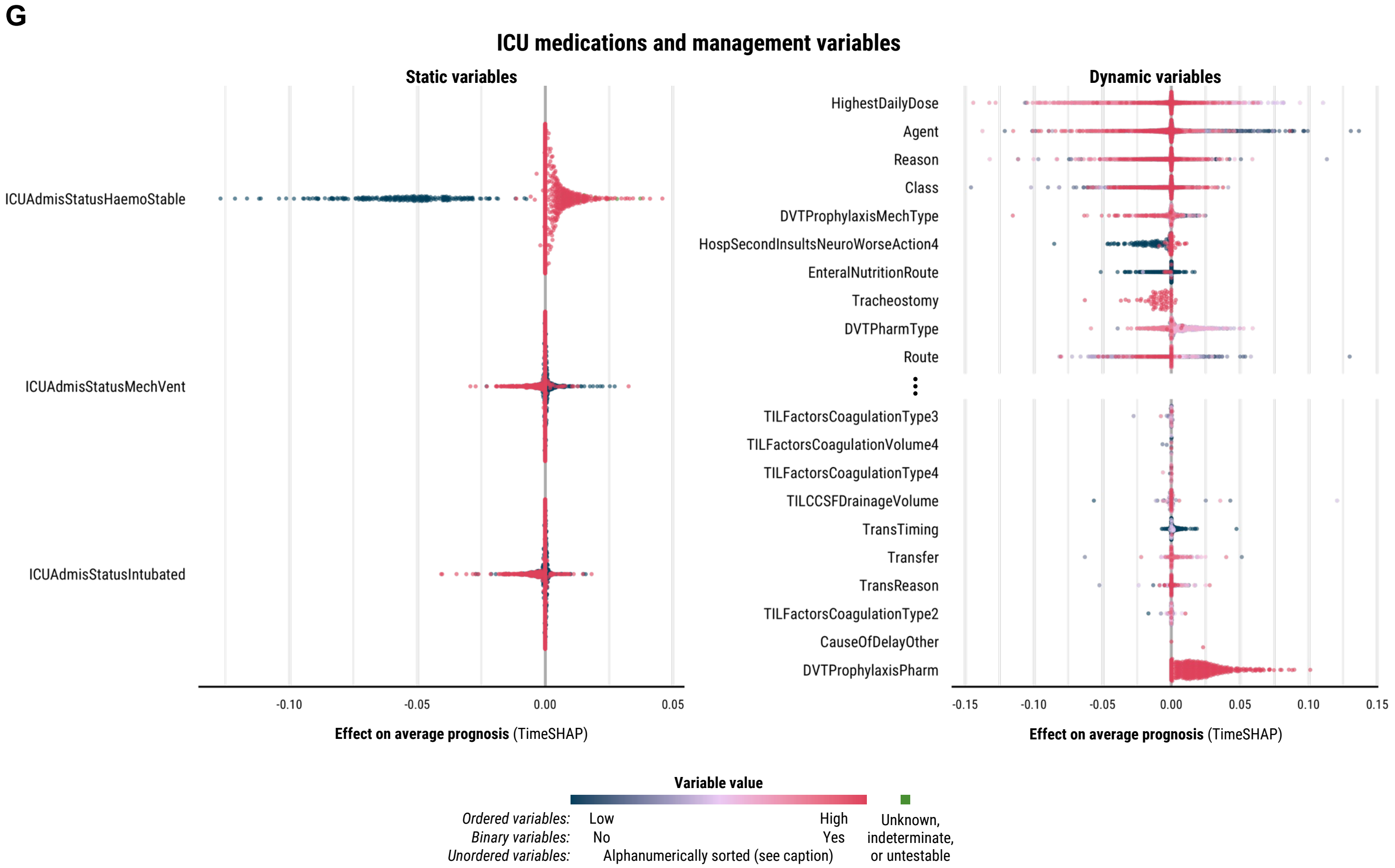

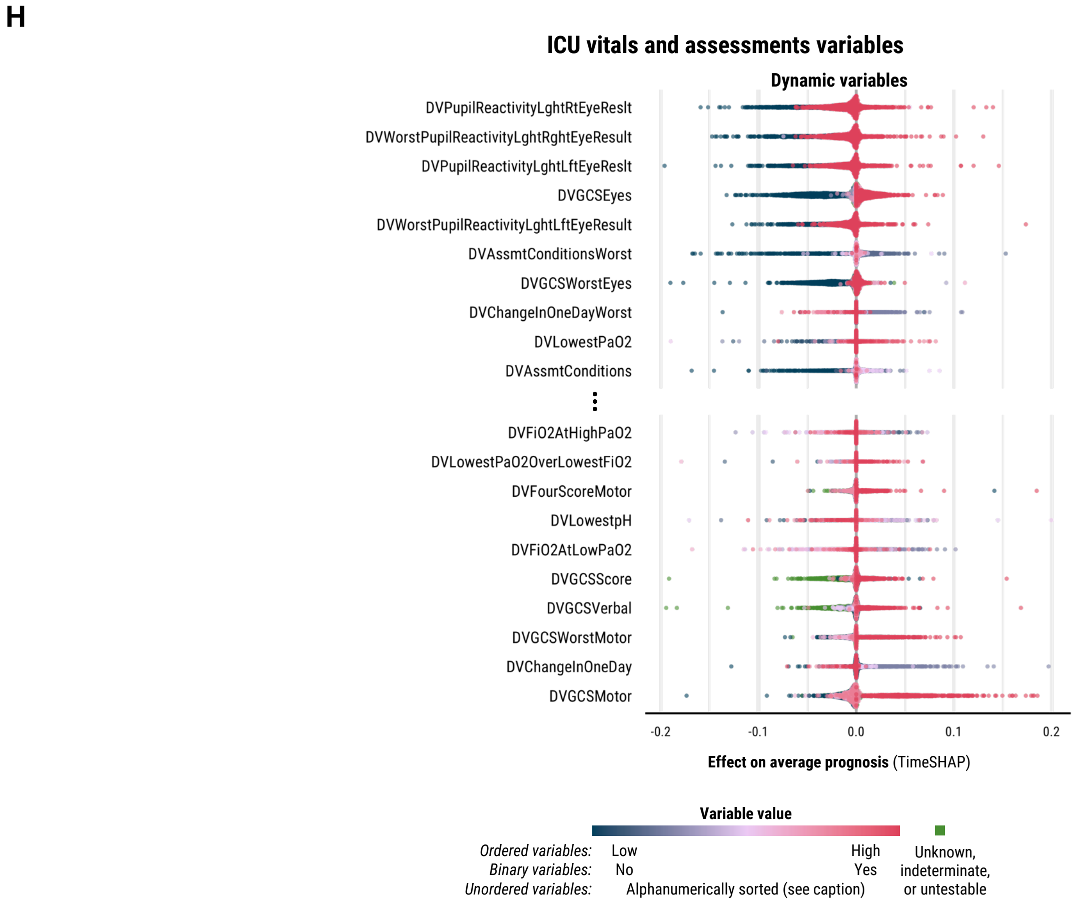

Supplementary Figure 9 (*continued*).

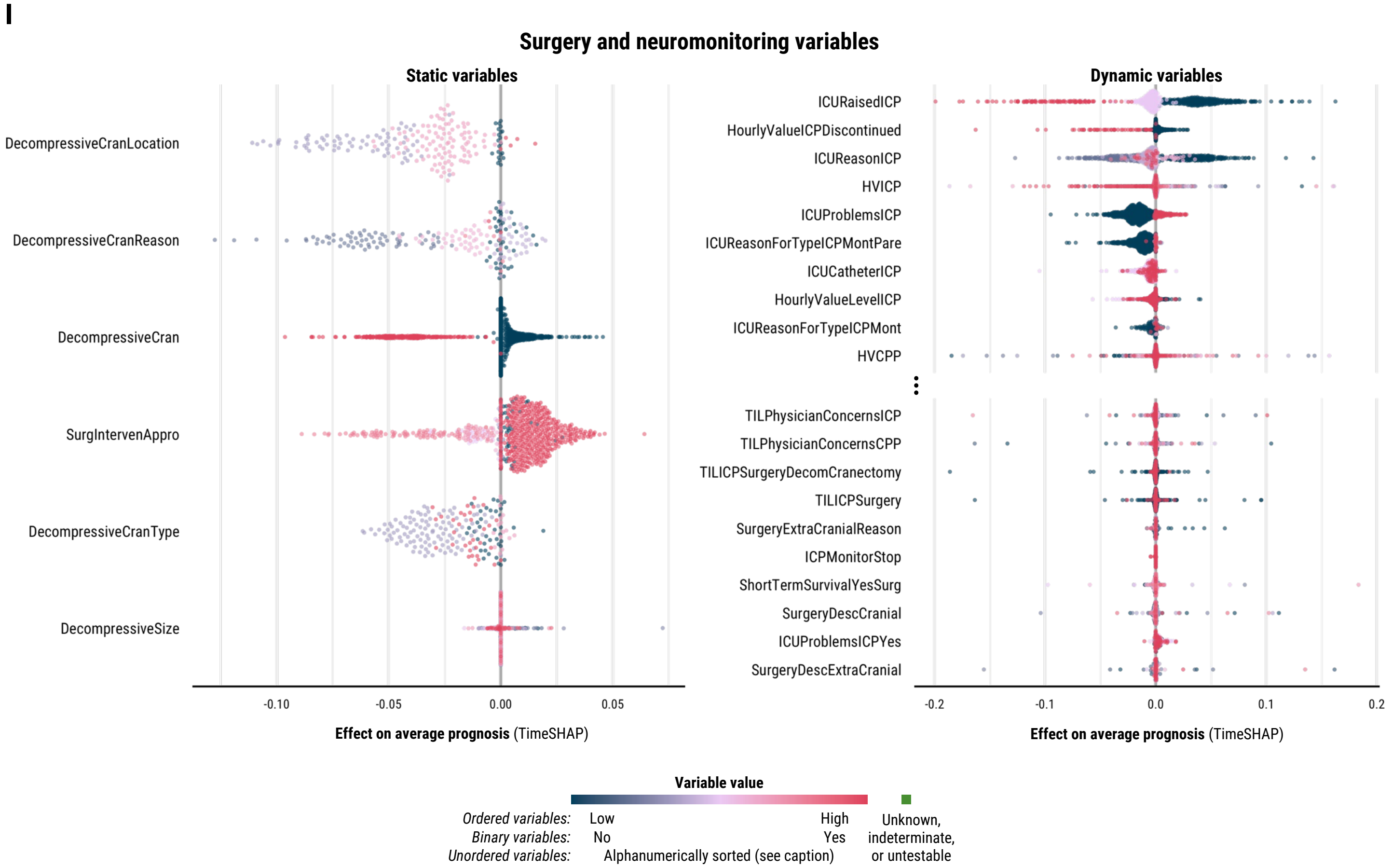

Supplementary Figure 9. Population-level TimeSHAP values for each category of variables. All abbreviated variable names are decoded in Supplementary Note 1. TimeSHAP values are interpreted as contributions of variables towards the difference in a patient's expected six-month functional outcome output from that of the average patient (Supplementary Figure 13). The variables were selected – for each category of variables – by first identifying the ten variables with non-missing value tokens with the most negative median TimeSHAP values across the population (above the ellipses) and then, among the remaining variables, selecting the ten with non-missing value tokens with the most positive median TimeSHAP values (below the ellipses). Each point represents the mean TimeSHAP value for a token across an individual patient's high-magnitude transitions. The colour of the point represents the relative ordered value of a token within a variable, and for unordered variables (e.g., employment status before injury), tokens were sorted alphanumerically (the sort index per possible unordered variable token is provided in Supplementary Note 1). Green points represent variable tokens that are not missing but explicitly encode an unknown value.

Supplementary Figure 10. TimeSHAP values stratified by WLST and inclusion of physician impressions. Legend provided at end of figure (pp 16).

A

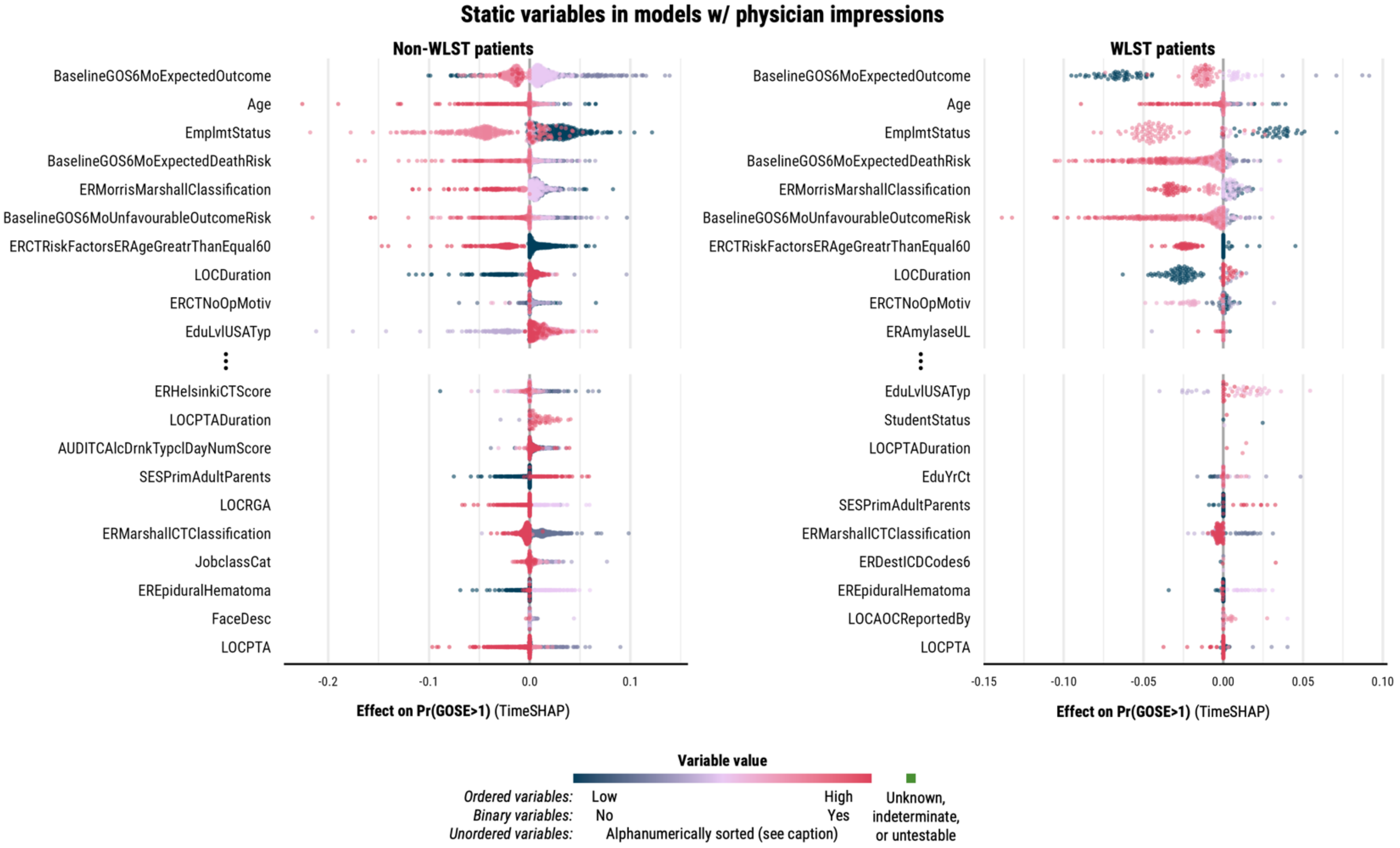

B

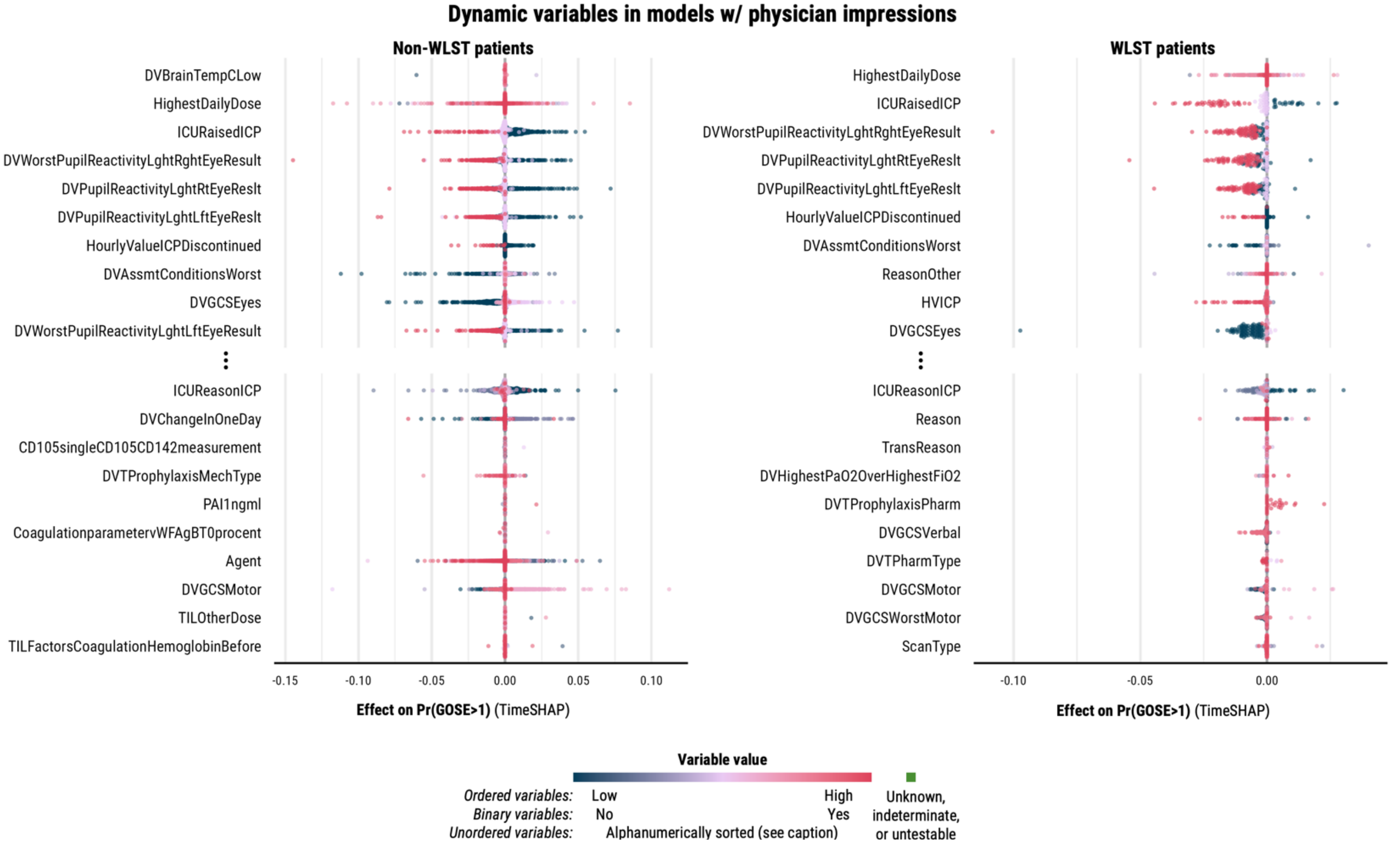

Supplementary Figure 10 (*continued*).

C

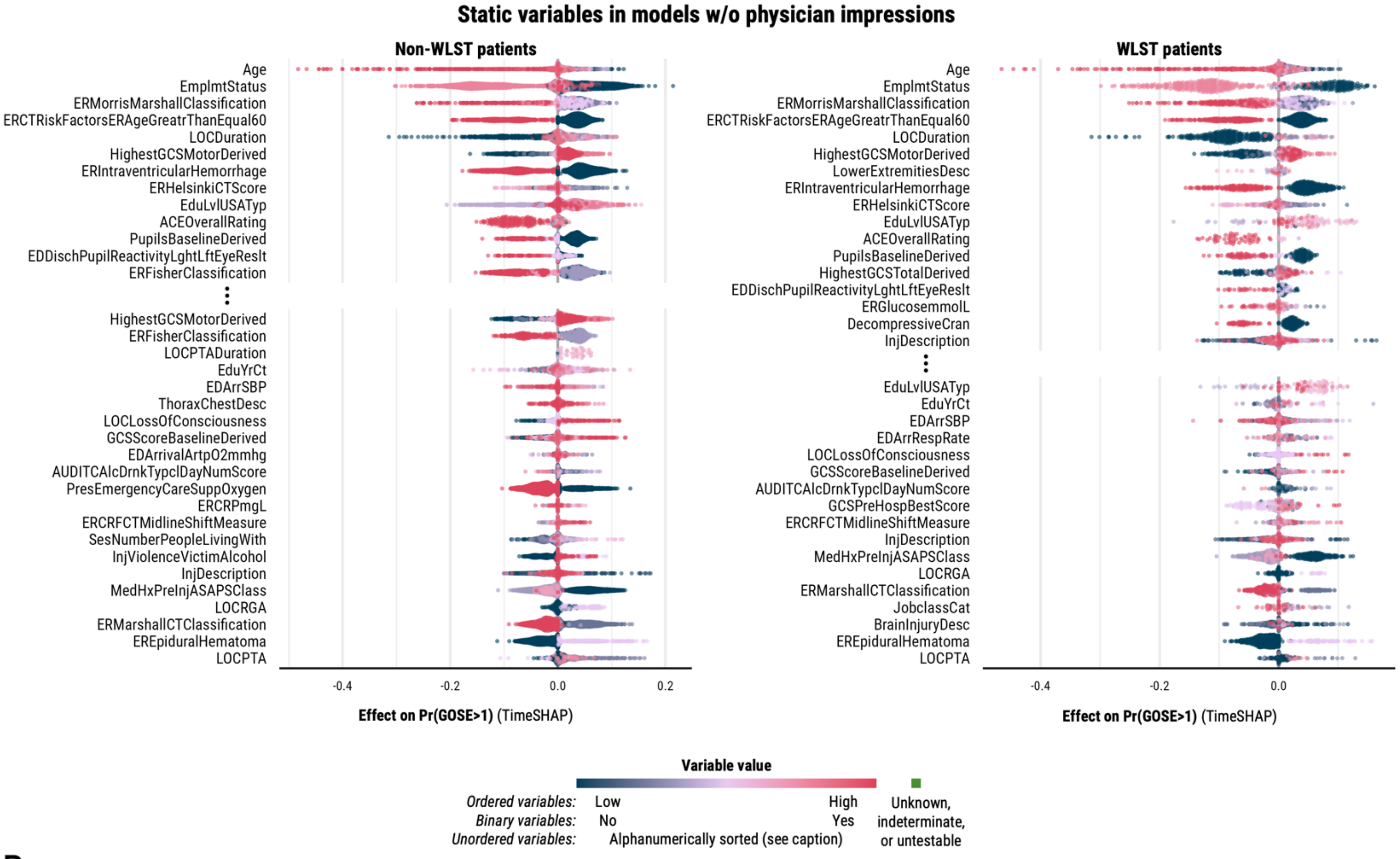

D

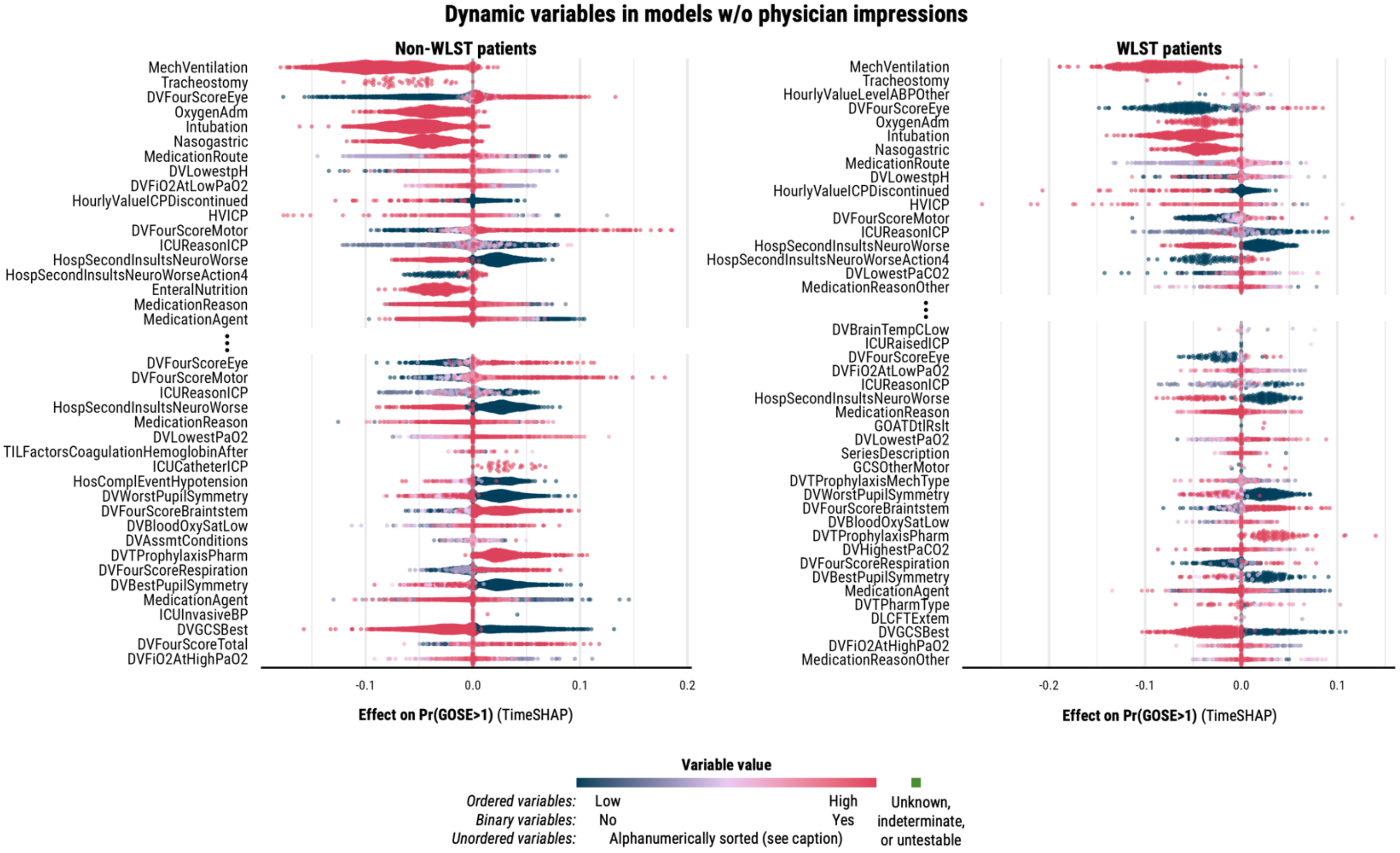

Supplementary Figure 10. TimeSHAP values stratified by WLST and inclusion of physician impressions. All abbreviated variable names are decoded in Supplementary Note 1. TimeSHAP values are interpreted as contributions of variables towards the difference in a patient's probability of survival (i.e., Pr(GOSE>1)) from that of the average patient (Supplementary Figure 13). The variables were selected – for each category of variables – by first identifying the ten variables with non-missing value tokens with the most negative median TimeSHAP values across the population (above the ellipses) and then, among the remaining variables, selecting the ten with non-missing value tokens with the most positive median TimeSHAP values (below the ellipses). Each point represents the mean TimeSHAP value for a token across an individual patient's high-magnitude transitions. The colour of the point represents the relative ordered value of a token within a variable, and for unordered variables (e.g., employment status before injury), tokens were sorted alphanumerically (the sort index per possible unordered variable token is provided in Supplementary Note 1). Green points represent variable tokens that are not missing but explicitly encode an unknown value.

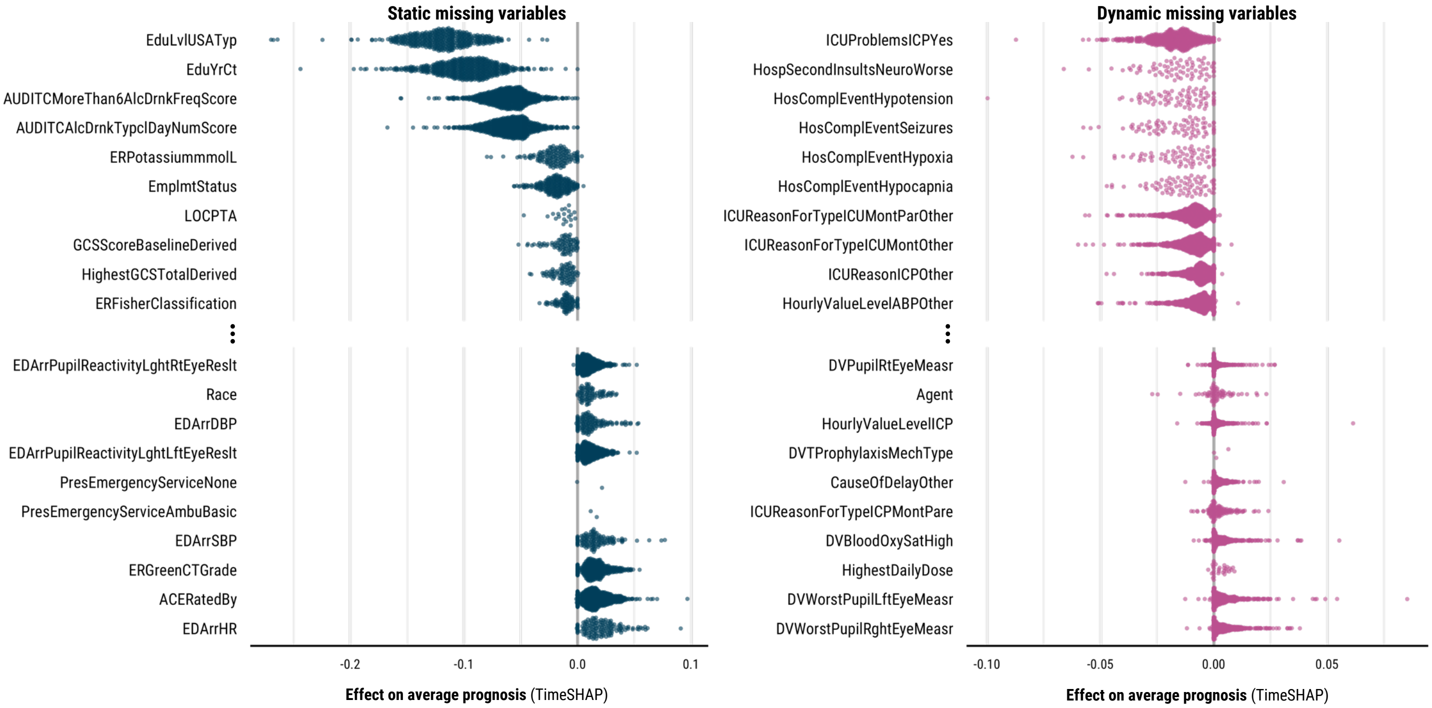

Supplementary Figure 11. Population-level TimeSHAP values for missing value tokens. All abbreviated variable names are decoded in Supplementary Note 1. TimeSHAP values are interpreted as contributions of a variable's missingness towards the difference in a patient's expected six-month functional outcome output from that of the average patient (Supplementary Figure 13). The variables were selected – for each category of variables – by first identifying the ten variables with missing value tokens with the most negative median TimeSHAP values across the population (above the ellipses) and then, among the remaining variables, selecting the ten with missing value tokens with the most positive median TimeSHAP values (below the ellipses). Each point represents the mean TimeSHAP value for a missing value token across an individual patient's high-magnitude transitions.

Supplementary Figure 12. Example of individual ICU disease course for each level of six-month functional outcome. Legend provided at end of figure (pp 19).

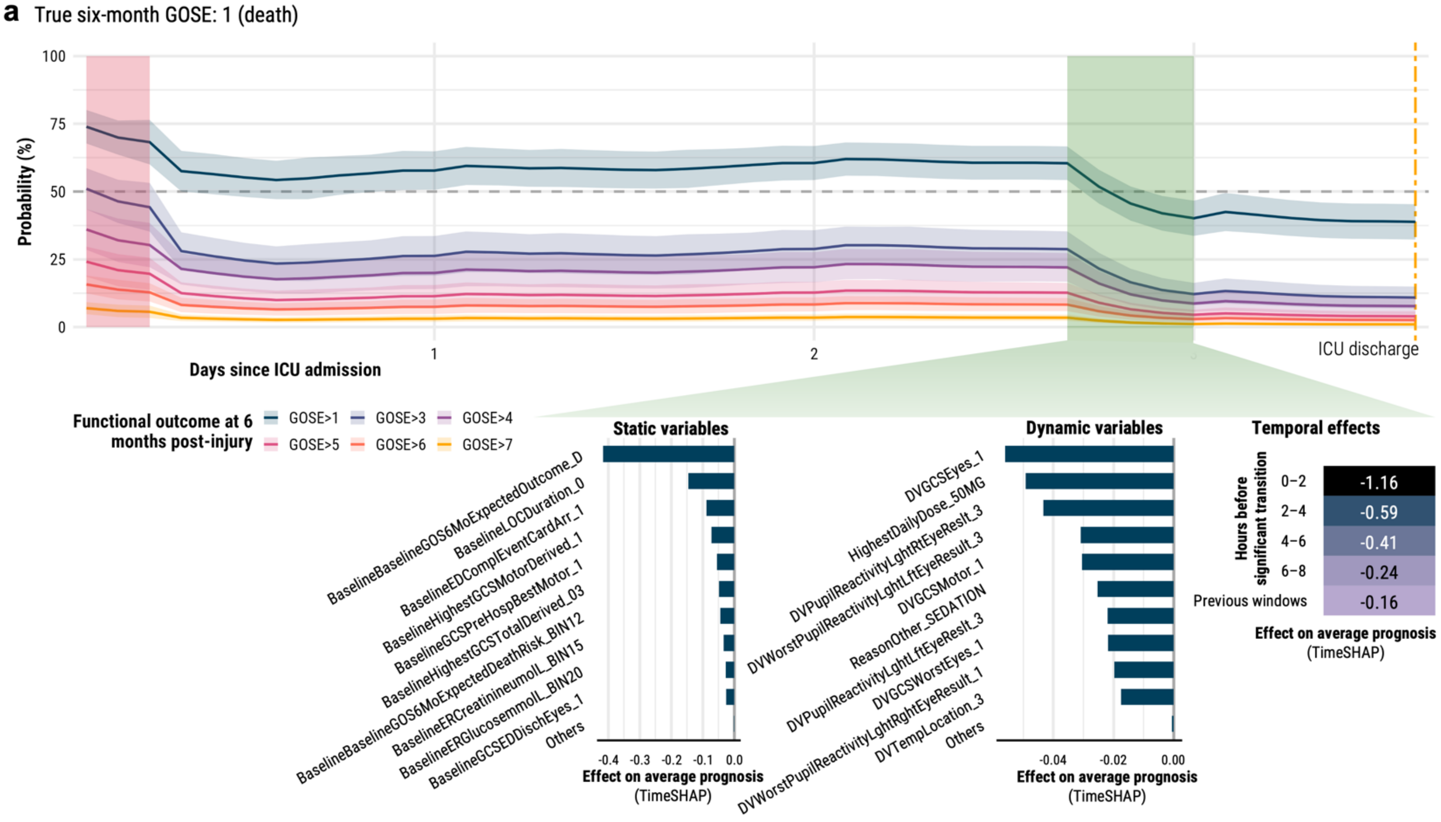

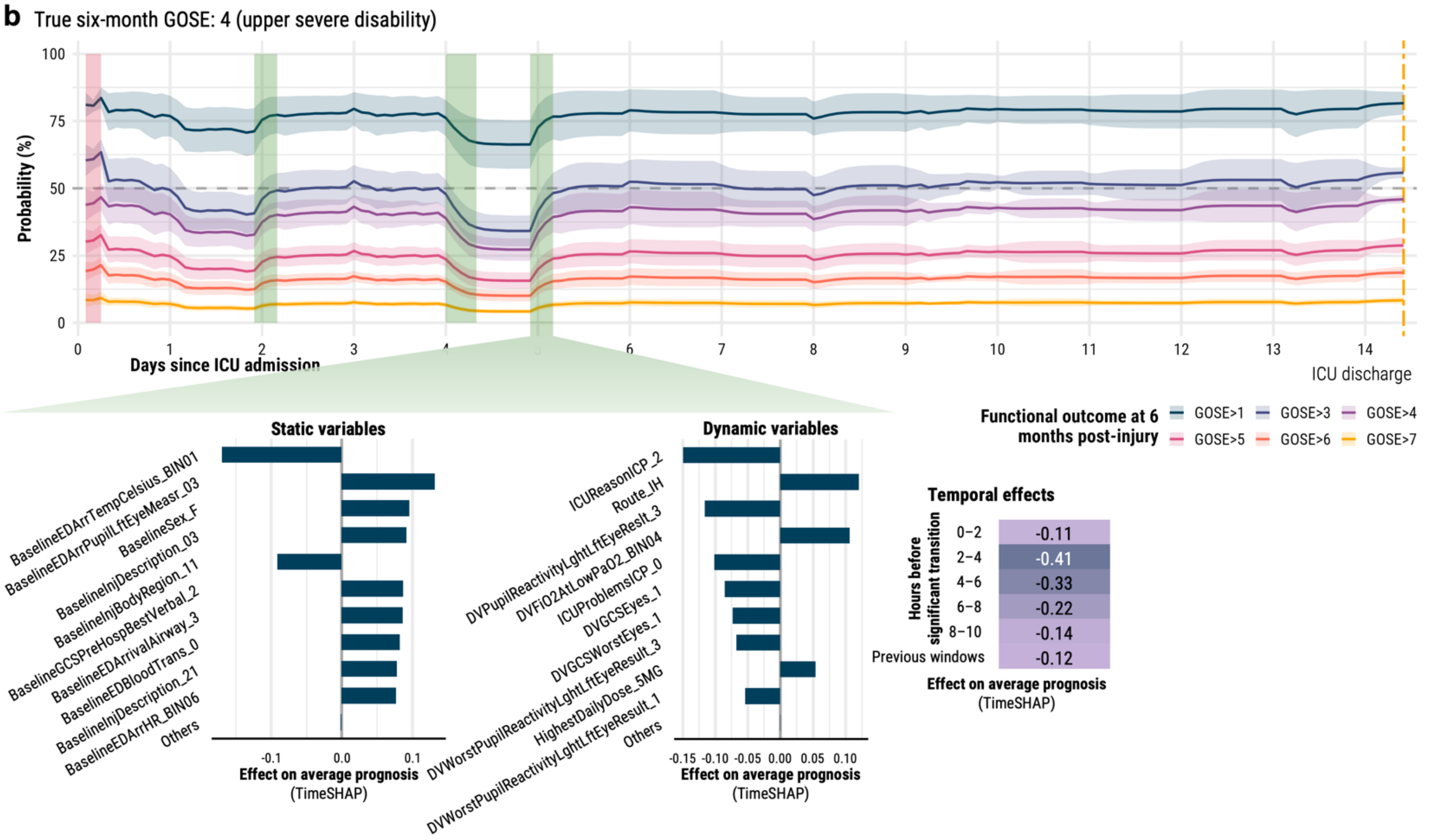

Supplementary Figure 12 (*continued*).

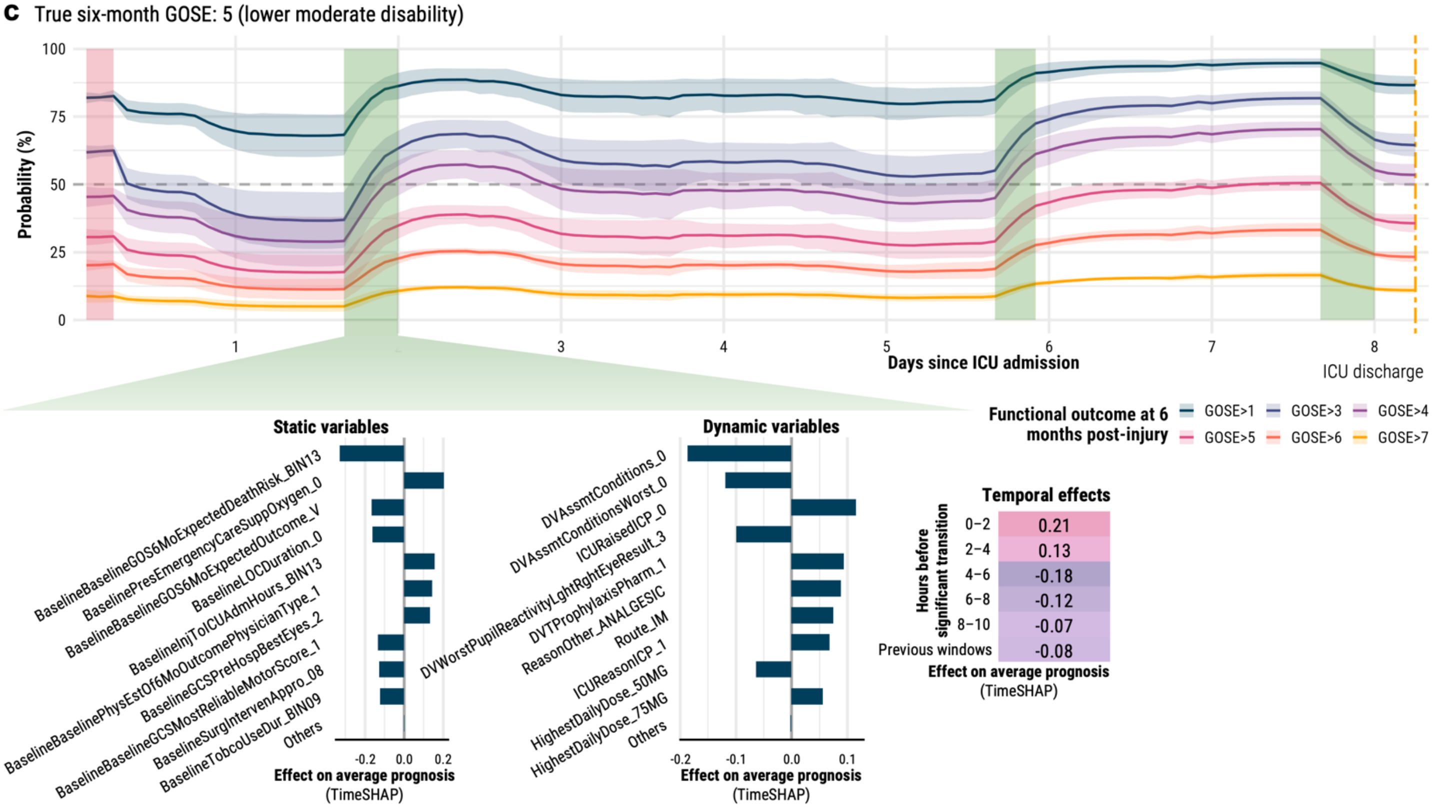

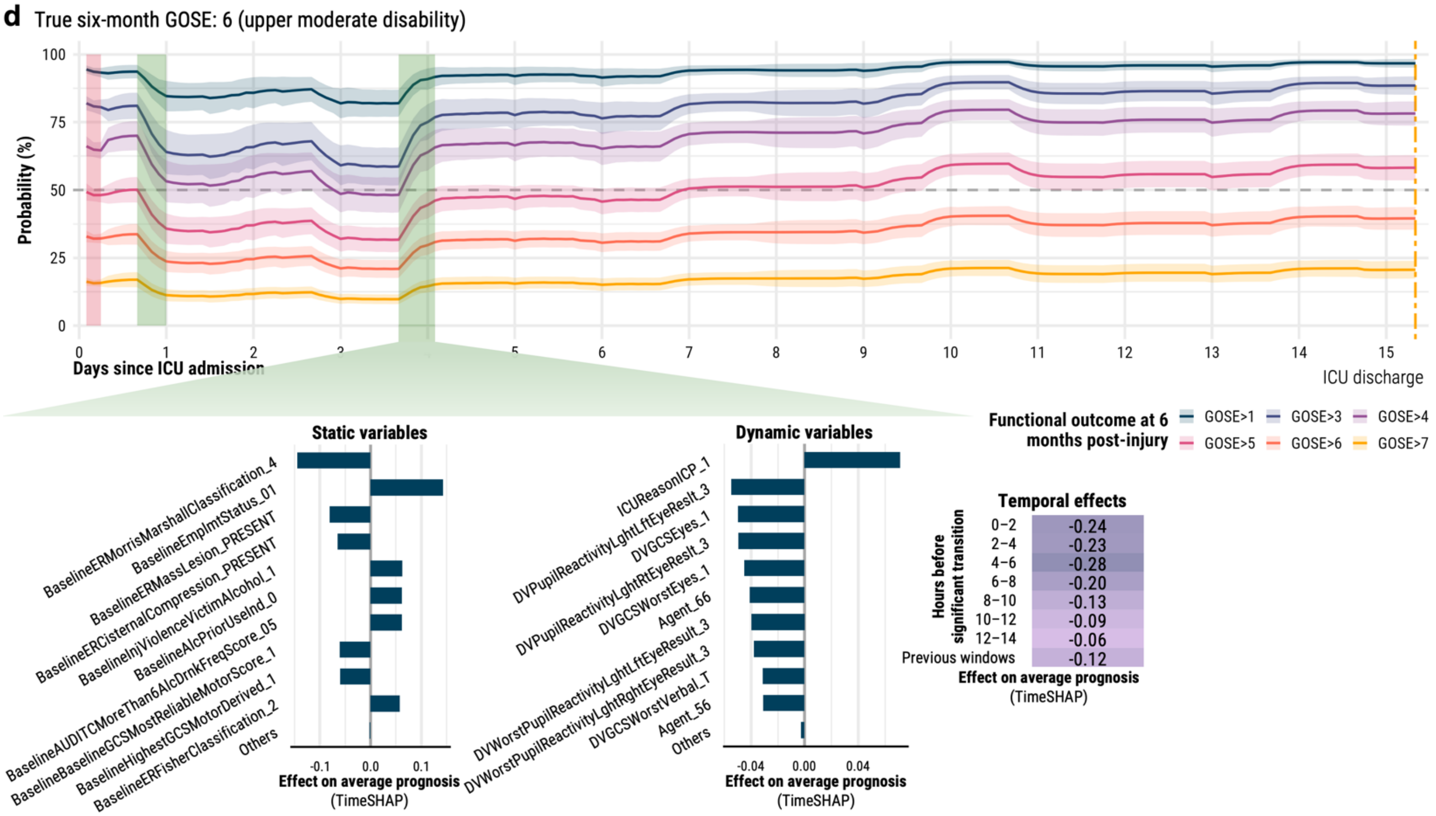

Supplementary Figure 12 (*continued*).

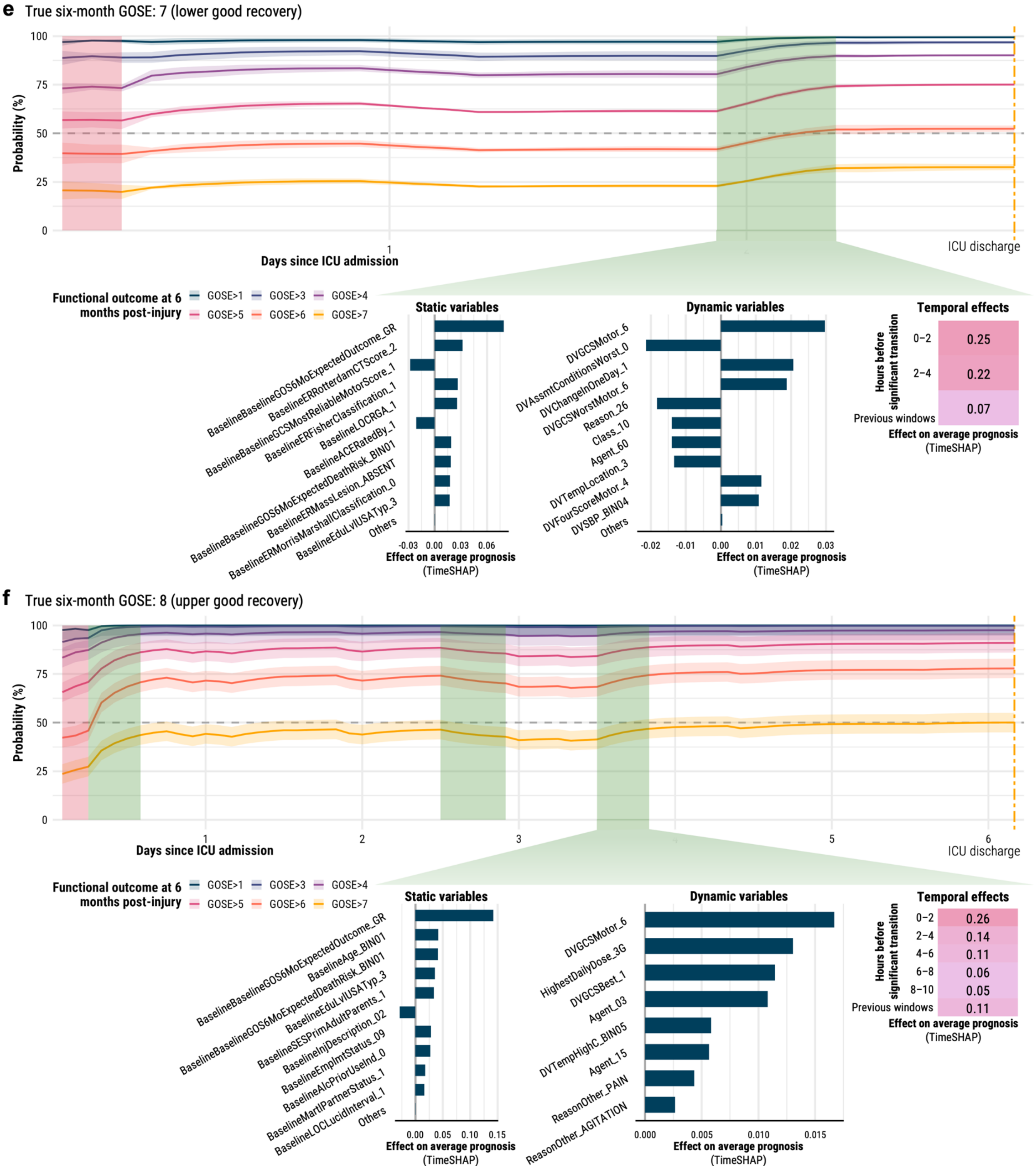

Supplementary Figure 12. Example of individual ICU disease course for each level of six-month functional outcome. All abbreviated variable names are decoded in Supplementary Note 1. Shaded red regions represent the pre-calibration range of model outputs (Figure 1) while shaded green regions represent high-magnitude transitions (as defined in the Methods). TimeSHAP values are interpreted as contributions of variables towards the difference in a patient's expected six-month functional outcome output from that of the average patient (Supplementary Figure 13). (**a**) A c. 40-year-old male, admitted to the ICU after a severe TBI (GCS 3), who died within six months of TBI (GOSE 1). (**b**) A c. 20-year-old female, admitted to the ICU after a severe TBI (GCS 7), who became severely disabled with partial functional dependency by six months post-injury (GOSE 4). (**c**) A c. 60-year-old male, admitted to the ICU after a

severe TBI (GCS 5), who became functionally independent but unable to participate in one or more life roles by six months post-injury (GOSE 5). (**d**) A c. 40-year-old male, admitted to the ICU after a severe TBI (GCS 3), who became functionally independent and able to participate in a limited capacity in life roles by six months post-injury (GOSE 6). (**e**) A c. 30-year-old female, admitted to the ICU after a moderate TBI (GCS 11), who returned to normal life with some neurological symptoms by six months post-injury (GOSE 7). (**f**) A c. 20-year-old male, admitted to the ICU after a mild TBI (GCS 15), who fully returned to normal life by six months post-injury (GOSE 8).

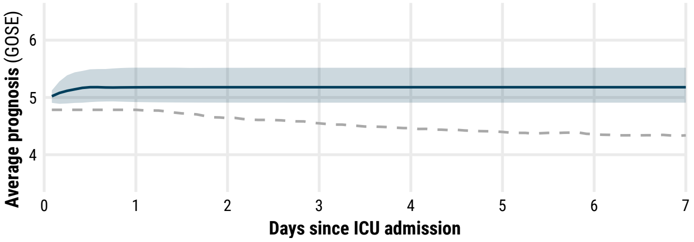

Supplementary Figure 13. Expected six-month functional outcome output for the average patient token set. The expected six-month functional outcome is defined as the GOSE corresponding to $\widehat{\mathbb{E}}[i]$, as defined in the Supplementary Methods. The average patient token set was defined to be one with tokens that are in 50+% of training set time windows, and the corresponding model output is the baseline for TimeSHAP calculation, against which variable contributions are calculated. The curve represents the median output across the 100 training sets of the repeated cross-validation partitions, and the shaded region represents the interquartile range. The dashed grey line represents the true average six-month functional outcome for the population over time, as patients left the ICU.

SUPPLEMENTARY TABLES

Supplementary Table 1. IMPACT extended model variables stratified by six-month functional outcome.

IMPACT extended model variables	Overall (n = 1550)	Glasgow Outcome Scale – Extended (GOSE) at 6 months post-injury							p-value
		**1** (n = 318)	**2 or 3** (n = 262)	**4** (n = 120)	**5** (n = 227)	**6** (n = 200)	**7** (n = 206)	**8** (n = 217)	
Age [years]	51 (31–66)	66 (50–76)	55 (36–68)	48 (29–61)	44 (31–56)	41 (27–53)	48 (31–65)	41 (24–61)	<0.0001
GCS motor score (n = 1509)	5 (1–6)	2 (1–5)	3 (1–5)	5 (1–6)	5 (1–6)	5 (2–6)	5 (3–6)	6 (5–6)	<0.0001
(1) No response	484 (32.1%)	152 (50.0%)	104 (40.6%)	35 (29.9%)	63 (28.5%)	46 (23.6%)	47 (23.0%)	37 (17.5%)	
(2) Abnormal extension	54 (3.6%)	17 (5.6%)	20 (7.8%)	4 (3.4%)	6 (2.7%)	3 (1.5%)	2 (1.0%)	2 (0.9%)	
(3) Abnormal flexion	63 (4.2%)	14 (4.6%)	12 (4.7%)	8 (6.8%)	11 (5.0%)	8 (4.1%)	4 (2.0%)	6 (2.8%)	
(4) Withdrawal from stimulus	114 (7.6%)	27 (8.9%)	23 (9.0%)	8 (6.8%)	20 (9.0%)	21 (10.8%)	8 (3.9%)	7 (3.3%)	
(5) Movement localised to stimulus	305 (20.2%)	52 (17.1%)	47 (18.4%)	24 (20.5%)	50 (22.6%)	46 (23.6%)	44 (21.6%)	42 (19.8%)	
(6) Obeys commands	489 (32.4%)	42 (13.8%)	50 (19.5%)	38 (32.5%)	71 (32.1%)	71 (36.4%)	99 (48.5%)	118 (55.7%)	
Unreactive pupils (n = 1465)									<0.0001
One	111 (7.6%)	31 (10.5%)	31 (12.3%)	7 (6.3%)	20 (9.3%)	5 (2.6%)	8 (4.1%)	9 (4.4%)	
Two	168 (11.5%)	84 (28.5%)	33 (13.0%)	8 (7.2%)	14 (6.5%)	8 (4.2%)	16 (8.2%)	5 (2.4%)	
Hypoxia	207 (13.4%)	60 (18.9%)	33 (12.6%)	14 (11.7%)	35 (15.4%)	33 (16.5%)	16 (7.8%)	16 (7.4%)	0.37
Hypotension	210 (13.5%)	56 (17.6%)	51 (19.5%)	21 (17.5%)	32 (14.1%)	22 (11.0%)	15 (7.3%)	13 (6.0%)	0.0038
Marshall CT score (n = 1255)	VI (II–VI)	III (II–VI)	II (II–VI)	II (II–VI)	II (II–II)	II (II–III)	II (II–II)	VI (II–VI)	0.043
No visible pathology (I)	118 (9.4%)	8 (3.3%)	11 (5.3%)	5 (5.2%)	17 (8.7%)	25 (15.2%)	24 (13.6%)	28 (16.5%)	
Diffuse injury II	592 (47.2%)	56 (22.8%)	84 (40.6%)	54 (56.2%)	92 (47.2%)	100 (60.6%)	103 (58.5%)	103 (60.6%)	
Diffuse injury III	108 (8.6%)	42 (17.1%)	17 (8.2%)	10 (10.4%)	14 (7.2%)	9 (5.5%)	6 (3.4%)	10 (5.9%)	
Diffuse injury IV	16 (1.3%)	7 (2.8%)	1 (0.5%)	1 (1.0%)	4 (2.1%)	1 (0.6%)	1 (0.6%)	1 (0.6%)	
Mass lesion (V & VI)	421 (33.5%)	133 (54.0%)	94 (45.4%)	26 (27.1%)	68 (34.9%)	30 (18.2%)	42 (23.9%)	28 (16.5%)	
Traumatic subarachnoid haemorrhage (n = 1254)	957 (76.3%)	221 (90.2%)	176 (84.2%)	73 (76.0%)	150 (76.9%)	106 (63.9%)	125 (71.4%)	106 (63.1%)	0.16
Extradural haematoma (n = 1257)	244 (19.4%)	31 (12.7%)	32 (15.3%)	21 (21.9%)	46 (23.6%)	32 (19.3%)	42 (23.9%)	40 (23.5%)	0.016
Glucose [mmol/L] (n = 1062)	7.7 (6.6–9.4)	8.8 (7.3–11)	8.0 (6.5–9.8)	7.6 (6.5–9.3)	7.8 (6.6–9.6)	7.7 (6.5–8.7)	7.3 (6.3–8.5)	7.1 (6.3–8.1)	0.013
Haemoglobin [g/dL] (n = 1140)	13 (12–14)	13 (11–14)	13 (11–14)	14 (12–14)	13 (12–14)	14 (12–15)	13 (12–15)	14 (13–15)	0.038

Data are median (IQR) for continuous variables and n (% of column group) for categorical variables. Units of variables are provided in square brackets. If a characteristic had missing values for some patients in the population, the non-missing sample size was provided in parentheses – e.g., Marshall CT score (n = 1255). p-values are determined from proportional odds logistic regression (POLR) analysis trained on all IMPACT variables concurrently[A1] and are combined across 100 missing value imputations via z-transformation[A2]. For categorical variables with $k > 2$ categories (e.g., GCSm), p-values were calculated with a likelihood ratio test (with k-1 degrees of freedom) on POLR.

Supplementary Table 2. Calculated cut-offs to define high-magnitude transitions.

Threshold	Cutoffs (Δ%)	
	**Negative transitions**, i.e., worsening (≤)	**Positive transitions**, i.e., improvement (≥)
GOSE>1	-6.258903	4.017577
GOSE>3	-6.168803	4.727354
GOSE>4	-5.831151	4.435974
GOSE>5	-5.253576	3.712104
GOSE>6	-4.371744	2.740238
GOSE>7	-3.044348	1.758345

For negative transitions, data represent the 1st percentile of negative differences in consecutive time-window probabilities (in percentage) for the given GOSE threshold across the population. High-magnitude negative transitions were defined as those with probability differences less than or equal to the cut-off value for the given GOSE threshold. For positive transitions, data represent the 99th percentile of positive differences in consecutive time-window probabilities (in percentage) for the given GOSE threshold across the population. High-magnitude positive transitions were defined as those with probability differences greater than or equal to the cut-off value for the given GOSE threshold.

Supplementary Table 3. CENTER-TBI study sites, ethical committees, approval numbers, and approval dates.

Country	City	Insitute	Ethical Commitee	Number Approval	Date Approval
**Austria**	Vienna	Medizinische Universität Wien Universitätsklinik für Unfallchirurgie	Ethikkommission der Medizinischen Universität Wien	1646/2014	3/05/2016
**Austria**	Innsbruck	Medizinische Universität Innsbruck Universitätsklinik für Neurologie	Ethikkommission der Medizinischen Universität Innsbruck	AN2014-0336 343/4.22	1/12/2014
**Belgium**	Antwerp	Antwerp University Hospital	Centraal Ethisch Comité - Ethisch Comité Universitair Ziekenhuis Antwerpen en de Universiteit Antwerpen	B300201422714	17/11/2014
**Belgium**	Liège	CHR Citadelle	- Centraal Ethisch Comité - Ethisch Comité Universitair Ziekenhuis Antwerpen en de Universiteit Antwerpen<br>- Comité d'Ethique 412	- B300201422714<br>- 1427	-17/11/2014<br>- 25/11/2014
**Belgium**	Liège	CHU	- Centraal Ethisch Comité - Ethisch Comité Universitair Ziekenhuis Antwerpen en de Universiteit Antwerpen<br>- Comité d'Ethique hospitalo-facultaire niversitaire de Liège (707)	- B300201422714<br>- B707201422102 / 2014-244	- 17/11/2014<br>- 07/10/2014
**Belgium**	Leuven	UZ Leuven	- Centraal Ethisch Comité - Ethisch Comité Universitair Ziekenhuis Antwerpen en de Universiteit Antwerpen<br>- Comissie Medische Ethiek UZ KU Leuven / Onderzoek	- B300201422714<br>- B322201523981 / S57019 (ML11365)	- 06/13/2015<br>- 07/10/2014
**Denmark**	Odense	Odense Universiteitshospital - Neurokirurgisk afdeling	De Videnskabsetiske Komitéer for Region Syddanmark	S-20140215	16/03/2015
**Denmark**	Copenhagen	Region Hovedstaden Rigshopitalet	De Videnskabsetiske Komitéer for Region Syddanmark	S-20140215	16/03/2015
**Finland**	Turku	Turku University Hospital	Varsinais suomen sairaanhoitopiirin kuntayhtyma - Eettinen Toimikunta	95/1801/2014	24/10/2014
**Finland**	Helsinki	Helsinki University Central Hospital	Varsinais suomen sairaanhoitopiirin kuntayhtyma - Eettinen Toimikunta	95/1801/2014	24/10/2014
**France**	Paris	APHP	Agence Nationale de Sécurité du Médicament et des Produits de Santé ANSM	141421B-31	19/12/2014
**France**	Besançon	CHRU de Besançon	Agence Nationale de Sécurité du Médicament et des Produits de Santé ANSM	141421B-31	19/12/2014
**France**	Lille	Lille University Hospital	Agence Nationale de Sécurité du Médicament et des Produits de Santé ANSM	141421B-31	19/12/2014
**France**	Grenoble	University Hospital of Grenoble	Agence Nationale de Sécurité du Médicament et des Produits de Santé ANSM	141421B-31	19/12/2014
**France**	Nancy	University Hospital Nancy	Agence Nationale de Sécurité du Médicament et des Produits de Santé ANSM	141421B-31	19/12/2014
**France**	Poitiers	CHU Poitiers	Agence Nationale de Sécurité du Médicament et des Produits de Santé ANSM	141421B-31	19/12/2014
**Germany**	Heidelberg	Universitätsklinikum Heidelberg Neurochirurgische Kliniki	Ethikkommission Medizinsche Fakultät Heidelberg	S-435/2014	27/05/2015
**Germany**	Berlin	Charité | Campus Virchow Klinikum	Ethikkommission an der Medizinsche Fakultät Der rheinsch-Westfälischen Technischen Hocgschule Aachen	1098/15	26/02/2016
**Germany**	Aachen	Uniklinik RWTH Aachen	Ethikkommission an der Medizinsche Fakultät Der rheinsch-Westfälischen Technischen Hocgschule Aachen	EK 174/15	2/07/2015
**Germany**	Ludwigsburg	Klinikum Ludwigsburg	Ethikkommission Medizinsche Fakultät Heidelberg	S-435/2014	29/01/2016
**Hungary**	Pecs	Pecsi Tudomanyegyetem Klinikai Kozpont	- ETT TUKEB Egészségügyi Tudományos Tanács<br>- Pécsi Tudományegyetem	- 42558-3/2014/EKU<br>- 5421	- 2/10/2014<br>- 28/03/2015
**Hungary**	Szeged	zegedi tudományegyetem orvostudományi kar Szent Györgyi Albert Klinikai Központ	-ETT TUKEB Egészségügyi Tudományos Tanács<br>- Szegedi Tudományegyetem	- 42558-3/2014/EKU<br>- 3803	- 2/10/2014<br>- 23/05/2016
**Israel**	Haifa	Rambam Medical Center	Helsinki Committee, Rambam Health Care Campus	RMB 373-14	22/07/2015
**Israel**	Jeruzalem	Hadassah-hebrew University Medical Center	Hadassah Medical Organization IRB	0590-16 HMO	6/12/2016
**Italy**	Milan	Fondazione IRCCS Ca' Granda Ospedale Maggiore Policlinico	Fondazione IRCCS Ca' Granda Ospedale Maggiore Policlinico - Direzione Scientifica Comitato Etico	542/2014	20/10/2014
**Italy**	Milan	Ospedale San Raffaele	Comitato Etico - Ospedale San Raffaele	217/2014	10/04/2015
**Italy**	Torino	AOU Città della Salute e della Scienza di Torino	Comitato Etico Interaziendale A.O.U. Città della Salute e della Scienza di Torino - A.O. Ordine Mauriziano - A.S.L.	0015269	15/02/2016
**Italy**	Cesena	Bufalini Hospital	Comitato Etico IRST IRCCS AVR	1675/2015 I.5/207	18/03/2015

**Italy**	Padova	Azienda Ospedaliera Università di Padova	Comitato Etico - Ospedale San Raffaele	217/2014	10/04/2015
**Italy**	Monza	San Gerardo Hospital/ASST	Comitato Etico Della Provincia Monza Brianza	1978/2014	22/12/2014
**Italy**	Novara	Maggiore Della Carità Hospital	Comitato Etico Interaziendale A.O.U. 'Maggiore della Carità'	CE 46/15	3/07/2015
**Italy**	Milan	Niguarda Hospital	Comitato Etico - Ospedale Niguarda Ca' Granda	636-122015	23/12/2015
**Latvia**	Riga	Pauls Stradins Clinical University Hospital	Ethics Commiitee for Clinical Research at Pauls Stradins Clinical University Hospital Development Society	171215-1E	17/12/2015
**Latvia**	Riga	Riga Eastern Clinical University Hospital	Ethics Commiitee for Clinical Research at Pauls Stradins Clinical University Hospital Development Society	171215-1E	17/12/2015
**Latvia**	Rezekne	Rezekne Hospital	Ethics Commiitee for Clinical Research at Pauls Stradins Clinical University Hospital Development Society	171215-1E	17/12/2015
**Lithuania**	Vilnius	Vilniaus Universiteto Ligonine	VILNIAUS REGIONINIS BIOMEDICININIŲ TYRIMŲ ETIKOS KOMITETAS	158200-15-801-323	6/10/2015
**Lithuania**	Kaunas	LSMUL Kauno klinikos Skubio pagalbos skyrius	KAUNO REGIONINIS BIOMEDICININIŲ TYRIMŲ ETIKOS KOMITETAS	BE-2-6	6/01/2015
**Netherlands**	Leiden	Het Leids Universitair Medisch Centrum te Leiden	Leids Universitair Centrum - Commissie Medische Ethiek	P14.222/NV/nv	3/12/2014
**Netherlands**	Rotterdam	Erasmus MC	Leids Universitair Centrum - Commissie Medische Ethiek	P14.222/NV/nv	3/12/2014
**Netherlands**	The Hague	Medisch Centrum Haaglanden	Leids Universitair Centrum - Commissie Medische Ethiek	P14.222/NV/nv	3/12/2014
**Netherlands**	The Hague	Het Haga Hospital	Leids Universitair Centrum - Commissie Medische Ethiek	P14.222/NV/nv	25/11/2015
**Netherlands**	Nijmegen	Radboud UMC	Leids Universitair Centrum - Commissie Medische Ethiek	P14.222/NV/nv	2/07/2015
**Netherlands**	Tilburg	St. Elisabeth Ziekenhuis	Leids Universitair Centrum - Commissie Medische Ethiek	P14.222/NV/nv	2/07/2015
**Netherlands**	Groningen	UMC Groningen	Leids Universitair Centrum - Commissie Medische Ethiek	P14.222/NV/nv	3/12/2014
**Norway**	Tromso	Universitetssykehuset Nord-Norge	Regional komité for medisinsk og helsefaglig forskningsetikk REK midt-Norge (REK midt)	2014/1454	23/03/2015
**Norway**	Trondheim	St.Olavs Hospital	Regional komité for medisinsk og helsefaglig forskningsetikk REK midt-Norge (REK midt)	2014/1454	23/03/2015
**Norway**	Oslo	Oslo Universitetssykehus	Regional komité for medisinsk og helsefaglig forskningsetikk REK midt-Norge (REK midt)	2014/1454	23/03/2015
**Romania**	Timisoara	Clinica de Neurochirurgie Universitatea de Medicina si Farmacie "Victor Babes" Timisoara	Comitetului de Etica a Spitalului Clinic Judeteam de Urgenta Timisoara	73	16/10/2014
**Serbia**	Novi Sad	Klinidkog centra Vojvodine	Etidkog odbora Klinidkog centra Vojvodine	00-08/332	11/07/2014
**Spain**	Madrid	Hospital Universitario 12 de Octubre	Comité Etico de Investigacion Clinica del Hospital Universitario 12 de Octubre	14/262	3/11/2014
**Spain**	Barcelona	Vall d'Hebron University Hospital	Comité ético de investigación clínica y comisión de proyectos de investigación del hospital universitari Vall d'Hebron	ID-RTF080	13/11/2014
**Spain**	Bilbao	Clínico Universitario de Cruces	Comité Etico de Investigacion Clinica de Euskadi	PI2014158	24/02/2015
**Spain**	Valencia	Clínico Universitario de Valencia	Comité Etico de Investigacion Clinica del Clínico Universitario de Valencia	F-CE-GEva-15	23/04/2015
**Sweden**	Stockholm	Karolinksa University Hospital	EPN (Regionala Etikprövningsnämnden i Stockholm)	2014/1473-31/4	24/09/2014
**Sweden**	Umea	Umea University Hosptital	EPN (Regionala Etikprövningsnämnden i Stockholm)	2014/1473-31/4	24/09/2014
**Switzerland**	Lausanne	Centre hospitalier universitaire Vaudois	La Commission cantonale (VD) d'éthique de la recherche sur l'être humain (CER-VD)	473/11	9/12/2014
**UK**	Birmingham	Queen Elizabeth Hospital	- NHS HRA<br>- UHB Research Governance Office - Queen Elizabeth Hospital	- 14/SC/1370<br>- RRK5224	- 22/12/2014<br>- 24/02/2016
**UK**	Cambridge	Cambridge University Hospital NHS Foundation Trust	- NHS HRA<br>- Research and Development Department - Cambridge University Hospital NHS Foundation Trust	- 14/SC/1370<br>- AO93184	- 22/12/2014<br>- 30/01/2015
**UK**	Southampton	University Hospitals Southhampton NHS Trust	- NHS HRA<br>- Research Governance Office - University Hospitals Southhampton NHS Trust	- 14/SC/1370<br>- RHM CRI0294	- 22/12/2014<br>- 13/03/2015
**UK**	Sheffield	Sheffield Teaching Hospitals NHS Foundation Trust	- NHS HRA<br>- Research and Development Department - Sheffield Teaching Hospitals NHS Foundation Trust	- 14/SC/1370<br>- STH18187	- 22/12/2014<br>- 25/03/2015

**UK**	London	Kings college London	- NHS HRA<br>- Research & Innovation Office - Kings college London NHS Foundation Trust	- 14/SC/1370<br>- KCH15-204	-<br>22/12/2014<br>-<br>30/12/2015
**UK**	Salford	Salford Royal Hospital	- NHS HRA<br>- Research and Development Department -Salford Royal Hospital NHS Foundation Trust	- 14/SC/1370<br>- 2015/025ET	-<br>22/12/2014<br>-<br>27/07/2015
**UK**	Liverpool	The Walton centre NHS Foundation Trust	- NHS HRA<br>- Research & Innovation Office - The Walton centre NHS Foundation Trust	- 14/SC/1370<br>- RG154-15	-<br>22/12/2014<br>-<br>11/05/2015
**UK**	Bristol	Southmead Hospital Bristol	- NHS HRA<br>- Research & Innovation - North Bristol NHS Trust	- 14/SC/1370<br>- 3427	-<br>22/12/2014<br>-<br>23/12/2014
**UK/Scotland**	Edinburgh	Lothian Health Board	- NHS Scotland<br>- Research and Development Department - University Hospitals Division NHS Lothian	- 14/SS/1086<br>- 2015/0171	-<br>23/12/2014<br>-<br>19/06/2015

SUPPLEMENTARY NOTES

NOTE: Static variables are those with values fixed at ICU admission (e.g., helmet on during accident?). Intervention variable directly represent a treatment or management decision performed during a patient's ICU stay (i.e., administration of hypertonic saline). Since an intervention variable must take place during a patient's ICU stay, a variable cannot be both a static and an intervention variable. However, a variable can be both not static (i.e., dynamic) and not an intervention (e.g., a result from an ICU lab test or imaging report).

Supplementary Note 1. Variables by category.

Demographics and socioeconomic status

Name	Static	Intervention	Format	Description	Possible Values
Age	TRUE	FALSE	integer	Age, recorded in years.	Not Applicable
EduLvlEUROFather	TRUE	FALSE	integer	Father Highest level of education completed. Only if patient age <18	0=None, not currently in school;1=Currently in diploma or degree-oriented program;2=Primary school;3=Secondary school / High school;4=Post-high school training (e.g. trade/technical certificate);5=College / University (diploma or degree);88=Unknown
EduLvlEUROMother	TRUE	FALSE	integer	Mother Highest level of education completed. Only if patient age <18	0=None, not currently in school;1=Currently in diploma or degree-oriented program;2=Primary school;3=Secondary school / High school;4=Post-high school training (e.g. trade/technical certificate);5=College / University (diploma or degree);88=Unknown
EduLvlUSATyp	TRUE	FALSE	integer	Highest level of education of patient.	0=None, not currently in school;1=Currently in diploma or degree-oriented program;2=Primary school;3=Secondary school / High school;4=Post-high school training (e.g. trade/technical certificate);5=College / University (diploma or degree);88=Unknown
EduYrCt	TRUE	FALSE	integer	Number of years of education completed.	Not Applicable
EmplmtStatus	TRUE	FALSE	integer	Employment status before injury.	1=Working (35 hours or more per week);2=Working (20-34 hours per week);3=Working (less than 20 hours per week);4=In working force, but currently on sick leave;5=Special employment / sheltered employment;6=Looking for work, unemployed;7=Unable to work;8=Retired;9=Student / schoolgoing;10=Homemaker, keeping house;88=Unknown
JobclassCat	TRUE	FALSE	integer	Reflects job category. Only displayed if employment status (Subject.EmplmtStatus) is 1-4	0=None;1=Manager / Professional;2=Technician / Supervisor / Associate Professional;3=Clerk / Sales;4=Skilled manual worker;5=Manual worker;99=Other
JobclassCatOther	TRUE	FALSE	text	Specifies the "other" job category than predefined list. Only displayed if employment status (Subject.EmplmtStatus) is 1-4	Not Applicable
MartlPartnerStatus	TRUE	FALSE	integer	Marital status.	1=Never been married;2=Married;3=Living together/common law;4=Divorced;5=Separated;6=Widowed;88=Unknown;99=Other
Race	TRUE	FALSE	text	Race of Subject	Asian=Asian;Black=Black;NotAllowed=Not allowed; Unknown=Unknown;White=White
SesEduNoFather	TRUE	FALSE	integer	Father's number of years education completed. Only if patient age <18	Not Applicable
SesEduNoMother	TRUE	FALSE	integer	Mother's number of years of education completed. Only if patient age <18	Not Applicable
SesNumberPeopleLivingWith	TRUE	FALSE	integer	Living together and number of people living with provides an indication of "social support" - important to recovery and social re-integration.	Not Applicable
SESPrimAdultAlone	TRUE	FALSE	integer	Reflects living situation prior to the injury: --> Alone	0=No;1=Yes
SESPrimAdultChildren	TRUE	FALSE	integer	Reflects living situation prior to the injury: --> with Child/children	0=No;1=Yes
SESPrimAdultParents	TRUE	FALSE	integer	Reflects living situation prior to the injury: --> with parents	0=No;1=Yes
SESPrimAdultSiblings	TRUE	FALSE	integer	Reflects living situation prior to the injury: --> with siblings	0=No;1=Yes
SESPrimAdultSignOther	TRUE	FALSE	integer	Reflects living situation prior to the injury: --> with a significant other partner	0=No;1=Yes
SESPrimAdultSpousePartner	TRUE	FALSE	integer	Reflects living situation prior to the injury: --> with spouse	0=No;1=Yes
Sex	TRUE	FALSE	text	Sex of subject	F=Female;M=Male
SiteCode	TRUE	FALSE	text	Anonymized site code.	Not Applicable
StudentStatus	TRUE	FALSE	integer	Only displayed if employment status is student/school going	0=None;1=Full time, diploma / degree oriented;2=Part time, diploma / degree oriented;3=Other school;88=Unknown

Medical and behavioural history

Name	Static	Intervention	Format	Description	Possible Values
AlcPriorUseInd	TRUE	FALSE	integer	On presentation the behavioral history of the patient was recorded. This reflects his past use of alcoholic beverages (beer, wine, spirits).	0=No;1=Yes;88=Unknown
AlcUseDur	TRUE	FALSE	integer	On presentation the behavioral history of the patient was recorded. This reflects the number of years of alcohol use, if past use of alcoholic beverages (beer, wine, spirits) was 'yes'.	Not Applicable
AlcUseLstMoDaysDrankNum	TRUE	FALSE	integer	On presentation the behavioral history of the patient was recorded. This reflects his use in the past three months of alcoholic beverages (beer, wine, spirits) (>2/day)	0=No;1=Yes;88=Unknown
AnticoagAntiThrombinProtein	TRUE	FALSE	integer	Medical history. Use of anticoagulant or platelet aggregation inhibitor by the patient. This variable describes the use of antithrombin protein therapeutics (Atryn).	0=No;1=Yes
AnticoagCoumarin	TRUE	FALSE	integer	Medical history. Use of anticoagulant or platelet aggregation inhibitor by the patient. This variable describes the use of Coumarin derivative (Coumadin, Warfarin).	0=No;1=Yes
AnticoagDirectThrombinInhib	TRUE	FALSE	integer	Medical history. Use of anticoagulant or platelet aggregation inhibitor by the patient. This variable describes the use of direct thrombin inhibitors (eg. dabigatran, argatroban, melagatran).	0=No;1=Yes
AnticoagFactorXaInhib	TRUE	FALSE	integer	Medical history. Use of anticoagulant or platelet aggregation inhibitor by the patient. This variable describes the use of inhibitor of factor Xa (eg. rivaroxaban).	0=No;1=Yes
AnticoagHeparin	TRUE	FALSE	integer	Medical history. Use of anticoagulant or platelet aggregation inhibitor by the patient. This variable describes the use of heparin.	0=No;1=Yes
AnticoagLowMolHeparin	TRUE	FALSE	integer	Medical history. Use of anticoagulant or platelet aggregation inhibitor by the patient. This variable describes the use of low molecular weight heparin	0=No;1=Yes
AnticoagulantOther	TRUE	FALSE	integer	Medical history. Use of anticoagulant or platelet aggregation inhibitor by the patient. This variable describes the use of another type of anticoagulant or platelet aggregation inhibitor, not specified elsewhere.	0=No;1=Yes
AnticoagulantOtherTxt	TRUE	FALSE	text	Medical history. Use of anticoagulant or platelet aggregation inhibitor by the patient. This variable describes the use of another type of anticoagulant or platelet aggregation inhibitor, not specified elsewhere. Text field.	Not Applicable
AnticoagulantReasonCardiac	TRUE	FALSE	integer	Medical history. This variable is populated when the reason for using anticoagulants or platelet aggregation inhibitors by the patient is cardiac.	0=No;1=Yes
AnticoagulantReasonCardiacCABG	TRUE	FALSE	integer	Medical history. This variable is populated when the reason for using anticoagulants or platelet aggregation inhibitors by the patient is cardiac, specifically CABG.	0=No;1=Yes
AnticoagulantReasonCardiacFibrill	TRUE	FALSE	integer	Medical history. This variable is populated when the reason for using anticoagulants or platelet aggregation inhibitors by the patient is cardiac, specifically atrial fibrillation/flutter.	0=No;1=Yes
AnticoagulantReasonCardiacStent	TRUE	FALSE	integer	Medical history. This variable is populated when the reason for using anticoagulants or platelet aggregation inhibitors by the patient is cardiac, specifically a cardiac stent.	0=No;1=Yes
AnticoagulantReasonCardiacValve	TRUE	FALSE	integer	Medical history. This variable is populated when the reason for using anticoagulants or platelet aggregation inhibitors by the patient is cardiac, specifically a valve prosthesis.	0=No;1=Yes
AnticoagulantReasonCardiovas	TRUE	FALSE	integer	Medical history. This variable is populated when the reason for using anticoagulants or platelet aggregation inhibitors by the patient is cardiovascular.	0=N0;1=Yes

Name	Static	Intervention	Format	Description	Possible Values
AnticoagulantReasonCardiovasCarotidStent	TRUE	FALSE	integer	Medical history. This variable is populated when the reason for using anticoagulants or platelet aggregation inhibitors by the patient is cardiovascular, specifically a carotid or cerebral stent.	0=No;1=Yes
AnticoagulantReasonCardiovasLimbIsch	TRUE	FALSE	integer	Medical history. This variable is populated when the reason for using anticoagulants or platelet aggregation inhibitors by the patient is cardiovascular, specifically limb ischaemia.	0=No;1=Yes
AnticoagulantReasonCardiovasOtherStent	TRUE	FALSE	integer	Medical history. This variable is populated when the reason for using anticoagulants or platelet aggregation inhibitors by the patient is cardiovascular, specifically a stent not specified elsewhere.	0=No;1=Yes
AnticoagulantReasonCardiovasStenosis	TRUE	FALSE	integer	Medical history. This variable is populated when the reason for using anticoagulants or platelet aggregation inhibitors by the patient is cardiovascular, specifically a cardiovascular stenosis	0=No;1=Yes
AnticoagulantReasonCardiovasTIS	TRUE	FALSE	integer	Medical history. This variable is populated when the reason for using anticoagulants or platelet aggregation inhibitors by the patient is cardiovascular, specifically a transient ischaemic attack/stroke	0=No;1=Yes
AnticoagulantReasonOther	TRUE	FALSE	integer	Medical history. This variable is populated when the reason for using anticoagulants or platelet aggregation inhibitors by the patient is not specified elsewhere.	0=No;1=Yes
AnticoagulantReasonOtherTxt	TRUE	FALSE	text	Medical history. This variable is populated when the reason for using anticoagulants or platelet aggregation inhibitors by the patient is not specified elsewhere (text field).	Not Applicable
AnticoagulantReasonThrombo	TRUE	FALSE	integer	Medical history. This variable is populated when the reason for using anticoagulants or platelet aggregation inhibitors by the patient is thromboembolic.	0=No;1=Yes
AnticoagulantReasonThromboDVTLess6	TRUE	FALSE	integer	Medical history. This variable is populated when the reason for using anticoagulants or platelet aggregation inhibitors by the patient is thromboembolic, specifically a single episode of DVT (deep venous thrombosis) or PE (pulmonary embolism) <6 months.	0=No;1=Yes
AnticoagulantReasonThromboDVTMore6	TRUE	FALSE	integer	Medical history. This variable is populated when the reason for using anticoagulants or platelet aggregation inhibitors by the patient is thromboembolic, specifically a single episode of DVT (deep venous thrombosis) or PE (pulmonary embolism) >6 months.	0=No;1=Yes
AnticoagulantReasonThromboMultipleEpisode	TRUE	FALSE	integer	Medical history. This variable is populated when the reason for using anticoagulants or platelet aggregation inhibitors by the patient is thromboembolic, specifically two or more episodes of DVT (deep venous thrombosis) or PE (pulmonary embolism).	0=No;1=Yes
AnticoagXarelto	TRUE	FALSE	integer	Medical history. Use of anticoagulant or platelet aggregation inhibitor by the patient. This variable describes the use of Xarelto	0=No;1=Yes
AUDITCAlcDrnkTypclDayNumScore	TRUE	FALSE	integer	On presentation the behavioral history of the patient was recorded. In case of past use of alcoholic beverages (beer, wine, spirits), this reflects the alcohol frequency: average number of drinks on a "drinking" day	1=1-2;2=3-4;3=5-6;4=7-9;5=10 or more;88=Unknown
AUDITCDrnkContainAlcFreqScore	TRUE	FALSE	integer	Detailed questions on the use of alcohol are derived from the first 3 questions of the "AUDIT" questionnaire, a screening tool developed by the World Health Organization (WHO) to assess alcohol consumption, drinking behaviors, and alcohol-related problems. The same questions are asked post-injury during full follow-up assessments (including cognitive testing.) This reflects the frequency of having a drink containing alcohol.	0=Never;1=Monthly or less;2=2-4 times a month;3=2-3 times a week;4=4 or more times a week;88=Unknown

Name	Static	Intervention	Format	Description	Possible Values
AUDITCMoreThan6AlcDrnkFreqScore	TRUE	FALSE	integer	Detailed questions on the use of alcohol are derived from the first 3 questions of the "AUDIT" questionnaire, a screening tool developed by the World Health Organization (WHO) to assess alcohol consumption, drinking behaviors, and alcohol-related problems. The same questions are asked post-injury during full follow-up assessments (including cognitive testing.) This reflects the frequency of having six or more drinks on one occasion.	0=Never;1=Monthly or less (incorrect please correct);2=2-4 times a month (incorrect please correct);3=2-3 times a week (incorrect please correct);4=4 or more times a week (incorrect please correct);5=Less than monthly;6=Monthly;7=Weekly;8=Daily or almost daily;88=Unknown
BetaBlocker	TRUE	FALSE	integer	A specific question on the use of beta-blockers is included as some reports indicate better outcome with the use of beta blockers (Research interest Rotterdam). If yes, specification is requested and differentiated into: Non-selective blockers/Selective beta-1 blockers/alpha-1 and beta blockers.	0=No;1=Yes;88=Unknown
BetaBlockerAlphaBucundolol	TRUE	FALSE	integer	Medical history. Use of anticoagulant or platelet aggregation inhibitor by the patient. This variable describes the use of a beta blocker, specifically Bucindolol.	0=No;1=Yes
BetaBlockerAlphaCarvedilol	TRUE	FALSE	integer	Medical history. Use of anticoagulant or platelet aggregation inhibitor by the patient. This variable describes the use of a beta blocker, specifically Carvedilol (Eucardic).	0=No;1=Yes
BetaBlockerAlphaLabetolol	TRUE	FALSE	integer	Medical history. Use of anticoagulant or platelet aggregation inhibitor by the patient. This variable describes the use of a beta blocker, specifically Labetalol (Trandate).	0=No;1=Yes
BetaBlockerAlphaOther	TRUE	FALSE	integer	Medical history. Use of anticoagulant or platelet aggregation inhibitor by the patient. This variable describes the use of an alpha 1 and beta-blocker, not specified elsewhere.	0=No;1=Yes
BetaBlockerAlphaOtherTxt	TRUE	FALSE	text	Medical history. Use of anticoagulant or platelet aggregation inhibitor by the patient. This variable describes the use of an alpha 1 and beta-blocker, not specified elsewhere (text field).	Not Applicable
BetaBlockerNonSelectCarteolol	TRUE	FALSE	integer	Medical history. Use of anticoagulant or platelet aggregation inhibitor by the patient. This variable describes the use of beta blockers, specifically Carteolol.	0=No;1=Yes
BetaBlockerNonSelectNadolol	TRUE	FALSE	integer	Medical history. Use of anticoagulant or platelet aggregation inhibitor by the patient. This variable describes the use of beta blockers, specifically Nadolol	0=No;1=Yes
BetaBlockerNonSelectOther	TRUE	FALSE	integer	Medical history. Use of anticoagulant or platelet aggregation inhibitor by the patient. This variable describes the use of beta blockers, nonselective, not specified elsewhere.	0=No;1=Yes
BetaBlockerNonSelectOtherTxt	TRUE	FALSE	text	Medical history. Use of anticoagulant or platelet aggregation inhibitor by the patient. This variable describes the use of beta blockers, nonselective, not specified elsewhere (textfield).	Not Applicable
BetaBlockerNonSelectPenbutolol	TRUE	FALSE	integer	Medical history. Use of anticoagulant or platelet aggregation inhibitor by the patient. This variable describes the use of beta blockers, specifically Penbutolo.	0=No;1=Yes
BetaBlockerNonSelectPindolol	TRUE	FALSE	integer	Medical history. Use of anticoagulant or platelet aggregation inhibitor by the patient. This variable describes the use of beta blockers, specifically Pindolol (Viskeen)	0=No;1=Yes
BetaBlockerNonSelectPropranolol	TRUE	FALSE	integer	Medical history. This variable describes the use of beta blockers, specifically Propranolol	0=No;1=Yes
BetaBlockerNonSelectSotalol	TRUE	FALSE	integer	Medical history. This variable describes the use of beta blockers, specifically Sotalol (Sotacor)	0=No;1=Yes
BetaBlockerSelectAcebutolol	TRUE	FALSE	integer	Medical history. This variable describes the use of beta blockers, specifically Acebutolol (Sectral)	0=No;1=Yes

Name	Static	Intervention	Format	Description	Possible Values
BetaBlockerSelectAtenolol	TRUE	FALSE	integer	Medical history. This variable describes the use of beta blockers, specifically Atenolol (Tenormin)	0=No;1=Yes
BetaBlockerSelectBetaxolol	TRUE	FALSE	integer	Medical history. This variable describes the use of beta blockers, specifically Betaxolol (Kerlon)	0=No;1=Yes
BetaBlockerSelectBisoprolol	TRUE	FALSE	integer	Medical history. This variable describes the use of beta blockers, specifically Bisoprolol (Emcor)	0=No;1=Yes
BetaBlockerSelectCeliprolol	TRUE	FALSE	integer	Medical history. This variable describes the use of beta blockers, specifically Celiprolol (Dilanorm)	0=No;1=Yes
BetaBlockerSelectEsmolol	TRUE	FALSE	integer	Medical history. This variable describes the use of beta blockers, specifically Esmolol (Brevibloc)	0=No;1=Yes
BetaBlockerSelectMetoprolol	TRUE	FALSE	integer	Medical history. This variable describes the use of beta blockers, specifically Metoprolol (Selokeen).	0=No;1=Yes
BetaBlockerSelectNebivolol	TRUE	FALSE	integer	Medical history. This variable describes the use of beta blockers, specifically Nebivolol (Nebilet)	0=No;1=Yes
BetaBlockerSelectOther	TRUE	FALSE	integer	Medical history. This variable describes the use of beta blockers, specifically selective beta1blockers not specified elsewhere.	0=No;1=Yes
CannabisCurrentUse	TRUE	FALSE	integer	On presentation the behavioral history of the patient was recorded. This reflects the use in the past three months of Cannabis (marijuana, pot, grass, hash, etc.)	0=No;1=Yes;88=Unknown
CannabisPriorUse	TRUE	FALSE	integer	On presentation the behavioral history of the patient was recorded. This reflects his past use of Cannabis (marijuana, pot, grass, hash, etc.)	0=No;1=Yes;88=Unknown
CannabisPriorUseDuration	TRUE	FALSE	text	On presentation the behavioral history of the patient was recorded. This reflects the number of years of past use of Cannabis if applicable.	Not Applicable
DrgSubIllctCurntUseInd	TRUE	FALSE	integer	On presentation the behavioral history of the patient was recorded. This reflects the use in the past three months of Other recreational drugs (than Cannabis)	0=No;1=Yes;88=Unknown
DrgSubIllctUseCatOther	TRUE	FALSE	text	On presentation the behavioral history of the patient was recorded. This reflects his past use of which type of drugs.	Not Applicable
DrgSubIllctUseDur	TRUE	FALSE	integer	On presentation the behavioral history of the patient was recorded. This reflects the number of years of his past use of recreational drugs, if applicable.	Not Applicable
DrgSubPriorIllctUseInd	TRUE	FALSE	integer	On presentation the behavioral history of the patient was recorded. This reflects his past use of Other recreational drugs (other than Cannabis)	0=No;1=Yes;88=Unknown
DrugIllicitCurrentUseOther	TRUE	FALSE	text	On presentation the behavioral history of the patient was recorded. This reflects the use in the past three months of which type of drugs	Not Applicable
MedHxAnticoagulantsOrPlatelet	TRUE	FALSE	integer	Summary question to document if the subject was taking anticoagulants or platelet aggregation inhibitors prior to injury. If yes, details are requested concerning which (groups of) agents were used. This information is of high relevance in relation to the shift of epidemiologic patterns in TBI towards higher age (with more co-morbidities and medication).	0=No;1=Yes anticoagulants;2=Yes platelet aggregation inhibitors;3=Yes, both;88=Unknown
MedHxCardio	TRUE	FALSE	integer	Details on Medical History are captured for 15 body regions/disease area's. If overall question for the body region/disease is "yes", more detailed info is requested and captured in the sub-domains. This variable is used for documenting cardiovascular medical history.	0=No;1=Yes;88=Unknown
MedHxCardioArrhythmia	TRUE	FALSE	integer	Details on Medical History are captured for 15 body regions/disease area's. If overall question for the body region/disease is "yes", more detailed info is requested and	0=No;1=Yes

Name	Static	Intervention	Format	Description	Possible Values
				captured in the sub-domains. This variable is used for documenting cardiovascular medical history, specifically arrhythmia.	
MedHxCardioCongenitalHD	TRUE	FALSE	integer	Details on Medical History are captured for 15 body regions/disease area's. If overall question for the body region/disease is "yes", more detailed info is requested and captured in the sub-domains. This variable is used for documenting cardiovascular medical history, specifically congenital heart disease.	0=No;1=Yes
MedHxCardioHTN	TRUE	FALSE	integer	Details on Medical History are captured for 15 body regions/disease area's. If overall question for the body region/disease is "yes", more detailed info is requested and captured in the sub-domains. This variable is used for documenting cardiovascular medical history, specifically hypertension	0=No;1=Yes
MedHxCardioIschemicHD	TRUE	FALSE	integer	Details on Medical History are captured for 15 body regions/disease area's. If overall question for the body region/disease is "yes", more detailed info is requested and captured in the sub-domains. This variable is used for documenting cardiovascular medical history, specifically Ischemic heart disease	0=No;1=Yes
MedHxCardioNYHA	TRUE	FALSE	text	Details on Medical History are captured for 15 body regions/disease area's. If overall question for the body region/disease is "yes", more detailed info is requested and captured in the sub-domains. This variable is used for documenting cardiovascular medical history, specifically NYHA, a classification system for severity of cardiac disease - generally used for ischaemia, but used here in broader sense.	I=I;II=II;III=III;IV=IV
MedHxCardioOther	TRUE	FALSE	integer	Details on Medical History are captured for 15 body regions/disease area's. If overall question for the body region/disease is "yes", more detailed info is requested and captured in the sub-domains. This variable is used for documenting cardiovascular medical history, not specified elsewhere.	0=No;1=Yes
MedHxCardioOtherTxt	TRUE	FALSE	text	Details on Medical History are captured for 15 body regions/disease area's. If overall question for the body region/disease is "yes", more detailed info is requested and captured in the sub-domains. This variable is used for documenting cardiovascular medical history, not specified elsewhere (textfield)	Not Applicable
MedHxCardioPeripheralVascular	TRUE	FALSE	integer	Details on Medical History are captured for 15 body regions/disease area's. If overall question for the body region/disease is "yes", more detailed info is requested and captured in the sub-domains. This variable is used for documenting cardiovascular medical history, specifically peripheral vascular disease.	0=No;1=Yes
MedHxCardioThromboembolic	TRUE	FALSE	integer	Details on Medical History are captured for 15 body regions/disease area's. If overall question for the body region/disease is "yes", more detailed info is requested and captured in the sub-domains. This variable is used for documenting cardiovascular medical history, specifically thromboembolic	0=No;1=Yes
MedHxCardioValvularHD	TRUE	FALSE	integer	Details on Medical History are captured for 15 body regions/disease area's. If overall question for the body region/disease is "yes", more detailed info is requested and captured in the sub-domains. This variable is used for documenting cardiovascular	0=No;1=Yes

Name	Static	Intervention	Format	Description	Possible Values
				medical history, specifically valvular heart disease	
MedHxDevelopmental	TRUE	FALSE	integer	Details on Medical History are captured for 15 body regions/disease area's. If overall question for the body region/disease is "yes", more detailed info is requested and captured in the sub-domains. This variable is used for documenting developmental diseases.	0=No;1=Yes;88=Unknown
MedHxDevelopmentalADDandADHD	TRUE	FALSE	integer	Details on Medical History are captured for 15 body regions/disease area's. If overall question for the body region/disease is "yes", more detailed info is requested and captured in the sub-domains. This variable is used for documenting developmental diseases, specifically attention deficit/hyperactivity disorder.	0=No;1=Yes
MedHxDevelopmentalLearningDisability	TRUE	FALSE	integer	Details on Medical History are captured for 15 body regions/disease area's. If overall question for the body region/disease is "yes", more detailed info is requested and captured in the sub-domains. This variable is used for documenting developmental diseases, specifically learning disability	0=No;1=Yes
MedHxDevelopmentalOther	TRUE	FALSE	integer	Details on Medical History are captured for 15 body regions/disease area's. If overall question for the body region/disease is "yes", more detailed info is requested and captured in the sub-domains. This variable is used for documenting developmental diseases that are not specified elsewhere.	0=No;1=Yes
MedHxDevelopmentalOtherTxt	TRUE	FALSE	text	Details on Medical History are captured for 15 body regions/disease area's. If overall question for the body region/disease is "yes", more detailed info is requested and captured in the sub-domains. This variable is used for documenting developmental diseases that are not specified elsewhere (textfield).	Not Applicable
MedHxEndocrine	TRUE	FALSE	integer	Details on Medical History are captured for 15 body regions/disease area's. If overall question for the body region/disease is "yes", more detailed info is requested and captured in the sub-domains. This variable is used for documenting endocrine diseases	0=No;1=Yes;88=Unknown
MedHxEndocrineIDDM	TRUE	FALSE	integer	Details on Medical History are captured for 15 body regions/disease area's. If overall question for the body region/disease is "yes", more detailed info is requested and captured in the sub-domains. This variable is used for documenting endocrine diseases, specifically IDDM (Insulin dependent diabetes mellitus)	0=No;1=Yes
MedHxEndocrineIDDMControl	TRUE	FALSE	integer	Details on Medical History are captured for 15 body regions/disease area's. If overall question for the body region/disease is "yes", more detailed info is requested and captured in the sub-domains. This variable is used for documenting for endocrine diseases, specifically IDDM (Insulin dependent diabetes mellitus) - how well it is controlled.	1=Well controlled;2=Difficult controlled;88=Unknown
MedHxEndocrineNIDDM	TRUE	FALSE	integer	Details on Medical History are captured for 15 body regions/disease area's. If overall question for the body region/disease is "yes", more detailed info is requested and captured in the sub-domains. This variable is used for documenting endocrine diseases, specifically NIDDM (Non-insulin dependent diabetes mellitus)	0=No;1=Yes
MedHxEndocrineNIDDMControl	TRUE	FALSE	integer	Details on Medical History are captured for 15 body regions/disease area's. If overall question for the body region/disease is	1=Well controlled;2=Difficult controlled;88=Unknown

Name	Static	Intervention	Format	Description	Possible Values
				"yes", more detailed info is requested and captured in the sub-domains. This variable is used for documenting for endocrine diseases, specifically NIDDM (Non-insulin dependent diabetes mellitus), how well it is controlled.	
MedHxEndocrineOther	TRUE	FALSE	integer	Details on Medical History are captured for 15 body regions/disease area's. If overall question for the body region/disease is "yes", more detailed info is requested and captured in the sub-domains. This variable is used for documenting endocrine diseases not mentioned elsewhere.	0=No;1=Yes
MedHxEndocrineOtherTxt	TRUE	FALSE	text	Details on Medical History are captured for 15 body regions/disease area's. If overall question for the body region/disease is "yes", more detailed info is requested and captured in the sub-domains. This variable is used for documenting endocrine diseases not specified elsewhere (textfield).	Not Applicable
MedHxEndocrineThyroid	TRUE	FALSE	integer	Details on Medical History are captured for 15 body regions/disease area's. If overall question for the body region/disease is "yes", more detailed info is requested and captured in the sub-domains. This variable is used for documenting endocrine diseases, specifically thyroid disorder.	0=No;1=Yes
MedHxENT	TRUE	FALSE	integer	Details on Medical History are captured for 15 body regions/disease area's. If overall question for the body region/disease is "yes", more detailed info is requested and captured in the sub-domains. This variable is used for documenting ENT (Eye, Ear, Nose & Throat) disease	0=No;1=Yes;88=Unknown
MedHxENTHearing	TRUE	FALSE	integer	Details on Medical History are captured for 15 body regions/disease area's. If overall question for the body region/disease is "yes", more detailed info is requested and captured in the sub-domains. This variable is used for documenting ENT (Eye, Ear, Nose & Throat) disease, specifically hearing deficits.	0=No;1=Yes
MedHxENTOther	TRUE	FALSE	integer	Details on Medical History are captured for 15 body regions/disease area's. If overall question for the body region/disease is "yes", more detailed info is requested and captured in the sub-domains. This variable is used for documenting ENT (Eye, Ear, Nose & Throat) diseases not specified elsewhere.	0=No;1=Yes
MedHxENTOtherTxt	TRUE	FALSE	text	Details on Medical History are captured for 15 body regions/disease area's. If overall question for the body region/disease is "yes", more detailed info is requested and captured in the sub-domains. This variable is used for documenting ENT (Eye, Ear, Nose & Throat) diseases not specified elsewhere (textfield).	Not Applicable
MedHxENTSinusitis	TRUE	FALSE	integer	Details on Medical History are captured for 15 body regions/disease area's. If overall question for the body region/disease is "yes", more detailed info is requested and captured in the sub-domains. This variable is used for documenting ENT (Eye, Ear, Nose & Throat) diseases, specifically sinusitis.	0=No;1=Yes
MedHxENTVisionAbn	TRUE	FALSE	integer	Details on Medical History are captured for 15 body regions/disease area's. If overall question for the body region/disease is "yes", more detailed info is requested and captured in the sub-domains. This variable is used for documenting ENT (Eye, Ear, Nose & Throat) diseases, specifically vision.	0=No;1=Yes
MedHxGastro	TRUE	FALSE	integer	Details on Medical History are captured for 15 body regions/disease area's. If overall question for the body region/disease is	0=No;1=Yes;88=Unknown

Name	Static	Intervention	Format	Description	Possible Values
				"yes", more detailed info is requested and captured in the sub-domains. This variable is used for documenting gastrointestinal disease.	
MedHxGastroGERD	TRUE	FALSE	integer	Details on Medical History are captured for 15 body regions/disease area's. If overall question for the body region/disease is "yes", more detailed info is requested and captured in the sub-domains. This variable is used for documenting gastrointestinal disease, specifically GERD (Gastroesophageal Reflux Disease).	0=No;1=Yes
MedHxGastroGIBleed	TRUE	FALSE	integer	Details on Medical History are captured for 15 body regions/disease area's. If overall question for the body region/disease is "yes", more detailed info is requested and captured in the sub-domains. This variable is used for documenting gastrointestinal disease, specifically gastrointestinal bleeding.	0=No;1=Yes
MedHxGastroIBS	TRUE	FALSE	integer	Details on Medical History are captured for 15 body regions/disease area's. If overall question for the body region/disease is "yes", more detailed info is requested and captured in the sub-domains. This variable is used for documenting gastrointestinal disease, specifically inflammatory bowel disease.	0=No;1=Yes
MedHxGastroOther	TRUE	FALSE	integer	Details on Medical History are captured for 15 body regions/disease area's. If overall question for the body region/disease is "yes", more detailed info is requested and captured in the sub-domains. This variable is used for documenting gastrointestinal disease not specified elsewhere.	0=No;1=Yes
MedHxGastroOtherTxt	TRUE	FALSE	text	Details on Medical History are captured for 15 body regions/disease area's. If overall question for the body region/disease is "yes", more detailed info is requested and captured in the sub-domains. This variable is used for documenting gastrointestinal disease not specified elsewhere (textfield).	Not Applicable
MedHxHematologic	TRUE	FALSE	integer	Details on Medical History are captured for 15 body regions/disease area's. If overall question for the body region/disease is "yes", more detailed info is requested and captured in the sub-domains. This variable is used for documenting hematologic diseases.	0=No;1=Yes;88=Unknown
MedHxHematologicAIDS	TRUE	FALSE	integer	Details on Medical History are captured for 15 body regions/disease area's. If overall question for the body region/disease is "yes", more detailed info is requested and captured in the sub-domains. This variable is used for documenting hematologic diseases, specifically AIDS	0=No;1=Yes
MedHxHematologicAnemia	TRUE	FALSE	integer	Details on Medical History are captured for 15 body regions/disease area's. If overall question for the body region/disease is "yes", more detailed info is requested and captured in the sub-domains. This variable is used for documenting hematologic diseases, like anemia.	0=No;1=Yes
MedHxHematologicHIV	TRUE	FALSE	integer	Details on Medical History are captured for 15 body regions/disease area's. If overall question for the body region/disease is "yes", more detailed info is requested and captured in the sub-domains. This variable is used for documenting hematologic diseases, like HIV positive.	0=No;1=Yes
MedHxHematologicOther	TRUE	FALSE	integer	Details on Medical History are captured for 15 body regions/disease area's. If overall question for the body region/disease is "yes", more detailed info is requested and captured in the sub-domains. This variable is	0=No;1=Yes

Name	Static	Intervention	Format	Description	Possible Values
				used for documenting hematologic diseases, not specified elsewhere.	
MedHxHematologicOtherTxt	TRUE	FALSE	text	Details on Medical History are captured for 15 body regions/disease area's. If overall question for the body region/disease is "yes", more detailed info is requested and captured in the sub-domains. This variable is used for documenting hematologic diseases, not specified elsewhere (textfield).	Not Applicable
MedHxHematologicSickleCell	TRUE	FALSE	integer	Details on Medical History are captured for 15 body regions/disease area's. If overall question for the body region/disease is "yes", more detailed info is requested and captured in the sub-domains. This variable is used for documenting hematologic diseases, specifically sickle cell disease.	0=No;1=Yes
MedHxHepatic	TRUE	FALSE	integer	Details on Medical History are captured for 15 body regions/disease area's. If overall question for the body region/disease is "yes", more detailed info is requested and captured in the sub-domains. This variable is used for documenting hepatic diseases.	0=No;1=Yes;88=Unknown
MedHxHepaticCirrhosis	TRUE	FALSE	integer	Details on Medical History are captured for 15 body regions/disease area's. If overall question for the body region/disease is "yes", more detailed info is requested and captured in the sub-domains. This variable is used for documenting hepatic diseases, specifically cirrhosis.	0=No;1=Yes
MedHxHepaticFailure	TRUE	FALSE	integer	Details on Medical History are captured for 15 body regions/disease area's. If overall question for the body region/disease is "yes", more detailed info is requested and captured in the sub-domains. This variable is used for documenting hepatic diseases, specifically hepatic failure	0=No;1=Yes
MedHxHepaticHepatitis	TRUE	FALSE	integer	Details on Medical History are captured for 15 body regions/disease area's. If overall question for the body region/disease is "yes", more detailed info is requested and captured in the sub-domains. This variable is used for documenting hepatic diseases, specifically hepatitis.	0=No;1=Yes
MedHxHepaticInsufficiency	TRUE	FALSE	integer	Details on Medical History are captured for 15 body regions/disease area's. If overall question for the body region/disease is "yes", more detailed info is requested and captured in the sub-domains. This variable is used for documenting hepatic diseases, specifically hepatic insufficiency.	0=No;1=Yes
MedHxHepaticOther	TRUE	FALSE	integer	Details on Medical History are captured for 15 body regions/disease area's. If overall question for the body region/disease is "yes", more detailed info is requested and captured in the sub-domains. This variable is used for documenting hepatic diseases not specified elsewhere	0=No;1=Yes
MedHxHepaticOtherTxt	TRUE	FALSE	text	Details on Medical History are captured for 15 body regions/disease area's. If overall question for the body region/disease is "yes", more detailed info is requested and captured in the sub-domains. This variable is used for documenting hepatic diseases not specified elsewhere (textfield)	Not Applicable
MedHxMusculoskeletal	TRUE	FALSE	integer	Details on Medical History are captured for 15 body regions/disease area's. If overall question for the body region/disease is "yes", more detailed info is requested and captured in the sub-domains. This variable is used for documenting musculoskeletal diseases	0=No;1=Yes;88=Unknown
MedHxMusculoskeletalArthritis	TRUE	FALSE	integer	Details on Medical History are captured for 15 body regions/disease area's. If overall question for the body region/disease is	0=No;1=Yes

Name	Static	Intervention	Format	Description	Possible Values
				"yes", more detailed info is requested and captured in the sub-domains. This variable is used for documenting musculoskeletal diseases, specifically arthritis	
MedHxMusculoskeletalOther	TRUE	FALSE	integer	Details on Medical History are captured for 15 body regions/disease area's. If overall question for the body region/disease is "yes", more detailed info is requested and captured in the sub-domains. This variable is used for documenting musculoskeletal diseases not specified elsewhere.	0=No;1=Yes
MedHxMusculoskeletalOtherTxt	TRUE	FALSE	text	Details on Medical History are captured for 15 body regions/disease area's. If overall question for the body region/disease is "yes", more detailed info is requested and captured in the sub-domains. This variable is used for documenting musculoskeletal diseases not specified elsewhere (textfield)	Not Applicable
MedHxNeuro	TRUE	FALSE	integer	Details on Medical History are captured for 15 body regions/disease area's. If overall question for the body region/disease is "yes", more detailed info is requested and captured in the sub-domains. This variable is used for documenting neurological diseases.	0=No;1=Yes;88=Unknown
MedHxNeuroCerebrovascularAccident	TRUE	FALSE	integer	Details on Medical History are captured for 15 body regions/disease area's. If overall question for the body region/disease is "yes", more detailed info is requested and captured in the sub-domains. This variable is used for documenting neurological diseases, specifically cerebrovascular accidents.	0=No;1=Yes
MedHxNeuroEpilepsyGeneralized	TRUE	FALSE	integer	Details on Medical History are captured for 15 body regions/disease area's. If overall question for the body region/disease is "yes", more detailed info is requested and captured in the sub-domains. This variable is used for documenting neurological diseases, specifically epilepsy (generalized).	0=No;1=Yes
MedHxNeuroEpilepsyOther	TRUE	FALSE	integer	Details on Medical History are captured for 15 body regions/disease area's. If overall question for the body region/disease is "yes", more detailed info is requested and captured in the sub-domains. This variable is used for documenting neurological diseases, specifically epilepsy (other).	0=No;1=Yes
MedHxNeuroEpilepsyPartial	TRUE	FALSE	integer	Details on Medical History are captured for 15 body regions/disease area's. If overall question for the body region/disease is "yes", more detailed info is requested and captured in the sub-domains. This variable is used for documenting neurological diseases, specifically epilepsy (partial).	0=No;1=Yes
MedHxNeuroFebrileSeizures	TRUE	FALSE	integer	Details on Medical History are captured for 15 body regions/disease area's. If overall question for the body region/disease is "yes", more detailed info is requested and captured in the sub-domains. This variable is used for documenting neurological diseases, specifically febrile seizures (children).	0=No;1=Yes
MedHxNeuroHeadache	TRUE	FALSE	integer	Details on Medical History are captured for 15 body regions/disease area's. If overall question for the body region/disease is "yes", more detailed info is requested and captured in the sub-domains. This variable is used for documenting neurological diseases, specifically headache (non migraine).	0=No;1=Yes
MedHxNeuroMigraine	TRUE	FALSE	integer	Details on Medical History are captured for 15 body regions/disease area's. If overall question for the body region/disease is "yes", more detailed info is requested and captured in the sub-domains. This variable is used for documenting neurological diseases, specifically migraines.	0=No;1=Yes

Name	Static	Intervention	Format	Description	Possible Values
MedHxNeuroMigraineFamHist	TRUE	FALSE	integer	Details on Medical History are captured for 15 body regions/disease area's. If overall question for the body region/disease is "yes", more detailed info is requested and captured in the sub-domains. This variable is used for documenting neurological diseases, specifically family history of migraine.	0=No;1=Yes
MedHxNeuroOther	TRUE	FALSE	integer	Details on Medical History are captured for 15 body regions/disease area's. If overall question for the body region/disease is "yes", more detailed info is requested and captured in the sub-domains. This variable is used for documenting neurological diseases not specified elsewhere.	0=No;1=Yes
MedHxNeuroOtherTxt	TRUE	FALSE	text	Details on Medical History are captured for 15 body regions/disease area's. If overall question for the body region/disease is "yes", more detailed info is requested and captured in the sub-domains. This variable is used for documenting neurological diseases not specified elsewhere (textfield)	Not Applicable
MedHxNeuroPain	TRUE	FALSE	integer	Details on Medical History are captured for 15 body regions/disease area's. If overall question for the body region/disease is "yes", more detailed info is requested and captured in the sub-domains. This variable is used for documenting neurological diseases not specified elsewhere (textfield)	0=No;1=Yes;88=Unknown
MedHxNeuroTIA	TRUE	FALSE	integer	Details on Medical History are captured for 15 body regions/disease area's. If overall question for the body region/disease is "yes", more detailed info is requested and captured in the sub-domains. This variable is used for documenting neurological diseases, specifically transient ischemic attacks	0=No;1=Yes
MedHxOncologic	TRUE	FALSE	integer	Details on Medical History are captured for 15 body regions/disease area's. If overall question for the body region/disease is "yes", more detailed info is requested and captured in the sub-domains. This variable is used for documenting oncologic diseases.	0=No;1=Yes;88=Unknown
MedHxOncologicBreast	TRUE	FALSE	integer	Details on Medical History are captured for 15 body regions/disease area's. If overall question for the body region/disease is "yes", more detailed info is requested and captured in the sub-domains. This variable is used for documenting oncologic diseases, like breast cancer.	0=No;1=Yes
MedHxOncologicGI	TRUE	FALSE	integer	Details on Medical History are captured for 15 body regions/disease area's. If overall question for the body region/disease is "yes", more detailed info is requested and captured in the sub-domains. This variable is used for documenting oncologic diseases, specifically gastrointestinal cancer.	0=No;1=Yes
MedHxOncologicKidney	TRUE	FALSE	integer	Details on Medical History are captured for 15 body regions/disease area's. If overall question for the body region/disease is "yes", more detailed info is requested and captured in the sub-domains. This variable is used for documenting oncologic diseases, specifically kidney cancer.	0=No;1=Yes
MedHxOncologicLeukemia	TRUE	FALSE	integer	Details on Medical History are captured for 15 body regions/disease area's. If overall question for the body region/disease is "yes", more detailed info is requested and captured in the sub-domains. This variable is used for documenting oncologic diseases, specifically leukemia.	0=No;1=Yes
MedHxOncologicLung	TRUE	FALSE	integer	Details on Medical History are captured for 15 body regions/disease area's. If overall question for the body region/disease is "yes", more detailed info is requested and captured in the sub-domains. This variable is	0=No;1=Yes

Name	Static	Intervention	Format	Description	Possible Values
				used for documenting oncologic diseases, specifically lung cancer.	
MedHxOncologicLymphoma	TRUE	FALSE	integer	Details on Medical History are captured for 15 body regions/disease area's. If overall question for the body region/disease is "yes", more detailed info is requested and captured in the sub-domains. This variable is used for documenting oncologic diseases, specifically lymphoma.	0=No;1=Yes
MedHxOncologicOther	TRUE	FALSE	integer	Details on Medical History are captured for 15 body regions/disease area's. If overall question for the body region/disease is "yes", more detailed info is requested and captured in the sub-domains. This variable is used for documenting oncologic diseases not specified elsewhere	0=No;1=Yes
MedHxOncologicOtherTxt	TRUE	FALSE	text	Details on Medical History are captured for 15 body regions/disease area's. If overall question for the body region/disease is "yes", more detailed info is requested and captured in the sub-domains. This variable is used for documenting oncologic diseases not specified elsewhere (textfield).	Not Applicable
MedHxOncologicProstate	TRUE	FALSE	integer	Details on Medical History are captured for 15 body regions/disease area's. If overall question for the body region/disease is "yes", more detailed info is requested and captured in the sub-domains. This variable is used for documenting oncologic, specifically prostate cancer.	0=No;1=Yes
MedHxOther	TRUE	FALSE	integer	Details on Medical History are captured for 15 body regions/disease area's. If overall question for the body region/disease is "yes", more detailed info is requested and captured in the sub-domains. This variable is used for documenting other medical history, not specified elsewhere.	0=No;1=Yes
MedHxOtherTxt	TRUE	FALSE	text	Details on Medical History are captured for 15 body regions/disease area's. If overall question for the body region/disease is "yes", more detailed info is requested and captured in the sub-domains. This variable is used for documenting other medical history, not specified elsewhere (textfield)	Not Applicable
MedHxPreInjASAPSClass	TRUE	FALSE	integer	Preinjury ASAPS classification. Common classification system used in anaesthesia; denotes overall health	1=A normal healthy patient;2=A patient with mild systemic disease;3=A patient with severe systemic disease;4=A patient with a severe systemic disease that is a constant threat to life;88=Unknown
MedHxPreTBIConcussions	TRUE	FALSE	integer	Details on Medical History are captured for 15 body regions/disease area's. If overall question for the body region/disease is "yes", more detailed info is requested and captured in the sub-domains. This variable is used for documenting previous TBI/ concussions	0=No;1=Yes;88=Unknown
MedHxPreTBIConcussionsTotal	TRUE	FALSE	integer	Details on Medical History are captured for 15 body regions/disease area's. If overall question for the body region/disease is "yes", more detailed info is requested and captured in the sub-domains. This variable is used for documenting total number of previous TBI/ concussions	Not Applicable
MedHxPreTBIConcussionsTotalHosAdmit	TRUE	FALSE	integer	Details on Medical History are captured for 15 body regions/disease area's. If overall question for the body region/disease is "yes", more detailed info is requested and captured in the sub-domains. This variable is used for documenting total number of hospital admissions for previous TBI/ concussions	Not Applicable
MedHxPsychiatric	TRUE	FALSE	integer	Details on Medical History are captured for 15 body regions/disease area's. If overall question for the body region/disease is "yes", more detailed info is requested and	0=No;1=Yes;88=Unknown

Name	Static	Intervention	Format	Description	Possible Values
				captured in the sub-domains. This variable is used for documenting psychiatric diseases.	
MedHxPsychiatricAnx	TRUE	FALSE	integer	Details on Medical History are captured for 15 body regions/disease area's. If overall question for the body region/disease is "yes", more detailed info is requested and captured in the sub-domains. This variable is used for documenting psychiatric diseases, specifically anxiety.	0=No;1=Yes
MedHxPsychiatricDep	TRUE	FALSE	integer	Details on Medical History are captured for 15 body regions/disease area's. If overall question for the body region/disease is "yes", more detailed info is requested and captured in the sub-domains. This variable is used for documenting psychiatric diseases, specifically depression.	0=No;1=Yes
MedHxPsychiatricOther	TRUE	FALSE	integer	Details on Medical History are captured for 15 body regions/disease area's. If overall question for the body region/disease is "yes", more detailed info is requested and captured in the sub-domains. This variable is used for documenting psychiatric diseases not documented elsewhere.	0=No;1=Yes
MedHxPsychiatricOtherTxt	TRUE	FALSE	text	Details on Medical History are captured for 15 body regions/disease area's. If overall question for the body region/disease is "yes", more detailed info is requested and captured in the sub-domains. This variable is used for documenting psychiatric diseases not documented elsewhere (textfield).	Not Applicable
MedHxPsychiatricSchiz	TRUE	FALSE	integer	Details on Medical History are captured for 15 body regions/disease area's. If overall question for the body region/disease is "yes", more detailed info is requested and captured in the sub-domains. This variable is used for documenting psychiatric diseases, specifically schizophrenia.	0=No;1=Yes
MedHxPsychiatricSleep	TRUE	FALSE	integer	Details on Medical History are captured for 15 body regions/disease area's. If overall question for the body region/disease is "yes", more detailed info is requested and captured in the sub-domains. This variable is used for documenting psychiatric diseases, specifically sleep disorders.	0=No;1=Yes
MedHxPsychiatricSubstanceAbuse	TRUE	FALSE	integer	Details on Medical History are captured for 15 body regions/disease area's. If overall question for the body region/disease is "yes", more detailed info is requested and captured in the sub-domains. This variable is used for documenting psychiatric diseases, specifically substance abuse disorders.	0=No;1=Yes
MedHxPulmonary	TRUE	FALSE	integer	Details on Medical History are captured for 15 body regions/disease area's. If overall question for the body region/disease is "yes", more detailed info is requested and captured in the sub-domains. This variable is used for documenting pulmonary diseases	0=No;1=Yes;88=Unknown
MedHxPulmonaryAsthma	TRUE	FALSE	integer	Details on Medical History are captured for 15 body regions/disease area's. If overall question for the body region/disease is "yes", more detailed info is requested and captured in the sub-domains. This variable is used for documenting pulmonary diseases, specifically asthma.	0=No;1=Yes
MedHxPulmonaryCOPD	TRUE	FALSE	integer	Details on Medical History are captured for 15 body regions/disease area's. If overall question for the body region/disease is "yes", more detailed info is requested and captured in the sub-domains. This variable is used for documenting pulmonary diseases, specifically COPD (Chronic Obstructive Pulmonary Disease)	0=No;1=Yes
MedHxPulmonaryOther	TRUE	FALSE	integer	Details on Medical History are captured for 15 body regions/disease area's. If overall	0=No;1=Yes

Name	Static	Intervention	Format	Description	Possible Values
				question for the body region/disease is "yes", more detailed info is requested and captured in the sub-domains. This variable is used for documenting pulmonary diseases not specified elsewhere.	
MedHxPulmonaryOtherTxt	TRUE	FALSE	text	Details on Medical History are captured for 15 body regions/disease area's. If overall question for the body region/disease is "yes", more detailed info is requested and captured in the sub-domains. This variable is used for documenting pulmonary diseases not specified elsewhere (textfield).	Not Applicable
MedHxPulmonaryPneumonia	TRUE	FALSE	integer	Details on Medical History are captured for 15 body regions/disease area's. If overall question for the body region/disease is "yes", more detailed info is requested and captured in the sub-domains. This variable is used for documenting pulmonary diseases, specifically pneumonia.	0=No;1=Yes
MedHxPulmonaryTB	TRUE	FALSE	integer	Details on Medical History are captured for 15 body regions/disease area's. If overall question for the body region/disease is "yes", more detailed info is requested and captured in the sub-domains. This variable is used for documenting pulmonary diseases, specifically tuberculosis.	0=No;1=Yes
MedHxRenal	TRUE	FALSE	integer	Details on Medical History are captured for 15 body regions/disease area's. If overall question for the body region/disease is "yes", more detailed info is requested and captured in the sub-domains. This variable is used for documenting renal diseases.	0=No;1=Yes;88=Unknown
MedHxRenalFailure	TRUE	FALSE	integer	Details on Medical History are captured for 15 body regions/disease area's. If overall question for the body region/disease is "yes", more detailed info is requested and captured in the sub-domains. This variable is used for documenting renal diseases, specifically renal failure.	0=No;1=Yes
MedHxRenalInsufficiency	TRUE	FALSE	integer	Details on Medical History are captured for 15 body regions/disease area's. If overall question for the body region/disease is "yes", more detailed info is requested and captured in the sub-domains. This variable is used for documenting renal diseases, specifically renal insufficiency.	0=No;1=Yes
MedHxRenalOther	TRUE	FALSE	integer	Details on Medical History are captured for 15 body regions/disease area's. If overall question for the body region/disease is "yes", more detailed info is requested and captured in the sub-domains. This variable is used for documenting renal diseases not specified elsewhere.	0=No;1=Yes
MedHxRenalOtherTxt	TRUE	FALSE	text	Details on Medical History are captured for 15 body regions/disease area's. If overall question for the body region/disease is "yes", more detailed info is requested and captured in the sub-domains. This variable is used for documenting renal diseases not specified elsewhere (textfield).	Not Applicable
MedHxRenalUTI	TRUE	FALSE	integer	Details on Medical History are captured for 15 body regions/disease area's. If overall question for the body region/disease is "yes", more detailed info is requested and captured in the sub-domains. This variable is used for documenting renal diseases, specifically chronic UTI (urinary tract infection).	0=No;1=Yes
PlateletAggreOther	TRUE	FALSE	integer	Medical history. Variable documents the use of anticoagulants or platelet aggregation inhibitors. This variable contains the medication of this type not specified elsewhere.	0=No;1=Yes

Name	Static	Intervention	Format	Description	Possible Values
PlateletAggreOtherTxt	TRUE	FALSE	text	Medical history. Variable documents the use of anticoagulants or platelet aggregation inhibitors. This variable contains the medication of this type not specified elsewhere (textfield).	Not Applicable
PltAggregAdenosineInhib	TRUE	FALSE	integer	Medical history. Use of platelet aggregation inhibitor by the patient. This variable describes the use of an adenosine reuptake inhibitor (eg. Persantin, dipyridamole).	0=No;1=Yes
PltAggregADPReceptInhib	TRUE	FALSE	integer	Medical history. Use of platelet aggregation inhibitor by the patient. This variable describes the use of ADP receptor inhibitors.	0=No;1=Yes
PltAggregADPReceptInhibEffient	TRUE	FALSE	integer	Medical history. Use of platelet aggregation inhibitor by the patient. This variable describes the use of Parasugrel (Effient)	0=No;1=Yes
PltAggregADPReceptInhibOther	TRUE	FALSE	integer	Medical history. Use of platelet aggregation inhibitor by the patient. This variable describes the use of ADP receptor inhibitors, not specified elsewhere.	0=No;1=Yes
PltAggregADPReceptInhibOtherTxt	TRUE	FALSE	text	Medical history. Use of platelet aggregation inhibitor by the patient. This variable describes the use of ADP receptor inhibitors, not specified elsewhere (textfield).	Not Applicable
PltAggregADPReceptInhibPlavix	TRUE	FALSE	integer	Medical history. Use of platelet aggregation inhibitor by the patient. This variable describes the use of Clopidogrel (Plavix).	0=No;1=Yes
PltAggregADPReceptInhibTiclid	TRUE	FALSE	integer	Medical history. Use of platelet aggregation inhibitor by the patient. This variable describes the use of Ticlopidine (Ticlid).	0=No;1=Yes
PltAggregAspirin	TRUE	FALSE	integer	Medical history. Use of platelet aggregation inhibitor by the patient. This variable describes the use of Aspirin	0=No;1=Yes
PltAggregGlycoproteinInhib	TRUE	FALSE	integer	Medical history. Use of platelet aggregation inhibitor by the patient. This variable describes the use of glycoprotein IIB/IIIA inhibitors (eg. Aggrastat).	0=No;1=Yes
PmMedicalCode	TRUE	FALSE	integer	Medical History of patients has been recorded under MedHx.MedHx* A number of predefined medical conditions were coded in the e-CRF. If the patient was taking medication for this predefined medical condition, the same code needed to be entered here. An option other was also available for conditions not listed. Please check MedHx.MedHx* for the information on the medical condition(s) corresponding to this variable.	Not Applicable
PmMedicationName	TRUE	FALSE	text	These fields capture information on medication taken pre-injury. For each medication taken, the corresponding Medical History code is listed to document the reason for which this med was taken. If the medication taken was not linked to one of the recorded medication history codes, Investigators were asked to enter "777" as related history code.	Not Applicable
PriorMeds	TRUE	FALSE			Not Applicable
SedativeCurrentUse	TRUE	FALSE	integer	On presentation the behavioral history of the patient was recorded. This reflects if in the past three months the subjects used sedatives or sleeping pills.	0=No;1=Yes;88=Unknown
SedativePriorUse	TRUE	FALSE	integer	On presentation the behavioral history of the patient was recorded. This reflects his past use of Sedatives or sleeping pill.	0=No;1=Yes;88=Unknown
SedativePriorUseDuration	TRUE	FALSE	text	On presentation the behavioral history of the patient was recorded. This reflects the number sof years of his past use sedatives (if applicable).	Not Applicable
TobcoCurntUseInd	TRUE	FALSE	integer	On presentation the behavioral history of the patient was recorded. This reflects his use in the past three months of Tobacco products (cigarettes, cigars, pipe, chewing tobacco, etc.)	0=No;1=Yes;88=Unknown

Name	Static	Intervention	Format	Description	Possible Values
TobcoPriorUseInd	TRUE	FALSE	integer	The form "Behavioral History" captures information on past and current use of alcohol, tobacco, sedatives/sleeping pills, cannabis and other recreational drugs. Use is differentiated as "Past user" (eg stopped) versus "use in the past 3 months. Note: These variables do not reflect use of these substances at the time of injury.	0=No;1=Yes;88=Unknown
TobcoUseDur	TRUE	FALSE	integer	On presentation the behavioral history of the patient was recorded. This reflects the number of years of his past use of Tobacco (if applicable).	Not Applicable

Injury characteristics and severity

Name	Static	Intervention	Format	Description	Possible Values
AbdomenPelvicContentsAIS	TRUE	FALSE	integer	AIS score for the Abdomen/Pelvic Contents In the original AIS classification of injury severity, the grading is from 1 (minor) to 6 (unsurvivable). We added a score of 0 to designate absence of injuries.	0=None;1=Minor: no treatment needed;2=Moderate: requires only outpatient treatment;3=Serious: requires non-ICU hospital admission;4=Severe: requires ICU observation and/or basic treatment;5=Critical: requires intubation, mechanical ventilation or vasopressors for blood pressure support;6=Unsurvivable: not survivable
AbdomenPelvicContentsDesc	TRUE	FALSE	text	Injury description related to the AIS/ISS score for the Abdomen/Pelvic Contents	Not Applicable
BestOfAbdomenPelvicLumbarISS	TRUE	FALSE	integer	AbdomenPelvicLumbar region (Highest AIS of the region)^2 compare AbdomenPelvicContentsAIS, LumbarSpineAIS. This score is taken forward for ISS calculation	Not Applicable
BestOfChestSpineISS	TRUE	FALSE	integer	(highest AIS of the region)^2 Compare ThoraxChestAIS, ThoracicSpineAIS and select the highest for ISS calculation	Not Applicable
BestOfExternaISS	TRUE	FALSE	integer	External region (ExternaAIS)^2 select the highest external AIS severity code for ISS calculation.	Not Applicable
BestOfExtremitiesISS	TRUE	FALSE	integer	Extremities region (Highest AIS of the region)^2 compare UpperExtremitiesAIS, LowerExtremitiesAIS, PelvicGirdleAIS select the highest for ISS calculation	Not Applicable
BestOfFaceISS	TRUE	FALSE	integer	Face region (FaceAIS)^2 select the highest facial injury for ISS calculation	Not Applicable
BestOfHeadBrainCervicalISS	TRUE	FALSE	integer	HeadBrainCervical region (Highest AIS of the region)^2 Compare HeadNeckAIS, InjuryHx.BrainInjuryAIS, CervicalSpineAIS select the highest scoring injury in any of these 3 areas for ISS calculation	Not Applicable
BrainInjuryAIS	TRUE	FALSE	integer	AIS score for the Brain Injury In the original AIS classification of injury severity, the grading is from 1 (minor) to 6 (unsurvivable). We added a score of 0 to designate absence of injuries.	0=None;1=Minor: no treatment needed;2=Moderate: requires only outpatient treatment;3=Serious: requires non-ICU hospital admission;4=Severe: requires ICU observation and/or basic treatment;5=Critical: requires intubation, mechanical ventilation or vasopressors for blood pressure support;6=Unsurvivable: not survivable
BrainInjuryDesc	TRUE	FALSE	text	Injury description related to the AIS/ISS score for the Brain Injury. Injury description is coded by drop-down menus for each body region	Not Applicable
CervicalSpineAIS	TRUE	FALSE	integer	AIS score for the Cervical Spine region. In the original AIS classification of injury severity, the grading is from 1 (minor) to 6 (unsurvivable). We added a score of 0 to designate absence of injuries.	0=None;1=Minor: no treatment needed;2=Moderate: requires only outpatient treatment;3=Serious: requires non-ICU hospital admission;4=Severe: requires ICU observation and/or basic treatment;5=Critical: requires intubation, mechanical ventilation or vasopressors for blood pressure support;6=Unsurvivable: not survivable
CervicalSpineDesc	TRUE	FALSE	text	Injury description related to the AIS/ISS score for the Cervical Spine region.	Not Applicable
DayInjury	TRUE	FALSE	text	Day of injury. (Sunday - Saturday)	0=Sunday;1=Monday;2=Tuesday;3=Wednesday;4=Thursday;5=Friday;6=Saturday
ExternaAIS	TRUE	FALSE	integer	AIS score for the External skin In the original AIS classification of injury severity, the grading is from 1 (minor) to 6 (unsurvivable). We added a score of 0 to designate absence of injuries.	0=None;1=Minor: no treatment needed;2=Moderate: requires only outpatient treatment;3=Serious: requires non-ICU hospital admission;4=Severe: requires ICU observation and/or basic treatment;5=Critical: requires intubation, mechanical ventilation or vasopressors for blood pressure support;6=Unsurvivable: not survivable

Name	Static	Intervention	Format	Description	Possible Values
ExternaDesc	TRUE	FALSE	text	Injury description related to the AIS/ISS score for the Externa (skin) region.	Not Applicable
FaceAIS	TRUE	FALSE	integer	AIS score for Face (incl.maxillofacial) In the original AIS classification of injury severity, the grading is from 1 (minor) to 6 (unsurvivable). We added a score of 0 to designate absence of injuries.	0=None;1=Minor: no treatment needed;2=Moderate: requires only outpatient treatment;3=Serious: requires non-ICU hospital admission;4=Severe: requires ICU observation and/or basic treatment;5=Critical: requires intubation, mechanical ventilation or vasopressors for blood pressure support;6=Unsurvivable: not survivable
FaceDesc	TRUE	FALSE	text	Injury description related to the AIS/ISS score for the Face (incl.maxillofacial) region	Not Applicable
HeadNeckAIS	TRUE	FALSE	integer	AIS score for the Head Neck region In the original AIS classification of injury severity, the grading is from 1 (minor) to 6 (unsurvivable). We added a score of 0 to designate absence of injuries.	0=None;1=Minor: no treatment needed;2=Moderate: requires only outpatient treatment;3=Serious: requires non-ICU hospital admission;4=Severe: requires ICU observation and/or basic treatment;5=Critical: requires intubation, mechanical ventilation or vasopressors for blood pressure support;6=Unsurvivable: not survivable
HeadNeckDesc	TRUE	FALSE	text	Injury description for the Head and Neck AIS.	Not Applicable
InjAIS	TRUE	FALSE	integer	In the original AIS classification of injury severity, the grading is from 1 (minor) to 6 (unsurvivable). We added a score of 0 to designate absence of injuries. This is the AIS score for body regions as specified by AIS.InjBodyRegion.	0=None;1=Minor: no treatment needed;2=Moderate: requires only outpatient treatment;3=Serious: requires non-ICU hospital admission;4=Severe: requires ICU observation and/or basic treatment;5=Critical: requires intubation, mechanical ventilation or vasopressors for blood pressure support;6=Unsurvivable: not survivable
InjArea	TRUE	FALSE	integer	Reflects the area where the injury took place (urban or rural).	1=Urban (city);2=Rural;88=Unknown
InjBodyRegion	TRUE	FALSE	integer	Injuries and their severity are recorded according to a (modification of) the AIS. The AIS recognizes 6 main body regions. We included a further subdivision for some body regions, resulting in a total of 12 regions. For example, spine is not considered separately in the original AIS classification, but included under neck/chest and abdomen regions. For TBI, however, we considered it important to record spine separately.	1=Externa;2=Head and Neck-Other;3=Brain Injury;4=Cervical Spine;5=Face;6=Thorax/Chest;7=Thoracic Spine;8=Abdomen/Pelvic Contents;9=Lumbar Spine;10=Upper Extremities;11=Lower Extremities;12=Pelvic Girdle
InjCause	TRUE	FALSE	integer	Reflects the cause of injury.	1=Road traffic incident;2=Incidental fall;3=Other non-intentional injury;4=Violence/assault;5=Act of mass violence;6=Suicide attempt;88=Unknown;99=Other
InjCauseOther	TRUE	FALSE	text	Reflects if the cause of injury was "other" than the pre-listed causes. See also InjuryHx.InjCause	Not Applicable
InjDescription	TRUE	FALSE	integer	List of body regions with 55 subcategories describing the injury.	1=Brain Injury: Concussion;2=Brain Injury: Contusions;3=Brain Injury: EDH;4=Brain Injury: Diffuse Injury;5=Brain Injury: ASDH;6=Brain Injury: Other;7=Head and Neck-Other: Specify in comments box;8=Cervical Spine: Fracture;9=Cervical Spine: Dislocation;10=Cervical Spine: Other;11=Face: Maxillo-facial fracture le Fort I;12=Face: Maxillo-facial fracture le Fort II;13=Face: Maxillo-facial fracture le Fort III;14=Face: Orbital fracture;15=Face: Zygomatic arch fracture;16=Face: Other;17=Thorax/Chest: Rib fracture;18=Thorax/Chest: Lung contusion;19=Thorax/Chest: Cardiac contusion;20=Thorax/Chest: Aorta dissection;21=Thorax/Chest: Pneumo-thorax;22=Thorax/Chest: Hemato-thorax;23=Thorax/Chest: Other;24=Thoracic Spine: Fracture;25=Thoracic Spine: Dislocation;26=Abdomen/Pelvic Contents: Spleen rupture;27=Abdomen/Pelvic Contents: Liver rupture;28=Abdomen/Pelvic Contents: Perforating abdominal injury;29=Abdomen/Pelvic Contents: Kidney contusion;30=Abdomen/Pelvic Contents: Retroperitoneal hematoma;31=Abdomen/Pelvic Contents: Other;32=Lumbar Spine: Fracture;33=Lumbar Spine: Dislocation;34=Lumbar Spine: Sacral fracture;35=Lumbar Spine: Other;36=Upper Extremities: Humerus fracture;37=Upper Extremities: Radial and/or ulnar fracture;38=Upper Extremities: Dislocation;39=Upper Extremities: Hand;40=Upper Extremities: Finger;41=Lower Extremities: Femoral fracture;42=Lower Extremities: Tibia plateau fracture;43=Lower Extremities: Tibia fracture;44=Lower Extremities: Ankle fracture;45=Lower Extremities: Calcaneus fracture;46=Lower Extremities: Metatarsal/tarsal fracture (toe fracture);47=Lower Extremities: Fibula fracture;48=Pelvic Girdle: Pelvic fracture;49=Pelvic Girdle: Hip fracture;50=Pelvic Girdle: Hip dislocation;51=Externa: Other;52=Thoracic Spine: Other;53=Upper

Name	Static	Intervention	Format	Description	Possible Values
					Extremities: Other;54=Lower Extremities: Other;55=Pelvic Girdle: Other
InjDesOther	TRUE	FALSE	text	Free text specifying the injury when AIS.InjDescription is "other"	Not Applicable
InjIndContactSportType	TRUE	FALSE	integer	Reflects the kind of contact sport involved as cause of injury - Only applicable for sports/recreational injuries	1=Boxing;2=Martial Arts;99=Other
InjIndSportTypeOther	TRUE	FALSE	text	Reflects if the kind of contact sport involved as cause of injury was "other" than the pre-defined list - Only applicable for sports/recreational injuries. See also InjuryHx.InjIndContactSportType	Not Applicable
InjIntention	TRUE	FALSE	integer	Reflects if the cause on injury was intentional or unintentional.	1=Intentional;2=Unintentional;3=Undetermined
InjMech1	TRUE	FALSE	integer	Reflects the mechanism of injury - only applicable for Closed TBI	1=High velocity trauma (acceleration/deceleration); 2=Direct impact: blow to head; 3=Direct impact: head against object; 6=Ground level fall; 7=Fall from height > 1 meter/5 stairs; 99=Other closed head injury;
InjMech2	TRUE	FALSE	integer	Reflects the mechanism of injury - only applicable for Closed TBI	1=High velocity trauma (acceleration/deceleration); 2=Direct impact: blow to head; 3=Direct impact: head against object; 6=Ground level fall; 7=Fall from height > 1 meter/5 stairs; 99=Other closed head injury;
InjMech3	TRUE	FALSE	integer	Reflects the mechanism of injury - only applicable for Closed TBI	1=High velocity trauma (acceleration/deceleration); 2=Direct impact: blow to head; 3=Direct impact: head against object; 6=Ground level fall; 7=Fall from height > 1 meter/5 stairs; 99=Other closed head injury;
InjMech6	TRUE	FALSE	integer	Reflects the mechanism of injury - only applicable for Closed TBI	1=High velocity trauma (acceleration/deceleration); 2=Direct impact: blow to head; 3=Direct impact: head against object; 6=Ground level fall; 7=Fall from height > 1 meter/5 stairs; 99=Other closed head injury;
InjMech7	TRUE	FALSE	integer	Reflects the mechanism of injury - only applicable for Closed TBI	1=High velocity trauma (acceleration/deceleration); 2=Direct impact: blow to head; 3=Direct impact: head against object; 6=Ground level fall; 7=Fall from height > 1 meter/5 stairs; 99=Other closed head injury;
InjMech99	TRUE	FALSE	integer	Reflects the mechanism of injury - only applicable for Closed TBI	1=High velocity trauma (acceleration/deceleration); 2=Direct impact: blow to head; 3=Direct impact: head against object; 6=Ground level fall; 7=Fall from height > 1 meter/5 stairs; 99=Other closed head injury;
InjMechOther	TRUE	FALSE	text	Reflects if the mechanism of injury was "other" than the pre-defined list - only applicable for Closed TBI. See also InjuryHx.InjMech	Not Applicable
InjOtherPartyInvolved	TRUE	FALSE	integer	Reflects that "another party involved in the cause of injury = N/A".	0=No;1=Yes
InjOtherPartySleepingPills	TRUE	FALSE	integer	Reflects if sedatives or sleeping pills were involved in the cause of injury.	0=No;1=Suspect;2=Definite;88=Unknown
InjPenetratingType	TRUE	FALSE	integer	Reflects the mechanism of injury - only applicable if Penetrating brain injury	1=Gunshot wound;2=Fragment (incl. shell/shrapnel);99=Other penetrating brain injury
InjPenetratingTypeOther	TRUE	FALSE	text	Reflects if the mechanism of injury was other than the pre-defined list - only applicable if Penetrating brain injury. See also InjuryHx.InjPenetratingType	Not Applicable
InjPlace	TRUE	FALSE	integer	Reflects the place where the TBI injury occurred.	1=Street/highway;2=Home/domestic;3=Work/school;4=Sport/Recreational;5=Military deployment;6=Public location (eg. bar, station;88=Unknown;99=Other
InjPlaceOther	TRUE	FALSE	text	Reflects if the place where the TBI injury occurred was "other" than the pre-defined list. See also InjuryHx.InjPlace	Not Applicable
InjRecSportType	TRUE	FALSE	integer	Reflects of the cause of injury was "Other Sport & Recreational Activities" - Only applicable for sports/recreational injuries	1=Rollerblading/Skateboarding/Scootering;2=Skiing;3=Snowboarding;4=Hiking/Climbing;5=Horseriding;6=Golf;7=Cycling;8=Off-road vehicular sports;9=Water sports;10=Playground activity;88=Unknown;99=Other
InjRecSportTypeOther	TRUE	FALSE	text	Describes which was the cause of injury if "Other Sport & Recreational Activities" - Only applicable for sports/recreational injuries	Not Applicable
InjRoadAccEjectedFromVehicle	TRUE	FALSE	integer	Reflects if the subject was ejected from the vehicle - Only applicable if subject was motor vehicle occupant	0=No;1=Yes;88=Unknown
InjRoadAccOtherParty	TRUE	FALSE	integer	Reflects if another party was involved in the Cause of Injury in case of a Road Traffic accident	1=Motor vehicle;2=Pedestrian;3=Cyclist;4=Moped/Scooter;5=Tram/Bus;6=Train/Metro;7=Obstacle;10=Motor Bike;11=Lorry (camion);88=Unknown;99=Other

Name	Static	Intervention	Format	Description	Possible Values
InjRoadAccOtherPartyInvolved	TRUE	FALSE	integer	Describes if another party than the pre-defined list was involved in the Cause of Injury in case of a Road Traffic accident	0=No;1=Yes;88=Unknown
InjRoadAccOtherPartyOther	TRUE	FALSE	text	Describes which other party than the pre-defined list was involved in the Cause of Injury in case of a Road Traffic accident	Not Applicable
InjRoadAccVictim	TRUE	FALSE	integer	Describes the type of victim in case of a Road traffic accident.	1=Motor vehicle occupant;2=Pedestrian;3=Cyclist;4=Moped/Scooter;5=Motor Bike;99=Other
InjRoadAccVictimOther	TRUE	FALSE	text	Reflects if the type of victim was "other" than the predefined list in case of a Road traffic accident.	Not Applicable
InjRoadAccVictimVehiclePlace	TRUE	FALSE	integer	Reflects the occupant placement of the victim in the vehicle in case of a Road Traffic Accident	1=Driver;2=Front seat passenger;3=Back seat passenger
InjSafetyAirbag	TRUE	FALSE	integer	Perfects if the airbag was deployed - Only applicable if subject was motor vehicle occupant	0=No;1=Yes;77=Not Applicable;88=Unknown
InjSafetyHelmet	TRUE	FALSE	integer	Reflects if the victim was wearing a safety helmet. Only applicable in case of cyclist, scooter, motorbike incident. However, may also have been scored for various sports injuries.	0=No;1=Yes;77=Not Applicable;88=Unknown
InjSafetySeatbelt	TRUE	FALSE	integer	Reflects if the victim was wearing a seat-belt. Only applicable if subject was motor vehicle occupant	0=No;1=Yes;77=Not Applicable;88=Unknown
InjTeamSportType	TRUE	FALSE	integer	Reflects the type of team sport that was the cause of the injury - Only applicable for sports/recreational injuries	1=Football (soccer);2=Rugby;3=Field Hockey;4=Ice Hockey;5=Lacrosse;99=Other
InjTeamSportTypeOther	TRUE	FALSE	text	Reflects if the type of team sport that was the cause of the injury was "other" than the predefined list- Only applicable for sports/recreational injuries	Not Applicable
InjToICUAdmHours	TRUE	FALSE	decimal	Time between injury and admission to ICU	1=High velocity trauma (acceleration/deceleration); 2=Direct impact: blow to head; 3=Direct impact: head against object; 6=Ground level fall; 7=Fall from height > 1 meter/5 stairs; 99=Other closed head injury;
InjType	TRUE	FALSE	integer	Details of Injury are captured in 3 different variables: Type of Injury, Place of Injury and Mechanism of injury. This reflects the type of injury.	1=Closed;2=Blast;3=Crush;5=Penetrating;6=Penetrating-perforating;7=Penetrating-tangential;8=Closed with open depressed skull fracture;88=Unknown
InjVictimAlcoholTestType	TRUE	FALSE	text	Reflects type of alcohol test used (breath test or blood test) for the victim	Blood=Blood Test;Breath=Breath Test
InjVictimBloodAlcoholmgdL	TRUE	FALSE	decimal	Reflects the level of mg/dL alcohol recorded in the victim during the alcohol test in case alcohol was related to the cause of injury.	Not Applicable
InjVictimBloodAlcoholpermil	TRUE	FALSE	decimal	Reflects the level of alcohol per mil (0/00) recorded in the victim during the alcohol test in case alcohol was related to the cause of injury.	Not Applicable
InjVictimDrugsTypeOther	TRUE	FALSE	text	Describes which other drugs where involved for the victim in the cause of injury.	Not Applicable
InjVictimSleepingPills	TRUE	FALSE	integer	Reflects if for the victim use of sedatives of sleeping pills were involved in the cause of injury.	0=No;1=Suspect;2=Definite;88=Unknown
InjVictimTypeDrugs	TRUE	FALSE	integer	Rf elects which kind of drugs were involved in the cause of injury at the victims site.	1=Cannabis;2=Cocaine;3=Methamphetamine's;4=Opioids;5=XTC;88=Unknown;99=Other
InjViolence	TRUE	FALSE	integer	Reflects the type of violence used as cause of injury - Only applicable if violence was the cause of injury	1=Robbery;2=Interpersonal violence (fight);3=Domestic assault;4=Child abuse;5=Gang violence;6=Military deployment;88=Unknown;99=Other
InjViolenceOther	TRUE	FALSE	text	Reflects the "other" type of violence than the predefined list used as cause of injury - Only applicable if violence was the cause of injury	Not Applicable
InjViolenceOtherPartyAlcohol	TRUE	FALSE	integer	Information on drug and alcohol abuse of possible influence on the incident is different for victim versus "other party" (if involved) This reflects if alcohol was involved in the cause of injury for the other party involved	0=No;1=Definite;2=Suspect;88=Unknown
InjViolenceOtherPartyDrugs	TRUE	FALSE	integer	Information on drug and alcohol abuse of possible influence on the incident is different for victim versus "other party" (if	0=No;1=Definite;2=Suspect;88=Unknown

Name	Static	Intervention	Format	Description	Possible Values
				involved). This reflects if drugs was involved as cause of injury for the other party involved	
InjViolenceVictimAlcohol	TRUE	FALSE	integer	Information on drug and alcohol abuse of possible influence on the incident is different for victim versus "other party" (if involved) This reflects alcohol involvement for the victim.	0=No;1=Definite;2=Suspect;88=Unknown
InjViolenceVictimDrugs	TRUE	FALSE	integer	Information on drug and alcohol abuse of possible influence on the incident is different for victim versus "other party" (if involved) This reflects drugs involvement for the victim.	0=No;1=Definite;2=Suspect;88=Unknown
LowerExtremitiesAIS	TRUE	FALSE	integer	AIS score for the Lower extremities. In the original AIS classification of injury severity, the grading is from 1 (minor) to 6 (unsurvivable). We added a score of 0 to designate absence of injuries.	0=None;1=Minor: no treatment needed;2=Moderate: requires only outpatient treatment;3=Serious: requires non-ICU hospital admission;4=Severe: requires ICU observation and/or basic treatment;5=Critical: requires intubation, mechanical ventilation or vasopressors for blood pressure support;6=Unsurvivable: not survivable
LowerExtremitiesDesc	TRUE	FALSE	text	Injury Description for the AIS score for Lower extremities as subdomain of Extremities and pelvic girdle.	Not Applicable
LumbarSpineAIS	TRUE	FALSE	integer	AIS score for the Lumbar Spine region. In the original AIS classification of injury severity, the grading is from 1 (minor) to 6 (unsurvivable). We added a score of 0 to designate absence of injuries.	0=None;1=Minor: no treatment needed;2=Moderate: requires only outpatient treatment;3=Serious: requires non-ICU hospital admission;4=Severe: requires ICU observation and/or basic treatment;5=Critical: requires intubation, mechanical ventilation or vasopressors for blood pressure support;6=Unsurvivable: not survivable
LumbarSpineDesc	TRUE	FALSE	text	Injury description for AIS score for Lumbar spine as subdomain of Abdomen/Pelvic contents.	Not Applicable
MonthInjury	TRUE	FALSE	integer	Month of Injury (January - December)	1=January;2=February;3=March;4=April;5=May;6=June;7=July;8=August;9=September;10=October;11=November;12=December
PelvicGirdleAIS	TRUE	FALSE	integer	AIS score for the Pelvic Girdle region. In the original AIS classification of injury severity, the grading is from 1 (minor) to 6 (unsurvivable). We added a score of 0 to designate absence of injuries.	0=None;1=Minor: no treatment needed;2=Moderate: requires only outpatient treatment;3=Serious: requires non-ICU hospital admission;4=Severe: requires ICU observation and/or basic treatment;5=Critical: requires intubation, mechanical ventilation or vasopressors for blood pressure support;6=Unsurvivable: not survivable
PelvicGirdleDesc	TRUE	FALSE	text	Injury description for AIS score for Pelvic Girdle as subdomain of Extremities and pelvic girdle.	Not Applicable
ThoracicSpineAIS	TRUE	FALSE	integer	AIS score for the Thoracic spine Region In the original AIS classification of injury severity, the grading is from 1 (minor) to 6 (unsurvivable). We added a score of 0 to designate absence of injuries.	0=None;1=Minor: no treatment needed;2=Moderate: requires only outpatient treatment;3=Serious: requires non-ICU hospital admission;4=Severe: requires ICU observation and/or basic treatment;5=Critical: requires intubation, mechanical ventilation or vasopressors for blood pressure support;6=Unsurvivable: not survivable
ThoracicSpineDesc	TRUE	FALSE	text	Injury description for the AIS of Thoracic spine as subdomain of Thorax/Chest	Not Applicable
ThoraxChestAIS	TRUE	FALSE	integer	AIS score for the Thorax Chest region. In the original AIS classification of injury severity, the grading is from 1 (minor) to 6 (unsurvivable). We added a score of 0 to designate absence of injuries.	0=None;1=Minor: no treatment needed;2=Moderate: requires only outpatient treatment;3=Serious: requires non-ICU hospital admission;4=Severe: requires ICU observation and/or basic treatment;5=Critical: requires intubation, mechanical ventilation or vasopressors for blood pressure support;6=Unsurvivable: not survivable
ThoraxChestDesc	TRUE	FALSE	text	Injury description for the AIS of the Thorax/Chest region.	Not Applicable
TotalISS	TRUE	FALSE	integer	The Injury Severity Score is calculated as the sum of the squares of the the 3 body regions with the highest AIS score. The max score for the ISS = 75. If any body region AIS is assigned a score of "6", the ISS is automatically set to 75 (highest score). In the calculation of the ISS, only the 6 main body regions are taken into consideration.	Not Applicable
UpperExtremitiesAIS	TRUE	FALSE	integer	AIS score for the Upper extremities. In the original AIS classification of injury severity, the grading is from 1 (minor) to 6 (unsurvivable). We added a score of 0 to designate absence of injuries.	0=None;1=Minor: no treatment needed;2=Moderate: requires only outpatient treatment;3=Serious: requires non-ICU hospital admission;4=Severe: requires ICU observation and/or basic treatment;5=Critical: requires intubation, mechanical ventilation or vasopressors for blood pressure support;6=Unsurvivable: not survivable

Name	Static	Intervention	Format	Description	Possible Values
UpperExtremitiesDesc	TRUE	FALSE	text	Injury description for the AIS score of the Upper extremities as subdomain of Extremities and pelvic girdle.	Not Applicable

Emergency care and ICU admission

Name	Static	Intervention	Format	Description	Possible Values
ACEFocalNeuroDeficit	TRUE	FALSE	integer	On the neurological assessment at presentation overall rating was recorded on Focal neurological deficit (eg paresis or dysphasia)	0=No;1=Yes;88=Unknown
ACEFocalNeuroDeficitDysphasia	TRUE	FALSE	integer	On the neurological assessment at presentation overall rating was recorded on Focal neurological deficit (eg paresis or dysphasia). If this was rate as "yes", details were recorded on whether it was paresis or dysphasia.	0=No;1=Yes;88=Unknown
ACEFocalNeuroDeficitOther	TRUE	FALSE	integer	On the neurological assessment at presentation overall rating was recorded on Focal neurological deficit (eg paresis or dysphasia) If this was rate as "yes", details were recorded on whether it was paresis or dysphasia or other.	0=No;1=Yes
ACEFocalNeuroDeficitOtherTxt	TRUE	FALSE	text	On the neurological assessment at presentation overall rating was recorded on Focal neurological deficit (eg paresis or dysphasia) If this was rate as "yes", details were recorded on whether it was paresis or dysphasia or other, and for other it was specified which one.	Not Applicable
ACEFocalNeuroDeficitParesis	TRUE	FALSE	integer	On the neurological assessment at presentation overall rating was recorded on Focal neurological deficit (eg paresis or dysphasia) If this was rate as "yes", details were recorded on whether it was paresis or dysphasia.	0=No;1=Yes;88=Unknown
ACEOverallRating	TRUE	FALSE	integer	On the neurological assessment at presentation overall rating was recorded on "How different the person is acting compared to his/her usual self" The rating was on a scale from 1 (normal) to 6 (very different).	1=1;2=2;3=3;4=4;5=5;6=6
ACERatedBy	TRUE	FALSE	integer	On the neurological assessment at presentation overall rating was recorded on "How different the person is acting compared to his/her usual self" This variable reflects by whom the rating was performed.	1=Proxy;2=Subject;3=Both proxy and subject;4=Not done
BaselineGCSMostReliableAssessmentCondition	TRUE	FALSE	integer	A baseline risk assessment was performed at the hospital (ER). This reflects the Conditions of assessment for the Most reliable Motor Score for risk assessment.	0=No sedation/paralysis;1=Under sedation;3=After stopping sedation;4=After pharmacological reversal
BaselineGCSMostReliableAssessmentTime	TRUE	FALSE	integer	A baseline risk assessment was performed at the hospital (ER). This reflects the Time of assessment for the Most reliable Motor Score for risk assessment.	1=Admission;2=Post-stabilization;3=First hospital;4=Scene of accident;5=Other
BaselineGCSMostReliableMotorScore	TRUE	FALSE	text	A baseline risk assessment was performed at the hospital (ER). This reflects the Most reliable baseline Motor score of the GCS as given by sites - for use in prognostic models.	1=1-None;2=2-Abnormal extension;3=3-Abnormal flexion;4=4-Normal flexion/withdrawal;5=5-Localizes to pain;6=6-Obeys command;O=Untestable (Other);P=Untestable (Deep sedation/paralyzed);UN=Unknown
BaselineGOS6MoExpectedDeathRisk	TRUE	FALSE	text	At ER discharge, physician estimate of six month outcome was recorded as a baseline risk assessment: "Given all current available information, what is, in your subjective opinion, the most likely 6-month outcome of this patient? To be based upon information on discharge ER or admission to hospital/ICU". This reflects the Risk of death in %	Not Applicable
BaselineGOS6MoExpectedOutcome	TRUE	FALSE	text	At ER discharge, physician estimate of six month outcome was recorded as a baseline risk assessment: "Given all current available information, what is, in your subjective opinion, the most likely 6-month outcome of	D=D - Death;GR=GR - Good Recovery;MD=MD - Moderate Disability;SD=SD - Severe Disability;V=V - Vegetative State

Name	Static	Intervention	Format	Description	Possible Values
				this patient? To be based upon information on discharge ER or admission to hospital/ICU". This reflects the Expected outcome (GOS)	
BaselineGOS6MoUnfavourableOutcomeRisk	TRUE	FALSE	text	At ER discharge, physician estimate of six month outcome was recorded as a baseline risk assessment: "Given all current available information, what is, in your subjective opinion, the most likely 6-month outcome of this patient? To be based upon information on discharge ER or admission to hospital/ICU". This reflects the Risk of unfavorable outcome (D, VS, SD) in %	Not Applicable
BaselinePhysEstOf6MoOutcomePhysicianQual	TRUE	FALSE	integer	At ER discharge, physician estimate of six month outcome was recorded as a baseline risk assessment: "Given all current available information, what is, in your subjective opinion, the most likely 6-month outcome of this patient? To be based upon information on discharge ER or admission to hospital/ICU". This reflects the qualification of the physician who provided prognostic estimate on ER discharge/admission to hospital/ICU	1=Resident;2=Junior staff (< 5 years);3=Senior staff (>= 5 years);4=Head of department
BaselinePhysEstOf6MoOutcomePhysicianType	TRUE	FALSE	integer	At ER discharge, physician estimate of six month outcome was recorded as a baseline risk assessment: "Given all current available information, what is, in your subjective opinion, the most likely 6-month outcome of this patient? To be based upon information on discharge ER or admission to hospital/ICU". This reflects the type of the physician who provided prognostic estimate on ER discharge/admission to hospital/ICU	1=ER Physician;2=Intensive Care;3=Neurology;4=Neurosurgery;5=Traumatology;88=Unknown
DispER	TRUE	FALSE	integer	Destination of the patient at ER discharge.	1=Discharge home;2=Discharge other facility;3=Hospital admission--Ward;4=Hospital admission--Intermediate/high care unit;5=Hospital admission--ICU;6=Hospital admission--OR for immediate surgical procedure;7=Death;8=Hospital admission--Other (e.g. observation unit);88=Unknown
EDAirway	TRUE	FALSE	integer	Records treatment performed in the ER/on admission with regard to Airways. Pre-hospital inteventions are documented at: InjuryHx.PresEmergencyCare	1=No specific treatment;2=Supplemental oxygen (via nasal tube or mask);3=Adjunctive airway (eg. Mayo tube);4=Temporary support with bag, valve;5=Intubation;6=Mechanical ventilation;88=Unknown
EDArrDBP	TRUE	FALSE	integer	Reflects Clinical Exam (on arrival at Study Center) --> BP (mmHg) --> Diastolic	Not Applicable
EDArrHR	TRUE	FALSE	integer	Reflects the vital signs at ER arrival: Heart rate --> Beats per min.	Not Applicable
EDArrivalAirway	TRUE	FALSE	integer	The ABC status on arrival documents the status of Airway, Breathing and Circulation upon arrival to Study Hospital (ER); In addition, administration of supplemental oxygen and spinal immobilization is documented.	1=Clear;2=Obstructed;3=Adjunctive Airway;4=Intubated;88=Unknown
EDArrivalArtpCO2mmhg	TRUE	FALSE	decimal	Reflects the vital signs at ER arrival: Arterial pCO2 --> mmHg	Not Applicable
EDArrivalArtpO2mmhg	TRUE	FALSE	decimal	Reflects the vital signs at ER arrival: Arterial pO2 --> mmHg	Not Applicable
EDArrivalBaseExcess	TRUE	FALSE	decimal	Reflects the vital signs at ER arrival: Base excess --> mEq/l	Not Applicable
EDArrivalBMIKgCm	TRUE	FALSE	decimal	BMI using height in cm (EDArrivalHeightCm) and weight (EDArrivalBodyWeightKg) BMI= (Weight/(Height*Height))) * 10000	Not Applicable
EDArrivalBodyWeightKg	TRUE	FALSE	decimal	Body weight in KG at ER arrival	Not Applicable
EDArrivalBodyWeightMeasured	TRUE	FALSE	integer	Provides an indication of accuracy of reported body weight at ER arrival	1=Estimated;2=Self reported;3=Measured;4=Proxy reported;88=Unknown
EDArrivalBreathing	TRUE	FALSE	integer	The ABC status on arrival documents the status of Airway, Breathing and Circulation upon arrival to Study Hospital (ER); In addition, administration of supplemental oxygen and spinal immobilization is documented.	1=Spontaneous, adequate;2=Spontaneous, insufficient;3=Manual support with bag, valve;4=Mechanical ventilation;88=Unknown

Name	Static	Intervention	Format	Description	Possible Values
EDArrivalCirculation	TRUE	FALSE	integer	The ABC status on arrival documents the status of Airway, Breathing and Circulation upon arrival to Study Hospital (ER); In addition, administration of supplemental oxygen and spinal immobilization is documented.	0=No specific therapy;1=IV Fluids;2=Vasopressors;3=CPR;88=Unknown
EDArrivalFiO2	TRUE	FALSE	integer	Reflects the vital signs at ER arrival: FiO2 (in %) Information on FiO2 (Fraction of Inspired oxygen) at time of arterial blood gas sampling is requested in order to be able to calculate PaO2/FiO2 as measure of severity of hypoxaemia; dependent on altitude; at sea-level, normal values are > 500 mmHg	Not Applicable
EDArrivalHeightCm	TRUE	FALSE	decimal	Reflects Height in cm at ER arrival	Not Applicable
EDArrivalHeightMeasured	TRUE	FALSE	integer	Provides an indication of accuracy of reported height at ER arrival	1=Estimated;2=Self reported;3=Measured;4=Proxy reported;88=Unknown
EDArrivalLactate	TRUE	FALSE	decimal	Reflects the vital signs at ER arrival: Lactate --> mEq/l	Not Applicable
EDArrivalpH	TRUE	FALSE	decimal	Reflects the vital signs at ER arrival: pH	Not Applicable
EDArrivalSpinalImmob	TRUE	FALSE	integer	The ABC status on arrival documents the status of Airway, Breathing and Circulation upon arrival to Study Hospital (ER); In addition, administration of supplemental oxygen and spinal immobilization is documented.	0=No;1=Yes;88=Unknown
EDArrivalSupplementalOxygen	TRUE	FALSE	integer	The ABC status on arrival documents the status of Airway, Breathing and Circulation upon arrival to Study Hospital (ER); In addition, administration of supplemental oxygen and spinal immobilization is documented.	0=No;1=Yes;88=Unknown
EDArrPupilLftEyeMeasr	TRUE	FALSE	integer	Reflects Clinical Exam (on arrival at Study Center) --> Left eye Size	Not Applicable
EDArrPupilReactivityLghtLftEyeReslt	TRUE	FALSE	integer	Neurological assessment (GCS and pupils) was recorded for the scene of accident, the first hospital (if applicable), the Arrival to ER of the study hospital and post-stabilization. This reflects the reactivity of the left eye pupil for the assessment at Arrival to ER of the study hospital.	1=+ (Brisk);2=+ (Sluggish);3=- (Negative)
EDArrPupilReactivityLghtRtEyeReslt	TRUE	FALSE	integer	Neurological assessment (GCS and pupils) was recorded for the scene of accident, the first hospital (if applicable), the Arrival to ER of the study hospital and post-stabilization. This reflects the reactivity of the right eye pupil for the assessment at Arrival to ER of the study hospital.	1=+ (Brisk);2=+ (Sluggish);3=- (Negative)
EDArrPupilRtEyeMeasr	TRUE	FALSE	integer	Reflects Clinical Exam (on arrival at Study Center) --> Right eye size	Not Applicable
EDArrPupilSymmetry	TRUE	FALSE	integer	Neurological assessment (GCS and pupils) was recorded for the scene of accident, the first hospital (if applicable), the Arrival to ER of the study hospital and post-stabilization. This reflects the Pupil symmetry for the assessment at Arrival to ER of the study hospital.	1=Equal;2=Unequal R>L;3=Unequal L>R;66=Untestable;88=Unknown
EDArrRespRate	TRUE	FALSE	integer	Reflects the vital signs at ER arrival: Respiratory rate --> cycles per min	Not Applicable
EDArrSBP	TRUE	FALSE	integer	Reflects Clinical Exam (on arrival at Study Center) --> BP (mmHg) --> Systolic	Not Applicable
EDArrSpO2	TRUE	FALSE	decimal	Reflects Clinical Exam (on arrival at Study Center) --> Oxygen saturation (in %)	Not Applicable
EDArrTempCelsius	TRUE	FALSE	decimal	Reflects the vital signs at ER arrival: Temperature --> Celcius	Not Applicable
EDBloodGasConditions	TRUE	FALSE	integer	Reflects the vital signs at ER arrival: Conditions for First arterial blood gas done (if applicable)	1=Pre-intubation, room air;2=Pre-intubation, +O2;3=Post-intubation, not ventilated;4=Post-intubation, ventilated
EDBloodTrans	TRUE	FALSE	integer	Reflects of blood transfusion was done in the ER of the study hospital	0=No;1=Yes;88=Unknown
EDCircCPR	TRUE	FALSE	integer	Records treatment performed in the ER/on admission with regard to Circulation: CPR. Pre-hospital inteventions are documented at: InjuryHx.PresEmergencyCare	0=No;1=Yes

Name	Static	Intervention	Format	Description	Possible Values
EDCircIV	TRUE	FALSE	integer	Records treatment performed in the ER/on admission with regard to Circulation: IV fluids. Pre-hospital inteventions are documented at: InjuryHx.PresEmergencyCare	0=No;1=Yes
EDCircNone	TRUE	FALSE	integer	Records treatment performed in the ER/on admission with regard to Circulation: no specific treatment Pre-hospital inteventions are documented at: InjuryHx.PresCirculationTreatmentNone	0=No;1=Yes
EDCircVaso	TRUE	FALSE	integer	Records treatment performed in the ER/on admission with regard to Circulation: vasopressors. Pre-hospital inteventions are documented at: InjuryHx.PresEmergencyCare	0=No;1=Yes
EDCoagulopathyType1	TRUE	FALSE	integer	Reflects the type of transfusion or coagulopathy treatment given in the ER of the study hospital (if applicable)	1=Packed red blood cell concentrates (pRBCs);2=Fresh whole blood;3=Fresh frozen plasma (FFP);4=Freeze dried plasma / lypholized plasma;5=Platelet concentrates;6=PCC (prothrombin complex concentrates);7=Fibrinogen concentrate;8=Albumin;9=Recombinant factor FVIIa;10=Tranexamic acid (TXA);11=Cryoprecipitate;12=Desmopression (DDAVP);13=Factor XIII;14=Calcium;15=Vitamin K (Konakion)
EDCoagulopathyType2	TRUE	FALSE	integer	Reflects the type of transfusion or coagulopathy treatment given in the ER of the study hospital (if applicable)	1=Packed red blood cell concentrates (pRBCs);2=Fresh whole blood;3=Fresh frozen plasma (FFP);4=Freeze dried plasma / lypholized plasma;5=Platelet concentrates;6=PCC (prothrombin complex concentrates);7=Fibrinogen concentrate;8=Albumin;9=Recombinant factor FVIIa;10=Tranexamic acid (TXA);11=Cryoprecipitate;12=Desmopression (DDAVP);13=Factor XIII;14=Calcium;15=Vitamin K (Konakion)
EDCoagulopathyType3	TRUE	FALSE	integer	Reflects the type of transfusion or coagulopathy treatment given in the ER of the study hospital (if applicable)	1=Packed red blood cell concentrates (pRBCs);2=Fresh whole blood;3=Fresh frozen plasma (FFP);4=Freeze dried plasma / lypholized plasma;5=Platelet concentrates;6=PCC (prothrombin complex concentrates);7=Fibrinogen concentrate;8=Albumin;9=Recombinant factor FVIIa;10=Tranexamic acid (TXA);11=Cryoprecipitate;12=Desmopression (DDAVP);13=Factor XIII;14=Calcium;15=Vitamin K (Konakion)
EDCoagulopathyType4	TRUE	FALSE	integer	Reflects the type of transfusion or coagulopathy treatment given in the ER of the study hospital (if applicable)	1=Packed red blood cell concentrates (pRBCs);2=Fresh whole blood;3=Fresh frozen plasma (FFP);4=Freeze dried plasma / lypholized plasma;5=Platelet concentrates;6=PCC (prothrombin complex concentrates);7=Fibrinogen concentrate;8=Albumin;9=Recombinant factor FVIIa;10=Tranexamic acid (TXA);11=Cryoprecipitate;12=Desmopression (DDAVP);13=Factor XIII;14=Calcium;15=Vitamin K (Konakion)
EDCoagulopathyVolume1	TRUE	FALSE	text	Reflects the volume of transfusion or coagulopathy treatment given in the ER of the study hospital (if applicable)	Not Applicable
EDCoagulopathyVolume2	TRUE	FALSE	text	Reflects the volume of transfusion or coagulopathy treatment given in the ER of the study hospital (if applicable)	Not Applicable
EDCoagulopathyVolume3	TRUE	FALSE	text	Reflects the volume of transfusion or coagulopathy treatment given in the ER of the study hospital (if applicable)	Not Applicable
EDCoagulopathyVolume4	TRUE	FALSE	text	Reflects the volume of transfusion or coagulopathy treatment given in the ER of the study hospital (if applicable)	Not Applicable
EDCompEventHypothermia	TRUE	FALSE	integer	Second Insults reported here relate to the pre-hospital and ER phase. Hypothermia is defined as a documented core temperature of < 35 C.	0=No;1=Definite;2=Suspect;88=Unknown
EDComplEventCardArr	TRUE	FALSE	integer	Second Insults reported here relate to the pre-hospital and ER phase: Cardiac Arrest	0=No;1=Yes
EDComplEventHypotension	TRUE	FALSE	integer	Second Insults reported here relate to the pre-hospital and ER phase. Definite hypotension is defined as a documented systolic BP < 90 mm Hg (adults); "Suspected" was scored if the patient did not have a documented blood pressure, but was reported to be in shock or have an absent brachial pulse (not related to injury of the extremity)	0=No;1=Definite;2=Suspect;88=Unknown
EDComplEventHypoxia	TRUE	FALSE	integer	Second Insults reported here relate to the pre-hospital and ER phase. Definite hypoxia is defined as a documented PaO2 <8 kPa (60 mm Hg) and/or SaO2<90%; "Suspected"	0=No;1=Definite;2=Suspect;88=Unknown

Name	Static	Intervention	Format	Description	Possible Values
				was scored if the patient did not have documented hypoxia by PaO2 or SaO2, but there was a clinical suspicion , as evidenced by for example cyanosis, apnoea or respiratory distress	
EDComplEventSeizures	TRUE	FALSE	integer	Second Insults reported here relate to the pre-hospital and ER phase: seizures	0=No;1=Partial/Focal;2=Generalized;3=Status epilepticus;88=Unknown
EDCorrCoagulopathy	TRUE	FALSE	integer	Documents blood transfusions and treatment of coagulopathy in the acute phase at presentation.	0=No;1=Yes;88=Unknown
EDDischPupilLftEyeMeasr	TRUE	FALSE	integer	Neurological assessment (GCS and pupils) was recorded for the scene of accident, the first hospital (if applicable), the Arrival to ER of the study hospital and post-stabilization. This reflects the size of the left eye pupil for the assessment POST¬≠-STABILIZATION.	Not Applicable
EDDischPupilReactivityLghtLftEyeReslt	TRUE	FALSE	integer	Neurological assessment (GCS and pupils) was recorded for the scene of accident, the first hospital (if applicable), the Arrival to ER of the study hospital and post-stabilization. This reflects the reactivity of the left eye pupil for the assessment POST¬≠-STABILIZATION.	1=+ (Brisk);2=+ (Sluggish);3=- (Negative)
EDDischPupilReactivityLghtRtEyeReslt	TRUE	FALSE	integer	Neurological assessment (GCS and pupils) was recorded for the scene of accident, the first hospital (if applicable), the Arrival to ER of the study hospital and post-stabilization. This reflects the reactivity of the right eye pupil for the assessment POST¬≠-STABILIZATION.	1=+ (Brisk);2=+ (Sluggish);3=- (Negative)
EDDischPupilRtEyeMeasr	TRUE	FALSE	integer	Neurological assessment (GCS and pupils) was recorded for the scene of accident, the first hospital (if applicable), the Arrival to ER of the study hospital and post-stabilization. This reflects the size of the right eye pupil for the assessment POST¬≠-STABILIZATION.	Not Applicable
EDDischPupilSymmetry	TRUE	FALSE	integer	Neurological assessment (GCS and pupils) was recorded for the scene of accident, the first hospital (if applicable), the Arrival to ER of the study hospital and post-stabilization. This reflects pupil symmetry for the assessment POST¬≠-STABILIZATION.	1=Equal;2=Unequal R>L;3=Unequal L>R;66=Untestable;88=Unknown
EDICPMonitoring	TRUE	FALSE	integer	Scheduled for ICP monitoring; e.g. may not accurately reflect if ICP monitoring was indeed performed	0=No;1=Yes;88=Unknown
EDIVAlbumin	TRUE	FALSE	integer	Records treatment performed in the ER/on admission with regard to Circulation: IV fluids --> Albumin	0=No;1=Yes
EDIVBlood	TRUE	FALSE	integer	Records treatment performed in the ER/on admission with regard to Circulation: IV fluids --> Blood	0=No;1=Yes
EDIVColloids	TRUE	FALSE	integer	Records treatment performed in the ER/on admission with regard to Circulation: IV fluids --> Colloids	0=No;1=Yes
EDIVCrystalloids	TRUE	FALSE	integer	Records treatment performed in the ER/on admission with regard to Circulation: IV fluids --> Crystalloids	0=No;1=Yes
EDIVMannitol	TRUE	FALSE	integer	Records treatment performed in the ER/on admission with regard to Circulation: IV fluids --> Mannitol	0=No;1=Yes
EDIVSaline	TRUE	FALSE	integer	Records treatment performed in the ER/on admission with regard to Circulation: IV fluids --> Hypertonic saline	0=No;1=Yes
EDSecondInsultsNeuroWorse	TRUE	FALSE	integer	The importance of Neuroworsening was first described by Morris and Marshall. The occurrence of neuroworsening is related to poorer outcome in subjects with moderate to severe TBI. Neuroworsening is defined as 1) a decrease in GCS motor score of 2 or more points; 2) a new loss of pupillary reactivity or development of pupillary assymmetry >=	0=No;1=Yes;88=Unknown

Name	Static	Intervention	Format	Description	Possible Values
				2mm; 3) deterioration in neurological or CT status sufficient to warrant immediate medical or surgical intervention	
EDSecondInsultsNeuroWorseYes1	TRUE	FALSE	integer	This variable provides a specification of the type of neuroworsening if it occurs.	1=Decrease in motor score >= 2 points; 2=Development of pupillary abnormalities; 3=Other neurological and/or CT deterioration
EDSecondInsultsNeuroWorseYes2	TRUE	FALSE	integer	This variable provides a specification of the type of neuroworsening if it occurs.	1=Decrease in motor score >= 2 points; 2=Development of pupillary abnormalities; 3=Other neurological and/or CT deterioration
EDSecondInsultsNeuroWorseYes3	TRUE	FALSE	integer	This variable provides a specification of the type of neuroworsening if it occurs.	1=Decrease in motor score >= 2 points; 2=Development of pupillary abnormalities; 3=Other neurological and/or CT deterioration
EDSecondInsultsPreAdmisCourse	TRUE	FALSE	integer	The pre-admission course should only be considered an intracranial second insult in case of Deterioration. The nature of deterioration will in most cases be further detailed under the variable "Neuroworsening".	0=Deterioration;1=Stable;2=Improving;88=Unknown
EDSpinalImmob	TRUE	FALSE	integer	Records treatment performed in the ER/on admission with regard to Spinal immobilization. Pre-hospital inteventions are documented at: InjuryHx.PresEmergencyCare	0=No;1=Yes;88=Unknown
EmergSurgInterventionsExtraCran	TRUE	FALSE	integer	Documents emergency extracranial surgery performed in study hospital. Procedures performed in "first" hospital (in case of secondary referral) are documented at: InjuryHx.PresERextracranialSurg	0=No;1=Yes;88=Unknown
EmergSurgInterventionsExtraCranYes	TRUE	FALSE	integer	Documents emergency extracranial surgery performed in study hospital. Procedures performed in "first" hospital (in case of secondary referral) are documented at: InjuryHx.PresERextracranialSurg	1=Damage control thoracotomy;2=Damage control laparotomy;3=Extraperitoneal pelvic packing;4=External fixation limb;5=Cranio-maxillo-facial reconstruction;99=Other
EmergSurgInterventionsExtraCranYesOther	TRUE	FALSE	text	Documents emergency extracranial surgery performed in study hospital. Procedures performed in "first" hospital (in case of secondary referral) are documented at: InjuryHx.PresERextracranialSurg	Not Applicable
EmergSurgInterventionsIntraCran	TRUE	FALSE	integer	Documents emergency intracranial surgery performed in study hospital. Procedures performed in "first" hospital (in case of secondary referral) are documented at: InjuryHx.PresERIntracranialSurg	0=No;1=Yes;88=Unknown
EmergSurgInterventionsIntraCranYes	TRUE	FALSE	integer	Documents emergency intracranial surgery performed in study hospital. Procedures performed in "first" hospital (in case of secondary referral) are documented at: InjuryHx.PresERIntracranialSurg	1=Craniotomy for haematoma/contusion;2=Decompressive Craniectomy;3=Depressed skull fracture;99=Other intracranial procedure
EmerSurgIntraCranSurviveNoSurg	TRUE	FALSE	integer	"InjuryHx.EmerSurgIntraCranSurviveNoSurg" and "InjuryHx.EmerSurgIntraCranSurviveYesSurg" These 2 variables aim to capture information on the surgeon's expectations, eg if the surgeon considers a realistic expectation of benefit, or performs the surgery as a "last resort" in a likely hopeless case. 'The short term survival chances of the patients if I DO NOT operate will be (in %)'	Not Applicable
EmerSurgIntraCranSurviveYesSurg	TRUE	FALSE	integer	"InjuryHx.EmerSurgIntraCranSurviveNoSurg" and "InjuryHx.EmerSurgIntraCranSurviveYesSurg" These 2 variables aim to capture information on the surgeon's expectations, eg if the surgeon considers a realistic expectation of benefit, or performs the surgery as a "last resort" in a likely hopeless case. 'The short term survival chances of the patients if I DO operate will be (in %)'	Not Applicable
ERDestICDCodes1	TRUE	FALSE	text	Up to 16 fields available to enter diagnosis as recorded by hospital administration according to ICD codes; applicable to patients discharged directly from the ER. For patients admitted to hospital or ICU, ICD codes are documented in: Hospital.ICDCode1 and Hospital.ICUDischargeICDCode1	Not Applicable

Name	Static	Intervention	Format	Description	Possible Values
ERDestICDCodes2	TRUE	FALSE	text	Up to 16 fields available to enter diagnosis as recorded by hospital administration according to ICD codes; applicable to patients discharged directly from the ER. For patients admitted to hospital or ICU, ICD codes are documented in: Hospital.ICDCode1 and Hospital.ICUDischargeICDCode1	Not Applicable
ERDestICDCodes3	TRUE	FALSE	text	Up to 16 fields available to enter diagnosis as recorded by hospital administration according to ICD codes; applicable to patients discharged directly from the ER. For patients admitted to hospital or ICU, ICD codes are documented in: Hospital.ICDCode1 and Hospital.ICUDischargeICDCode1	Not Applicable
ERDestICDCodes4	TRUE	FALSE	text	Up to 16 fields available to enter diagnosis as recorded by hospital administration according to ICD codes; applicable to patients discharged directly from the ER. For patients admitted to hospital or ICU, ICD codes are documented in: Hospital.ICDCode1 and Hospital.ICUDischargeICDCode1	Not Applicable
ERDestICDCodes5	TRUE	FALSE	text	Up to 16 fields available to enter diagnosis as recorded by hospital administration according to ICD codes; applicable to patients discharged directly from the ER. For patients admitted to hospital or ICU, ICD codes are documented in: Hospital.ICDCode1 and Hospital.ICUDischargeICDCode1	Not Applicable
ERDestICDCodes6	TRUE	FALSE	text	Up to 16 fields available to enter diagnosis as recorded by hospital administration according to ICD codes; applicable to patients discharged directly from the ER. For patients admitted to hospital or ICU, ICD codes are documented in: Hospital.ICDCode1 and Hospital.ICUDischargeICDCode1	Not Applicable
ERDestICDCodesVersion	TRUE	FALSE	integer	Reflects the version used: ICD-9 or ICD-10. Up to 16 fields available to enter diagnosis as recorded by hospital administration according to ICD codes; applicable to patients discharged directly from the ER. For patients admitted to hospital or ICU, ICD codes are documented in: Hospital.ICDCode1 and Hospital.ICUDischargeICDCode1	9=ICD-9;10=ICD-10
ERDischMotivForDestChoice	TRUE	FALSE	integer	WHY Question: documents main reason for choice of destination at ER discharge.	1=Normal CT;2=Medical necessity;3=Social circumstances;4=No (ICU-) beds available;5=Requiring specialized facilities;88=Unknown;99=Other
ERDischMotivForDestChoiceOther	TRUE	FALSE	text	WHY Question: documents main reason for choice of destination after ER discharge --> Other	Not Applicable
FirstGCS	TRUE	FALSE			Not Applicable
FirstHospAssmtCondition	TRUE	FALSE	integer	Neurological assessment (GCS and pupils) was recorded for the scene of accident, the first hospital (if applicable), the Arrival to ER of the study hospital and post-stabilization. This Describes the condition under which the GCS was assessed at First Hospital.	0=No sedation or paralysis;1=Sedated;2=Paralyzed;3=Temporary stop of sedation/paralysis;4=Reversal of sedation/paralysis;5=Active reversal (pharmacologic) of sedation/paralysis;99=Other
GcsEDArrAssmtCond	TRUE	FALSE	integer	Neurological assessment (GCS and pupils) was recorded for the scene of accident, the first hospital (if applicable), the Arrival to ER of the study hospital and post-stabilization. This describes the condition under which the GCS was assessed at Arrival to ER of the study hospital.	0=No sedation or paralysis;1=Sedated;2=Paralyzed;3=Temporary stop of sedation/paralysis;4=Reversal of sedation/paralysis;5=Active reversal (pharmacologic) of sedation/paralysis;99=Other
GCSEDArrEyes	TRUE	FALSE	text	Neurological assessment (GCS and pupils) was recorded for the scene of accident, the first hospital (if applicable), the Arrival to ER of the study hospital and post-	1=1-None;2=2-To pain;3=3-To speech;4=4-Spontaneously;O=Untestable (other);S=Untestable (swollen);UN=Unknown

Name	Static	Intervention	Format	Description	Possible Values
				stabilization. This reflects the GCS¬†Eye¬†opening at Arrival to ER of study hospital.	
GCSEDArrMotor	TRUE	FALSE	text	Neurological assessment (GCS and pupils) was recorded for the scene of accident, the first hospital (if applicable), the Arrival to ER of the study hospital and post-stabilization. This reflects the GCS Motor score at Arrival to ER of study hospital.	1=1-None;2=2-Abnormal extension;3=3-Abnormal flexion;4=4-Normal flexion/withdrawal;5=5-Localizes to pain;6=6-Obeys command;O=Untestable (Other);P=Untestable (Deep sedation/paralyzed);UN=Unknown
GCSEDArrScore	TRUE	FALSE	text	Neurological assessment (GCS and pupils) was recorded for the scene of accident, the first hospital (if applicable), the Arrival to ER of the study hospital and post-stabilization. This is a Calculated score for Arrival at ER of study hospital: GCSEDArrEyes + GCSEDArrMotor + GCSEDArrVerbal. If one or more of these is Untestable or unknown then = "No Sum"	3=3;4=4;5=5;6=6;7=7;8=8;9=9;10=10;11=11;12=12;13=13;14=14;15=15;No Sum=Untestable/Unknown
GCSEDArrVerbal	TRUE	FALSE	text	Neurological assessment (GCS and pupils) was recorded for the scene of accident, the first hospital (if applicable), the Arrival to ER of the study hospital and post-stabilization. This reflects GCS Verbal score at Arrival to ER of study hospital	1=1-None;2=2-Incomprehensible sound;3=3-Inappropriate words;4=4-Confused;5=5-Oriented;O=Untestable (Other);T=Untestable (Tracheotomy/endotracheal tube);UN=Unknown
GcsEDDischAssmtCond	TRUE	FALSE	integer	Neurological assessment (GCS and pupils) was recorded for the scene of accident, the first hospital (if applicable), the Arrival to ER of the study hospital and post-stabilization. This describes the condition under which the GCS was assessed POST-STABILIZATION.	0=No Sedation or Paralysis;1=Sedated;2=Paralyzed;3=Temporary stop of sedation/paralysis;4=Reversal of sedation/paralysis;5=Active reversal (pharmacologic) of sedation/paralysis;99=Other
GCSEDDischEyes	TRUE	FALSE	text	Neurological assessment (GCS and pupils) was recorded for the scene of accident, the first hospital (if applicable), the Arrival to ER of the study hospital and post-stabilization. This reflects GCS eye opening for the assessment POST¬≠-STABILIZATION.	1=1-None;2=2-To pain;3=3-To speech;4=4-Spontaneously;O=Untestable (other);S=Untestable (swollen);UN=Unknown
GCSEDDischMotor	TRUE	FALSE	text	Neurological assessment (GCS and pupils) was recorded for the scene of accident, the first hospital (if applicable), the Arrival to ER of the study hospital and post-stabilization. This reflects GCS Motor score for the assessment POST¬≠-STABILIZATION.	1=1-None;2=2-Abnormal extension;3=3-Abnormal flexion;4=4-Normal flexion/withdrawal;5=5-Localizes to pain;6=6-Obeys command;O=Untestable (Other);P=Untestable (Deep sedation/paralyzed);UN=Unknown
GCSEDDischScore	TRUE	FALSE	text	Neurological assessment (GCS and pupils) was recorded for the scene of accident, the first hospital (if applicable), the Arrival to ER of the study hospital and post-stabilization. This is the Calculated score for the POST-STABILIZATION assessment: GCSEDDischEyes + GCSEDDischMotor + GCSEDDischVerbal. If one or more of these is Untestable or unknown then = "No Sum"	3=3;4=4;5=5;6=6;7=7;8=8;9=9;10=10;11=11;12=12;13=13;14=14;15=15;No Sum=Untestable/Unknown
GCSEDDischVerbal	TRUE	FALSE	text	Neurological assessment (GCS and pupils) was recorded for the scene of accident, the first hospital (if applicable), the Arrival to ER of the study hospital and post-stabilization. This reflects GCS Verbal score for the assessment POST¬≠-STABILIZATION.	1=1-None;2=2-Incomprehensible sound;3=3-Inappropriate words;4=4-Confused;5=5-Oriented;O=Untestable (Other);T=Untestable (Tracheotomy/endotracheal tube);UN=Unknown
GCSFirstHospEyes	TRUE	FALSE	text	Neurological assessment (GCS and pupils) was recorded for the scene of accident, the first hospital (if applicable), the Arrival to ER of the study hospital and post-stabilization. This reflects GCS eye opening for the assessment at First Hospital.	1=1-None;2=2-To pain;3=3-To speech;4=4-Spontaneously;O=Untestable (other);S=Untestable (swollen);UN=Unknown
GCSFirstHospMotor	TRUE	FALSE	text	Neurological assessment (GCS and pupils) was recorded for the scene of accident, the first hospital (if applicable), the Arrival to ER of the study hospital and post-stabilization. This reflects GCS Motor score for the assessment at First Hospital.	1=1-None;2=2-Abnormal extension;3=3-Abnormal flexion;4=4-Normal flexion/withdrawal;5=5-Localizes to pain;6=6-Obeys command;O=Untestable (Other);P=Untestable (Deep sedation/paralyzed);UN=Unknown

Name	Static	Intervention	Format	Description	Possible Values
GCSFirstHospPupilLftEyeMeasure	TRUE	FALSE	integer	Neurological assessment (GCS and pupils) was recorded for the scene of accident, the first hospital (if applicable), the Arrival to ER of the study hospital and post-stabilization. This reflects Left Pupil Size for the assessment at First Hospital.	Not Applicable
GCSFirstHospPupilReactivityLightLftEyeReslt	TRUE	FALSE	integer	Neurological assessment (GCS and pupils) was recorded for the scene of accident, the first hospital (if applicable), the Arrival to ER of the study hospital and post-stabilization. This reflects the reactivity of the Left Pupil for the assessment at First Hospital.	1=+ (Sluggish);2=+ (Brisk);3=- (Negative)
GCSFirstHospPupilReactivityLightRghtEyeReslt	TRUE	FALSE	integer	Neurological assessment (GCS and pupils) was recorded for the scene of accident, the first hospital (if applicable), the Arrival to ER of the study hospital and post-stabilization. This reflects the reactivity of the Right Pupil for the assessment at First Hospital.	1=+ (Sluggish);2=+ (Brisk);3=- (Negative)
GCSFirstHospPupilRightEyeMeasure	TRUE	FALSE	integer	Neurological assessment (GCS and pupils) was recorded for the scene of accident, the first hospital (if applicable), the Arrival to ER of the study hospital and post-stabilization. This reflects Right Pupil Size for the assessment at First Hospital.	Not Applicable
GCSFirstHospPupilSymmetry	TRUE	FALSE	integer	Neurological assessment (GCS and pupils) was recorded for the scene of accident, the first hospital (if applicable), the Arrival to ER of the study hospital and post-stabilization. This reflects the pupil symmetry for the assessment at First Hospital.	1=Equal;2=Unequal R>L;3=Unequal L>R;66=Untestable;88=Unknown
GCSFirstHospScore	TRUE	FALSE	text	Neurological assessment (GCS and pupils) was recorded for the scene of accident, the first hospital (if applicable), the Arrival to ER of the study hospital and post-stabilization. This is the Calculated Score for the assessment at First Hospital: GCSFirstHospEyes + GCSFirstHospMotor + GCSFirstHospVerbal. If one or more of these is Untestable or unknown then = "No Sum" Sum Score may be recorded with no components	3=3;4=4;5=5;6=6;7=7;8=8;9=9;10=10;11=11;12=12;13=13;14=14;15=15;No Sum=Untestable/Unknown
GCSFirstHospVerbal	TRUE	FALSE	text	Neurological assessment (GCS and pupils) was recorded for the scene of accident, the first hospital (if applicable), the Arrival to ER of the study hospital and post-stabilization. This reflects the GCS Verbal score for the assessment at First Hospital.	1=1-None;2=2-Incomprehensible sound;3=3-Inappropriate words;4=4-Confused;5=5-Oriented;O=Untestable (Other);T=Untestable (tracheotomy/endotracheal tube);UN=Unknown
GCSMotorBaselineDerived	TRUE	FALSE	integer	This is a derived variable calculated centrally. It represents the GCS motor score for baseline risk adjustment with missing values imputed using IMPACT methodology - take Poststabilisation value and if absent work back in time towards prehospital values until non-missing value found. RECOMMENDED FOR BASELINE RISK ADJUSTMENT.	1=1-None;2=2-Abnormal extension;3=3-Abnormal flexion;4=4-Normal flexion/withdrawal;5=5-Localizes to pain;6=6-Obeys command
GCSOtherAssmtConditions	TRUE	FALSE	integer	Neurological assessment (GCS and pupils) was recorded for the scene of accident, the first hospital (if applicable), the Arrival to ER of the study hospital and post-stabilization. There was also an additional option "Other" assessment. This describes the condition under which the GCS was assessed for the assessment "Other".	0=No Sedation or Paralysis;1=Sedated;2=Paralyzed;3=Temporary stop of sedation/paralysis;4=Reversal of sedation/paralysis;5=Active reversal (pharmacologic) of sedation/paralysis;99=Other
GCSOtherEyes	TRUE	FALSE	text	Neurological assessment (GCS and pupils) was recorded for the scene of accident, the first hospital (if applicable), the Arrival to ER of the study hospital and post-stabilization. There was also an additional option "Other" assessment. This describes	1=1-None;2=2-To pain;3=3-To speech;4=4-Spontaneously;O=Untestable (other);S=Untestable (swollen);UN=Unknown

Name	Static	Intervention	Format	Description	Possible Values
				the GCS Eye opening for the assessment "Other".	
GCSOtherMotor	TRUE	FALSE	text	Neurological assessment (GCS and pupils) was recorded for the scene of accident, the first hospital (if applicable), the Arrival to ER of the study hospital and post-stabilization. There was also an additional option "Other" assessment. This describes GCS Motor score for the assessment "Other".	1=1-None;2=2-Abnormal extension;3=3-Abnormal flexion;4=4-Normal flexion/withdrawal;5=5-Localizes to pain;6=6-Obeys command;O=Untestable (Other);P=Untestable (Deep sedation/paralyzed);UN=Unknown
GCSOtherPupilLftEyeMeasure	TRUE	FALSE	integer	Neurological assessment (GCS and pupils) was recorded for the scene of accident, the first hospital (if applicable), the Arrival to ER of the study hospital and post-stabilization. There was also an additional option "Other" assessment. This describes Left Pupil Size for the assessment "Other".	Not Applicable
GCSOtherPupilReactivityLightLftEyeReslt	TRUE	FALSE	integer	Neurological assessment (GCS and pupils) was recorded for the scene of accident, the first hospital (if applicable), the Arrival to ER of the study hospital and post-stabilization. There was also an additional option "Other" assessment. This describes the reactivity of the LEFT pupil for the assessment "Other".	1=+ (Sluggish);2=+ (Brisk);3=- (Negative)
GCSOtherPupilRightEyeMeasure	TRUE	FALSE	integer	Neurological assessment (GCS and pupils) was recorded for the scene of accident, the first hospital (if applicable), the Arrival to ER of the study hospital and post-stabilization. There was also an additional option "Other" assessment. This describes the Right pupil size for the assessment "Other".	Not Applicable
GCSOtherPupilSymmetry	TRUE	FALSE	integer	Neurological assessment (GCS and pupils) was recorded for the scene of accident, the first hospital (if applicable), the Arrival to ER of the study hospital and post-stabilization. There was also an additional option "Other" assessment. This describes the pupil symmetry for the assessment "Other".	1=Equal;2=Unequal R>L;3=Unequal L>R;66=Untestable;88=Unknown
GCSOtherReactivityLightRghtEyeReslt	TRUE	FALSE	integer	Neurological assessment (GCS and pupils) was recorded for the scene of accident, the first hospital (if applicable), the Arrival to ER of the study hospital and post-stabilization. There was also an additional option "Other" assessment. This describes the reactivity of the Right Pupil for the assessment "Other".	1=+ (Sluggish);2=+ (Brisk);3=- (Negative)
GCSOtherScore	TRUE	FALSE	text	Neurological assessment (GCS and pupils) was recorded for the scene of accident, the first hospital (if applicable), the Arrival to ER of the study hospital and post-stabilization. There was also an additional option "Other" assessment. This is the Calculated Score for the assessment "Other": GCSOtherEyes + GCSOtherMotor + GCSOtherVerbal. If one or more of these is Untestable or unknown then = "No Sum" Sum score may be reported when components not available	3=3;4=4;5=5;6=6;7=7;8=8;9=9;10=10;11=11;12=12;13=13;14=14;15=15;No Sum=Untestable/Unknown
GCSOtherVerbal	TRUE	FALSE	text	Neurological assessment (GCS and pupils) was recorded for the scene of accident, the first hospital (if applicable), the Arrival to ER of the study hospital and post-stabilization. There was also an additional option "Other" assessment. This describes the GCS Verbal score for the assessment "Other".	1=1-None;2=2-Incomprehensible sound;3=3-Inappropriate words;4=4-Confused;5=5-Oriented;O=Untestable (Other);T=Untestable (tracheotomy/endotracheal tube);UN=Unknown
GCSPreHospBestEyes	TRUE	FALSE	text	Neurological assessment (GCS and pupils) was recorded for the scene of accident, the first hospital (if applicable), the Arrival to ER of the study hospital and post-stabilization. This describes the GCS Eye	1=1-None;2=2-To pain;3=3-To speech;4=4-Spontaneously;O=Untestable (Other);S=Untestable (swollen);UN=Unknown

Name	Static	Intervention	Format	Description	Possible Values
				opening for the assessment at Scene Of Accident.	
GCSPreHospBestMotor	TRUE	FALSE	text	Neurological assessment (GCS and pupils) was recorded for the scene of accident, the first hospital (if applicable), the Arrival to ER of the study hospital and post-stabilization. This describes the GCS Motor score for the assessment at Scene Of Accident.	1=1-None;2=2-Abnormal extension;3=3-Abnormal flexion;4=4-Normal flexion/withdrawal;5=5-Localizes to pain;6=6-Obeys command;O=Untestable (Other);P=Untestable (Deep sedation/paralyzed);UN=Unknown
GCSPreHospBestScore	TRUE	FALSE	text	Neurological assessment (GCS and pupils) was recorded for the scene of accident, the first hospital (if applicable), the Arrival to ER of the study hospital and post-stabilization. This is the Calculated Score for the assessment at Scene Of Accident: GCSPreHospBestEyes + GCSPreHospBestMotor + GCSPreHospBestVerbal. If one or more of these is Untestable or unknown then = "No Sum" GCS sum score may be recorded when components not available - check "InjuryHx.GCSPreHospBestReportedTotalScore"	3=3;4=4;5=5;6=6;7=7;8=8;9=9;10=10;11=11;12=12;13=13;14=14;15=15;No Sum=Untestable/Unknown
GCSPreHospBestVerbal	TRUE	FALSE	text	Neurological assessment (GCS and pupils) was recorded for the scene of accident, the first hospital (if applicable), the Arrival to ER of the study hospital and post-stabilization. This describes the GCS Verbal score for the assessment at Scene Of Accident.	1=1-None;2=2-Incomprehensible sound;3=3-Inappropriate words;4=4-Confused;5=5-Oriented;O=Untestable (Other);T=Untestable (Tracheotomy/endotracheal tube);UN=Unknown
GcsPreHospLftEyeMeasr	TRUE	FALSE	text	Neurological assessment (GCS and pupils) was recorded for the scene of accident, the first hospital (if applicable), the Arrival to ER of the study hospital and post-stabilization. This describes the Left Pupil Size for the assessment at Scene Of Accident.	Not Applicable
GCSPreHospPupilReactivityLghtLftEyeResult	TRUE	FALSE	integer	Neurological assessment (GCS and pupils) was recorded for the scene of accident, the first hospital (if applicable), the Arrival to ER of the study hospital and post-stabilization. This describes the reactivity of the Left pupil for the assessment at Scene Of Accident.	1=+ (Sluggish);2=+ (Brisk);3=- (Negative)
GCSPreHospPupilReactivityLghtRghtEyeResult	TRUE	FALSE	integer	Neurological assessment (GCS and pupils) was recorded for the scene of accident, the first hospital (if applicable), the Arrival to ER of the study hospital and post-stabilization. This describes the reactivity of the Right pupil for the assessment at Scene Of Accident.	1=+ (Sluggish);2=+ (Brisk);3=- (Negative)
GCSPreHospPupilSymmetry	TRUE	FALSE	integer	Neurological assessment (GCS and pupils) was recorded for the scene of accident, the first hospital (if applicable), the Arrival to ER of the study hospital and post-stabilization. This describes the Pupil symmetry for the assessment at Scene Of Accident.	1=Equal;2=Unequal R>L;3=Unequal L>R;66=Untestable;88=Unknown
GcsPreHospRghtEyeMeasr	TRUE	FALSE	text	Neurological assessment (GCS and pupils) was recorded for the scene of accident, the first hospital (if applicable), the Arrival to ER of the study hospital and post-stabilization. This describes the Right Pupil Size for the assessment at Scene Of Accident.	Not Applicable
GCSScoreBaselineDerived	TRUE	FALSE	integer	This is a derived variable calculated centrally. It represents the total GCS (single timepoint) for baseline risk adjustment with missing values imputed using IMPACT methodology - take Poststabilisation value and if absent work back in time towards prehospital values until non-missing value found. Intubated / untestable V score treated	3=3;4=4;5=5;6=6;7=7;8=8;9=9;10=10;11=11;12=12;13=13;14=14;15=15

Name	Static	Intervention	Format	Description	Possible Values
				as unknown. RECOMMENDED FOR BASELINE RISK ADJUSTMENT.	
GOAT	TRUE	FALSE			Not Applicable
GOATBrnDate	TRUE	FALSE	integer	Reflects for the GOAT outcome test on the question "When were you born?" if the subject made an error or not in his reply.	-4=Error (-4);0=No Error
GOATBuldngLoc	TRUE	FALSE	integer	Reflects for the GOAT outcome test on the question "Where are you now?" if the subject made an error or not in his reply.	-5=Error (-5);0=No Error
GOATCityLoc	TRUE	FALSE	integer	Reflects for the GOAT outcome test on the question "Where are you now (which city)?" if the subject made an error or not in his reply.	-5=Error (-5);0=No Error
GOATCrntTm	TRUE	FALSE	integer	Reflects for the GOAT outcome test on the question "What time is it now?" if the subject made an error or not in his reply.	-5=Two and one-half hour + error (-5);-4=Two hour error (-4);-3=One and one-half hour error (-3);-2=One hour error (-2);-1=Half-hour error (-1);0=No Error
GOATDayDate	TRUE	FALSE	integer	Reflects for the GOAT outcome test on the question "What day of the week is it?" if the subject made an error or not in his reply.	-3=Three day error (-3);-2=Two day error (-2);-1=One day error (-1);0=No Error
GOATDayMnthDate	TRUE	FALSE	integer	Reflects for the GOAT outcome test on the question "What day of the month is it? (i.e. the date)" if the subject made an error or not in his reply.	-5=Five day + error (-5);-4=Four day error (-4);-3=Three day error (-3);-2=Two day error (-2);-1=One day error (-1);0=No Error
GOATDtlRslt	TRUE	FALSE	integer	Reflects for the GOAT outcome test on the question " What is the first event you can remember after the injury?" if the subject made an error or not in his reply.	-5=Error (-5);0=No Error
GOATFirEvntRslt	TRUE	FALSE	integer	Reflects for the GOAT outcome test on the question "What is the first event you can remember after the injury?" if the subject made an error or not in his reply.	-5=Error (-5);0=No Error
GOATFirEvntRsltTxt	TRUE	FALSE	text	Reflects for the GOAT outcome test on the question "What is the first event you can remember after the injury?" the details described by the subject.	Not Applicable
GOATHospAdmDate	TRUE	FALSE	integer	Reflects for the GOAT outcome test on the question "On what date were you admitted to the hospital?" if the subject made an error or not in his reply.	-5=Error (-5);0=No Error
GOATLiveLoc	TRUE	FALSE	integer	Reflects for the GOAT outcome test on the question "Where do you live?" if the subject made an error or not in his reply.	-4=Error (-4);0=No Error
GOATLstEvntDtlRslt	TRUE	FALSE	integer	Reflects for the GOAT outcome test on the question "What is the last event you can recall before the injury - Can you describe in detail?" if the subject made an error or not in his reply.	-5=Error (-5);0=No Error
GOATLstEvntRslt	TRUE	FALSE	integer	Reflects for the GOAT outcome test on the question "What is the last event you can recall before the injury?" if the subject made an error or not in his reply.	-5=Error (-5);0=No Error
GOATLstEvntRsltTxt	TRUE	FALSE	text	Reflects for the GOAT outcome test the reply on the question "What is the last event you can recall before the injury - Can you describe in detail?"	Not Applicable
GOATMnthDate	TRUE	FALSE	integer	Reflects for the GOAT outcome test on the question "What is the month?" if the subject made an error or not in his reply.	-15=Three or more month error (-15);-10=Two month error (-10);-5=One month error (-5);0=No Error
GOATNeuroPsychCompCode	TRUE	FALSE	integer	Reflects if GOAT test was done or not. Neuropsych testing was conducted during scheduled follow-up visits to hospital according to study protocol. Cross-sectional assessments across all strata was performed at 6 mnths after injury. For patients included in the MR substudy full testing was conducted for ER patients at: 2-3 weeks, 3 mnths and 6 mnths; Adm and ICU strata: 6 mnths, 12 mnths and 24 mnths Subjects were only considered testable if the GOAT was >=65. Neuropsych testing included: GOAT, RAVLT, TMT, CANTAB subtests, 10 m walk, Timed up-and-go, as well as Participant Questionnaire B.. "Untestable"	1=1.0 Test not done;2=2.0 Test attempted but not completed;3=3.0 Test completed

Name	Static	Intervention	Format	Description	Possible Values
				patients received the GOAT and JFK-Coma Recovery Scale-revised..	
GOATNm	TRUE	FALSE	integer	Reflects for the GOAT outcome test on the question "What is your name?" if the subject made an error or not in his reply.	-2=Error (-2);0=No Error
GOATOutcome	TRUE	FALSE	text	The total GOAT score is calculated as 100 - Total error points; Interpretation: 76-100 = Normal / 66-75 = Borderline / <66 = Impaired	Borderline=Borderline;Impaired=Impaired;Normal=Normal
GOATTestAttemptdNotCompOptions	TRUE	FALSE	decimal	Documents reasons why GOAT was not completed when it was initially attempted (7 categories)	2.1=Not completed - Cognitive/neurological deficits;2.2=Not completed - Non-neurological/physical reason;2.3=Not completed - Lack of effort/uncooperative;2.4=Not completed - Language;2.5=Not completed - Illness/fatigue;2.6=Not completed - Logistical reasons, other reasons;2.7=Not completed - Examiner error
GOATTestCompletedOptions	TRUE	FALSE	decimal	Reflects the completion of the GOAT outcome test (Test completed in full results valid/Test completed Nonstandard, results valid/Nonstandard administration/Other)	3=Test completed in full - results valid;3.1=Test completed - Non-standard, results valid;3.2=Non-standard administration - Other
GOATTestComplNonStandAdminOTHER	TRUE	FALSE	text		Not Applicable
GOATTestNotDoneOptions	TRUE	FALSE	decimal	Documents reasons why GOAT was not attempted (8 categories)	1.1=Not attempted - Cognitive/neurological deficits;1.2=Not attempted - Non-neurological/physical reasons;1.3=Not attempted - Lack of effort/uncooperative;1.4=Not attempted - Language;1.5=Not attempted - Illness/fatigue;1.6=Not attempted - Logistical reasons, other reasons;1.7=Not attempted- Return to all normal activities;1.8=Not attempted - Patient not available
GOATTestNotDoneOptionsOTHER	TRUE	FALSE	text	Documents the "other" reason why GOAT was not attempted	Not Applicable
GOATTotError	TRUE	FALSE	integer	Total error score for the GOAT outcome test. The total error score is calculated automatically.	Not Applicable
GOATTotScr	TRUE	FALSE	integer	Total actual score for the GOAT test. The total GOAT score is calculated as 100 - Total error points; Interpretation: 76-100 = Normal / 66-75 = Borderline / <66 = Impaired	Not Applicable
GOATTranspTyp	TRUE	FALSE	integer	Reflects for the GOAT outcome test on the question "How did you get to the hospital?" if the subject made an error or not in his reply.	-5=Error (-5);0=No Error
GOATYrDate	TRUE	FALSE	integer	Reflects for the GOAT outcome test on the question "What is the year?" if the subject made an error or not in his reply.	-30=Three or more year error (-30);-20=Two year error (-20);-10=One year error (-10);0=No Error
HighestGCSMotorDerived	TRUE	FALSE	integer	This is a derived variable calculated centrally. It represents the GCS motor score for baseline risk adjustment with missing values imputed using ‚Äòhighest‚Äô value (best neurology) methodology- take best neurology of any of prehospital to Poststabilisation time points. DEPRECATED: we recommend using GCSMotorBaselineDerived for baseline risk adjustment instead (higher pseudo-R-squared in proportional odds model).	1=1-None;2=2-Abnormal extension;3=3-Abnormal flexion;4=4-Normal flexion/withdrawal;5=5-Localizes to pain;6=6-Obeys command
HighestGCSTotalDerived	TRUE	FALSE	integer	This is a derived variable calculated centrally. Total GCS (single timepoint) for baseline risk adjustment with missing values imputed using ‚Äòhighest‚Äô value (best neurology) methodology - take best neurology of any of prehospital to Poststabilisation time points. Intubated / untestable V score treated as unknown. DEPRECATED: we recommend using GCSScoreBaselineDerived for baseline risk adjustment instead (higher pseudo-R-squared in proportional odds model).	3=3;4=4;5=5;6=6;7=7;8=8;9=9;10=10;11=11;12=12;13=13;14=14;15=15
HighestPupilsDerived	TRUE	FALSE	integer	This is a derived variable calculated centrally. Number of unreactive pupils for baseline risk adjustment with missing values imputed using ‚Äòhighest‚Äô value (best neurology) methodology- take best neurology of any of prehospital to Poststabilisation time points. Untestable pupil ignored: I.e. 1 reactive + 1 untestable	0=Both reacting;1=One reacting;2=Both unreacting

Name	Static	Intervention	Format	Description	Possible Values
				= 1 reactive (this assumption applies only to a small proportion of the data). DEPRECATED: we recommend using PupilsBaselineDerived for baseline risk adjustment instead (higher pseudo-R-squared in proportional odds model).	
ICUAdmReason	TRUE	FALSE	integer	Main reason for admission to ICU	1=Mechanical ventilation;2=Frequent neurological observations;3=Haemodynamic invasive monitoring;4=Extracranial injuries;5=Neurological operation;6=Clinical deterioration;99=Other
ICUAdmReasonOther	TRUE	FALSE	text	Specifies the "other" if the main reason for admission to the ICU was other than the pre-defined list.	Not Applicable
InterventRadiology	TRUE	FALSE	integer	Reflects if at time of discharge from the ER some Interventional Radiology was scheduled	0=No;1=Yes;88=Unknown
LOCAOC	TRUE	FALSE	integer	TBI may be present in the absence of LOC or PTA. Alteration of Consciousness (AOC) is then the main presenting symptom and considered diagnostic of TBI. Details of symptoms are captured in the Rivermead Questionnaire.	0=No;1=Yes, immediate;2=Not tested due to LOC;3=Suspected;4=Yes, delayed onset;88=Unknown
LOCAOCDelayedHrs	TRUE	FALSE	integer	TBI may be present in the absence of LOC or PTA. Alteration of Consciousness (AOC) is then the main presenting symptom and considered diagnostic of TBI. This reflects the Number of hours after injury that alteration of consciousness occurred - Only in case of delayed onset. Details of symptoms are captured in the Rivermead Questionnaire.	Not Applicable
LOCAOCDuration	TRUE	FALSE	integer	TBI may be present in the absence of LOC or PTA. Alteration of Consciousness (AOC) is then the main presenting symptom and considered diagnostic of TBI. This reflects the Duration of alteration of consciousness. Details of symptoms are captured in the Rivermead Questionnaire.	0=None;2=<1 minute;3=1-29 minutes;4=30-59 minutes;5=1-24 hours;6=1-7 days;7=>7 days;88=Unknown
LOCAOCReportedBy	TRUE	FALSE	integer	TBI may be present in the absence of LOC or PTA. Alteration of Consciousness (AOC) is then the main presenting symptom and considered diagnostic of TBI. This reflects by whom the alteration of consciousness was reported. Details of symptoms are captured in the Rivermead Questionnaire.	1=Patient;2=Witness;3=Clinical interview;4=Medical chart;5=Not available
LOCDuration	TRUE	FALSE	integer	LOC and PTA are reported as part of the neurological assessment. This reflects the duration of Loss of Consciousness (LOC). Note: for patients admitted to hospital, the time to obeying commands is documented on hospital discharge: Hospital.HospDischargeTimeToObeyCommands	0=No return of consciousness;2=<1 minute;3=1-29 minutes;4=30-59 minutes;5=1-24 hours;6=1-7 days;7=>7 days;88=Unknown
LOCGCSSumDet	TRUE	FALSE	integer	LOC and PTA are reported as part of the neurological assessment. This reflects for the Loss of Consciousness (LOC) the GCS sum score deterioration within one hour after presentation.	0=None;1=1 point;2=2 or more points;88=Unknown
LOCLossOfConsciousness	TRUE	FALSE	integer	LOC and PTA are reported as part of the neurological assessment. Loss of Consciousness (LOC) is a definite sign of TBI. However, TBI may be present without any LOC. Presence and duration is captured.	0=No;1=Yes;3=Suspected;88=Unknown
LOCLucidInterval	TRUE	FALSE	integer	LOC and PTA are reported as part of the neurological assessment. Lucid Interval is defined as a temporary improvement in a patient's condition after a traumatic brain injury, after which the condition deteriorates. A lucid interval is especially indicative of an epidural hematoma. An estimated 20 to 50% of patients with epidural hematoma experience such a lucid interval.	0=No;1=Yes;88=Unknown
LOCLucidIntervalHrs	TRUE	FALSE	decimal	Lucid Interval is defined as a temporary improvement in a patient's condition after a	Not Applicable

Name	Static	Intervention	Format	Description	Possible Values
				traumatic brain injury, after which the condition deteriorates. This reflects the Number of hours after injury that secondary deterioration occurred (in case Lucid Interval = Yes)	
LOCPTA	TRUE	FALSE	integer	LOC and PTA are reported as part of the neurological assessment. Post-traumatic amnesia (PTA) is the period after the injury that the patient cannot remember. In contrast to retrograde amnesia, the duration of PTA remains constant over time. To document presence/absence of PTA on discharge from the ER, the GOAT questionnaire is requested: Outcomes.GOATDate	0=No;1=Yes, ongoing;2=Yes, resolved;3=Suspected;88=Unknown
LOCPTADuration	TRUE	FALSE	integer	LOC and PTA are reported as part of the neurological assessment. This variable is recorded only Only if PTA is yes. The duration of PTA reflects the severity of TBI. In patients with more sever TBI, the duration of PTA cannot be determined on presentation. For patients admitted to hospital, the duration of TBI in days is also captured on hospital discharge:.Hospital.HospDischPTADays	0=None;2=<1 hour;5=1-24 hours;6=1-7 days;7=7-28 days;8=1-2 hours;9=2-4 hours;10=4-24 hours;11=>1 day;28=>28;77=N/A (e.g. death);88=Unknown
LOCPTAReportedBy	TRUE	FALSE	integer	LOC and PTA are reported as part of the neurological assessment. This reflects by whom PTA is reported.	1=Patient;2=Witness;3=Retrospective assessment/ clinical interview;4=Medical chart;5=Not available;6=Prospective assessment with PTA scale
LOCPTAScale	TRUE	FALSE	integer	LOC and PTA are reported as part of the neurological assessment. In some centres, prospective assessment of amnesia (PTA) after TBI is performed using a dedicated scale. This variable documents the scale used.	1=GOAT;2=Westmead;3=O-Log;4=Nijmegen PTA scale;99=Other
LOCReportedBy	TRUE	FALSE	integer	LOC and PTA are reported as part of the neurological assessment. This variable reflects by whom LOC was reported.	1=Self report;2=Witness;3=Clinical interview;4=Medical chart;5=Not available
LOCRGA	TRUE	FALSE	integer	This reflects presence or absence of retrograde amnesia during neurological assessment. Amnesia after injury is a sign of TBI. Retrograde amnesia is the period before the injury that the patient cannot remember. The duration of retrograde amnesia becomes shorter as the injury is longer ago. The duration of retrograde amnesia is therefore dependent on time after injury at which it was assessed.	0=No;1=Yes;88=Unknown
LOCRGADur	TRUE	FALSE	integer	This reflects the duration of retrograde amnesia is present during neurological assessment.	0=None;1=<30;2=>= 30 minutes;88=Unknown
LOCRGAReportBy	TRUE	FALSE	integer	This reflects by whom Retrograde amnesia was reported if present during neurological assessment.	1=Self report;2=Witness;3=Clinical interview;4=Medical chart;5=Not available
NeuroAssmtsAVPU	TRUE	FALSE	text	AVPU is scored as part of the neurological assessment on arrival to the ER. The AVPU scale (an acronym from "alert, voice, pain, unresponsive") is a system by which a health care professional can measure and record a patient's responsiveness, indicating their level of consciousness.	88=Unknown;A=Patient is awake;P=The patient responds to painful stimulation;U=The patient is completely unresponsive;V=Patient responds to verbal stimulation
OtherGCS	TRUE	FALSE			Not Applicable
PainScale	TRUE	FALSE	integer	During neurological assessment at arrival to ER an overall rating was recorded for pain intensity going from 0 (zero pain) to 100 (unbearable pain).	Not Applicable
PreHospAssmtConditions	TRUE	FALSE	integer	Neurological assessment (GCS and pupils) was recorded for the scene of accident, the first hospital (if applicable), the Arrival to ER of the study hospital and post-stabilization. This describes the condition under which the GCS was assessed for the assessment at Scene Of Accident.	0=No sedation or paralysis;1=Sedated;2=Paralyzed;3=Temporary stop of sedation/paralysis;4=Reversal of sedation/paralysis;5=Active reversal (pharmacologic) of sedation/paralysis;99=Other
PresArrivalMethod	TRUE	FALSE	integer	Reflects the mode of transportation used to transport the subject from the scene of accident to the hospital.	1=Ambulance;2=Helicopter;3=Medical mobile team;4=Walk in or drop off;99=Other

Name	Static	Intervention	Format	Description	Possible Values
PresCirculationTreatmentCPR	TRUE	FALSE	integer	The status of Airway, Breathing and Circulation on scene are documented. This records Emergency care treatment on scene performed with regard to Circulation: CPR (Cardio-pulmonary resuscitation) ER arrival status is documented at: InjuryHX.EDArrivalCirculation	0=No;1=Yes
PresCirculationTreatmentIVFluids	TRUE	FALSE	integer	The status of Airway, Breathing and Circulation on scene are documented. This records Emergency care treatment on scene performed with regard to Circulation: IV Fluids ER arrival status is documented at: InjuryHX.EDArrivalCirculation	0=No;1=Yes
PresCirculationTreatmentNone	TRUE	FALSE	integer	The status of Airway, Breathing and Circulation on scene are documented. This records Emergency care treatment on scene performed with regard to Circulation: None ER arrival status is documented at: InjuryHX.EDArrivalCirculation	0=No;1=Yes
PresCTBrain	TRUE	FALSE	integer	Only applicable in case of secondary referral. Reflects CT brain procedure performed at first hospital (not study hospital).	0=No;1=Yes;88=Unknown
PresEmergencyCare	TRUE	FALSE	integer	Reflects if and by whom emergency medical care was given at the scene of accident (highest level of assistance)	0=None;1=Untrained person (by stander);2=Trainer/coach;3=Military, non-medic;4=Paramedic;5=Nurse;6=Physician;7=Medical rescue team;99=Other
PresEmergencyCareIntubation	TRUE	FALSE	integer	Reflects if intubation was performed on scene.	0=No;1=Yes;88=Unknown
PresEmergencyCareSuppOxygen	TRUE	FALSE	integer	Reflects if supplemental oxygen was given on scene.	0=No;1=Yes;88=Unknown
PresEmergencyCareVentilation	TRUE	FALSE	integer	Reflects if Mechanical Ventilation was done on scene.	0=No;1=Yes;88=Unknown
PresEmergencyServiceAmbuBasic	TRUE	FALSE	integer	Reflects type of Emergency service involved at accident scene --> Ambulance (basic EMT¬≠B)	0=No;1=Yes
PresEmergencyServiceAmbuSpec	TRUE	FALSE	integer	Reflects type of Emergency service involved at accident scene --> Ambulance specialized (EMT¬≠P)	0=No;1=Yes
PresEmergencyServiceFirefighter	TRUE	FALSE	integer	Reflects type of Emergency service involved at accident scene --> Firefighter	0=No;1=Yes
PresEmergencyServiceHelicopter	TRUE	FALSE	integer	Reflects type of Emergency service involved at accident scene --> Helicopter	0=No;1=Yes
PresEmergencyServiceNone	TRUE	FALSE	integer	Reflects type of mergency service involved at accident scene --> None	0=No;1=Yes
PresEmergencyServicePolice	TRUE	FALSE	integer	Reflects type of Emergency service involved at accident scene --> Police	0=No;1=Yes
PresERExtracranialSurg	TRUE	FALSE	integer	Only applicable in case of secondary referral. Reflects Emergency Extracranial surgery procedure performed at first hospital (not study hospital).	0=No;1=Yes;88=Unknown
PresERIntracranialSurg	TRUE	FALSE	integer	Only applicable in case of secondary referral. Reflects the Emergency intracranial surgery Procedure performed at first hospital (not study hospital).	0=No;1=Yes;88=Unknown
PresIntubation	TRUE	FALSE	integer	Only applicable in case of secondary referral. Reflects procedure of intubation performed at first hospital (not study hospital).	0=No;1=Yes;88=Unknown
PresTBIRef	TRUE	FALSE	integer	Reflects if the subject was transported from the scene of accident immediately to the study hospital (primary referral) or if secondary referral occurred from a first hospital to the Study hospital.	1=Primary;2=Secondary
PupilsBaselineDerived	TRUE	FALSE	integer	This is a derived variable calculated centrally. Number of unreactive pupils for baseline risk adjustment with missing values imputed using IMPACT methodology- take Poststabilisation value and if absent work back in time towards prehospital values until non-missing value found. Untestable pupil ignored: I.e. 1 reactive + 1 untestable = 1 reactive (this assumption applies only to a small proportion of the data).	0=Both reacting;1=One reacting;2=Both unreacting

Name	Static	Intervention	Format	Description	Possible Values
				RECOMMENDED FOR BASELINE RISK ADJUSTMENT.	
PupilsNonSymmetric	TRUE	FALSE	integer	Pupil symmetry derived variable calculated from (GCSFirstHospPupilSymmetry,EDArrPupilSymmetry,GCSPreHospPupilSymmetry,EDDischPupilSymmetry,PupilsNonSymmetric)	0=No;1=Yes
RPQ	TRUE	FALSE			Not Applicable
RPQ13Score	TRUE	FALSE	integer	Score for the Rivermead Assessment RPQ-13. Score for the Not scored by investigator, calculated score. Baseline RPQ documents complaints and symptoms commonly reported after TBI. Baseline RPQ can only be assessed in conscious subjects who are out of PTA. Provides further details on AOC.	Not Applicable
RPQ3Score	TRUE	FALSE	integer	Score for the Rivermead Assessment RPQ-3. Not scored by investigator, calculated score. Baseline RPQ documents complaints and symptoms commonly reported after TBI. Baseline RPQ can only be assessed in conscious subjects who are out of PTA. Provides further details on AOC.	Not Applicable
RPQBlurredVision	TRUE	FALSE	integer	This variable reflects if the subject, compared with before the accident, now (over the last 24 hours) suffers from blurred vision, as part of the Rivermead RPQ assessment.	0=0-Not experienced at all;1=1- No more of a problem;2=2- A mild problem;3=3- A moderate problem;4=4- A severe problem
RPQDepressed	TRUE	FALSE	integer	This variable reflects if the subject, compared with before the accident, now (over the last 24 hours) suffers from feeling depressed or tearful, as part of the Rivermead RPQ assessment.	0=0-Not experienced at all;1=1- No more of a problem;2=2- A mild problem;3=3- A moderate problem;4=4- A severe problem
RPQDizziness	TRUE	FALSE	integer	This variable reflects if the subject, compared with before the accident, now (over the last 24 hours) suffers from feelings of dizziness, as part of the Rivermead RPQ assessment.	0=0-Not experienced at all;1=1- No more of a problem;2=2- A mild problem;3=3- A moderate problem;4=4- A severe problem
RPQDoubleVision	TRUE	FALSE	integer	This variable reflects if the subject, compared with before the accident, now (over the last 24 hours) suffers from double vision, as part of the Rivermead RPQ assessment.	0=0-Not experienced at all;1=1- No more of a problem;2=2- A mild problem;3=3- A moderate problem;4=4- A severe problem
RPQFatigue	TRUE	FALSE	integer	This variable reflects if the subject, compared with before the accident, now (over the last 24 hours) suffers from Fatigue, tiring more easily, as part of the Rivermead RPQ assessment.	0=0-Not experienced at all;1=1- No more of a problem;2=2- A mild problem;3=3- A moderate problem;4=4- A severe problem
RPQForgetful	TRUE	FALSE	integer	This variable reflects if the subject, compared with before the accident, now (over the last 24 hours) suffers from Forgetfulness, poor memory, as part of the Rivermead RPQ assessment.	0=0-Not experienced at all;1=1- No more of a problem;2=2- A mild problem;3=3- A moderate problem;4=4- A severe problem
RPQFrustrated	TRUE	FALSE	integer	This variable reflects if the subject, compared with before the accident, now (over the last 24 hours) suffers from Feeling frustrated or impatient, as part of the Rivermead RPQ assessment.	0=0-Not experienced at all;1=1- No more of a problem;2=2- A mild problem;3=3- A moderate problem;4=4- A severe problem
RPQHeadaches	TRUE	FALSE	integer	This variable reflects if the subject, compared with before the accident, now (over the last 24 hours) suffers from Headaches, as part of the Rivermead RPQ assessment.	0=0-Not experienced at all;1=1- No more of a problem;2=2- A mild problem;3=3- A moderate problem;4=4- A severe problem
RPQIrritable	TRUE	FALSE	integer	This variable reflects if the subject, compared with before the accident, now (over the last 24 hours) suffers from Being irritable, easily angered, as part of the Rivermead RPQ assessment.	0=0-Not experienced at all;1=1- No more of a problem;2=2- A mild problem;3=3- A moderate problem;4=4- A severe problem
RPQLightSensitivity	TRUE	FALSE	integer	This variable reflects if the subject, compared with before the accident, now (over the last 24 hours) suffers from Light sensitivity (easily upset by bright light), as part of the Rivermead RPQ assessment.	0=0-Not experienced at all;1=1- No more of a problem;2=2- A mild problem;3=3- A moderate problem;4=4- A severe problem

Name	Static	Intervention	Format	Description	Possible Values
RPQLongerToThink	TRUE	FALSE	integer	This variable reflects if the subject, compared with before the accident, now (over the last 24 hours) suffers from Taking longer to think, as part of the Rivermead RPQ assessment.	0=0-Not experienced at all;1=1- No more of a problem;2=2- A mild problem;3=3- A moderate problem;4=4- A severe problem
RPQNausea	TRUE	FALSE	integer	This variable reflects if the subject, compared with before the accident, now (over the last 24 hours) suffers from Nausea and/or vomiting, as part of the Rivermead RPQ assessment.	0=0-Not experienced at all;1=1- No more of a problem;2=2- A mild problem;3=3- A moderate problem;4=4- A severe problem
RPQNoiseSensitivity	TRUE	FALSE	integer	This variable reflects if the subject, compared with before the accident, now (over the last 24 hours) suffers from Noise sensitivity (easily upset by loud noise), as part of the Rivermead RPQ assessment.	0=0-Not experienced at all;1=1- No more of a problem;2=2- A mild problem;3=3- A moderate problem;4=4- A severe problem
RPQOther1	TRUE	FALSE	integer	This variable reflects if the subject, compared with before the accident, now (over the last 24 hours) suffers from Other difficulties than the pre-defined list, as part of the Rivermead RPQ assessment.	0=0-Not experienced at all;1=1-No more of a problem;2=2-A mild problem;3=3-A moderate problem;4=4-A severe problem
RPQOther1Text	TRUE	FALSE	text	This variable reflects which other difficulties, if the subject, compared with before the accident, now (over the last 24 hours) suffers from Other difficulties than the pre-defined list, as part of the Rivermead RPQ assessment.	Not Applicable
RPQOther2	TRUE	FALSE	integer	This variable reflects if the subject, compared with before the accident, now (over the last 24 hours) suffers from Other difficulties than the pre-defined list, as part of the Rivermead RPQ assessment.	0=0-Not experienced at all;1=1-No more of a problem;2=2-A mild problem;3=3-A moderate problem;4=4-A severe problem
RPQOther2Text	TRUE	FALSE	text	This variable reflects which other difficulties, if the subject, compared with before the accident, now (over the last 24 hours) suffers from Other difficulties than the pre-defined list, as part of the Rivermead RPQ assessment.	Not Applicable
RPQPoorConcentration	TRUE	FALSE	integer	This variable reflects if the subject, compared with before the accident, now (over the last 24 hours) suffers from Poor concentration, as part of the Rivermead RPQ assessment.	0=0-Not experienced at all;1=1- No more of a problem;2=2- A mild problem;3=3- A moderate problem;4=4- A severe problem
RPQQuestionnaireMode	TRUE	FALSE	integer	The mode in which the Rivermead RPQ assessment was performed; could be "Personal interview", "Postal questionnaire", "Telephone interview", "Web-based completion"	1=Telephone interview;2=Postal questionnaire;3=Web-based completion;4=Personal interview
RPQRestless	TRUE	FALSE	integer	This variable reflects if the subject, compared with before the accident, now (over the last 24 hours) suffers from Restlessness, as part of the Rivermead RPQ assessment.	0=0-Not experienced at all;1=1- No more of a problem;2=2- A mild problem;3=3- A moderate problem;4=4- A severe problem
RPQSleepDisturbance	TRUE	FALSE	integer	This variable reflects if the subject, compared with before the accident, now (over the last 24 hours) suffers from Sleep disturbance, as part of the Rivermead RPQ assessment.	0=0-Not experienced at all;1=1- No more of a problem;2=2- A mild problem;3=3- A moderate problem;4=4- A severe problem
RPQTotalScore	TRUE	FALSE	integer	RPQ (Rivermead post-concussion symptoms questionnaire) Total Score. Calculated centrally.	Not Applicable
SympSkullFract	TRUE	FALSE	integer	During neurological assessment at arrival in the ER, Clinical signs of skull base fracture (e.g. raccoon eyes, battle sign, hemotympanun, CSF otorrhea, CRF rhinorrhea, bleeding from ear) were recorded.	0=No;1=Yes;88=Unknown
SympVomiting	TRUE	FALSE	integer	During neurological assessment at arrival in the ER, Vomiting was recorded.	0=No;1=Once;2=More than once;88=Unknown

Brain imaging reports

Name	Static	Intervention	Format	Description	Possible Values
AnyIntracranTraumaticAbnormality	FALSE	FALSE	text	This variable was assessed by a central review panel, according the TBI-Common Data Elements. This variable indicates whether any of the 12 following CDEs is present (Mass lesion, ExtraaxialHematoma, EpiduralHematoma, SubduralHematomaAcute, SubduralHematomaSubacuteChronic, SubduralCollectionMixedDensity, Contusion, TAI, traumaticSubarachnoidHemorrhage, IntraventricularHemorrhage, MidlineShift or CisternalCompression.	absent=Absent;present=Present;indeteminate=Indeterminate;uninterpretable=Uninterpretable
CisternalCompression	FALSE	FALSE	text	This variable was assessed by a central review panel, according the TBI-Common Data Elements. This variable indicates whether there is compression of one or more the basal cisterns (i.e. suprasellar, quadrigeminal, prepontine, ambient, cisterna magna). More descriptive information (location, compression vs. absence) can be found in the JSON files.	absent=Absent;present=Present;indeteminate=Indeterminate;uninterpretable=Uninterpretable
Contusion	FALSE	FALSE	text	This variable was assessed by a central review panel, according the TBI-Common Data Elements. This variable indicates whether one or more contusions are present. More descriptive information (location, volume, number) and advanced information (e.g. hemorrhagic, non-hemorrhagic, intracerebral hemorrhage, etc.) can be found in the JSON files. Volume was estimated using the AxBxC/2 method. Edema was included in the measurements.	absent=Absent;present=Present;indeteminate=Indeterminate;uninterpretable=Uninterpretable
CRFCTAngulation	FALSE	FALSE	integer	This variable describes if a CT scan is performed with or without angulation. There are three options: no angulation (volume scan), orbital-meatal line and other. There is a risk of different interpretation, since there was no definition.	1=No angulation (volume scan);2=Orbital-meatal line;99=Other
CRFCTManuf	FALSE	FALSE	text	This variable describes the CT scans manufacturer.	99=Other;AGFA=Agfa;CARE=Carestream;GE=GE;HITA=Hitachi;KONI=Konica Minolta;PHIL=Philips;SIEM=Siemens;TOSH=Toshiba
CRFCTMidlineShift	FALSE	FALSE	integer	CT parameters scored by the investigator: reflects if there was midline shift on the CT.	0=No;1=Yes
CRFCTMidlineShiftMeasure	FALSE	FALSE	decimal	CT parameters scored by the investigator: reflects the volume in mm if there was a midline shift on the CT Check also "Imaging.CRFCTMidlineShift"	Not Applicable
CRFCTReason	FALSE	FALSE	text	This variable contains the main reason why a CT-scan, during hospital stay, was performed. One of following options: standard follow-up, post-operative control, clinical deterioration, (suspicion of) increasing ICP, lack of improvement, unknown, other (specified in CTMRI.CTReasonOther) The reason for making an early CT-scan/ER scan can be found in: CTMRI.CTERReason	CD=Clinical deterioration;ICUADM88=Unknown;ICUADM99=Other;IICP=(Suspicion of) Increasing ICP;LOP=Lack of improvement;POC=Post-operative control;SFU=Standard follow-up
CRFCTScannerType	FALSE	FALSE	integer	This variable specifies the type of CT-scanner by the number of slices.	16=16-slice;32=32-slice;64=64-slice;99=Other;128=128-slice;256=256-slice;320=320-slice
CRFCTTypeCCT	FALSE	FALSE	integer	This variable describes the type of CT scan that has been made. Multiple options can be selected from: Non-contrast CT, Contrast CT, CT Angiography, Perfusion CT.	0=No;1=Yes
CRFCTTypeCTA	FALSE	FALSE	integer	This variable describes the type of CT scan that has been made. Multiple options can be selected from: Non-contrast CT, Contrast CT, CT Angiography, Perfusion CT.	0=No;1=Yes
CRFCTTypeNCCT	FALSE	FALSE	integer	This variable describes the type of CT scan that has been made. Multiple options can be selected from: Non-contrast CT, Contrast CT, CT Angiography, Perfusion CT.	0=No;1=Yes

Name	Static	Intervention	Format	Description	Possible Values
CRFCTTypePCT	FALSE	FALSE	integer	This variable describes the type of CT scan that has been made. Multiple options can be selected from: Non-contrast CT, Contrast CT, CT Angiography, Perfusion CT.	0=No;1=Yes
CRFForm	FALSE	FALSE	text	Acute or follow up form in the e-CRF.	CTMRI=CT/MRI;FollowUp=Follow-up
CRFMRIManuf	FALSE	FALSE	text	This variable describes the MRI-scan manufacturer. This variable combines data from CTMRI.MRIManuf and FollowUp.MRIManuf	99=Other;GE=GE;PHIL=Philips;SIEM=Siemens;TOSH=Toshiba
CRFMRIReason	FALSE	FALSE	text	This variable contains the main reason why an MRI was performed. This variable combines data from CTMRI.MRIReason and FollowUp.MRIReason	88=Unknown;99=Other;ICUADM1=Discrepancy between CT and clinical condition;ICUADM2=Standard Care;ICUADM3=Detection of brainstem lesions;STUDYPROT=Study protocol
CRFMRIReasonOther	FALSE	FALSE	text	This variable contains the main reason why an MRI was performed, when in the variable "MRIReason", the option "other" was chosen. This is a text field. This variable combines data from CTMRI.MRIReasonOther and FollowUp.MRIReasonOther	Not Applicable
CRFMRIResultPreExistAbnorm	FALSE	FALSE	integer	This variable describes whether or not there are pre-existing abnormalities present on MRI scan (yes, no, unknown). Assessment by investigator and or physician. This variable combines data from CTMRI.MRIResultPreExistAbnorm and FollowUp.MRIResultPreExistAbnorm	0=No;1=Yes;88=Unknown
CRFMRIResultTraumaticAbnorm	FALSE	FALSE	integer	This variable describes whether or not there are traumatic abnormalities present on MRI scan (yes, no, unknown). Assessment by investigator and/or physician. This variable combines data from CTMRI.MRIResultTraumaticAbnorm and FollowUp.MRIResultTraumaticAbnorm	0=No;1=Yes;88=Unknown
CRFMRIScannerStrength	FALSE	FALSE	text	This variable describes the MRI scanner strength. This is a text field. This variable combines data from CTMRI.MRIScannerStrength and FollowUp.MRIScannerStrength	Not Applicable
CRFMRISequences99	FALSE	FALSE	integer	This variable describes the MRI sequence. Options are: T1, T2, FLAIR, DWI, GRE, SWI, DTI, MRSI, PWI, Other. Multiple options can be selected. This variable combines data from CTMRI.MRISequences and FollowUp.MRISequences	0=No;1=Yes
CRFMRISequencesDTI	FALSE	FALSE	integer	This variable describes the MRI sequence. Options are: T1, T2, FLAIR, DWI, GRE, SWI, DTI, MRSI, PWI, Other. Multiple options can be selected. This variable combines data from CTMRI.MRISequences and FollowUp.MRISequences	0=No;1=Yes
CRFMRISequencesDWI	FALSE	FALSE	integer	This variable describes the MRI sequence. Options are: T1, T2, FLAIR, DWI, GRE, SWI, DTI, MRSI, PWI, Other. Multiple options can be selected. This variable combines data from CTMRI.MRISequences and FollowUp.MRISequences	0=No;1=Yes
CRFMRISequencesFLAIR	FALSE	FALSE	integer	This variable describes the MRI sequence. Options are: T1, T2, FLAIR, DWI, GRE, SWI, DTI, MRSI, PWI, Other. Multiple options can be selected. This variable combines data from CTMRI.MRISequences and FollowUp.MRISequences	0=No;1=Yes
CRFMRISequencesGRE	FALSE	FALSE	integer	This variable describes the MRI sequence. Options are: T1, T2, FLAIR, DWI, GRE, SWI, DTI, MRSI, PWI, Other. Multiple options can be selected. This variable combines data from CTMRI.MRISequences and FollowUp.MRISequences	0=No;1=Yes
CRFMRISequencesMRSI	FALSE	FALSE	integer	This variable describes the MRI sequence. Options are: T1, T2, FLAIR, DWI, GRE, SWI, DTI, MRSI, PWI, Other. Multiple options can be selected. This variable	0=No;1=Yes

Name	Static	Intervention	Format	Description	Possible Values
				combines data from CTMRI.MRISequences and FollowUp.MRISequences	
CRFMRISequencesPWI	FALSE	FALSE	integer	This variable describes the MRI sequence. Options are: T1, T2, FLAIR, DWI, GRE, SWI, DTI, MRSI, PWI, Other. Multiple options can be selected. This variable combines data from CTMRI.MRISequences and FollowUp.MRISequences	0=No;1=Yes
CRFMRISequencesSWI	FALSE	FALSE	integer	This variable describes the MRI sequence. Options are: T1, T2, FLAIR, DWI, GRE, SWI, DTI, MRSI, PWI, Other. Multiple options can be selected. This variable combines data from CTMRI.MRISequences and FollowUp.MRISequences	0=No;1=Yes
CRFMRISequencesT1	FALSE	FALSE	integer	This variable describes the MRI sequence. Options are: T1, T2, FLAIR, DWI, GRE, SWI, DTI, MRSI, PWI, Other. Multiple options can be selected. This variable combines data from CTMRI.MRISequences and FollowUp.MRISequences	0=No;1=Yes
CRFMRISequencesT2	FALSE	FALSE	integer	This variable describes the MRI sequence. Options are: T1, T2, FLAIR, DWI, GRE, SWI, DTI, MRSI, PWI, Other. Multiple options can be selected. This variable combines data from CTMRI.MRISequences and FollowUp.MRISequences	0=No;1=Yes
CRFMRITraumAbnormASDH	FALSE	FALSE	integer	This variable describes whether or not there is an acute subdural hematoma present on MRI scan (yes, no, unknown). Assessment by investigator and/or physician. This variable combines data from CTMRI.MRITraumAbnormASDH and FollowUp.MRITraumAbnormASDH	0=No;1=Yes;88=Unknown
CRFMRITraumAbnormContusion	FALSE	FALSE	integer	This variable describes whether or not there is a contusion present on MRI scan (yes, no, unknown). Assessment by investigator and/or physician. This variable combines data from CTMRI.MRITraumAbnormContusion and FollowUp.MRITraumAbnormContusion	0=No;1=Yes;88=Unknown
CRFMRITraumAbnormDAI	FALSE	FALSE	integer	This variable describes whether or not there is DAI (diffuse axonal injury) present on MRI scan (yes, no, unknown). Assessment by investigator and/or physician. This variable combines data from CTMRI.MRITraumAbnormDAI and FollowUp.MRITraumAbnormDAI	0=No;1=Yes;88=Unknown
CRFMRITraumAbnormDAILesionLocBrainstem	FALSE	FALSE	integer	This variable describes whether or not there is a brain stem lesion present on MRI scan (yes, no, unknown). Assessment by investigator and/or physician. This variable combines data from CTMRI.MRITraumAbnormDAILesionLocBrainstem and FollowUp.MRITraumAbnormDAILesionLocBrainstem	0=No;1=Yes;88=Unknown
CRFMRITraumAbnormDAILesionLocCorpusCallosum	FALSE	FALSE	integer	This variable describes whether or not there is a corpus callosum lesion present on MRI scan (yes, no, unknown). Assessment by investigator and/or physician. This variable combines data from CTMRI.MRITraumAbnormDAILesionLocCorpusCallosum and FollowUp.MRITraumAbnormDAILesionLocCorpusCallosum	0=No;1=Yes;88=Unknown
CRFMRITraumAbnormDAILesionLocDiffuseWhiteMatter	FALSE	FALSE	integer	This variable describes whether or not there is a lesion in diffuse white matter present on MRI scan (yes, no, unknown). Assessment by investigator and/or physician. This variable combines data from CTMRI.MRITraumAbnormDAILesionLocDiffuseWhiteMatter and FollowUp.MRITraumAbnormDAILesionLocDiffuseWhiteMatter	0=No;1=Yes;88=Unknown

Name	Static	Intervention	Format	Description	Possible Values
CRFMRITraumAbnormDAINumLesions	FALSE	FALSE	integer	This variable describes how many DAI lesions are present on MRI scan (1,2,3,4,>=5). Assessment by investigator and/or physician. This variable combines data from CTMRI.MRITraumAbnormDAINumLesions and FollowUp.MRITraumAbnormDAINumLesions	1=1;2=2;3=3;4=4;5=>= 5
CRFMRITraumAbnormEDH	FALSE	FALSE	integer	This variable describes whether or not there is an epidural hematoma present on MRI scan (yes, no, unknown). Assessment by investigator and/or physician. This variable combines data from CTMRI.MRITraumAbnormEDH and FollowUp.MRITraumAbnormEDH	0=No;1=Yes;88=Unknown
CRFMRITypeMRA	FALSE	FALSE	integer	This variable describes the type of MRI scan that has been made. Options are: MRI, MRA This variable combines data from CTMRI.MRIType and FollowUp.MRIType	0=No;1=Yes
CRFMRITypeMRI	FALSE	FALSE	integer	This variable describes the type of MRI scan that has been made. Options are: MRI, MRA This variable combines data from CTMRI.MRIType and FollowUp.MRIType	0=No;1=Yes
CTAcuteSubdurHema	FALSE	FALSE	integer	Assessment by clinician/investigator whether or not in his/her interpretation an acute subdural hematoma is present. Also, the size (not quantified) is requested to be estimated.	0=No;1=Small;2=Large (mass);88=Unknown
CTBasalCisternsAbsentCompressed	FALSE	FALSE	integer	Assessment by clinician/investigator whether or not in his/her interpretation the basal cisterns are compressed.	0=No;1=Yes
CTContusion	FALSE	FALSE	integer	Assessment by clinician/investigator whether or not in his/her interpretation an intracerebral hematoma/contusion is present. Also, the size (not quantified) is requested to be estimated.	0=No;1=Small;2=Large (mass);88=Unknown
CTDeprSkullFract	FALSE	FALSE	integer	Assessment by clinician/investigator whether or not in his/her interpretation a depressed skull fracture is present. In addition, when present, clinician/investigator has to document whether the fracture is associated with an open wound or not (compound vs closed)	0=No;1=Closed;2=Open (compound)
CTExtraduralHema	FALSE	FALSE	integer	Assessment by clinician/investigator whether or not in his/her interpretation an acute extradural/epidural hematoma is present. Also, the size (not quantified) is requested to be estimated.	0=No;1=Small;2=Large (mass);88=Unknown
CTFrames	FALSE	FALSE			Not Applicable
CTICLesionDAI	FALSE	FALSE	integer	Assessment by clinician/investigator whether or not in his/her interpretation diffuse axonal injury is present.	0=No;1=Yes;88=Unknown
CTIschemia	FALSE	FALSE	integer	Assessment by clinician/investigator whether or not in his/her interpretation ischemia is present. Only applicable for clinical follow-up CT, not applicable to initial CT. In addition, the severity, in terms of how many arterial territories have ischemia, is requested to be answered.	0=No;1=Single arterial territory;2=Multiple territories;3=Hemisphere
CTNoOpMotiv	FALSE	FALSE	integer	WHY question: documents reason for not having an indication for (intra)cranial surgery.	0=No surgical lesion;1=Lesion present, but Acceptable/good neurologic condition;2=Lesion present, but Guideline adherence;3=Lesion present, but Little/no mass effect;4=Lesion present, but Not hospital policy;5=Lesion present, but Extremely poor prognosis;6=Lesion present, but Brain Death;7=Lesion present, but Old age;8=Lesion present, but Wish family;88=Unknown;99=Lesion present, but Other
CTNoOpMotivOther	FALSE	FALSE	text	Specification, only applicable if "CTMRI.CTNoOpMotiv" was "other"	Not Applicable
CTSchedForOp	FALSE	FALSE	integer	Whether or not the patient is scheduled for (intra)cranial surgery. The main aim here is to capture whether the clinical team taking	0=No;1=Yes

Name	Static	Intervention	Format	Description	Possible Values
				care of the patient sees a neurosurgical indication.	
CTSubarachnoidHem	FALSE	FALSE	integer	Assessment by clinician/investigator whether or not in his/her interpretation subarachnoid hemorrhage is present. In addition, the location of the hemorrhage is requested to be answered.	0=No;1=Basal;2=Cortical;3=Basal and Cortical
CTYesOpMotiv	FALSE	FALSE	integer	WHY question: documents reason for having an indication for (intra)cranial surgery.	1=Emergency/life saving;2=Clinical deterioration;3=Mass effect on CT;4=Radiological progression;5=(Suspicion of) raised ICP;6=Guideline adherence;7=To prevent deterioration;8=Depressed skull fracture;99=Other
CTYesOpMotivOther	FALSE	FALSE	text	Free text if "CTMRI.CTYesOpMotiv" was marked as 'Other'. Relates to the WHY question: documents reason for having an indication for (intra)cranial surgery.	Not Applicable
EpiduralHematoma	FALSE	FALSE	text	This variable was assessed by a central review panel, according the TBI-Common Data Elements. This variable indicates whether an epidural hematoma or multiple epidural hematomas are present. More descriptive information (location, volume, number) and advanced information (e.g. arterial versus venous) can be found in the JSON files. Note: Volume was estimated using the AxBxC/2 method. In the JSON files, "descriptive_volume" is the estimated volume of the lesion and can be used for analysis, "descriptive_length", "descriptive_width", "descriptive_max_thickness" are measurements that should not be used in any analysis.	absent=Absent;present=Present;indeteminate=Indeterminate;uninterpretable=Uninterpretable
ERAnyIntracranTraumaticAbnormality	TRUE	FALSE	text	In emergency room: This variable was assessed by a central review panel, according the TBI-Common Data Elements. This variable indicates whether any of the 12 following CDEs is present (Mass lesion, ExtraaxialHematoma, EpiduralHematoma, SubduralHematomaAcute, SubduralHematomaSubacuteChronic, SubduralCollectionMixedDensity, Contusion, TAI, traumaticSubarachnoidHemorrhage, IntraventricularHemorrhage, MidlineShift or CisternalCompression.	absent=Absent;present=Present;indeteminate=Indeterminate;uninterpretable=Uninterpretable
ERCisternalCompression	TRUE	FALSE	text	In emergency room: This variable was assessed by a central review panel, according the TBI-Common Data Elements. This variable indicates whether there is compression of one or more the basal cisterns (i.e. suprasellar, quadrigeminal, prepontine, ambient, cisterna magna). More descriptive information (location, compression vs. absence) can be found in the JSON files.	absent=Absent;present=Present;indeteminate=Indeterminate;uninterpretable=Uninterpretable
ERContusion	TRUE	FALSE	text	In emergency room: This variable was assessed by a central review panel, according the TBI-Common Data Elements. This variable indicates whether one or more contusions are present. More descriptive information (location, volume, number) and advanced information (e.g. hemorrhagic, non-hemorrhagic, intracerebral hemorrhage, etc.) can be found in the JSON files. Volume was estimated using the AxBxC/2 method. Edema was included in the measurements.	absent=Absent;present=Present;indeteminate=Indeterminate;uninterpretable=Uninterpretable
ERCRFCTAngulation	TRUE	FALSE	integer	In emergency room: This variable describes if a CT scan is performed with or without angulation. There are three options: no angulation (volume scan), orbital-meatal line and other. There is a risk of different interpretation, since there was no definition.	1=No angulation (volume scan);2=Orbital-meatal line;99=Other

Name	Static	Intervention	Format	Description	Possible Values
ERCRFCTManuf	TRUE	FALSE	text	In emergency room: This variable describes the CT scans manufacturer.	99=Other;AGFA=Agfa;CARE=Carestream;GE=GE;HITA=Hitachi;KONI=Konica Minolta;PHIL=Philips;SIEM=Siemens;TOSH=Toshiba
ERCRFCTMidlineShift	TRUE	FALSE	integer	In emergency room: CT parameters scored by the investigator: reflects if there was midline shift on the CT.	0=No;1=Yes
ERCRFCTMidlineShiftMeasure	TRUE	FALSE	decimal	In emergency room: CT parameters scored by the investigator: reflects the volume in mm if there was a midline shift on the CT Check also "Imaging.CRFCTMidlineShift"	Not Applicable
ERCRFCTScannerType	TRUE	FALSE	integer	In emergency room: This variable specifies the type of CT-scanner by the number of slices.	16=16-slice;32=32-slice;64=64-slice;99=Other;128=128-slice;256=256-slice;320=320-slice
ERCRFCTTypeCCT	TRUE	FALSE	integer	In emergency room: This variable describes the type of CT scan that has been made. Multiple options can be selected from: Non-contrast CT, Contrast CT, CT Angiography, Perfusion CT.	0=No;1=Yes
ERCRFCTTypeCTA	TRUE	FALSE	integer	In emergency room: This variable describes the type of CT scan that has been made. Multiple options can be selected from: Non-contrast CT, Contrast CT, CT Angiography, Perfusion CT.	0=No;1=Yes
ERCRFCTTypeNCCT	TRUE	FALSE	integer	In emergency room: This variable describes the type of CT scan that has been made. Multiple options can be selected from: Non-contrast CT, Contrast CT, CT Angiography, Perfusion CT.	0=No;1=Yes
ERCRFCTTypePCT	TRUE	FALSE	integer	In emergency room: This variable describes the type of CT scan that has been made. Multiple options can be selected from: Non-contrast CT, Contrast CT, CT Angiography, Perfusion CT.	0=No;1=Yes
ERCRFForm	TRUE	FALSE	text	In emergency room: Acute or follow up form in the e-CRF.	CTMRI=CT/MRI;FollowUp=Follow-up
ERCRFMRIManuf	TRUE	FALSE	text	In emergency room: This variable describes the MRI-scan manufacturer. This variable combines data from CTMRI.MRIManuf and FollowUp.MRIManuf	99=Other;GE=GE;PHIL=Philips;SIEM=Siemens;TOSH=Toshiba
ERCRFMRIResultPreExistAbnorm	TRUE	FALSE	integer	In emergency room: This variable describes whether or not there are pre-existing abnormalities present on MRI scan (yes, no, unknown). Assessment by investigator and or physician. This variable combines data from CTMRI.MRIResultPreExistAbnorm and FollowUp.MRIResultPreExistAbnorm	0=No;1=Yes;88=Unknown
ERCRFMRIResultTraumaticAbnorm	TRUE	FALSE	integer	In emergency room: This variable describes whether or not there are traumatic abnormalities present on MRI scan (yes, no, unknown). Assessment by investigator and/or physician. This variable combines data from CTMRI.MRIResultTraumaticAbnorm and FollowUp.MRIResultTraumaticAbnorm	0=No;1=Yes;88=Unknown
ERCRFMRIScannerStrength	TRUE	FALSE	text	In emergency room: This variable describes the MRI scanner strength. This is a text field. This variable combines data from CTMRI.MRIScannerStrength and FollowUp.MRIScannerStrength	Not Applicable
ERCRFMRISequences99	TRUE	FALSE	integer	In emergency room: This variable describes the MRI sequence. Options are: T1, T2, FLAIR, DWI, GRE, SWI, DTI, MRSI, PWI, Other. Multiple options can be selected. This variable combines data from CTMRI.MRISequences and FollowUp.MRISequences	0=No;1=Yes
ERCRFMRISequencesDTI	TRUE	FALSE	integer	In emergency room: This variable describes the MRI sequence. Options are: T1, T2, FLAIR, DWI, GRE, SWI, DTI, MRSI, PWI, Other. Multiple options can be selected. This variable combines data from CTMRI.MRISequences and FollowUp.MRISequences	0=No;1=Yes

Name	Static	Intervention	Format	Description	Possible Values
ERCRFMRISequencesDWI	TRUE	FALSE	integer	In emergency room: This variable describes the MRI sequence. Options are: T1, T2, FLAIR, DWI, GRE, SWI, DTI, MRSI, PWI, Other. Multiple options can be selected. This variable combines data from CTMRI.MRISequences and FollowUp.MRISequences	0=No;1=Yes
ERCRFMRISequencesFLAIR	TRUE	FALSE	integer	In emergency room: This variable describes the MRI sequence. Options are: T1, T2, FLAIR, DWI, GRE, SWI, DTI, MRSI, PWI, Other. Multiple options can be selected. This variable combines data from CTMRI.MRISequences and FollowUp.MRISequences	0=No;1=Yes
ERCRFMRISequencesGRE	TRUE	FALSE	integer	In emergency room: This variable describes the MRI sequence. Options are: T1, T2, FLAIR, DWI, GRE, SWI, DTI, MRSI, PWI, Other. Multiple options can be selected. This variable combines data from CTMRI.MRISequences and FollowUp.MRISequences	0=No;1=Yes
ERCRFMRISequencesMRSI	TRUE	FALSE	integer	In emergency room: This variable describes the MRI sequence. Options are: T1, T2, FLAIR, DWI, GRE, SWI, DTI, MRSI, PWI, Other. Multiple options can be selected. This variable combines data from CTMRI.MRISequences and FollowUp.MRISequences	0=No;1=Yes
ERCRFMRISequencesPWI	TRUE	FALSE	integer	In emergency room: This variable describes the MRI sequence. Options are: T1, T2, FLAIR, DWI, GRE, SWI, DTI, MRSI, PWI, Other. Multiple options can be selected. This variable combines data from CTMRI.MRISequences and FollowUp.MRISequences	0=No;1=Yes
ERCRFMRISequencesSWI	TRUE	FALSE	integer	In emergency room: This variable describes the MRI sequence. Options are: T1, T2, FLAIR, DWI, GRE, SWI, DTI, MRSI, PWI, Other. Multiple options can be selected. This variable combines data from CTMRI.MRISequences and FollowUp.MRISequences	0=No;1=Yes
ERCRFMRISequencesT1	TRUE	FALSE	integer	In emergency room: This variable describes the MRI sequence. Options are: T1, T2, FLAIR, DWI, GRE, SWI, DTI, MRSI, PWI, Other. Multiple options can be selected. This variable combines data from CTMRI.MRISequences and FollowUp.MRISequences	0=No;1=Yes
ERCRFMRISequencesT2	TRUE	FALSE	integer	In emergency room: This variable describes the MRI sequence. Options are: T1, T2, FLAIR, DWI, GRE, SWI, DTI, MRSI, PWI, Other. Multiple options can be selected. This variable combines data from CTMRI.MRISequences and FollowUp.MRISequences	0=No;1=Yes
ERCRFMRITraumAbnormASDH	TRUE	FALSE	integer	In emergency room: This variable describes whether or not there is an acute subdural hematoma present on MRI scan (yes, no, unknown). Assessment by investigator and/or physician. This variable combines data from CTMRI.MRITraumAbnormASDH and FollowUp.MRITraumAbnormASDH	0=No;1=Yes;88=Unknown
ERCRFMRITraumAbnormContusion	TRUE	FALSE	integer	In emergency room: This variable describes whether or not there is a contusion present on MRI scan (yes, no, unknown). Assessment by investigator and/or physician. This variable combines data from CTMRI.MRITraumAbnormContusion and FollowUp.MRITraumAbnormContusion	0=No;1=Yes;88=Unknown
ERCRFMRITraumAbnormDAI	TRUE	FALSE	integer	In emergency room: This variable describes whether or not there is DAI (diffuse axonal injury) present on MRI scan (yes, no,	0=No;1=Yes;88=Unknown

Name	Static	Intervention	Format	Description	Possible Values
				unknown). Assessment by investigator and/or physician. This variable combines data from CTMRI.MRITraumAbnormDAI and FollowUp.MRITraumAbnormDAI	
ERCRFMRITraumAbnormDAILesionLocBrainstem	TRUE	FALSE	integer	In emergency room: This variable describes whether or not there is a brain stem lesion present on MRI scan (yes, no, unknown). Assessment by investigator and/or physician. This variable combines data from CTMRI.MRITraumAbnormDAILesionLocBrainstem and FollowUp.MRITraumAbnormDAILesionLocBrainstem	0=No;1=Yes;88=Unknown
ERCRFMRITraumAbnormDAILesionLocCorpusCallosum	TRUE	FALSE	integer	In emergency room: This variable describes whether or not there is a corpus callosum lesion present on MRI scan (yes, no, unknown). Assessment by investigator and/or physician. This variable combines data from CTMRI.MRITraumAbnormDAILesionLocCorpusCallosum and FollowUp.MRITraumAbnormDAILesionLocCorpusCallosum	0=No;1=Yes;88=Unknown
ERCRFMRITraumAbnormDAILesionLocDiffuseWhiteMatter	TRUE	FALSE	integer	In emergency room: This variable describes whether or not there is a lesion in diffuse white matter present on MRI scan (yes, no, unknown). Assessment by investigator and/or physician. This variable combines data from CTMRI.MRITraumAbnormDAILesionLocDiffuseWhiteMatter and FollowUp.MRITraumAbnormDAILesionLocDiffuseWhiteMatter	0=No;1=Yes;88=Unknown
ERCRFMRITraumAbnormDAINumLesions	TRUE	FALSE	integer	In emergency room: This variable describes how many DAI lesions are present on MRI scan (1,2,3,4,>=5). Assessment by investigator and/or physician. This variable combines data from CTMRI.MRITraumAbnormDAINumLesions and FollowUp.MRITraumAbnormDAINumLesions	1=1;2=2;3=3;4=4;5=>= 5
ERCRFMRITraumAbnormEDH	TRUE	FALSE	integer	In emergency room: This variable describes whether or not there is an epidural hematoma present on MRI scan (yes, no, unknown). Assessment by investigator and/or physician. This variable combines data from CTMRI.MRITraumAbnormEDH and FollowUp.MRITraumAbnormEDH	0=No;1=Yes;88=Unknown
ERCRFMRITypeMRA	TRUE	FALSE	integer	In emergency room: This variable describes the type of MRI scan that has been made. Options are: MRI, MRA This variable combines data from CTMRI.MRIType and FollowUp.MRIType	0=No;1=Yes
ERCRFMRITypeMRI	TRUE	FALSE	integer	In emergency room: This variable describes the type of MRI scan that has been made. Options are: MRI, MRA This variable combines data from CTMRI.MRIType and FollowUp.MRIType	0=No;1=Yes
ERCTAcuteSubdurHema	TRUE	FALSE	integer	In emergency room: Assessment by clinician/investigator whether or not in his/her interpretation an acute subdural hematoma is present. Also, the size (not quantified) is requested to be estimated.	0=No;1=Small;2=Large (mass);88=Unknown
ERCTBasalCisternsAbsentCompressed	TRUE	FALSE	integer	In emergency room: Assessment by clinician/investigator whether or not in his/her interpretation the basal cisterns are compressed.	0=No;1=Yes
ERCTContusion	TRUE	FALSE	integer	In emergency room: Assessment by clinician/investigator whether or not in his/her interpretation an intracerebral hematoma/contusion is present. Also, the size (not quantified) is requested to be estimated.	0=No;1=Small;2=Large (mass);88=Unknown

Name	Static	Intervention	Format	Description	Possible Values
ERCTDeprSkullFract	TRUE	FALSE	integer	In emergency room: Assessment by clinician/investigator whether or not in his/her interpretation a depressed skull fracture is present. In addition, when present, clinician/investigator has to document whether the fracture is associated with an open wound or not (compound vs closed)	0=No;1=Closed;2=Open (compound)
ERCTERReason1	TRUE	FALSE	integer	In emergency room: WHY question: reason for performing CT; only applicable to initial scan (presentation).	1=GCS <= 14; 2=GCS = 15 + risk factors; 3=Head wound; 4=Exclusion of abnormalities prior to discharge; 5=Suspicion of maxillofacial injury; 88=Unknown; 99=Other
ERCTERReason2	TRUE	FALSE	integer	In emergency room: WHY question: reason for performing CT; only applicable to initial scan (presentation).	1=GCS <= 14; 2=GCS = 15 + risk factors; 3=Head wound; 4=Exclusion of abnormalities prior to discharge; 5=Suspicion of maxillofacial injury; 88=Unknown; 99=Other
ERCTERReason3	TRUE	FALSE	integer	In emergency room: WHY question: reason for performing CT; only applicable to initial scan (presentation).	1=GCS <= 14; 2=GCS = 15 + risk factors; 3=Head wound; 4=Exclusion of abnormalities prior to discharge; 5=Suspicion of maxillofacial injury; 88=Unknown; 99=Other
ERCTERReason4	TRUE	FALSE	integer	In emergency room: WHY question: reason for performing CT; only applicable to initial scan (presentation).	1=GCS <= 14; 2=GCS = 15 + risk factors; 3=Head wound; 4=Exclusion of abnormalities prior to discharge; 5=Suspicion of maxillofacial injury; 88=Unknown; 99=Other
ERCTERReason5	TRUE	FALSE	integer	In emergency room: WHY question: reason for performing CT; only applicable to initial scan (presentation).	1=GCS <= 14; 2=GCS = 15 + risk factors; 3=Head wound; 4=Exclusion of abnormalities prior to discharge; 5=Suspicion of maxillofacial injury; 88=Unknown; 99=Other
ERCTERReason88	TRUE	FALSE	integer	In emergency room: WHY question: reason for performing CT; only applicable to initial scan (presentation).	1=GCS <= 14; 2=GCS = 15 + risk factors; 3=Head wound; 4=Exclusion of abnormalities prior to discharge; 5=Suspicion of maxillofacial injury; 88=Unknown; 99=Other
ERCTERReason99	TRUE	FALSE	integer	In emergency room: WHY question: reason for performing CT; only applicable to initial scan (presentation).	1=GCS <= 14; 2=GCS = 15 + risk factors; 3=Head wound; 4=Exclusion of abnormalities prior to discharge; 5=Suspicion of maxillofacial injury; 88=Unknown; 99=Other
ERCTERReasonOther	TRUE	FALSE	text	In emergency room: Specification, only applicable if "CTMRI.CTERReason" was "other"	Not Applicable
ERCTExtraduralHema	TRUE	FALSE	integer	In emergency room: Assessment by clinician/investigator whether or not in his/her interpretation an acute extradural/epidural hematoma is present. Also, the size (not quantified) is requested to be estimated.	0=No;1=Small;2=Large (mass);88=Unknown
ERCTFrames	TRUE	FALSE			Not Applicable
ERCTICLesionDAI	TRUE	FALSE	integer	In emergency room: Assessment by clinician/investigator whether or not in his/her interpretation diffuse axonal injury is present.	0=No;1=Yes;88=Unknown
ERCTImaging	TRUE	FALSE			Not Applicable
ERCTNoOpMotiv	TRUE	FALSE	integer	In emergency room: WHY question: documents reason for not having an indication for (intra)cranial surgery.	0=No surgical lesion;1=Lesion present, but Acceptable/good neurologic condition;2=Lesion present, but Guideline adherence;3=Lesion present, but Little/no mass effect;4=Lesion present, but Not hospital policy;5=Lesion present, but Extremely poor prognosis;6=Lesion present, but Brain Death;7=Lesion present, but Old age;8=Lesion present, but Wish family;88=Unknown;99=Lesion present, but Other
ERCTNoOpMotivOther	TRUE	FALSE	text	In emergency room: Specification, only applicable if "CTMRI.CTNoOpMotiv" was "other"	Not Applicable
ERCTRiskFactorsERAgeGreatrThanEqual60	TRUE	FALSE	integer	In emergency room: This variable describes the presence of risk factors (here: age greater than or equal to 60 years) for structural abnormalities on an early CT/MRI. Information for this variable was meant to be only entered for early CT, when CTMRI.CTERReason = "GCS = 15 + risk factors". However, due to the e-CRF lay-out, it will also be populated for patients with CTMRI.CTERReason is not "GCS = 15 + risk factors" or patients with an MRI.	0=No;1=Yes
ERCTRiskFactorsERAlterationOfConsc	TRUE	FALSE	integer	In emergency room: This variable describes the presence of risk factors (here: alteration of consciousness) for structural abnormalities on an initial/ER CT/MRI. Information for this variable was meant to be only entered for initial/ER CT, when CTMRI.CTERReason = "GCS = 15 + risk factors". However, due to the e-CRF lay-out, it will also be populated for patients with	0=No;1=Yes

Name	Static	Intervention	Format	Description	Possible Values
				CTMRI.CTERReason is not "GCS = 15 + risk factors" or patients with an MRI.	
ERCTRiskFactorsERAnticoagTx	TRUE	FALSE	integer	In emergency room: This variable describes the presence of risk factors (here: use of anticoagulant Tx) for structural abnormalities on an early CT/MRI. Information for this variable was meant to be only entered for early CT, when CTMRI.CTERReason = "GCS = 15 + risk factors". However due to the e-CRF lay-out, it will also be populated for patients with CTMRI.CTERReason is not "GCS = 15 + risk factors" or patients with an MRI.	0=No;1=Yes
ERCTRiskFactorsERAnyNeuroDef	TRUE	FALSE	integer	In emergency room: This variable describes the presence of risk factors (here: 'any neurological deficit') for structural abnormalities on an initial/ER CT/MRI. Information for this variable was meant to be only entered for initial/ER CT, when CTMRI.CTERReason = "GCS = 15 + risk factors". However due to the e-CRF lay-out, it will also be populated for patients with CTMRI.CTERReason is not "GCS = 15 + risk factors" or patients with an MRI.	0=No;1=Yes
ERCTRiskFactorsERClinSignsOfFractSkullBaseVault	TRUE	FALSE	integer	In emergency room: This variable describes the presence of risk factors (here: 'clinical signs of fracture skull base or vault') for structural abnormalities on an initial/ER CT/MRI. Information for this variable was meant to be only entered for initial/ER CT, when CTMRI.CTERReason = "GCS = 15+risk factors". However, due to the e-CRF lay-out, it will also be populated for patients with CTMRI.CTERReason is not "GCS = 15 + risk factors" or patients with an MRI.	0=No;1=Yes
ERCTRiskFactorsERContusionFace	TRUE	FALSE	integer	In emergency room: This variable describes the presence of risk factors (here: 'contusion of the face') for structural abnormalities on an early CT/MRI. Information for this variable was meant to be only entered for early CT, when CTMRI.CTERReason = "GCS = 15 + risk factors". However due to the e-CRF lay-out, it will also be populated for patients with CTMRI.CTERReason is not "GCS = 15 + risk factors" or patients with an MRI.	0=No;1=Yes
ERCTRiskFactorsERFallFromAnyElev	TRUE	FALSE	integer	In emergency room: This variable describes the presence of risk factors (here: fall from any elevation) for structural abnormalities on an early CT/MRI. Information for this variable was meant to be only entered for early CT, when CTMRI.CTERReason = "GCS = 15 + risk factors". However, due to the e-CRF lay-out, it will also be populated for patients with CTMRI.CTERReason is not "GCS = 15 + risk factors" or patients with an MRI.	0=No;1=Yes
ERCTRiskFactorsERHeadache	TRUE	FALSE	integer	In emergency room: This variable describes the presence of risk factors (here: headache) for structural abnormalities on an early CT/MRI. Information for this variable was meant to be only entered for early CT, when CTMRI.CTERReason = "GCS = 15 + risk factors". However, due to the e-CRF lay-out, it will also be populated for patients with CTMRI.CTERReason is not "GCS = 15 + risk factors" or patients with an MRI.	0=No;1=Yes
ERCTRiskFactorsERIntoxication	TRUE	FALSE	integer	In emergency room: This variable describes the presence of risk factors (here: intoxication) for structural abnormalities on an initial/ER CT/MRI. Information for this variable was meant to be only entered for initial/ER CT, when CTMRI.CTERReason = "GCS = 15+risk factors". However due to	0=No;1=Yes

Name	Static	Intervention	Format	Description	Possible Values
				the e-CRF lay-out, it will also be populated for patients with CTMRI.CTERReason is not "GCS = 15 + risk factors" or patients with an MRI.	
ERCTRiskFactorsERLOC	TRUE	FALSE	integer	In emergency room: This variable describes the presence of risk factors (here: loss of consciousness) for structural abnormalities on an initial/ER CT/MRI. Information for this variable was meant to be only entered for initial/ER CT, when CTMRI.CTERReason = "GCS = 15 + risk factors". However, due to the e-CRF lay-out, it will also be populated for patients with CTMRI.CTERReason is not "GCS = 15 + risk factors" or patients with an MRI.	0=No;1=Yes
ERCTRiskFactorsEROther	TRUE	FALSE	integer	In emergency room: This variable describes the presence of risk factors (other reason, not specified elsewhere) for structural abnormalities on an early CT/MRI. Information for this variable was meant to be only entered for early CT, when CTMRI.CTERReason = "GCS = 15 + risk factors". However, due to the e-CRF lay-out, it will also be populated for patients with CTMRI.CTERReason is not "GCS = 15 + risk factors" or patients with an MRI.	0=No;1=Yes
ERCTRiskFactorsEROtherTxt	TRUE	FALSE	text	In emergency room: This variable describes the presence of risk factors (other reason, not specified elsewhere: textfield) for structural abnormalities on an early CT/MRI. Information for this variable was meant to be only entered for early CT, when CTMRI.CTERReason = "GCS = 15 + risk factors". However, due to the e-CRF lay-out, it will also be populated for patients with CTMRI.CTERReason is not "GCS = 15 + risk factors" or patients with an MRI.	Not Applicable
ERCTRiskFactorsERPhysEvidTraumaHeadSkull	TRUE	FALSE	integer	In emergency room: This variable describes the presence of risk factors (here: physical evidence of trauma to head/skull) for structural abnormalities on an initial/ER CT/MRI. Information for this variable was meant to be only entered for initial/ER CT, when CTMRI.CTERReason = "GCS = 15 + risk factors". However, due to the e-CRF lay-out, it will also be populated for patients with CTMRI.CTERReason is not "GCS = 15 + risk factors" or patients with an MRI.	0=No;1=Yes
ERCTRiskFactorsERPTAGreatrThanEqual4hrs	TRUE	FALSE	integer	In emergency room: This variable describes the presence of risk factors (here: PTA >= 4 hours) for structural abnormalities on an initial/ER CT/MRI. Information for this variable was meant to be only entered for initial/ER CT, when CTMRI.CTERReason = "GCS = 15 + risk factors". However, due to the e-CRF lay-out, it will also be populated for patients with CTMRI.CTERReason is not "GCS = 15 + risk factors" or patients with an MRI.	0=No;1=Yes
ERCTRiskFactorsERSeizure	TRUE	FALSE	integer	In emergency room: This variable describes the presence of risk factors (here: seizure) for structural abnormalities on an initial/ER CT/MRI. Information for this variable was meant to be only entered for initial/ER CT, when CTMRI.CTERReason = "GCS = 15 + risk factors". However, due to the e-CRF lay-out, it will also be populated for patients with CTMRI.CTERReason is not "GCS = 15 + risk factors" or patients with an MRI.	0=No;1=Yes
ERCTRiskFactorsERSignsFacialFract	TRUE	FALSE	integer	In emergency room: This variable describes the presence of risk factors (here: signs of facial fracture) for structural abnormalities on an initial/ER CT/MRI. Information for this variable was meant to be only entered	0=No;1=Yes

Name	Static	Intervention	Format	Description	Possible Values
				for initial/ER CT, when CTMRI.CTERReason = "GCS = 15 + risk factors". However, due to the e-CRF lay-out, it will also be populated for patients with CTMRI.CTERReason is not "GCS = 15 + risk factors" or patients with an MRI.	
ERCTRiskFactorsERVomit	TRUE	FALSE	integer	In emergency room: This variable describes the presence of risk factors (here: vomiting) for structural abnormalities on an initial/ER CT/MRI. Information for this variable was meant to be only entered for initial/ER CT, when CTMRI.CTERReason = "GCS = 15 + risk factors". However, due to the e-CRF lay-out, it will also be populated for patients with CTMRI.CTERReason is not "GCS = 15 + risk factors" or patients with an MRI.	0=No;1=Yes
ERCTRiskFactorsERVulnRoadUser	TRUE	FALSE	integer	In emergency room: This variable describes the presence of risk factors (here: vulnerable road users, like pedestrians or cyclists) for structural abnormalities on an early CT/MRI. Information for this variable was meant to be only entered for early CT, when CTMRI.CTERReason = "GCS = 15 + risk factors". However, due to the e-CRF lay-out, it will also be populated for patients with CTMRI.CTERReason is not "GCS = 15 + risk factors" or patients with an MRI.	0=No;1=Yes
ERCTSchedForOp	TRUE	FALSE	integer	In emergency room: Whether or not the patient is scheduled for (intra)cranial surgery. The main aim here is to capture whether the clinical team taking care of the patient sees a neurosurgical indication.	0=No;1=Yes
ERCTSubarachnoidHem	TRUE	FALSE	integer	In emergency room: Assessment by clinician/investigator whether or not in his/her interpretation subarachnoid hemorrhage is present. In addition, the location of the hemorrhage is requested to be answered.	0=No;1=Basal;2=Cortical;3=Basal and Cortical
ERCTYesOpMotiv	TRUE	FALSE	integer	In emergency room: WHY question: documents reason for having an indication for (intra)cranial surgery.	1=Emergency/life saving;2=Clinical deterioration;3=Mass effect on CT;4=Radiological progression;5=(Suspicion of) raised ICP;6=Guideline adherence;7=To prevent deterioration;8=Depressed skull fracture;99=Other
ERCTYesOpMotivOther	TRUE	FALSE	text	In emergency room: Free text if "CTMRI.CTYesOpMotiv" was marked as 'Other'. Relates to the WHY question: documents reason for having an indication for (intra)cranial surgery.	Not Applicable
EREpiduralHematoma	TRUE	FALSE	text	In emergency room: This variable was assessed by a central review panel, according the TBI-Common Data Elements. This variable indicates whether an epidural hematoma or multiple epidural hematomas are present. More descriptive information (location, volume, number) and advanced information (e.g. arterial versus venous) can be found in the JSON files. Note: Volume was estimated using the AxBxC/2 method. In the JSON files, "descriptive_volume" is the estimated volume of the lesion and can be used for analysis, "descriptive_length", "descriptive_width", "descriptive_max_thickness" are measurements that should not be used in any analysis.	absent=Absent;present=Present;indeteminate=Indeterminate;uninterpretable=Uninterpretable
ERExtraaxialHematoma	TRUE	FALSE	text	In emergency room: This variable was assessed by a central review panel, according the TBI-Common Data Elements. This variable indicates whether an extra-axial hematoma or multiple extra-axial hematomas are present. This term was mostly used for bleedings that were difficult to classify or for bleedings that were evacuated (i.e. after craniectomy). More descriptive information (location, volume,	absent=Absent;present=Present;indeteminate=Indeterminate;uninterpretable=Uninterpretable

Name	Static	Intervention	Format	Description	Possible Values
				number) and advanced information (e.g. arterial versus venous) can be found in the JSON files. Note: Volume was estimated using the AxBxC/2 method. In the JSON files, "descriptive_volume" is the estimated volume of the lesion and can be used for analysis, "descriptive_length", "descriptive_width", "descriptive_max_thickness" are measurements that should not be used in any analysis.	
ERFisherClassification	TRUE	FALSE	integer	In emergency room: S: A CT grading scale for traumatic Subarachnoid Hemorrhage B: Useful in predicting the occurrence and severity of cerebral vasospasm. Ranges from 1-4 (No tSAH, no IVH (1), no IVH, trace or moderate tSAH (can be in multiple locations) (2), No IVH, full tSAH (3), IVH (4) R: Only available for CT scans. For each patient, the FisherClassification can be found in the Imaging.LesionData variable. For CENTER-TBI, only the older version of Fisher was scored, not the newer "modified" version.	0=grade 0;1=grade 1;2=grade 2;3=grade 3;4=grade 4
ERGreenCTGrade	TRUE	FALSE	integer	In emergency room: A CT grading scale for traumatic Subarachnoid Hemorrhage (tSAH). Useful for outcome prediction. Ranges from 1-4, with a subdivision of 3 into "31", "32" and 4 into "41" and "42". (thin tSAH (1), thick tSAH (2), thin tSAH with mass lesion and no MLS (31), thin tSAH with mass lesion and MLS (32), thick tSAH with mass lesion and no MLS (41), thick tSAH with mass lesion and MLS (42). Only available for CT scans. For each patient, the GreenCTgrade can be found in the Imaging.LesionData variable. Could only be filled out when tSAH was present.	Not Applicable
ERHelsinkiCTScore	TRUE	FALSE	integer	In emergency room: S: A general CT grading scale B: Useful for outcome prediction. Ranges from -3 to 14. Scoring is done as follows: subdural (+2), intracerebral hematoma (+2), epidural hematoma (-3), mass lesion size >25 cc (+2), IVH (+3), compressed cisterns (+1), obliterated cisterns (+5) R: Only available for CT scans. For each patient, the HelsinkiCTscore can be found in the Imaging.LesionData variable. Empty values for the HelsinkiCTscore stand for a normal CT scan! A score of 0 does not necessarily mean the patient had a normal CT. (e.g. epidural hematoma -3, + IVH = 3 = 0).	Not Applicable
ERIntraventricularHemorrhage	TRUE	FALSE	text	In emergency room: This variable was assessed by a central review panel, according the TBI-Common Data Elements. This variable indicates whether intraventricular blood is present. More descriptive information (location) can be found in the JSON files.	absent=Absent;present=Present;indeteminate=Indeterminate;uninterpretable=Uninterpretable
ERLesionData	TRUE	FALSE	text	In emergency room: This variable contains ALL lesion information as assessed by central review. There are 23 CDEs that have been evaluated for each patient and 6 classifications that have been filled out. For 13 CDEs and all 6 classifications separate variables have been made available. (see "imaging." variables: SkullFracture, Mass lesion, ExtraaxialHematoma, EpiduralHematoma, SubduralHematomaAcute, SubduralHematomaSubacuteChronic, SubduralCollectionMixedDensity, Contusion, TAI,	Not Applicable

Name	Static	Intervention	Format	Description	Possible Values
				traumaticSubarachnoidHemorrhage, IntraventricularHemorrhage, MidlineShift, CisternalCompression. A derived variable was also created with AnyIntracranTraumaticAbnormality). These 13 CDEs only contain basic information (i.e. lesion is present, absent, indeterminate, uninterpretable, not interpreted).	
ERMarshallCTClassification	TRUE	FALSE	integer	In emergency room: A general CT grading scale. Useful for outcome prediction. Ranges from 1 to 6 in our dataset. (No visible pathology on CT (1), Cisterns present, MLS < 5 mm (2), Cisterns compressed or absent, MLS < 5 mm (3), MLS > 5 mm, no mass lesion > 25 cc (4), Evacuated mass lesion (5), Non-evacuated mass lesion (6)) Only available for CT scans. For each patient, the MarshallCTClassification can be found in a Imaging.LesionData variable. For the Marshall CT classification the admission scan was scored. (which is not necessarily the "worst").	1=Diffuse injury I (no visible pathology);2=Diffuse injury II;3=Diffuse injury III (swelling);4=Diffuse injury IV (shift);5=Evacuated mass lesion V;6=Non-evacuated mass lesion VI
ERMassLesion	TRUE	FALSE	text	In emergency room: This variable was assessed by a central review panel, according the TBI-Common Data Elements. This variable indicates whether a mass lesion is present. "Mass Lesion" in this case was defined as a total brain lesion volume > 25 cc. Note that this can mean that there is at least one large lesion or multiple co-existing lesions (contusions, subdural hematomas, etc.) that add up to > 25 cc. More information can be found in the JSON files.	absent=Absent;present=Present;indeteminate=Indeterminate;uninterpretable=Uninterpretable
ERMidlineShift	TRUE	FALSE	text	In emergency room: This variable was assessed by a central review panel, according the TBI-Common Data Elements. This variable indicates whether a midline shift of > 5 mm is present. More descriptive information (direction) can be found in the JSON files.	absent=Absent;present=Present;indeteminate=Indeterminate;uninterpretable=Uninterpretable
ERMorrisMarshallClassification	TRUE	FALSE	integer	In emergency room: S: A CT grading scale for traumatic Subarachnoid Hemorrhage (tSAH) B: Useful for outcome prediction. Ranges from 0-4. (no tSAH (0), trace or moderate tSAH in one location (basal, cortical or tentorial) (1), one location full, or 2 not full (2), two locations full (3), 3 locations or more (4)) R: Only available for CT scans. For each patient, the MorrisMarshallClassification can be found in a Imaging.LesionData variable.	Not Applicable
ERMRFrames	TRUE	FALSE			Not Applicable
ERMRIERReason88	TRUE	FALSE	integer	In emergency room: This variable contains the main reason why an MRI, ultra-early MR (within 72 hrs), was performed. Possible options are ER only: Discrepancy between clinical symptomatology and (lack of) CT abnormalities; suspicion non¬≠metal foreign object; instead of CT (limiting radiation exposure); suspicion spinal cord lesion; unknown; other (further specified in text field CTMRI.MRIERReasonOther. Reasons for clinical MRI's can be found in variable CTMRI.MRIReason	1=ER only: Discrepancy between clinical symptomatology and (lack of) CT abnormalities; 2=ER only: Suspicion non-metal foreign object; 3=ER only: Instead of CT (limiting radiation exposure); 4=ER only: Suspicion spinal cord lesion; 88=Unknown; 99=Other
ERMRIERReason99	TRUE	FALSE	integer	In emergency room: This variable contains the main reason why an MRI, ultra-early MR (within 72 hrs), was performed. Possible options are ER only: Discrepancy between clinical symptomatology and (lack of) CT abnormalities; suspicion non¬≠metal foreign object; instead of CT (limiting radiation exposure); suspicion spinal cord lesion; unknown; other (further specified in	1=ER only: Discrepancy between clinical symptomatology and (lack of) CT abnormalities; 2=ER only: Suspicion non-metal foreign object; 3=ER only: Instead of CT (limiting radiation exposure); 4=ER only: Suspicion spinal cord lesion; 88=Unknown; 99=Other

Name	Static	Intervention	Format	Description	Possible Values
				text field CTMRI.MRIERReasonOther. Reasons for clinical MRI's can be found in variable CTMRI.MRIReason	
ERMRIERReasonER1	TRUE	FALSE	integer	In emergency room: This variable contains the main reason why an MRI, ultra-early MR (within 72 hrs), was performed. Possible options are ER only: Discrepancy between clinical symptomatology and (lack of) CT abnormalities; suspicion non¬≠metal foreign object; instead of CT (limiting radiation exposure); suspicion spinal cord lesion; unknown; other (further specified in text field CTMRI.MRIERReasonOther. Reasons for clinical MRI's can be found in variable CTMRI.MRIReason	1=ER only: Discrepancy between clinical symptomatology and (lack of) CT abnormalities; 2=ER only: Suspicion non-metal foreign object; 3=ER only: Instead of CT (limiting radiation exposure); 4=ER only: Suspicion spinal cord lesion; 88=Unknown; 99=Other
ERMRIERReasonER2	TRUE	FALSE	integer	In emergency room: This variable contains the main reason why an MRI, ultra-early MR (within 72 hrs), was performed. Possible options are ER only: Discrepancy between clinical symptomatology and (lack of) CT abnormalities; suspicion non¬≠metal foreign object; instead of CT (limiting radiation exposure); suspicion spinal cord lesion; unknown; other (further specified in text field CTMRI.MRIERReasonOther. Reasons for clinical MRI's can be found in variable CTMRI.MRIReason	1=ER only: Discrepancy between clinical symptomatology and (lack of) CT abnormalities; 2=ER only: Suspicion non-metal foreign object; 3=ER only: Instead of CT (limiting radiation exposure); 4=ER only: Suspicion spinal cord lesion; 88=Unknown; 99=Other
ERMRIERReasonER3	TRUE	FALSE	integer	In emergency room: This variable contains the main reason why an MRI, ultra-early MR (within 72 hrs), was performed. Possible options are ER only: Discrepancy between clinical symptomatology and (lack of) CT abnormalities; suspicion non¬≠metal foreign object; instead of CT (limiting radiation exposure); suspicion spinal cord lesion; unknown; other (further specified in text field CTMRI.MRIERReasonOther. Reasons for clinical MRI's can be found in variable CTMRI.MRIReason	1=ER only: Discrepancy between clinical symptomatology and (lack of) CT abnormalities; 2=ER only: Suspicion non-metal foreign object; 3=ER only: Instead of CT (limiting radiation exposure); 4=ER only: Suspicion spinal cord lesion; 88=Unknown; 99=Other
ERMRIERReasonER4	TRUE	FALSE	integer	In emergency room: This variable contains the main reason why an MRI, ultra-early MR (within 72 hrs), was performed. Possible options are ER only: Discrepancy between clinical symptomatology and (lack of) CT abnormalities; suspicion non¬≠metal foreign object; instead of CT (limiting radiation exposure); suspicion spinal cord lesion; unknown; other (further specified in text field CTMRI.MRIERReasonOther. Reasons for clinical MRI's can be found in variable CTMRI.MRIReason	1=ER only: Discrepancy between clinical symptomatology and (lack of) CT abnormalities; 2=ER only: Suspicion non-metal foreign object; 3=ER only: Instead of CT (limiting radiation exposure); 4=ER only: Suspicion spinal cord lesion; 88=Unknown; 99=Other
ERMRImaging	TRUE	FALSE			Not Applicable
ERRotterdamCTScore	TRUE	FALSE	integer	In emergency room: S: A general CT grading scale B: Useful for outcome prediction. Ranges from 1 to 6 in our dataset. Scoring was as follows: IVH or tSAH (+1), Epidural Mass not present (+1), MLS > 5 mm (+1), cisterns compressed (+1), cisterns absent (+1), SUM SCORE = +1 R: Only available for CT scans. For each patient, the RotterdamCTscore can be found in the Imaging.LesionData variable.	1=Diffuse injury I (no visible pathology);2=Diffuse injury II;3=Diffuse injury III (swelling);4=Diffuse injury IV (shift);5=Evacuated mass lesion V;6=Non-evacuated mass lesion VI
ERScanType	TRUE	FALSE	text	In emergency room: S: MR Mapping for series description B: Since all centers use different series description, a mapping for T2, T1, FLAIR, T2*, DTI and rs-fMRI is provided. R: For CT, ScanType and Series Description should be the same.	Not Applicable
ERSeriesDescription	TRUE	FALSE	text	In emergency room: S: Scan Series description retrieved from DICOM header A: Serie Name Given by the image acquisition center. R: DICOM Tag (0008,103E) Series Description	Not Applicable

Name	Static	Intervention	Format	Description	Possible Values
ERSkullFracture	TRUE	FALSE	text	In emergency room: This variable was assessed by a central review panel, according the TBI-Common Data Elements. This variable indicates whether a skull fracture or multiple skull fractures are present. More descriptive information (location, number) and advanced information (morphology: e.g. depressed, compound, etc.) can be found in the JSON files.	absent=Absent;present=Present;indeteminate=Indeterminate;uninterpretable=Uninterpretable
ERSubduralCollectionMixedDensity	TRUE	FALSE	text	In emergency room: This variable was assessed by a central review panel, according the TBI-Common Data Elements. This variable indicates whether one or multiple subdural collections/mixed density hematomas are present. More descriptive information (location, volume, number) and advanced information (e.g. isodensity, hypodensity, acute on chronic, chronic recurrent etc.) can be found in the JSON files. Volume was estimated using the AxBxC/2 method. Note: cfr. epidural and subdural hematoms regarding volumes. Note: In the JSON files length, width and depth of lesions can not be used as metrics in any analysis! Only "descriptive volume" is a valid metric (in cc)	absent=Absent;present=Present;indeteminate=Indeterminate;uninterpretable=Uninterpretable
ERSubduralHematomaAcute	TRUE	FALSE	text	In emergency room: This variable was assessed by a central review panel, according the TBI-Common Data Elements. This variable indicates whether an acute subdural hematoma or multiple acute subdural hematomas are present. More descriptive information (location, volume, number) and advanced information (homogeneous versus heterogeneous) can be found in the JSON files.Volume was estimated using the AxBxC/2 method, however, small traces of acute subdural blood on the tentorium or interhemispheric were not measured. Note: cfr. epidural: only "descriptive_volume" can be used for analysis. Note: In the JSON files length, width and depth of lesions can not be used as metrics in any analysis! Only "descriptive volume" is a valid metric (in cc)	absent=Absent;present=Present;indeteminate=Indeterminate;uninterpretable=Uninterpretable
ERSubduralHematomaSubacuteChronic	TRUE	FALSE	text	In emergency room: This variable was assessed by a central review panel, according the TBI-Common Data Elements. This variable indicates whether one or multiple subacute/chronic subdural hematoma(s) are present. More descriptive information (location, volume, number) can be found in the JSON files.Note: Volume was estimated using the AxBxC/2 method. Note: this variable is best considered together with the "SubduralCollectionMixedDensity" variable, as the central reviewer did not have information to time of injury. Many subacute/chronic subdurals were therefore categorised as "mixed density". Note: cfr. epidural and acute subdural hematoma regarding volumes. Note: In the JSON files length, width and depth of lesions can not be used as metrics in any analysis! Only "descriptive volume" is a valid metric (in cc)	absent=Absent;present=Present;indeteminate=Indeterminate;uninterpretable=Uninterpretable
ERTAI	TRUE	FALSE	text	In emergency room: This variable was assessed by a central review panel, according the TBI-Common Data Elements. This variable indicates whether axonal injury is present. More descriptive information (location, TAI versus DAI) can be found in the JSON files. Note: be aware	absent=Absent;present=Present;indeteminate=Indeterminate;uninterpretable=Uninterpretable

Name	Static	Intervention	Format	Description	Possible Values
				of the modality you are interested in (i.e. CT or MRI).	
ERTimepoint	TRUE	FALSE	text	In emergency room: Outcome timepoints are 2-3 weeks, 3 months, 6 months, 12 months and 24 months. Depending on the stratum and sub-studies of a patient, follow up was performed at some of these time points or at all timepoints. For an overview, please check the SOP Manual.	Clinical=Clinical;CT Early=CT Early;CT Followup=CT Follow-up;CT Post-Op=CT Post-Op;MR 12 months=MR 12 months;MR 2 weeks=MR 2 weeks;MR 24 months=MR 24 months;MR 3 months=MR 3 months;MR 6 months=MR 6 months;MR Early=MR Early
ERTraumaticSubarachnoidHemorrhage	TRUE	FALSE	text	In emergency room: This variable was assessed by a central review panel, according the TBI-Common Data Elements. This variable indicates whether traumatic subarachnoid blood is present. More descriptive information (location and amount (e.g. trace, moderate, severe)) can be found in the JSON files. * Location can be: cortical, basal, interhemispheric or tentorial.	absent=Absent;present=Present;indeteminate=Indeterminate;uninterpretable=Uninterpretable
ERWindowDetectionQuality	TRUE	FALSE	text	In emergency room: S: Automated Detection for bone versus brain. B: Possible options: Bone Window, Brain Window, Window Uncertainty R: Only available for CT scans. No brain window scans should be classified as bone window.	Not Applicable
ERXsiType	TRUE	FALSE	text	In emergency room: S: Type of imaging (CT/MR) A: Assess if the scan is CT or MR R: Possible values 'xnat:mrSessionData' and 'xnat:ctSessionData'	xnat:ctSessionData=xnat:ctSessionData;xnat:mrSessionData=xnat:mrSessionData
ExtraaxialHematoma	FALSE	FALSE	text	This variable was assessed by a central review panel, according the TBI-Common Data Elements. This variable indicates whether an extra-axial hematoma or multiple extra-axial hematomas are present. This term was mostly used for bleedings that were difficult to classify or for bleedings that were evacuated (i.e. after craniectomy). More descriptive information (location, volume, number) and advanced information (e.g. arterial versus venous) can be found in the JSON files. Note: Volume was estimated using the AxBxC/2 method. In the JSON files, "descriptive_volume" is the estimated volume of the lesion and can be used for analysis, "descriptive_length", "descriptive_width", "descriptive_max_thickness" are measurements that should not be used in any analysis.	absent=Absent;present=Present;indeteminate=Indeterminate;uninterpretable=Uninterpretable
FisherClassification	FALSE	FALSE	integer	S: A CT grading scale for traumatic Subarachnoid Hemorrhage B: Useful in predicting the occurrence and severity of cerebral vasospasm. Ranges from 1-4 (No tSAH, no IVH (1), no IVH, trace or moderate tSAH (can be in multiple locations) (2), No IVH, full tSAH (3), IVH (4) R: Only available for CT scans. For each patient, the FisherClassification can be found in the Imaging.LesionData variable. For CENTER-TBI, only the older version of Fisher was scored, not the newer "modified" version.	0=grade 0;1=grade 1;2=grade 2;3=grade 3;4=grade 4
GreenCTGrade	FALSE	FALSE	integer	A CT grading scale for traumatic Subarachnoid Hemorrhage (tSAH). Useful for outcome prediction. Ranges from 1-4, with a subdivision of 3 into "31", "32" and 4 into "41" and "42". (thin tSAH (1), thick tSAH (2), thin tSAH with mass lesion and no MLS (31), thin tSAH with mass lesion and MLS (32), thick tSAH with mass lesion and no MLS (41), thick tSAH with mass lesion and MLS (42). Only available for CT scans. For each patient, the GreenCTgrade can be found in the Imaging.LesionData	Not Applicable

Name	Static	Intervention	Format	Description	Possible Values
				variable. Could only be filled out when tSAH was present.	
HelsinkiCTScore	FALSE	FALSE	integer	S: A general CT grading scale B: Useful for outcome prediction. Ranges from -3 to 14. Scoring is done as follows: subdural (+2), intracerebral hematoma (+2), epidural hematoma (-3), mass lesion size >25 cc (+2), IVH (+3), compressed cisterns (+1), obliterated cisterns (+5) R: Only available for CT scans. For each patient, the HelsinkiCTscore can be found in the Imaging.LesionData variable. Empty values for the HelsinkiCTscore stand for a normal CT scan! A score of 0 does not necessarily mean the patient had a normal CT. (e.g. epidural hematoma -3, + IVH = 3 = 0).	Not Applicable
IntraventricularHemorrhage	FALSE	FALSE	text	This variable was assessed by a central review panel, according the TBI-Common Data Elements. This variable indicates whether intraventricular blood is present. More descriptive information (location) can be found in the JSON files.	absent=Absent;present=Present;indeteminate=Indeterminate;uninterpretable=Uninterpretable
LesionData	FALSE	FALSE	text	This variable contains ALL lesion information as assessed by central review. There are 23 CDEs that have been evaluated for each patient and 6 classifications that have been filled out. For 13 CDEs and all 6 classifications separate variables have been made available. (see "imaging." variables: SkullFracture, Mass lesion, ExtraaxialHematoma, EpiduralHematoma, SubduralHematomaAcute, SubduralHematomaSubacuteChronic, SubduralCollectionMixedDensity, Contusion, TAI, traumaticSubarachnoidHemorrhage, IntraventricularHemorrhage, MidlineShift, CisternalCompression. A derived variable was also created with AnyIntracranTraumaticAbnormality). These 13 CDEs only contain basic information (i.e. lesion is present, absent, indeterminate, uninterpretable, not interpreted).	Not Applicable
MarshallCTClassification	FALSE	FALSE	integer	A general CT grading scale. Useful for outcome prediction. Ranges from 1 to 6 in our dataset. (No visible pathology on CT (1), Cisterns present, MLS < 5 mm (2), Cisterns compressed or absent, MLS < 5 mm (3), MLS > 5 mm, no mass lesion > 25 cc (4), Evacuated mass lesion (5), Non-evacuated mass lesion (6)) Only available for CT scans. For each patient, the MarshallCTClassification can be found in a Imaging.LesionData variable. For the Marshall CT classification the admission scan was scored. (which is not necessarily the "worst").	1=Diffuse injury I (no visible pathology);2=Diffuse injury II;3=Diffuse injury III (swelling);4=Diffuse injury IV (shift);5=Evacuated mass lesion V;6=Non-evacuated mass lesion VI
MassLesion	FALSE	FALSE	text	This variable was assessed by a central review panel, according the TBI-Common Data Elements. This variable indicates whether a mass lesion is present. "Mass Lesion" in this case was defined as a total brain lesion volume > 25 cc. Note that this can mean that there is at least one large lesion or multiple co-existing lesions (contusions, subdural hematomas, etc.) that add up to > 25 cc. More information can be found in the JSON files.	absent=Absent;present=Present;indeteminate=Indeterminate;uninterpretable=Uninterpretable
MidlineShift	FALSE	FALSE	text	This variable was assessed by a central review panel, according the TBI-Common Data Elements. This variable indicates whether a midline shift of > 5 mm is present. More descriptive information (direction) can be found in the JSON files.	absent=Absent;present=Present;indeteminate=Indeterminate;uninterpretable=Uninterpretable

Name	Static	Intervention	Format	Description	Possible Values
MorrisMarshallClassification	FALSE	FALSE	integer	S: A CT grading scale for traumatic Subarachnoid Hemorrhage (tSAH) B: Useful for outcome prediction. Ranges from 0-4. (no tSAH (0), trace or moderate tSAH in one location (basal, cortical or tentorial) (1), one location full, or 2 not full (2), two locations full (3), 3 locations or more (4)) R: Only available for CT scans. For each patient, the MorrisMarshallClassification can be found in a Imaging.LesionData variable.	Not Applicable
MRFrames	FALSE	FALSE			Not Applicable
RotterdamCTScore	FALSE	FALSE	integer	S: A general CT grading scale B: Useful for outcome prediction. Ranges from 1 to 6 in our dataset. Scoring was as follows: IVH or tSAH (+1), Epidural Mass not present (+1), MLS > 5 mm (+1), cisterns compressed (+1), cisterns absent (+1), SUM SCORE = +1 R: Only available for CT scans. For each patient, the RotterdamCTscore can be found in the Imaging.LesionData variable.	1=Diffuse injury I (no visible pathology);2=Diffuse injury II;3=Diffuse injury III (swelling);4=Diffuse injury IV (shift);5=Evacuated mass lesion V;6=Non-evacuated mass lesion VI
ScanType	FALSE	FALSE	text	S: MR Mapping for series description B: Since all centers use different series description, a mapping for T2, T1, FLAIR, T2*, DTI and rs-fMRI is provided. R: For CT, ScanType and Series Description should be the same.	Not Applicable
SeriesDescription	FALSE	FALSE	text	S: Scan Series description retrieved from DICOM header A: Serie Name Given by the image acquisition center. R: DICOM Tag (0008,103E) Series Description	Not Applicable
SkullFracture	FALSE	FALSE	text	This variable was assessed by a central review panel, according the TBI-Common Data Elements. This variable indicates whether a skull fracture or multiple skull fractures are present. More descriptive information (location, number) and advanced information (morphology: e.g. depressed, compound, etc.) can be found in the JSON files.	absent=Absent;present=Present;indeteminate=Indeterminate;uninterpretable=Uninterpretable
SubduralCollectionMixedDensity	FALSE	FALSE	text	This variable was assessed by a central review panel, according the TBI-Common Data Elements. This variable indicates whether one or multiple subdural collections/mixed density hematomas are present. More descriptive information (location, volume, number) and advanced information (e.g. isodensity, hypodensity, acute on chronic, chronic recurrent etc.) can be found in the JSON files. Volume was estimated using the AxBxC/2 method. Note: cfr. epidural and subdural hematoms regarding volumes. Note: In the JSON files length, width and depth of lesions can not be used as metrics in any analysis! Only "descriptive volume" is a valid metric (in cc)	absent=Absent;present=Present;indeteminate=Indeterminate;uninterpretable=Uninterpretable
SubduralHematomaAcute	FALSE	FALSE	text	This variable was assessed by a central review panel, according the TBI-Common Data Elements. This variable indicates whether an acute subdural hematoma or multiple acute subdural hematomas are present. More descriptive information (location, volume, number) and advanced information (homogeneous versus heterogeneous) can be found in the JSON files.Volume was estimated using the AxBxC/2 method, however, small traces of acute subdural blood on the tentorium or interhemispheric were not measured. Note: cfr. epidural: only "descriptive_volume" can be used for analysis. Note: In the JSON files length, width and depth of lesions can not be	absent=Absent;present=Present;indeteminate=Indeterminate;uninterpretable=Uninterpretable

Name	Static	Intervention	Format	Description	Possible Values
				used as metrics in any analysis! Only "descriptive volume" is a valid metric (in cc)	
SubduralHematomaSubacuteChronic	FALSE	FALSE	text	This variable was assessed by a central review panel, according the TBI-Common Data Elements. This variable indicates whether one or multiple subacute/chronic subdural hematoma(s) are present. More descriptive information (location, volume, number) can be found in the JSON files.Note: Volume was estimated using the AxBxC/2 method. Note: this variable is best considered together with the "SubduralCollectionMixedDensity" variable, as the central reviewer did not have information to time of injury. Many subacute/chronic subdurals were therefore categorised as "mixed density". Note: cfr. epidural and acute subdural hematoma regarding volumes. Note: In the JSON files length, width and depth of lesions can not be used as metrics in any analysis! Only "descriptive volume" is a valid metric (in cc)	absent=Absent;present=Present;indeteminate=Indeterminate;uninterpretable=Uninterpretable
TAI	FALSE	FALSE	text	This variable was assessed by a central review panel, according the TBI-Common Data Elements. This variable indicates whether axonal injury is present. More descriptive information (location, TAI versus DAI) can be found in the JSON files. Note: be aware of the modality you are interested in (i.e. CT or MRI).	absent=Absent;present=Present;indeteminate=Indeterminate;uninterpretable=Uninterpretable
Timepoint	FALSE	FALSE	text	Outcome timepoints are 2-3 weeks, 3 months, 6 months, 12 months and 24 months. Depending on the stratum and sub-studies of a patient, follow up was performed at some of these time points or at all timepoints. For an overview, please check the SOP Manual.	Clinical=Clinical;CT Early=CT Early;CT Followup=CT Follow-up;CT Post-Op=CT Post-Op;MR 12 months=MR 12 months;MR 2 weeks=MR 2 weeks;MR 24 months=MR 24 months;MR 3 months=MR 3 months;MR 6 months=MR 6 months;MR Early=MR Early
Timepoint	FALSE	FALSE	text	Outcome timepoints are 2-3 weeks, 3 months, 6 months, 12 months and 24 months. Depending on the stratum and sub-studies of a patient, follow up was performed at some of these time points or at all timepoints. For an overview, please check the SOP Manual.	12mo=12 months;24mo=24 months;2wk=2 weeks;3mo=3 months;6mo=6 months;Base=Baseline
TraumaticSubarachnoidHemorrhage	FALSE	FALSE	text	This variable was assessed by a central review panel, according the TBI-Common Data Elements. This variable indicates whether traumatic subarachnoid blood is present. More descriptive information (location and amount (e.g. trace, moderate, severe)) can be found in the JSON files. * Location can be: cortical, basal, interhemispheric or tentorial.	absent=Absent;present=Present;indeteminate=Indeterminate;uninterpretable=Uninterpretable
WindowDetectionQuality	FALSE	FALSE	text	S: Automated Detection for bone versus brain. B: Possible options: Bone Window, Brain Window, Window Uncertainty R: Only available for CT scans. No brain window scans should be classified as bone window.	Not Applicable
XsiType	FALSE	FALSE	text	S: Type of imaging (CT/MR) A: Assess if the scan is CT or MR R: Possible values 'xnat:mrSessionData' and 'xnat:ctSessionData'	xnat:ctSessionData=xnat:ctSessionData;xnat:mrSessionData=xnat:mrSessionData

Laboratory measurements

Name	Static	Intervention	Format	Description	Possible Values
AnnexinVsingleCD105Annexmeasurement	FALSE	FALSE	decimal	Measurement of CD105- and AnnexinV-positive microparticles for quantification of endothelial derived microparticles (EDMP) - results for endothelial derived microparticles (EDMP) (single positive Annexin V+) - using flow cytometry techniques. Citrated plasma sample was centrifuged for 20 min at	Not Applicable

Name	Static	Intervention	Format	Description	Possible Values
				2,500 x g at RT. Supernatants were used to determine EDMP. Run on the BD Accuri C6 Plus flow cytometer (BD Biosciences; Heidelberg, Germany) at Institute for Research in Operative Medicine (IFOM, Cologne, Germany), University Witten/Herdecke.	
AnnexinVsingleCD42bAnnexmeasurement	FALSE	FALSE	decimal	Measurement of CD42b- and AnnexinV-positive microparticles for quantification of platelet-derived microparticles (PDMP) - results for platelet-derived microparticles (PDMP) (single positive Annexin V+) - using flow cytometry techniques. Citrated plasma sample was centrifuged for 20 min at 2,500 x g at RT. Supernatants were used to determine PDMP. Run on the BD Accuri C6 Plus flow cytometer (BD Biosciences; Heidelberg, Germany) at Institute for Research in Operative Medicine (IFOM, Cologne, Germany), University Witten/Herdecke.	Not Applicable
CD105AnnexinVdoubleCD105Annexmeasurement	FALSE	FALSE	decimal	Measurement of CD105- and AnnexinV-positive microparticles for quantification of endothelial derived microparticles (EDMP) - results for endothelial derived microparticles (EDMP) (double positive CD105+/Annexin V+) - using flow cytometry techniques. Citrated plasma sample was centrifuged for 20 min at 2,500 x g at RT. Supernatants were used to determine EDMP. Run on the BD Accuri C6 Plus flow cytometer (BD Biosciences; Heidelberg, Germany) at Institute for Research in Operative Medicine (IFOM, Cologne, Germany), University Witten/Herdecke.	Not Applicable
CD105CD142doubleCD105CD142measurement	FALSE	FALSE	decimal	Measurement of CD105- and CD142-positive microparticles for quantification of endothelial derived microparticles (EDMP) - results for endothelial derived microparticles (EDMP) (double positive CD105+/CD142+) - using flow cytometry techniques. Citrated plasma sample was centrifuged for 20 min at 2,500 x g at RT. Supernatants were used to determine EDMP. Run on the BD Accuri C6 Plus flow cytometer (BD Biosciences; Heidelberg, Germany) at Institute for Research in Operative Medicine (IFOM, Cologne, Germany), University Witten/Herdecke.	Not Applicable
CD105CD62EdoubleCD105CD62Emeasurement	FALSE	FALSE	decimal	Measurement of CD105- and CD62e-positive microparticles for quantification of endothelial derived microparticles (EDMP) - results for endothelial derived microparticles (EDMP) (double positive CD105+/CD62e+) - using flow cytometry techniques. Citrated plasma sample was centrifuged for 20 min at 2,500 x g at RT. Supernatants were used to determine EDMP. Run on the BD Accuri C6 Plus flow cytometer (BD Biosciences; Heidelberg, Germany) at Institute for Research in Operative Medicine (IFOM, Cologne, Germany), University Witten/Herdecke..	Not Applicable
CD105singleCD105Annexmeasurement	FALSE	FALSE	decimal	Measurement of CD105- and AnnexinV-positive microparticles for quantification of endothelial derived microparticles (EDMP) - results for endothelial derived microparticles (EDMP) (single positive CD105+) - using flow cytometry techniques. Citrated plasma sample was centrifuged for 20 min at 2,500 x g at RT. Supernatants were used to determine EDMP. Run on the BD Accuri C6 Plus flow cytometer (BD Biosciences; Heidelberg, Germany) at Institute for	Not Applicable

Name	Static	Intervention	Format	Description	Possible Values
				Research in Operative Medicine (IFOM, Cologne, Germany), University Witten/Herdecke.	
CD105singleCD105CD142measurement	FALSE	FALSE	decimal	Measurement of CD105- and CD142-positive microparticles for quantification of endothelial derived microparticles (EDMP) - results for endothelial derived microparticles (EDMP) (single positive CD105+) - using flow cytometry techniques. Citrated plasma sample was centrifuged for 20 min at 2,500 x g at RT. Supernatants were used to determine EDMP. Run on the BD Accuri C6 Plus flow cytometer (BD Biosciences; Heidelberg, Germany) at Institute for Research in Operative Medicine (IFOM, Cologne, Germany), University Witten/Herdecke.	Not Applicable
CD105singleCD105CD62Emeasurement	FALSE	FALSE	decimal	Measurement of CD105- and CD62e-positive microparticles for quantification of endothelial derived microparticles (EDMP) - results for endothelial derived microparticles (EDMP) (single positive CD105+) - using flow cytometry techniques. Citrated plasma sample was centrifuged for 20 min at 2,500 x g at RT. Supernatants were used to determine EDMP. Run on the BD Accuri C6 Plus flow cytometer (BD Biosciences; Heidelberg, Germany) at Institute for Research in Operative Medicine (IFOM, Cologne, Germany), University Witten/Herdecke.	Not Applicable
CD142singleCD105CD142measurement	FALSE	FALSE	decimal	Measurement of CD105- and CD142-positive microparticles for quantification of endothelial derived microparticles (EDMP) - results for endothelial derived microparticles (EDMP) (single positive CD142+) - using flow cytometry techniques. Citrated plasma sample was centrifuged for 20 min at 2,500 x g at RT. Supernatants were used to determine EDMP. Run on the BD Accuri C6 Plus flow cytometer (BD Biosciences; Heidelberg, Germany) at Institute for Research in Operative Medicine (IFOM, Cologne, Germany), University Witten/Herdecke.	Not Applicable
CD42bAnnexinVdoubleCD42bAnnexmeasurement	FALSE	FALSE	decimal	Measurement of CD42b- and AnnexinV-positive microparticles for quantification of platelet-derived microparticles (PDMP) - results for platelet-derived micro-particles (double positive for CD42b+/Annexin V+) - using flow cytometry techniques. Citrated plasma sample was centrifuged for 20 min at 2,500 x g at RT. Supernatants were used to determine PDMP. Run on the BD Accuri C6 Plus flow cytometer (BD Biosciences; Heidelberg, Germany) at Institute for Research in Operative Medicine (IFOM, Cologne, Germany), University Witten/Herdecke.	Not Applicable
CD42bCD62pdoubleCD42bCD62pmeasurement	FALSE	FALSE	decimal	Measurement of CD42b- and CD62p-positive microparticles for quantification of platelet-derived microparticles (PDMP) - results for platelet-derived microparticles (PDMP) (double positive CD42b+/CD62p+) - using flow cytometry techniques. Citrated plasma sample was centrifuged for 20 min at 2,500 x g at RT. Supernatants were used to determine PDMP. Run on the BD Accuri C6 Plus flow cytometer (BD Biosciences; Heidelberg, Germany) at Institute for Research in Operative Medicine (IFOM, Cologne, Germany), University Witten/Herdecke.	Not Applicable

Name	Static	Intervention	Format	Description	Possible Values
CD42bsingleCD42bAnnexVmeasurement	FALSE	FALSE	decimal	Measurement of CD42b and AnnexinV positive microparticles for quantification of platelet-derived microparticles (PDMP) - results for platelet-derived microparticles (PDMP) (single positive CD42b+) - using flow cytometry techniques. Citrated plasma sample was centrifuged for 20 min at 2,500 x g at RT. Supernatants were used to determine PDMP. Run on the BD Accuri C6 Plus flow cytometer (BD Biosciences; Heidelberg, Germany) at Institute for Research in Operative Medicine (IFOM, Cologne, Germany), University Witten/Herdecke.	Not Applicable
CD42bsingleCD42bCD62pmeasurement	FALSE	FALSE	decimal	Measurement of CD42b- and CD62p-positive microparticles for quantification of platelet-derived microparticles (PDMP) - results for platelet-derived microparticles (PDMP) (single positive CD42b+) - using flow cytometry techniques. Citrated plasma sample was centrifuged for 20 min at 2,500 x g at RT. Supernatants were used to determine PDMP. Run on the BD Accuri C6 Plus flow cytometer (BD Biosciences; Heidelberg, Germany) at Institute for Research in Operative Medicine (IFOM, Cologne, Germany), University Witten/Herdecke.	Not Applicable
CD62EsingleCD105CD62Emeasurement	FALSE	FALSE	decimal	Measurement of CD105- and CD62e-positive microparticles for quantification of endothelial derived microparticles (EDMP) - results for endothelial derived microparticles (EDMP) (single positive CD62e+) - using flow cytometry techniques. Citrated plasma sample was centrifuged for 20 min at 2,500 x g at RT. Supernatants were used to determine EDMP. Run on the BD Accuri C6 Plus flow cytometer (BD Biosciences; Heidelberg, Germany) at Institute for Research in Operative Medicine (IFOM, Cologne, Germany), University Witten/Herdecke.	Not Applicable
CD62psingleCD42bCD62pmeasurement	FALSE	FALSE	decimal	Measurement of CD42b- and CD62p-positive microparticles for quantification of platelet-derived microparticles (PDMP) - results for platelet-derived microparticles (PDMP) (single positive CD62p+) - using flow cytometry techniques. Citrated plasma sample was centrifuged for 20 min at 2,500 x g at RT. Supernatants were used to determine PDMP. Run on the BD Accuri C6 Plus flow cytometer (BD Biosciences; Heidelberg, Germany) at Institute for Research in Operative Medicine (IFOM, Cologne, Germany), University Witten/Herdecke.	Not Applicable
CoagulationparameterAntithrombinprocent	FALSE	FALSE	decimal	Assay results for standard coagulation test (Antithrombin) - Antithrombin in human citrated plasma measured with an automated chromogenic assay technology using the HemosIL¬Æ aliquid Antithrombin kit (Werfen, Bedford, USA). Run on the ACL TOP CTS 700 (Werfen, Barcelona, Spain). Coagulationparameter were performed by the Institute of Transfusion Medicine (ITM), Cologne-Merheim Medical Centre. Normal range: 83-128 Reference: HemosIL¬Æ package insert	Not Applicable
CoagulationparameterDdimersugl	FALSE	FALSE	decimal	Assay results for standard coagulation test (D-Dimers) - using the HemosIL¬Æ D-Dimer Controls kit (Werfen, Bedford, USA). Run on the ACL TOP CTS 700 (Werfen, Barcelona, Spain). Coagulationparameter were performed by the Institute of	Not Applicable

Name	Static	Intervention	Format	Description	Possible Values
				Transfusion Medicine (ITM), Cologne-Merheim Medical Centre. Normal range: 0-232 Reference: HemosIL¬Æ package insert	
Coagulationparameter FactorIXprocent	FALSE	FALSE	decimal	Assay results for standard coagulation test (Factor IX) - human plasma immunodepleted of factor IX for the quantitaive determination of factor IX activity based on activated partial thromboplastin time (APTT) assay - using factor IX deficient plasma (Werfen, Barcelona, Spain). Run on the ACL TOP CTS 700 (Werfen, Barcelona, Spain). Coagulationparameter were performed by the Institute of Transfusion Medicine (ITM), Cologne-Merheim Medical Centre. Normal range: 65-150 Reference: HemosIL¬Æ package insert	Not Applicable
Coagulationparameter FactorVIIIprocent	FALSE	FALSE	decimal	Assay results for standard coagulation test (Factor VIII) - using a Coamatic factor VIII kit (Chromogenix, Bedford, USA) for chromogenic determination of factor VIII activity in human citrated plasma. Run on the ACL TOP CTS 700 (Werfen; Barcelona, Spain). Coagulationparameter were performed by the Institute of Transfusion Medicine (ITM), Cologne-Merheim Medical Centre. Normal range: 50-150 Reference: HemosIL¬Æ package insert	Not Applicable
Coagulationparameter FactorVprocent	FALSE	FALSE	decimal	Assay results for standard coagulation test (Factor V) - human plasma immunodepleted of factor V for the quantitaive determination of factor V activity based on the prothrombin time (PT) assay - using factor V deficient plasma (Werfen, Bedford, USA). Run on the ACL TOP CTS 700 (Werfen, Barcelona, Spain). Coagulationparameter were performed by the Institute of Transfusion Medicine (ITM), Cologne-Merheim Medical Centre. Normal range: 62-139 Reference: HemosIL¬Æ package insert	Not Applicable
Coagulationparameter FactorXIIIAgprocent	FALSE	FALSE	decimal	Assay results for standard coagulation test (Factor XIII) - measured with Chromogenix factor XIII Antigen kit (Chromogenix, Bedford, USA) based on an automated latex enhanced immunoassay techniques. Run on the ACL TOP CTS 700 (Werfen; Barcelona, Spain). Coagulationparameter were performed by the Institute of Transfusion Medicine (ITM), Cologne-Merheim Medical Centre. Normal range: 75.2-154.8 Reference: Chromogenix¬Æ package insert	Not Applicable
Coagulationparameter Fibrinogenmgdl	FALSE	FALSE	decimal	Assay results for standard coagulation test (Fibrinogen) - using a high sensitivity thromboplastin reagent (RecombiPasTin 2G (HemosIL¬Æ), Werfen, Bedford, USA) based on recombinant human tissue factor (RTF) for quantitative determination in citrated plasma of Prothrombin time (PT) and Fibrinogen. Run on the ACL TOP CTS 700 (Werfen, Barcelona, Spain). Coagulationparameter were performed by the Institute of Transfusion Medicine (ITM), Cologne-Merheim Medical Centre. Normal range: 276-471 Reference: HemosIL¬Æ package insert	Not Applicable
Coagulationparameter INR	FALSE	FALSE	decimal	Assay results for standard coagulation test (INR) - using a high sensitivity thromboplastin reagent (RecombiPasTin 2G (HemosIL¬Æ), Werfen, Bedford, USA) based on recombinant human tissue factor (RTF) for quantitative determination in citrated plasma of Prothrombin time (PT) and Fibrinogen. Run on the ACL TOP CTS 700 (Werfen, Barcelona, Spain).	Not Applicable

Name	Static	Intervention	Format	Description	Possible Values
				Coagulationparameter were performed by the Institute of Transfusion Medicine (ITM), Cologne-Merheim Medical Centre.	
Coagulationparameter Plasminogenprocent	FALSE	FALSE	decimal	Assay results for standard coagulation test (Plasminogen) - Plasminogen in human citrated plasma measured with an automated chromogenic assay technology using the HemosIL¬Æ Plasminogen kit (Werfen, Bedford, USA). Run on the ACL TOP CTS 700 (Werfen, Barcelona, Spain). Coagulationparameter were performed by the Institute of Transfusion Medicine (ITM), Cologne-Merheim Medical Centre. Normal range: 80-133 Reference: HemosIL¬Æ package insert	Not Applicable
Coagulationparameter ProteinCprocent	FALSE	FALSE	decimal	Assay results for standard coagulation test (Protein C) - Protein C in human citrated plasma measured with an automated chromogenic assay technology using the HemosIL¬Æ Protein C kit (Werfen, Bedford, USA). Run on the ACL TOP CTS 700 (Werfen; Barcelona, Spain). Coagulationparameter were performed by the Institute of Transfusion Medicine (ITM), Cologne-Merheim Medical Centre. Normal range: 70-140 Reference: HemosIL¬Æ package insert	Not Applicable
Coagulationparameter ProteinSprocent	FALSE	FALSE	decimal	Assay results for standard coagulation test (Protein S) - using the HemosIL¬Æ Protein S Activity kit (Werfen, Bedford, USA). Determination of the functional acitivty of free Protein S by measuring the degree of prolongation of a prothrombin time in the presence of recombinant human tissue factor, phospholipids, calcium ions and protein C. Run on the ACL TOP CTS 700 (Werfen, Barcelona, Spain). Coagulationparameter were performed by the Institute of Transfusion Medicine (ITM), Cologne-Merheim Medical Centre. Normal range: 63.5-149 Reference: HemosIL¬Æ package insert	Not Applicable
Coagulationparameter PTTsec	FALSE	FALSE	decimal	Assay results for standard coagulation test (PTT) - using the HemosIL¬Æ APTT-SP kit (Werfen, Bedford, USA). Run on the ACL TOP CTS 700 (Werfen; Barcelona, Spain). Coagulationparameter were performed by the Institute of Transfusion Medicine (ITM), Cologne-Merheim Medical Centre. Normal range: 23-36 Reference: HemosIL¬Æ package insert	Not Applicable
Coagulationparameter Quickprocent	FALSE	FALSE	decimal	Assay results for standard coagulation test (Quick) - using a high sensitivity thromboplastin reagent (RecombiPasTin 2G (HemosIL¬Æ), Werfen, Bedford, USA) based on recombinant human tissue factor (RTF) for quantitative determination in citrated plasma of Prothrombin time (PT) and Fibrinogen. Run on the ACL TOP CTS 700 (Werfen, Barcelona, Spain). Coagulationparameter were performed by the Institute of Transfusion Medicine (ITM), Cologne-Merheim Medical Centre. Normal range: 70-130 Reference: HemosIL¬Æ package insert	Not Applicable
Coagulationparameter Thrombintimesec	FALSE	FALSE	decimal	Assay results for standard coagulation test (Thrombin Time) - using the HemosIL¬Æ Thrombin Time kit (Werfen, Bedford, USA). Fibrinogen in the citrated plasma sample is converted to fibrin by the addition of purified bovine thrombin and the time required to form the clot is measured. Run on the ACL TOP CTS 700 (Werfen; Barcelona, Spain). Coagulationparameter	Not Applicable

Name	Static	Intervention	Format	Description	Possible Values
				were performed by the Institute of Transfusion Medicine (ITM), Cologne-Merheim Medical Centre. Normal range: 10-17 Reference: HemosIL¬Æ package insert	
Coagulationparameter vWFABT0procent	FALSE	FALSE	decimal	Assay results for standard coagulation test (von Willebrand Factor Activity) in citrated plasma samples in patients with bloodtype 0 - measured with the HemosIL¬Æ von Willebrand Activity kit (Werfen, Bedford, USA) based on an automated latex enhanced immunoassay technique. Run on the ACL TOP CTS 700 (Werfen; Barcelona, Spain). Coagulationparameter were performed by the Institute of Transfusion Medicine (ITM), Cologne-Merheim Medical Centre. Normal range: 40-126 Reference: HemosIL¬Æ package insert	Not Applicable
Coagulationparameter vWFABTABABprocent	FALSE	FALSE	decimal	Assay results for standard coagulation test (von Willebrand Factor Activity) in citrated plasma samples in patients with bloodtype A, B and AB - measured with the HemosIL¬Æ von Willebrand Activity kit (Werfen, Bedford, USA) based on an automated latex enhanced immunoassay technique. Run on the ACL TOP CTS 700 (Werfen, Barcelona, Spain). Coagulationparameter were performed by the Institute of Transfusion Medicine (ITM), Cologne-Merheim Medical Centre. Normal range: 49-163 Reference: HemosIL¬Æ package insert	Not Applicable
Coagulationparameter vWFAgABABprocent	FALSE	FALSE	decimal	Assay results for standard coagulation test (von Willebrand Factor Antigen) in citrated plasma samples in patients with bloodtype A, B and AB - measured with the HemosIL¬Æ von Willebrand Antigen kit (Werfen, Bedford, USA) based on an automated latex enhanced immunoassay technique. Run on the ACL TOP CTS 700 (Werfen, Barcelona, Spain). Coagulationparameter were performed by the Institute of Transfusion Medicine (ITM), Cologne-Merheim Medical Centre. Normal range: 66-176 Reference: HemosIL¬Æ package insert	Not Applicable
Coagulationparameter vWFAgBT0procent	FALSE	FALSE	decimal	Assay results for standard coagulation test (von Willebrand Factor Antigen) in citrated plasma samples in patients with bloodtype 0 - measured with the HemosIL¬Æ von Willebrand Antigen kit (Werfen, Bedford, USA) based on an automated latex enhanced immunoassay technique. Run on the ACL TOP CTS 700 (Werfen, Barcelona, Spain). Coagulationparameter were performed by the Institute of Transfusion Medicine (ITM), Cologne-Merheim Medical Centre. Normal range: 42-141 Reference: HemosIL¬Æ package insert	Not Applicable
DLA10Extem	FALSE	FALSE	text	Only applicable to sites doing ROTEM studies. ROTEM --> A10 --> EXTEM	Not Applicable
DLA10Fibtem	FALSE	FALSE	text	Only applicable to sites doing ROTEM studies. ROTEM --> A10 --> FIBTEM	Not Applicable
DLA5Extem	FALSE	FALSE	integer	Only applicable to sites doing ROTEM studies. ROTEM --> A5 --> EXTEM	Not Applicable
DLA5Fibtem	FALSE	FALSE	integer	Only applicable to sites doing ROTEM studies ROTEM --> A5 --> FIBTEM	Not Applicable
DLaAngleExtem	FALSE	FALSE	integer	Only applicable to sites doing ROTEM studies. ROTEM --> Œ±-angle --> EXTEM	Not Applicable
DLaAngleFibtem	FALSE	FALSE	integer	Only applicable to sites doing ROTEM studies. ROTEM --> Œ±-angle --> FIBTEM	Not Applicable
DLADPAggreg	FALSE	FALSE	decimal	Only applicable to sites doing multiplate studies. MULTIPLATE TEST --> ADP Test --> Aggregation	Not Applicable

Name	Static	Intervention	Format	Description	Possible Values
DLADPAUC	FALSE	FALSE	integer	Only applicable to sites doing multiplate studies. MULTIPLATE TEST --> ADP Test --> AUC (AU*min)	Not Applicable
DLADPVelocity	FALSE	FALSE	decimal	Only applicable to sites doing multiplate studies. MULTIPLATE TEST --> ADP Test --> Velocity	Not Applicable
DLAlatSgptUL	FALSE	FALSE	decimal	BLOOD CHEMISTRY --> ALAT/SGPT Recorded in "preferred" units (U/L)	Not Applicable
DLAlbumingL	FALSE	FALSE	decimal	BLOOD CHEMISTRY --> Albumin Recorded in "preferred" units (g/dL)	Not Applicable
DLAlkalinePhosphataseUL	FALSE	FALSE	decimal	BLOOD CHEMISTRY --> Alkaline Phosphatase Recorded in "preferred" units (U/L)	Not Applicable
DLAmylaseUL	FALSE	FALSE	decimal	BLOOD CHEMISTRY --> Amylase Recorded in "preferred" units (U/L)	Not Applicable
DLaPttsec	FALSE	FALSE	decimal	HAEMATOLOGY --> Activated thromboplastine time (aPTT) Recorded in "preferred" units (sec.)	Not Applicable
DLAsatSgotUL	FALSE	FALSE	decimal	BLOOD CHEMISTRY --> ASAT/SGOT Recorded in "preferred" units (U/L)	Not Applicable
DLASPIAggreg	FALSE	FALSE	decimal	Only applicable to sites doing multiplate studies. MULTIPLATE TEST --> ASPI Test --> Aggregation	Not Applicable
DLASPIAUC	FALSE	FALSE	integer	Only applicable to sites doing multiplate studies	Not Applicable
DLASPIVelocity	FALSE	FALSE	decimal	Only applicable to sites doing multiplate studies MULTIPLATE TEST --> ASPI Test --> Velocity (AU*min)	Not Applicable
DLCalciummmolL	FALSE	FALSE	decimal	BLOOD CHEMISTRY --> Calcium Recorded in "preferred" units (mmol/L)	Not Applicable
DLCFTExtem	FALSE	FALSE	integer	Only applicable to sites doing ROTEM studies. ROTEM --> CFT --> EXTEM	Not Applicable
DLCFTFibtem	FALSE	FALSE	integer	Only applicable to sites doing ROTEM studies. ROTEM --> CFT --> FIBTEM	Not Applicable
DLCL30	FALSE	FALSE	integer	Only applicable to sites doing ROTEM/TEG studies. TEG --> CL30	Not Applicable
DLCL60	FALSE	FALSE	integer	Only applicable to sites doing ROTEM/TEG studies. TEG --> CL60	Not Applicable
DLCOLAggreg	FALSE	FALSE	decimal	Only applicable to sites doing multiplate studies MULTIPLATE TEST --> COL Test --> Aggregation	Not Applicable
DLCOLAUC	FALSE	FALSE	integer	Only applicable to sites doing multiplate studies MULTIPLATE TEST --> COL Test --> AUC (AU*min)	Not Applicable
DLCOLVelocity	FALSE	FALSE	decimal	Only applicable to sites doing multiplate studies MULTIPLATE TEST --> COL Test --> Velocity (AU*min)	Not Applicable
DLCreatinineumolL	FALSE	FALSE	decimal	BLOOD CHEMISTRY --> Creatinine Recorded in "preferred" units (¬µmol/L)	Not Applicable
DLCRPmgL	FALSE	FALSE	decimal	HAEMATOLOGY --> C-reactive protein (CRP) Recorded in "preferred" units (mg/L)	Not Applicable
DLCTExtem	FALSE	FALSE	integer	Only applicable to sites doing ROTEM studies ROTEM --> CT --> EXTEM	Not Applicable
DLCTFibtem	FALSE	FALSE	integer	Only applicable to sites doing ROTEM studies ROTEM --> CT --> FIBTEM	Not Applicable
DLDdimersugL	FALSE	FALSE	decimal	HAEMATOLOGY --> D-dimers Recorded in "preferred" units (¬µg/L)	Not Applicable
DLEosinophilspct	FALSE	FALSE	decimal	HAEMATOLOGY --> Eosinophils Recorded in "preferred" units (%)	Not Applicable
DLEPL	FALSE	FALSE	integer	Only applicable to sites doing TEG/ROTEM studies TEG --> EPL	Not Applicable
DLFibrinogenmgdL	FALSE	FALSE	decimal	HAEMATOLOGY --> Fibrinogen Recorded in "preferred" units (mg/dL)	Not Applicable
DLGlucosemmolL	FALSE	FALSE	decimal	BLOOD CHEMISTRY --> Glucose Recorded in "preferred" units (mmol/L)	Not Applicable
DLHematocritpct	FALSE	FALSE	decimal	HAEMATOLOGY --> Hematocrit Recorded in "preferred" units (%)	Not Applicable
DLHemoglobingdL	FALSE	FALSE	decimal	HAEMATOLOGY --> Hemoglobin Recorded in "preferred" units (g/dL)	Not Applicable
DLInr	FALSE	FALSE	decimal	HAEMATOLOGY --> INR	Not Applicable
DLK	FALSE	FALSE	integer	Only applicable to sites doing TEG/ROTEM studies TEG --> K	Not Applicable

Name	Static	Intervention	Format	Description	Possible Values
DLLdhUL	FALSE	FALSE	decimal	BLOOD CHEMISTRY --> LDH (Lactate Dehydrogenase) Recorded in "preferred" units (U/L)	Not Applicable
DLLY30Extem	FALSE	FALSE	text	Only applicable to sites doing ROTEM studies ROTEM --> LY30 --> EXTEM	Not Applicable
DLLY30Fibtem	FALSE	FALSE	text	Only applicable to sites doing ROTEM studies ROTEM --> LY30 --> FIBTEM	Not Applicable
DLLymphocytespct	FALSE	FALSE	decimal	HAEMATOLOGY --> Lymphocytes Recorded in "preferred" units (%)	Not Applicable
DLMA	FALSE	FALSE	integer	Only applicable to sites doing TEG/ROTEM studies TEG --> MA	Not Applicable
DLMagnesiummmolL	FALSE	FALSE	decimal	BLOOD CHEMISTRY --> Magnesium Recorded in "preferred" units (mmol/L)	Not Applicable
DLMCFExtem	FALSE	FALSE	integer	Only applicable to sites doing ROTEM studies ROTEM --> MCF --> EXTEM	Not Applicable
DLMCFFibtem	FALSE	FALSE	integer	Only applicable to sites doing ROTEM studies ROTEM --> MCF --> FIBTEM	Not Applicable
DLMCFtExtem	FALSE	FALSE	integer	Only applicable to sites doing ROTEM studies ROTEM --> MCF-t --> EXTEM	Not Applicable
DLMCFtFibtem	FALSE	FALSE	integer	Only applicable to sites doing ROTEM studies ROTEM --> MCF-t --> FIBTEM	Not Applicable
DLMLExtem	FALSE	FALSE	text	Only applicable to sites doing ROTEM studies ROTEM --> ML --> EXTEM	Not Applicable
DLMLFibtem	FALSE	FALSE	text	Only applicable to sites doing ROTEM studies ROTEM --> ML --> FIBTEM	Not Applicable
DLNeutrophilspct	FALSE	FALSE	decimal	HAEMATOLOGY --> Neutrophils Recorded in "preferred" units (%)	Not Applicable
DLPlatelet105L	FALSE	FALSE	decimal	HAEMATOLOGY --> Platelet Recorded in "preferred" units (X10^9/L or X10^3/¬µL)	Not Applicable
DLPotassiummmolL	FALSE	FALSE	decimal	BLOOD CHEMISTRY --> Potassium Recorded in "preferred" units (mmol/L)	Not Applicable
DLProthrombineTimeSec	FALSE	FALSE	decimal	HAEMATOLOGY --> Prothrombine Time Recorded in "preferred" units (sec.)	Not Applicable
DLR	FALSE	FALSE	integer	Only applicable to sites doing TEG/ROTEM studies TEG --> R	Not Applicable
DLReason	FALSE	FALSE	integer		1=Study protocol;2=Unit policy;3=Clinical indication in specific patient
DLRISTOAggreg	FALSE	FALSE	decimal	Only applicable to sites doing multiplate studies MULTIPLATE TEST --> RISTO Test --> Aggregation	Not Applicable
DLRISTOAUC	FALSE	FALSE	integer	Only applicable to sites doing multiplate studies MULTIPLATE TEST --> RISTO Test --> AUC (AU*min)	Not Applicable
DLRISTOVelocity	FALSE	FALSE	decimal	Only applicable to sites doing multiplate studies MULTIPLATE TEST --> RISTO Test --> Velocity (AU*min)	Not Applicable
DLS100BugL	FALSE	FALSE	decimal	BLOOD CHEMISTRY --> S100B Recorded in "preferred" units (¬µg/L)	Not Applicable
DLSodiummmolL	FALSE	FALSE	decimal	BLOOD CHEMISTRY --> Sodium Recorded in "preferred" units (mmol/L)	Not Applicable
DLTEGaAngle	FALSE	FALSE	integer	Only applicable for sites doing TEG TEG --> Œ±-angle	Not Applicable
DLTEGType	FALSE	FALSE	text	Reflects type of TEG done - Only applicable for selected sites doing TEG	RAPID=Rapid;STAND=Standard
DLTMA	FALSE	FALSE	integer	Only applicable for sites doing TEG TEG --> TMA	Not Applicable
DLTotalBilirubinumolL	FALSE	FALSE	decimal	BLOOD CHEMISTRY --> Total Bilirubin Recorded in "preferred" units (¬µmol/L)	Not Applicable
DLToxScreen	FALSE	FALSE	text	Toxic Drug Screen Result Only if performed as part of clinical routine	NEG=Negative;POS=Positive
DLToxScreenPosAmphet	FALSE	FALSE	integer	Reflects if Toxic Drug Screen was positive for Amphetamines. Only if performed as part of clinical routine.	0=No;1=Yes
DLToxScreenPosBarb	FALSE	FALSE	integer	Reflects if Toxic Drug Screen was positive for Barbiturates. Only if performed as part of clinical routine.	0=No;1=Yes
DLToxScreenPosBenzo	FALSE	FALSE	integer	Reflects if Toxic Drug Screen was positive for Benzodiazepines. Only if performed as part of clinical routine.	0=No;1=Yes
DLToxScreenPosCannabis	FALSE	FALSE	integer	Reflects if Toxic Drug Screen was positive for Cannabinoids. Only if performed as part of clinical routine.	0=No;1=Yes

Name	Static	Intervention	Format	Description	Possible Values
DLToxScreenPosCocaine	FALSE	FALSE	integer	Reflects if Toxic Drug Screen was positive for Cocaine. Only if performed as part of clinical routine.	0=No;1=Yes
DLToxScreenPosMeth	FALSE	FALSE	integer	Reflects if Toxic Drug Screen was positive for Methadone. Only if performed as part of clinical routine.	0=No;1=Yes
DLToxScreenPosMethaqual	FALSE	FALSE	integer	Reflects if Toxic Drug Screen was positive for Methaqualone. Only if performed as part of clinical routine.	0=No;1=Yes
DLToxScreenPosOpiate	FALSE	FALSE	integer	Reflects if Toxic Drug Screen was positive for Opiates. Only if performed as part of clinical routine.	0=No;1=Yes
DLToxScreenPosOther	FALSE	FALSE	integer	Reflects if Toxic Drug Screen was positive for Other drugs than the predefined list. Only if performed as part of clinical routine.	0=No;1=Yes
DLToxScreenPosOtherTxt	FALSE	FALSE	text	Specifies for which drugs, if Toxic Drug Screen was positive for Other drugs than the predefined list. Only if performed as part of clinical routine.	Not Applicable
DLToxScreenPosPhency	FALSE	FALSE	integer	Reflects if Toxic Drug Screen was positive for Phencyclidine. Only if performed as part of clinical routine.	0=No;1=Yes
DLToxScreenType	FALSE	FALSE	text	Specifies the type of sample, Urine or Serum, if Toxic Drug Screen was performed. Only if performed as part of clinical routine.	SERUM=Serum;URINE=Urine
DLTRAPAggreg	FALSE	FALSE	decimal	Only applicable to sites doing multiplate studies. MULTIPLATE TEST --> TRAP Test --> Aggregation	Not Applicable
DLTRAPAUC	FALSE	FALSE	integer	Only applicable to sites doing multiplate studies. MULTIPLATE TEST --> TRAP Test --> AUC (AU*min)	Not Applicable
DLTRAPVelocity	FALSE	FALSE	decimal	Only applicable to sites doing multiplate studies. MULTIPLATE TEST --> TRAP Test --> Velocity (AU*min)	Not Applicable
DLUreammolL	FALSE	FALSE	decimal	BLOOD CHEMISTRY --> Urea Recorded in "preferred" units (mmol/L)	Not Applicable
DLWhiteBloodCellpct	FALSE	FALSE	decimal	HAEMATOLOGY --> White blood cell Recorded in "preferred" units (X10^9/L or X10^3/ŒºL)	Not Applicable
ERA10Extem	TRUE	FALSE	text	In emergency room: Only applicable to sites doing ROTEM studies. ROTEM --> A10 --> EXTEM	Not Applicable
ERA10Fibtem	TRUE	FALSE	text	In emergency room: Only applicable to sites doing ROTEM studies. ROTEM --> A10 --> FIBTEM	Not Applicable
ERA5Extem	TRUE	FALSE	integer	In emergency room: Only applicable to sites doing ROTEM studies. ROTEM --> A5 --> EXTEM	Not Applicable
ERA5Fibtem	TRUE	FALSE	integer	In emergency room: Only applicable to sites doing ROTEM studies ROTEM --> A5 --> FIBTEM	Not Applicable
ERaAngleExtem	TRUE	FALSE	integer	In emergency room: Only applicable to sites doing ROTEM studies. ROTEM --> Œ±-angle --> EXTEM	Not Applicable
ERaAngleFibtem	TRUE	FALSE	integer	In emergency room: Only applicable to sites doing ROTEM studies. ROTEM --> Œ±-angle --> FIBTEM	Not Applicable
ERADPAggreg	TRUE	FALSE	decimal	In emergency room: Only applicable to sites doing multiplate studies. MULTIPLATE TEST --> ADP Test --> Aggregation	Not Applicable
ERADPVelocity	TRUE	FALSE	decimal	In emergency room: Only applicable to sites doing multiplate studies. MULTIPLATE TEST --> ADP Test --> Velocity	Not Applicable
ERAlatSgptUL	TRUE	FALSE	decimal	In emergency room: BLOOD CHEMISTRY --> ALAT/SGPT Recorded in "preferred" units (U/L)	Not Applicable
ERAlbumingL	TRUE	FALSE	decimal	In emergency room: BLOOD CHEMISTRY --> Albumin Recorded in "preferred" units (g/dL)	Not Applicable
ERAlkalinePhosphataseUL	TRUE	FALSE	decimal	In emergency room: BLOOD CHEMISTRY --> Alkaline Phosphatase Recorded in "preferred" units (U/L)	Not Applicable

Name	Static	Intervention	Format	Description	Possible Values
ERAmylaseUL	TRUE	FALSE	decimal	In emergency room: BLOOD CHEMISTRY --> Amylase Recorded in "preferred" units (U/L)	Not Applicable
ERaPttsec	TRUE	FALSE	decimal	In emergency room: HAEMATOLOGY --> Activated thromboplastine time (aPTT) Recorded in "preferred" units (sec.)	Not Applicable
ERAsatSgotUL	TRUE	FALSE	decimal	In emergency room: BLOOD CHEMISTRY --> ASAT/SGOT Recorded in "preferred" units (U/L)	Not Applicable
ERASPIAggreg	TRUE	FALSE	decimal	In emergency room: Only applicable to sites doing multiplate studies. MULTIPLATE TEST --> ASPI Test --> Aggregation	Not Applicable
ERASPIVelocity	TRUE	FALSE	decimal	In emergency room: Only applicable to sites doing multiplate studies MULTIPLATE TEST --> ASPI Test --> Velocity (AU*min)	Not Applicable
ERCalciummmolL	TRUE	FALSE	decimal	In emergency room: BLOOD CHEMISTRY --> Calcium Recorded in "preferred" units (mmol/L)	Not Applicable
ERCFTExtem	TRUE	FALSE	integer	In emergency room: Only applicable to sites doing ROTEM studies. ROTEM --> CFT --> EXTEM	Not Applicable
ERCFTFibtem	TRUE	FALSE	integer	In emergency room: Only applicable to sites doing ROTEM studies. ROTEM --> CFT --> FIBTEM	Not Applicable
ERCL30	TRUE	FALSE	integer	In emergency room: Only applicable to sites doing ROTEM/TEG studies. TEG --> CL30	Not Applicable
ERCL60	TRUE	FALSE	integer	In emergency room: Only applicable to sites doing ROTEM/TEG studies. TEG --> CL60	Not Applicable
ERCOLAggreg	TRUE	FALSE	decimal	In emergency room: Only applicable to sites doing multiplate studies MULTIPLATE TEST --> COL Test --> Aggregation	Not Applicable
ERCOLVelocity	TRUE	FALSE	decimal	In emergency room: Only applicable to sites doing multiplate studies MULTIPLATE TEST --> COL Test --> Velocity (AU*min)	Not Applicable
ERCreatinineumolL	TRUE	FALSE	decimal	In emergency room: BLOOD CHEMISTRY --> Creatinine Recorded in "preferred" units (¬µmol/L)	Not Applicable
ERCRPmgL	TRUE	FALSE	decimal	In emergency room: HAEMATOLOGY --> C-reactive protein (CRP) Recorded in "preferred" units (mg/L)	Not Applicable
ERCTExtem	TRUE	FALSE	integer	In emergency room: Only applicable to sites doing ROTEM studies ROTEM --> CT --> EXTEM	Not Applicable
ERCTFibtem	TRUE	FALSE	integer	In emergency room: Only applicable to sites doing ROTEM studies ROTEM --> CT --> FIBTEM	Not Applicable
ERDdimersugL	TRUE	FALSE	decimal	In emergency room: HAEMATOLOGY --> D-dimers Recorded in "preferred" units (¬µg/L)	Not Applicable
EREosinophilspct	TRUE	FALSE	decimal	In emergency room: HAEMATOLOGY --> Eosinophils Recorded in "preferred" units (%)	Not Applicable
EREPL	TRUE	FALSE	integer	In emergency room: Only applicable to sites doing TEG/ROTEM studies TEG --> EPL	Not Applicable
ERFibrinogenmgdL	TRUE	FALSE	decimal	In emergency room: HAEMATOLOGY --> Fibrinogen Recorded in "preferred" units (mg/dL)	Not Applicable
ERGlucosemmolL	TRUE	FALSE	decimal	In emergency room: BLOOD CHEMISTRY --> Glucose Recorded in "preferred" units (mmol/L)	Not Applicable
ERHematocritpct	TRUE	FALSE	decimal	In emergency room: HAEMATOLOGY --> Hematocrit Recorded in "preferred" units (%)	Not Applicable
ERHemoglobingdL	TRUE	FALSE	decimal	In emergency room: HAEMATOLOGY --> Hemoglobin Recorded in "preferred" units (g/dL)	Not Applicable
ERInr	TRUE	FALSE	decimal	In emergency room: HAEMATOLOGY --> INR	Not Applicable
ERK	TRUE	FALSE	integer	In emergency room: Only applicable to sites doing TEG/ROTEM studies TEG --> K	Not Applicable
ERLabs	TRUE	FALSE			Not Applicable

Name	Static	Intervention	Format	Description	Possible Values
ERLdhUL	TRUE	FALSE	decimal	In emergency room: BLOOD CHEMISTRY --> LDH (Lactate Dehydrogenase) Recorded in "preferred" units (U/L)	Not Applicable
ERLY30Extem	TRUE	FALSE	text	In emergency room: Only applicable to sites doing ROTEM studies ROTEM --> LY30 --> EXTEM	Not Applicable
ERLY30Fibtem	TRUE	FALSE	text	In emergency room: Only applicable to sites doing ROTEM studies ROTEM --> LY30 --> FIBTEM	Not Applicable
ERLymphocytespct	TRUE	FALSE	decimal	In emergency room: HAEMATOLOGY --> Lymphocytes Recorded in "preferred" units (%)	Not Applicable
ERMA	TRUE	FALSE	integer	In emergency room: Only applicable to sites doing TEG/ROTEM studies TEG --> MA	Not Applicable
ERMagnesiummmolL	TRUE	FALSE	decimal	In emergency room: BLOOD CHEMISTRY --> Magnesium Recorded in "preferred" units (mmol/L)	Not Applicable
ERMCFExtem	TRUE	FALSE	integer	In emergency room: Only applicable to sites doing ROTEM studies ROTEM --> MCF --> EXTEM	Not Applicable
ERMCFFibtem	TRUE	FALSE	integer	In emergency room: Only applicable to sites doing ROTEM studies ROTEM --> MCF --> FIBTEM	Not Applicable
ERMCFtExtem	TRUE	FALSE	integer	In emergency room: Only applicable to sites doing ROTEM studies ROTEM --> MCF-t --> EXTEM	Not Applicable
ERMCFtFibtem	TRUE	FALSE	integer	In emergency room: Only applicable to sites doing ROTEM studies ROTEM --> MCF-t --> FIBTEM	Not Applicable
ERMLExtem	TRUE	FALSE	text	In emergency room: Only applicable to sites doing ROTEM studies ROTEM --> ML --> EXTEM	Not Applicable
ERMLFibtem	TRUE	FALSE	text	In emergency room: Only applicable to sites doing ROTEM studies ROTEM --> ML --> FIBTEM	Not Applicable
ERNeutrophilspct	TRUE	FALSE	decimal	In emergency room: HAEMATOLOGY --> Neutrophils Recorded in "preferred" units (%)	Not Applicable
ERPlatelet105L	TRUE	FALSE	decimal	In emergency room: HAEMATOLOGY --> Platelet Recorded in "preferred" units (X10^9/L or X10^3/¬µL)	Not Applicable
ERPotassiummmolL	TRUE	FALSE	decimal	In emergency room: BLOOD CHEMISTRY --> Potassium Recorded in "preferred" units (mmol/L)	Not Applicable
ERProthrombineTimeSec	TRUE	FALSE	decimal	In emergency room: HAEMATOLOGY --> Prothrombine Time Recorded in "preferred" units (sec.)	Not Applicable
ERR	TRUE	FALSE	integer	In emergency room: Only applicable to sites doing TEG/ROTEM studies TEG --> R	Not Applicable
ERS100BugL	TRUE	FALSE	decimal	In emergency room: BLOOD CHEMISTRY --> S100B Recorded in "preferred" units (¬µg/L)	Not Applicable
ERSodiummmolL	TRUE	FALSE	decimal	In emergency room: BLOOD CHEMISTRY --> Sodium Recorded in "preferred" units (mmol/L)	Not Applicable
ERTEGaAngle	TRUE	FALSE	integer	In emergency room: Only applicable for sites doing TEG TEG --> Œ±-angle	Not Applicable
ERTEGType	TRUE	FALSE	text	In emergency room: Reflects type of TEG done - Only applicable for selected sites doing TEG	RAPID=Rapid;STAND=Standard
ERTMA	TRUE	FALSE	integer	In emergency room: Only applicable for sites doing TEG TEG --> TMA	Not Applicable
ERTotalBilirubinumolL	TRUE	FALSE	decimal	In emergency room: BLOOD CHEMISTRY --> Total Bilirubin Recorded in "preferred" units (¬µmol/L)	Not Applicable
ERToxScreen	TRUE	FALSE	text	In emergency room: Toxic Drug Screen Result Only if performed as part of clinical routine	NEG=Negative;POS=Positive
ERToxScreenPosAmphet	TRUE	FALSE	integer	In emergency room: Reflects if Toxic Drug Screen was positive for Amphetamines. Only if performed as part of clinical routine.	0=No;1=Yes

Name	Static	Intervention	Format	Description	Possible Values
ERToxScreenPosBarb	TRUE	FALSE	integer	In emergency room: Reflects if Toxic Drug Screen was positive for Barbiturates. Only if performed as part of clinical routine.	0=No;1=Yes
ERToxScreenPosBenzo	TRUE	FALSE	integer	In emergency room: Reflects if Toxic Drug Screen was positive for Benzodiazepines. Only if performed as part of clinical routine.	0=No;1=Yes
ERToxScreenPosCannabis	TRUE	FALSE	integer	In emergency room: Reflects if Toxic Drug Screen was positive for Cannabinoids. Only if performed as part of clinical routine.	0=No;1=Yes
ERToxScreenPosCocaine	TRUE	FALSE	integer	In emergency room: Reflects if Toxic Drug Screen was positive for Cocaine. Only if performed as part of clinical routine.	0=No;1=Yes
ERToxScreenPosMeth	TRUE	FALSE	integer	In emergency room: Reflects if Toxic Drug Screen was positive for Methadone. Only if performed as part of clinical routine.	0=No;1=Yes
ERToxScreenPosMethaqual	TRUE	FALSE	integer	In emergency room: Reflects if Toxic Drug Screen was positive for Methaqualone. Only if performed as part of clinical routine.	0=No;1=Yes
ERToxScreenPosOpiate	TRUE	FALSE	integer	In emergency room: Reflects if Toxic Drug Screen was positive for Opiates. Only if performed as part of clinical routine.	0=No;1=Yes
ERToxScreenPosOther	TRUE	FALSE	integer	In emergency room: Reflects if Toxic Drug Screen was positive for Other drugs than the predefined list. Only if performed as part of clinical routine.	0=No;1=Yes
ERToxScreenPosOtherTxt	TRUE	FALSE	text	In emergency room: Specifies for which drugs, if Toxic Drug Screen was positive for Other drugs than the predefined list. Only if performed as part of clinical routine.	Not Applicable
ERToxScreenPosPhency	TRUE	FALSE	integer	In emergency room: Reflects if Toxic Drug Screen was positive for Phencyclidine. Only if performed as part of clinical routine.	0=No;1=Yes
ERToxScreenType	TRUE	FALSE	text	In emergency room: Specifies the type of sample, Urine or Serum, if Toxic Drug Screen was performed. Only if performed as part of clinical routine.	SERUM=Serum;URINE=Urine
ERTRAPAggreg	TRUE	FALSE	decimal	In emergency room: Only applicable to sites doing multiplate studies. MULTIPLATE TEST --> TRAP Test --> Aggregation	Not Applicable
ERTRAPVelocity	TRUE	FALSE	decimal	In emergency room: Only applicable to sites doing multiplate studies. MULTIPLATE TEST --> TRAP Test --> Velocity (AU*min)	Not Applicable
ERUreammolL	TRUE	FALSE	decimal	In emergency room: BLOOD CHEMISTRY --> Urea Recorded in "preferred" units (mmol/L)	Not Applicable
ERWhiteBloodCellpct	TRUE	FALSE	decimal	In emergency room: HAEMATOLOGY --> White blood cell Recorded in "preferred" units (X10^9/L or X10^3/ŒºL)	Not Applicable
FibrinolysisFibrinogenMonomerugml	FALSE	FALSE	decimal	Assay results for fibrin monomers -using an immunoturbidimetric determination technology (STA - Liatest FM-Kit, Stago; France). Measurement were performed at the Ludwig Boltzman Institute (Salzburg, Vienna). Reference interval of Liatest FM-Kit of 6	Not Applicable
FibrinolysisregulatorAntiplasminProzent	FALSE	FALSE	decimal	Assay results for Antiplasmin - measured with a colorimetric assay technology (STA-Stachrom¬Æ-TAFI-Kit, Stago; France). Measurement were performed at the Ludwig Boltzman Institute (Salzburg, Vienna). Normal range: 80-120 Reference: STA-Stachrom¬Æ-TAFI-Kit package insert	Not Applicable
FibrinolysisregulatorTAFIprocent	FALSE	FALSE	decimal	Assay results for Thrombin-Activatable Fibrinolysis Inhibitor (TAFI) -measured with a colorimetric assay technology (STA-Stachrom-TAFI-Kit, Stago; France). Measurement were performed at the Ludwig Boltzman Institute (Salzburg, Vienna). Detection limit of Stachrom-TAFI-Kit: 5-195	Not Applicable
GFAP	FALSE	FALSE	decimal	Assay results for Glial fibrillary acidic protein [GFAP] - measured with an ultrasensitive immunoassay using digital	Not Applicable

Name	Static	Intervention	Format	Description	Possible Values
				array technology (Single Molecule Arrays, SiMoA)-based Human Neurology 4-Plex B assay (N4PB) run on the SR-X benchtop assay platform (Quanterix Corp., Lexington, MA) at the University of Florida (Gainesville, Florida).	
NFL	FALSE	FALSE	decimal	Assay results for Neurofilament protein-light (NFL) - measured with an ultrasensitive immunoassay using digital array technology (Single Molecule Arrays, SiMoA)-based Human Neurology 4-Plex B assay (N4PB) run on the SR-X benchtop assay platform (Quanterix Corp., Lexington, MA) at the University of Florida (Gainesville, Florida).	Not Applicable
NSE	FALSE	FALSE	decimal	Assay results for Neuron-specific enolase (NSE) - measured with a clinical-use automated system, using an electrochemiluminescence immunoassay kit (ECLIA) (Elecsys S100 and Elecsys NSE assays) run on the e 602 module of Cobas 8000 modular analyzer (Roche Diagnostics, Mannheim, Germany) at the University of Pecs (Pecs, Hungary).	Not Applicable
PAI1ngml	FALSE	FALSE	decimal	Assay results for Plasminogen-activator-inhibitor-1 (PAI-1) - using an ELISA Duo Set Kit from R&D Systems (Minneapolis, USA) based on the sandwich principle according to manufacturer‚Äôs instructions. EDTA plasma samples were used. Run on the EPOCH 2 (BioTek; Winooski, USA) microplate reader at Institute for Research in Operative Medicine (IFOM, Cologne, Germany), University Witten/Herdecke.	Not Applicable
S100B	FALSE	FALSE	decimal	Assay results for S100 calciumbinding protein B (S100B) - measured with a clinical-use automated system, using an electrochemiluminescence immunoassay kit (ECLIA) (Elecsys S100 and Elecsys NSE assays) run on the e 602 module of Cobas 8000 modular analyzer (Roche Diagnostics, Mannheim, Germany) at the University of Pecs (Pecs, Hungary).	Not Applicable
Syndecan1pgml	FALSE	FALSE	decimal	Assay results for Syndecan-1 - using an ELISA Duo Set Kit from R&D Systems (Minneapolis, USA) based on the sandwich principle according to manufacturer‚Äôs instructions. EDTA plasma samples were used. Run on the EPOCH 2 (BioTek; Winooski, USA) microplate reader at Institute for Research in Operative Medicine (IFOM, Cologne, Germany), University Witten/Herdecke.	Not Applicable
Tau	FALSE	FALSE	decimal	Assay results for T-TAU - measured with an ultrasensitive immunoassay using digital array technology (Single Molecule Arrays, SiMoA)-based Human Neurology 4-Plex B assay (N4PB) run on the SR-X benchtop assay platform (Quanterix Corp., Lexington, MA) at the University of Florida (Gainesville, Florida).	Not Applicable
ThrombingenerationETPnmmin	FALSE	FALSE	decimal	Assay results for endogenous thrombin potential (ETP) (STG¬Æ-BleedScreen, Stago, France) in citrated plasma - using a fluorogenic method (ST Genesia analyzer; Stago, France) - triggered by a low concentration of tissue factor. Measurement were performed at the Ludwig Boltzman Institute (Salzburg, Vienna).	Not Applicable
ThrombingenerationETPprocent	FALSE	FALSE	decimal	Assay results for endogenous thrombin potential (ETP) (STG¬Æ-BleedScreen, Stago, France) in citrated plasma - using a fluorogenic method (ST Genesia analyzer, Stago; France) - triggered by a low	Not Applicable

Name	Static	Intervention	Format	Description	Possible Values
				concentration of tissue factor. Measurement were performed at the Ludwig Boltzman Institute (Salzburg, Vienna).	
ThrombingenerationLagTimemin	FALSE	FALSE	decimal	Assay results for quantitative determination of thrombin generation (STG¬Æ-BleedScreen, Stago, France) in citrated plasma (Lag Time) - using a fluorogenic method (ST Genesia analyzer, Stago; France) - triggered by a low concentration of tissue factor. Measurement were performed at the Ludwig Boltzman Institute (Salzburg, Vienna).	Not Applicable
ThrombingenerationLagTimeratio	FALSE	FALSE	decimal	Assay results for quantitative determination of thrombin generation (STG¬Æ-BleedScreen, Stago, France) in citrated plasma (Lag Time) - using a fluorogenic method (ST Genesia analyzer, Stago; France) - triggered by a low concentration of tissue factor. Measurement were performed at the Ludwig Boltzman Institute (Salzburg, Vienna).	Not Applicable
ThrombingenerationPeakHeightnm	FALSE	FALSE	decimal	Assay results for quantitative determination of thrombin generation (STG¬Æ-BleedScreen, Stago, France) in citrated plasma (Peak Height) - using a fluorogenic method (ST Genesia analyzer Stago; France) - triggered by a low concentration of tissue factor. Measurement were performed at the Ludwig Boltzman Institute (Salzburg, Vienna).	Not Applicable
ThrombingenerationPeakHeightprocent	FALSE	FALSE	decimal	Assay results for quantitative determination of thrombin generation (STG¬Æ-BleedScreen, Stago, France) in citrated plasma (Peak Height) - using a fluorogenic method (ST Genesia analyzer, Stago; France) - triggered by a low concentration of tissue factor. Measurement were performed at the Ludwig Boltzman Institute (Salzburg, Vienna).	Not Applicable
ThrombingenerationStartTailmin	FALSE	FALSE	decimal	Assay results for quantitative determination of thrombin generation in citrated plasma (Start Tail) - using a fluorogenic method (ST Genesia analyzer, Stago; France) - triggered by a low concentration of tissue factor. Measurement were performed at the Ludwig Boltzman Institute (Salzburg, Vienna).	Not Applicable
ThrombingenerationStartTailratio	FALSE	FALSE	decimal	Assay results for quantitative determination of thrombin generation in citrated plasma (Start Tail) - using a fluorogenic method (ST Genesia analyzer, Stago; France) - triggered by a low concentration of tissue factor. Measurement were performed at the Ludwig Boltzman Institute (Salzburg, Vienna).	Not Applicable
ThrombingenerationTimetoPeakmin	FALSE	FALSE	decimal	Assay results for quantitative determination of thrombin generation in citrated plasma (Time to Peak) - using a fluorogenic method (ST Genesia analyzer; Stago, France) - triggered by a low concentration of tissue factor. Measurement were performed at the Ludwig Boltzman Institute (Salzburg, Vienna).	Not Applicable
ThrombingenerationTimetoPeakratio	FALSE	FALSE	decimal	Assay results for quantitative determination of thrombin generation in citrated plasma (Time to Peak) - using a fluorogenic method (ST Genesia analyzer, Stago; France) - triggered by a low concentration of tissue factor. Measurement were performed at the Ludwig Boltzman Institute (Salzburg, Vienna).	Not Applicable
ThrombingenerationVelIndexnmmin	FALSE	FALSE	decimal	Assay results for quantitative determination of thrombin generation in citrated plasma (Velocity Index) - using a fluorogenic method (ST Genesia analyzer, Stago; France) - triggered by a low concentration of	Not Applicable

Name	Static	Intervention	Format	Description	Possible Values
				tissue factor. Measurement were performed at the Ludwig Boltzman Institute (Salzburg, Vienna).	
ThrombingenerationVelIndexprocent	FALSE	FALSE	decimal	Assay results for quantitative determination of thrombin generation in in citrated plasma (Velocity index) - using a fluorogenic method (ST Genesia analyzer, Stago; France) - triggered by a low concentration of tissue factor. Measurement were performed at the Ludwig Boltzman Institute (Salzburg, Vienna).	Not Applicable
VECadherinngml	FALSE	FALSE	decimal	Assay results for VE-Cadherin - using an ELISA Duo Set Kit from R&D Systems (Minneapolis, USA) based on the sandwich principle according to manufacturer‚Äôs instructions. EDTA plasma samples were used. Run on the EPOCH 2 (BioTek; Winooski, USA) microplate reader at Institute for Research in Operative Medicine (IFOM, Cologne, Germany), University Witten/Herdecke.	Not Applicable

ICU medications and management

Name	Static	Intervention	Format	Description	Possible Values
Agent	FALSE	TRUE	integer	Details on medication captured information on Class, Agent, Reason, Highest daily dose, Route, start and stop date and whether or not medication has been ongoing after discharge. This variable describes the Agent. Agents commonly used for treatment of raised ICP are listed in the TIL section, but not included in the medication lists here.	1=Analgesic: paracetamol;2=Analgesic: NSAIDs;3=Analgesic: tramadol;4=Analgesic: opioids (morphine, ect);5=Sedatives/treatment of agitation: barbiturates (penthothal, ect);6=Sedatives/treatment of agitation: clondine;7=Sedatives/treatment of agitation: dexmedetomidine;8=Sedatives/treatment of agitation: diazepam;9=Sedatives/treatment of agitation: fentanyl;10=Sedatives/treatment of agitation: haloperidol (haldol);11=Sedatives/treatment of agitation: lorazepam (tenesta, ect);12=Sedatives/treatment of agitation: midazolam;13=Sedatives/treatment of agitation: morphine;14=Sedatives/treatment of agitation: propofol;15=Sedatives/treatment of agitation: other;16=Neuromuscular blockade: pancuronium (pavulon);17=Neuromuscular blockade: atracurium (tracium);18=Neuromuscular blockade: cisatracurium (nimbex);19=Neuromuscular blockad: gallamine (flaxedil);20=Neuromuscular blockade: rocuronium (zemuron);21=Neuromuscular blockade: vecuronium (norcuron);22=Neuromuscular blockade: other;23=Anti- epileptic: carbamazepine (tegretol);24=Anti- epileptic: lamotrigine (lamectal);25=Anti- epileptic: levetirazetam (keppra);26=Anti- epileptic: phenytoine (diphantoine);27=Anti- epileptic: valproate (depakine);28=Anti- epileptic: other;29=Antibiotics: aminoglycoside (amikacine, gentamicine etc);30=Antibiotics: carbapemens (meronem etc);31=Antibiotics: cephalosporin 1st gen (cefalexin etc);32=Antibiotics: cephalosporin 2nd gen (cefuroxim etc);33=Antibiotics: cephalosporin 3rd gen (cefotaxine etc);34=Antibiotics: cephalosporin 4th gen (cefepime, maxipime etc);35=Antibiotics: cephalosporin 5th gen (ceftasoline etc);36=Antibiotics: glycopeptides (vancomycine);37=Antibiotics: lincosamides (clindamycine etc);38=Antibiotics: macrolidis (erythromycine etc);39=Antibiotics: nitrofurones (furoxone, furadantine etc);40=Antibiotics: penicillines (ampicilline, cloxacilline);41=Antibiotics: amoxycilline/clavulanic acid (augmentin, tyclav etc);42=Antibiotics: quinolones (ciprofloxacine etc);43=Antibiotics: sulfonamides (co-trimoxazole, doxycycline);44=Antibiotics: other;45=Anti-hypertensive: ACE blockers (captopril, perindopril etc);46=Anti-hypertensive: angiotensininhibitors (cardesartan etc);47=Anti- hypertensive: bA"tablockers (propanolol);48=Anti-hypertensive: clonidine;49=Anti-hypertensive: diuretics;50=Calcium channel blockers: nimodipine;51=Calcium channel blockers: nicardipine;52=Calcium channel blockers: verapamil;53=Steroids: methylprednisolone;54=Steroids: bA(c)tametasone;55=Steroids: dexametasone;56=Steroids: hydrocortisone/cortisone;57=Antacids: Aluminium hydroxide;58=Antacids: other;59=H2 receptor antagonist: Cimetidine;60=H2 receptor antagonist: Ranitidine (Zantac);61=Proton pump inhibitors: Omeprazol (Losec);62=Proton pump inhibitors: Esomeprazol (Nexium);63=Proton pump inhibitors: Pantoprazole (Pantozol);64=Prokinetics: Domperidon

Name	Static	Intervention	Format	Description	Possible Values
					(Motilium);65=Prokinetics: Erythromycin;66=Prokinetics: Metoclopramide (Primperan);67=Analgesic: other;68=Anti-hypertensive: other;69=Calcium channel blockers: other;70=Steroids: other;71=H2 receptor antagonist: other;72=Proton pump inhibitors: other;73=Prokinetics:other;99=Other, specify in Agent Other: Other
AgentOther	FALSE	TRUE	text	Details on medication captured information on Class, Agent, Reason, Highest daily dose, Route, start and stop date and whether or not medication has been ongoing after discharge. This variable describes if the Agent was "other" than the predefined list.	Not Applicable
CauseOfDelay	FALSE	FALSE	integer	WHY question: documents reason for delayed transition of care	1=Unavailability of beds in the receiving unit;2=Unavailability of transport;3=Wish of patient/proxies;4=Need for isolation due to multi resistant bacteria;5=Funding issues;6=Bureaucratic causes;99=Other
CauseOfDelayOther	FALSE	FALSE	text	WHY question: documents "other" reason for delayed transition of care than predefined list	Not Applicable
Class	FALSE	TRUE	integer	Details on medication captured information on Class, Agent, Reason, Highest daily dose, Route, start and stop date and whether or not medication has been ongoing after discharge. This variable describes the Classes. Classes: analgesic, sedatives, neuromuscular blocking agents, anti-epileptic drugs, antibiotiucs, anti-hypertensive, calcium channel blockers, steroids, antacids, H2 receptor antagonists, proton pump inhibitors and prokinetics. Agents commonly used for treatment of raised ICP are listed in the TIL section, but not included in the medication lists here.	1=Analgesic;2=Sedatives/treatment of agitation;3=Neuromuscular blockade;4=Anti-epileptic;5=Antibiotics;6=Anti-hypertensive;7=Calcium channel blockers;8=Steroids;9=Antacids;10=H2 receptor antagonist;11=Proton pump inhibitors;12=Prokinetics;99=Other, specify in Agent Other
DVTPharmType	FALSE	TRUE	integer	These variables aim to document specific information on the use of DVT prophylaxis. Little evidence exists on the use and timing of DVT prophylaxis after TBI, and considerable practice variation exists. This variable describes the Type of prophylaxis in case of Pharmacologic DVT.	1=Heparin;2=Low molecular weight Heparin;3=Dalteparin (Fragmin);4=Enoxaparin;5=Nadroparin (Fraxiparine, Fraxodil);6=Parnaparin;7=Reviparin;8=Tinzaparin
DVTProphylaxisMech	FALSE	TRUE	integer	These variables aim to document specific information on the use of DVT prophylaxis. Little evidence exists on the use and timing of DVT prophylaxis after TBI, and considerable practice variation exists. This variable describes presence or absence of Mechanical DVT .	0=No;1=Yes
DVTProphylaxisMechType	FALSE	TRUE	text	These variables aim to document specific information on the use of DVT prophylaxis. Little evidence exists on the use and timing of DVT prophylaxis after TBI, and considerable practice variation exists. This variable describes the Type of prophylaxis in case of Mechanical DVT.	Not Applicable
DVTProphylaxisPharm	FALSE	TRUE	integer	These variables aim to document specific information on the use of DVT prophylaxis. Little evidence exists on the use and timing of DVT prophylaxis after TBI, and considerable practice variation exists. This variable describes presence or absence of Pharmacologic DVT.	0=No;1=Yes
EnteralNutrition	FALSE	TRUE	integer	These variables aim to document specific information on nutritional support, provided by parenteral and/or enteral routes. This variable describes the absence or presence of Enteral Nutrition.	0=No;1=Yes
EnteralNutritionRoute	FALSE	TRUE	integer	These variables aim to document specific information on nutritional support, provided by parenteral and/or enteral routes. This variable describes the route of administration for Enteral Nutrition.	1=Nasogastric tube;2=Transpyloric tube;3=Gastrostomy
HighestDailyDose	FALSE	TRUE	text	Details on medication captured information on Class, Agent, Reason, Highest daily dose, Route, start and stop date and whether or not medication has been ongoing after discharge. This variable describes the	Not Applicable

Name	Static	Intervention	Format	Description	Possible Values
				Highest Daily Dose. These details should be entered for each agent.	
HospSecondInsultsNeuroWorseAction1	FALSE	TRUE	integer	Action taken in case of Neuroworsening. The importance of Neuroworsening was first described by Morris and Marshall. The occurrence of neuroworsening is related to poorer outcome in subjects with moderate to severe TBI. Neuroworsening is defined as 1) a decrease in GCS motor score of 2 or more points; 2) a new loss of pupillary reactivity or development of pupillary assymmetry >= 2mm; 3) deterioration in neurological or CT status sufficient to warrant immediate medical or surgical intervention	1=None; 2=Unscheduled CT scan; 3=Change in medical therapy' 4=Surgical intervention
HospSecondInsultsNeuroWorseAction2	FALSE	TRUE	integer	Action taken in case of Neuroworsening. The importance of Neuroworsening was first described by Morris and Marshall. The occurrence of neuroworsening is related to poorer outcome in subjects with moderate to severe TBI. Neuroworsening is defined as 1) a decrease in GCS motor score of 2 or more points; 2) a new loss of pupillary reactivity or development of pupillary assymmetry >= 2mm; 3) deterioration in neurological or CT status sufficient to warrant immediate medical or surgical intervention	1=None; 2=Unscheduled CT scan; 3=Change in medical therapy' 4=Surgical intervention
HospSecondInsultsNeuroWorseAction3	FALSE	TRUE	integer	Action taken in case of Neuroworsening. The importance of Neuroworsening was first described by Morris and Marshall. The occurrence of neuroworsening is related to poorer outcome in subjects with moderate to severe TBI. Neuroworsening is defined as 1) a decrease in GCS motor score of 2 or more points; 2) a new loss of pupillary reactivity or development of pupillary assymmetry >= 2mm; 3) deterioration in neurological or CT status sufficient to warrant immediate medical or surgical intervention	1=None; 2=Unscheduled CT scan; 3=Change in medical therapy' 4=Surgical intervention
HospSecondInsultsNeuroWorseAction4	FALSE	TRUE	integer	Action taken in case of Neuroworsening. The importance of Neuroworsening was first described by Morris and Marshall. The occurrence of neuroworsening is related to poorer outcome in subjects with moderate to severe TBI. Neuroworsening is defined as 1) a decrease in GCS motor score of 2 or more points; 2) a new loss of pupillary reactivity or development of pupillary assymmetry >= 2mm; 3) deterioration in neurological or CT status sufficient to warrant immediate medical or surgical intervention	1=None; 2=Unscheduled CT scan; 3=Change in medical therapy' 4=Surgical intervention
HVTIL	FALSE	TRUE	integer	Bihourly change in therapy intensity level (TIL)	0=No change;1=Increasing intensity;2=Decreasing intensity
HVTILChangeReason	FALSE	FALSE	integer	Reason for bihourly change in therapy intensity level (TIL)	1=Intensified: Clinical deterioration;2=Intensified: Suspicion of increased of ICP (not measured);3=Intensified: Increased ICP (documented);4=Intensified: Clinical decision to target other mechanism;5=Intensified: Change of doctor (different shift);6=Decreasing: Clinical improvement;7=Decreasing: Adequate control over ICP;8=Decreasing: Upper treatment limit reached/past;9=Decreasing: Further treatment considered futile;10=Decreasing: Change of doctor (different shift)
ICUAdmisStatusHaemoStable	TRUE	FALSE	integer	Reflects the status of the patient on admission to the ICU: Haemodynamically stable	0=No;1=Yes;88=Unknown
ICUAdmisStatusIntubated	TRUE	FALSE	integer	Reflects the status of the patient on admission to the ICU: Intubated	0=No;1=Yes;88=Unknown
ICUAdmisStatusMechVent	TRUE	FALSE	integer	Reflects the status of the patient on admission to the ICU: Mechanically ventilated	0=No;1=Yes;88=Unknown
Intubation	FALSE	TRUE	integer	This variable describes the absence or presence of Intubation as Ventilation Management (only for ICU patients).	0=No;1=Yes
MechVentilation	FALSE	TRUE	integer	This variable describes the absence or presence of Mechanical Ventilation (any respiratory mode except for CPAP).	0=No;1=Yes

Name	Static	Intervention	Format	Description	Possible Values
Nasogastric	FALSE	TRUE	integer	Reflects absence or presence of a Nasogastric feeding tube.	0=No;1=Yes
OxygenAdm	FALSE	TRUE	integer	Reflects presence or absence of Oxygen Administration.	0=No;1=Yes;88=Unknown
ParenteralNutrition	FALSE	TRUE	integer	These variables aim to document specific information on nutritional support, provided by parenteral and/or enteral routes. This variable describes the absence or presence of Parenteral Nutrition.	0=No;1=Yes
PEGTube	FALSE	TRUE	integer	This variable describes the absence or presence of a PEG tube (gastrostomy).	0=No;1=Yes
Reason	FALSE	FALSE	integer	Details on medication captured information on Class, Agent, Reason, Highest daily dose, Route, start and stop date and whether or not medication has been ongoing after discharge. This variable describes the Reason for medication. These details should be entered for each agent.	1=Sedatives/treatment of agitation: mechanical ventilation;2=Sedatives/treatment of agitation: metabolic suppression;3=Anti-epileptic: prophylaxis;4=Anti-epileptic: treatment of overt seizure;6=Anti-epileptic: treatment of (silent) seizure activity;7=Antibiotics: fever, no clear focus;8=Antibiotics: pneumonia;9=Antibiotics: urinary tract infection;10=Antibiotics: catheter related bloodstream infection;11=Antibiotics: intracranial abces/empyeme;12=Antibiotics: periprocedural prophylaxis;13=Antibiotics: meningitis;14=Anti-hypertensive: to lower blood pressure;15=Anti-hypertensive: treatment agitation;16=Calcium channel blockers: prevention of vasospasm;17=Calcium channel blockers: treatment of vasospasm;18=Calcium channel blockers: anti-hypertensive;19=Calcium channel blockers: cardiac indication;20=Steroids: traumatic brain injury;21=Steroids: ARDS;22=Steroids: hypopituitarism;23=Steroids: sepsis;24=Antacids: gastric protection;25=Antacids: reflux;26=H2 receptor antagonist: gastric protection;27=H2 receptor antagonist: treatment of ulcer;28=Proton pump inhibitors: gastric protection;29=Proton pump inhibitors: treatment of ulcer;30=Prokinetics: gastric retention;31=Prokinetics: vomiting;32=Prokinetics: constipation;33=Prokinetics: routine care;35=Analgesic: other;36=Neuromuscular blockade: other;37=Sedatives/treatment of agitation: Other;38=Anti-epileptic: Other;39=Antibiotics: Other;40=Anti-hypertensive: Other;41=Calcium channel blockers: Other;42=Steroids: Other;43=Antacids: Other;44=H2 receptor antagonist: Other;45=Proton pump inhibitors: Other;46=Prokinetics: Other;99=Other, specify in Agent Other: Other
ReasonOther	FALSE	FALSE	text	Details on medication captured information on Class, Agent, Reason, Highest daily dose, Route, start and stop date and whether or not medication has been ongoing after discharge. This variable describes the Reason for medication if this was "other than the predefined ones. These details should be entered for each agent.	Not Applicable
ReIntubation	FALSE	TRUE	integer	Reflects if there has been a need for re-intubation.	0=No;1=Yes
ReMechVentilation	FALSE	TRUE	integer	Reflects the need for re-instituting mechanical ventilation.	0=No;1=Yes
ReMechVentilationReason	FALSE	FALSE	integer	Reflects the reason for the need of re-instituting mechanical ventilation.	1=Respiratory failure;2=Neurologic deterioration;3=Spontaneous hyperventilation;4=Sepsis;99=Other
ReMechVentilationReasonOther	FALSE	FALSE	text	Reflects the "other" reason for the need of re-instituting mechanical ventilation.	Not Applicable
Route	FALSE	TRUE	text	Details on medication captured information on Class, Agent, Reason, Highest daily dose, Route, start and stop date and whether or not medication has been ongoing after discharge. This variable describes the Route. These details should be entered for each agent.	ED=Epidural;Ih=Inhaled;Im=Intramuscular;IvCont=Continuous IV;IvInt=Intermittent IV;PO=Oral;Pv=Vaginal;Re=Rectal;Sc=Subcutaneous;To=Topical
TILCCSFDrainageVolume	FALSE	TRUE	integer	Specification of volume drained, only applicable if "DailyTIL.TILCSFDrainage" was "yes"	Not Applicable
TILCSFDrainage	FALSE	TRUE	integer	Daily TIL: reflects if CSF drainage occurred yes or no	0=No;1=Yes
TILDobutamineDose	FALSE	TRUE	text	Indicates the total dose of dobutamine administered (in mg.) if applicable. Calculated over a 24-hour period	Not Applicable
TILDopamineDose	FALSE	TRUE	text	Indicates the total dose of dopamine administered (in mg.) if applicable. Calculated over a 24-hour period	Not Applicable

Name	Static	Intervention	Format	Description	Possible Values
TILFactorsCaloricIntakeEnteralKcal	FALSE	TRUE	integer	Daily TIL: reflects the caloric intake via Enteral route in Kcal	Not Applicable
TILFactorsCaloricIntakeParenKcal	FALSE	TRUE	integer	Daily TIL: reflects the caloric intake via Parenteral route in Kcal	Not Applicable
TILFactorsCaloricIntakeRouteEnteral	FALSE	TRUE	integer	The variables "parenteral" and "enteral route" are used to document if the patient received enteral feeding or not and if so, what the total number of Kcal was given by each route.	1=Parenteral route;2=Enteral route
TILFactorsCaloricIntakeRouteParen	FALSE	TRUE	integer	The variables "parenteral" and "enteral route" are used to document if the patient received enteral feeding or not and if so, what the total number of Kcal was given by each route.	0=No;1=Yes
TILFactorsCoagulation	FALSE	TRUE	integer	A maximum of 4 "types" of treatment (drop down box) can be selected and entered under treatment 1-4. Details on volume/dose of the products administered should correspond to treatment 1-4 and be entered in the variable volume 1-4.	0=No;1=Yes, for clinical reasons;2=Yes, according to study protocol;88=Unknown
TILFactorsCoagulationHemoglobinAfter	FALSE	FALSE	decimal	Reflects the level of hemoglobin after transfusion in the standard unit (g/dL)	Not Applicable
TILFactorsCoagulationHemoglobinBefore	FALSE	FALSE	decimal	Reflects the level of hemoglobin before transfusion in the standard unit (g/dL)	Not Applicable
TILFactorsCoagulationType1	FALSE	TRUE	integer	Reflects the details on volume/dose of the products administered	1=Packed red blood cell concentrates (pRBCs);2=Fresh whole blood;3=Fresh frozen plasma (FFP);4=Freeze dried plasma / lypholized plasma;5=Platelet concentrates;6=PCC (prothrombin complex concentrates);7=Fibrinogen concentrate;8=Albumine;9=Recombinant factor FVIIa;10=Tranexamic acid (TXA);11=Cryoprecipitate;12=Desmopression (DDAVP);13=Factor XIII;14=Calcium
TILFactorsCoagulationType2	FALSE	TRUE	integer	Reflects the details on volume/dose of the products administered	1=Packed red blood cell concentrates (pRBCs);2=Fresh whole blood;3=Fresh frozen plasma (FFP);4=Freeze dried plasma / lypholized plasma;5=Platelet concentrates;6=PCC (prothrombin complex concentrates);7=Fibrinogen concentrate;8=Albumine;9=Recombinant factor FVIIa;10=Tranexamic acid (TXA);11=Cryoprecipitate;12=Desmopression (DDAVP);13=Factor XIII;14=Calcium
TILFactorsCoagulationType3	FALSE	TRUE	integer	Reflects the details on volume/dose of the products administered	1=Packed red blood cell concentrates (pRBCs);2=Fresh whole blood;3=Fresh frozen plasma (FFP);4=Freeze dried plasma / lypholized plasma;5=Platelet concentrates;6=PCC (prothrombin complex concentrates);7=Fibrinogen concentrate;8=Albumine;9=Recombinant factor FVIIa;10=Tranexamic acid (TXA);11=Cryoprecipitate;12=Desmopression (DDAVP);13=Factor XIII;14=Calcium
TILFactorsCoagulationType4	FALSE	TRUE	integer	Reflects the details on volume/dose of the products administered	1=Packed red blood cell concentrates (pRBCs);2=Fresh whole blood;3=Fresh frozen plasma (FFP);4=Freeze dried plasma / lypholized plasma;5=Platelet concentrates;6=PCC (prothrombin complex concentrates);7=Fibrinogen concentrate;8=Albumine;9=Recombinant factor FVIIa;10=Tranexamic acid (TXA);11=Cryoprecipitate;12=Desmopression (DDAVP);13=Factor XIII;14=Calcium
TILFactorsCoagulationVolume1	FALSE	TRUE	text	Reflects the details on volume/dose of the products administered	Not Applicable
TILFactorsCoagulationVolume2	FALSE	TRUE	text	Reflects the details on volume/dose of the products administered	Not Applicable
TILFactorsCoagulationVolume3	FALSE	TRUE	text	Reflects the details on volume/dose of the products administered	Not Applicable
TILFactorsCoagulationVolume4	FALSE	TRUE	text	Reflects the details on volume/dose of the products administered	Not Applicable
TILFactorsGlucoseManagement	FALSE	TRUE	integer	Indicates whether glucose management was applied and if so, which therapy used (prophylactic, insulin administration of tight glycemic control).	0=No specific therapy;1=Prophylactic;2=Insulin administration to correct hyperglycemias;3=Tight glycemic control (targeting blood glucose levels of 80-110mg/dL [4.4-6.1mmol/L])
TILFever	FALSE	TRUE	integer	Records for the Daily TIL whether there was treatment of fever (temperature <38¬∞C) or spontaneous temperature of 34.5¬∞C	0=No;1=Yes
TILFeverHypothermia	FALSE	FALSE	integer	Records for the Daily TIL whether there was hypothermia below 35¬∞C	0=No;1=Yes

Name	Static	Intervention	Format	Description	Possible Values
TILFeverMildHypothermia	FALSE	FALSE	integer	Records for the Daily TIL whether there was mild hypothermia for ICP control with a lower limit of 35¬∞C.	0=No;1=Yes
TILFluidColloids	FALSE	TRUE	integer	Indicates whether colloids were administered with regard to Fluid Balance.	0=No;1=Yes;88=Unknown
TILFluidColloidsTotalVolume	FALSE	TRUE	integer	Total volume of colloids administered (in ml)	Not Applicable
TILFluidColloidsType	FALSE	TRUE	integer	Type of colloids administered	1=Albumin 5%;2=Albumin 20%;3=Dextran;4=Gelatin (e.g. gelofusion);5=HES (hydroxyethyl starches);6=Tetrastarches (e.g. HES 130/04)
TILFluidIn	FALSE	TRUE	integer	Recorded preferably over 24-hour period, exact details to be derived from start and stop date/time for calculation	Not Applicable
TILFluidLoading	FALSE	TRUE	integer	Records for the Daily TIL whether there was fluid loading for maintenance of cerebral perfusion.	0=No;1=Yes
TILFluidLoadingVasopressor	FALSE	FALSE	integer	Records for the Daily TIL whether there was vasopressor therapy required for management of cerebral perfusion	0=No;1=Yes
TILFluidOutCSFDrain	FALSE	TRUE	integer	Daily TIL - Number of fluid out: CSF drainage in ml	Not Applicable
TILFluidOutGastric	FALSE	FALSE	integer	Daily TIL - Number of fluid out: Gastic loss in ml	Not Applicable
TILFluidOutOther	FALSE	FALSE	integer	Daily TIL - Number of fluid out: other fluid (than Urine, Gastic loss or CSF drainage) in ml	Not Applicable
TILFluidOutUrine	FALSE	FALSE	integer	Daily TIL - Number of fluid out: Urine in ml	Not Applicable
TILFluidsRenalReplacement	FALSE	FALSE	integer	Indicates for the fluid balance whether there was a need for renal replacement therapy.	0=No;1=Yes
TILHyperosmolarThearpy	FALSE	TRUE	integer	Records for the Daily TIL whether there was Hyperosmolar therapy with mannitol up to 2 g/kg/24 hours	0=No;1=Yes
TILHyperosomolarTherapyHigher	FALSE	TRUE	integer	Records for the Daily TIL whether there was Hyperosmolar therapy with hypertonic saline > 0.3 g/kg/24 hours	0=No;1=Yes
TILHyperosomolarTherapyHypertonicLow	FALSE	TRUE	integer	Records for the Daily TIL whether there was Hyperosmolar therapy with hypertonic saline up to 0.3 g/kg/24 hours	0=No;1=Yes
TILHyperosomolarTherapyMannitolGreater2g	FALSE	TRUE	integer	Records for the Daily TIL whether there was Hyperosmolar therapy with mannitol > 2 g/kg/24 hours	0=No;1=Yes
TILHypertonicSalineDose	FALSE	TRUE	text	Indicates the total dose of hypertonic saline administered (in g.) if applicable. Calculated over a 24-hour period.	Not Applicable
TILHyperventilation	FALSE	TRUE	integer	Records for the Daily TIL whether there was Mild hypocapnia for ICP control [PaCO2 4.6 - 5.3 kPa (35 - 40 mmHg)]	0=No;1=Yes
TILHyperventilationIntensive	FALSE	TRUE	integer	Records for the Daily TIL whether there was Intensive hypocapnia for ICP control [PaCO2 < 4.0 kPa (30 mmHg)]	0=No;1=Yes
TILHyperventilationModerate	FALSE	TRUE	integer	Records for the Daily TIL whether there was Moderate hypocapnia for ICP control [PaCO2 4.0 - 4.5 kPa (30 - 35 mmHg)]	0=No;1=Yes
TILMannitolDose	FALSE	TRUE	text	Indicates the total dose of Mannitol administered (in g.) if applicable. Calculated over a 24-hour period	Not Applicable
TILNoradrenalineDose	FALSE	TRUE	text	Indicates the total dose of Noradrenaline administered (in mg.) if applicable. Calculated over a 24-hour period	Not Applicable
TILOtherDose	FALSE	TRUE	text	Indicates the dose (in mg.) of other vasopressors drugs administered (if applicable)	Not Applicable
TILOtherTxt	FALSE	TRUE	text	Indicates which other vasopressors drugs was administered (if applicable)	Not Applicable
TILOtherVaso	FALSE	TRUE	integer	Indicates whether any other Vasopressor drug was administered (other than Dobutamine, Dopamine, Noradrenaline or Phenylephrine)	0=No;1=Yes
TILPhenylephrineDose	FALSE	TRUE	text	Indicates the total dose of phenylephrine administered (in mg.) if applicable. Calculated over a 24-hour period	Not Applicable

Name	Static	Intervention	Format	Description	Possible Values
TILPhysicianConcernsContusionpregression	FALSE	FALSE	integer	Reflects daily the physician concern and satisfaction with regard to contusion progression. Scales from 1 (not concerned) to 10 (very concerned)	1=1;2=2;3=3;4=4;5=5;6=6;7=7;8=8;9=9;10=10
TILPhysicianConcernsEpilepsy	FALSE	FALSE	integer	Reflects daily the physician concern and satisfaction with regard to epilepsy. Scales from 1 (not concerned) to 10 (very concerned)	1=1;2=2;3=3;4=4;5=5;6=6;7=7;8=8;9=9;10=10
TILPhysicianConcernsFocalSwelling	FALSE	FALSE	integer	Reflects daily the physician concern and satisfaction with regard to focal swelling. Scales from 1 (not concerned) to 10 (very concerned)	1=1;2=2;3=3;4=4;5=5;6=6;7=7;8=8;9=9;10=10
TILPhysicianConcernsHematomaProgression	FALSE	FALSE	integer	Reflects daily the physician concern and satisfaction with regard to hematoma progression. Scales from 1 (not concerned) to 10 (very concerned)	1=1;2=2;3=3;4=4;5=5;6=6;7=7;8=8;9=9;10=10
TILPhysicianConcernsHypoperfusion	FALSE	FALSE	integer	Reflects daily the physician concern and satisfaction with regard to suspected hypoperfusion. Scales from 1 (not concerned) to 10 (very concerned)	1=1;2=2;3=3;4=4;5=5;6=6;7=7;8=8;9=9;10=10
TILPhysicianConcernsIntracranialInfection	FALSE	FALSE	integer	Reflects daily the physician concern and satisfaction with regard to intracranial infections. Scales from 1 (not concerned) to 10 (very concerned)	1=1;2=2;3=3;4=4;5=5;6=6;7=7;8=8;9=9;10=10
TILPhysicianConcernsVasospasm	FALSE	FALSE	integer	Reflects daily the physician concern and satisfaction with regard to vasospasm. Scales from 1 (not concerned) to 10 (very concerned)	1=1;2=2;3=3;4=4;5=5;6=6;7=7;8=8;9=9;10=10
TILPhysicianOverallSatisfaction	FALSE	FALSE	integer	This variable aims to capture the overall satisfaction of the physician with the clinical course of this patient; "not at all satisfied" would indicate that the patient did much more poorly than expected; "very satisfied" would indicate that the patient did much better than expected. Physician satisfaction should be assessed on a daily basis,	0=Not at all;1=Slightly;2=Moderately;3=Quite;4=Very
TILPhysicianOverallSatisfactionSurvival	FALSE	FALSE	integer	This variable aims to capture the opinion of the treating physician as to whether the short time survival change have chnged in comparision to the previous assessment	1=Much worse;2=A little worse;3=Unchanged;4=A little better;5=Much better
TILPosition	FALSE	TRUE	integer	Records for the Daily TIL whether there was head elevation for ICP control	0=No;1=Yes
TILPositionNursedFlat	FALSE	TRUE	integer	Records for the Daily TIL whether there was a patient position of Nursed flat (180¬∞C) for CPP management	0=No;1=Yes
TILReasonForChange	FALSE	FALSE	integer	Reflects the reason for change in TIL therapy over the day.	0=No change;1=Intensified: Clinical deterioration;2=Intensified:Suspicion of increased of ICP (not measured);3=Intensified:Increased ICP (documented);4=Intensified:Clinical decision to target other mechanism;5=Intensified:Change of doctor (different shift);6=Decreasing:Clinical improvement;7=Decreasing:Adequate control over ICP;8=Decreasing:Upper treatment limit reached/past;9=Decreasing:Further treatment considered futile;10=Decreasing:Change of doctor (different shift)
TILSedation	FALSE	TRUE	integer	Records for the Daily TIL whether there was sedation (low dose as required for mechanical ventilation)	0=No;1=Yes
TILSedationHigher	FALSE	TRUE	integer	Records for the Daily TIL whether there was a higher dose sedation for ICP control (not aiming for burst supression)	0=No;1=Yes
TILSedationMetabolic	FALSE	TRUE	integer	Records for the Daily TIL whether there was metabolic suppression for ICP control with high dose barbiturates or propofol	0=No;1=Yes
TILSedationNeuromuscular	FALSE	TRUE	integer	Records for the Daily TIL whether there was neuromuscular blockade (paralysis)	0=No;1=Yes
TILSedationScaleUsed	FALSE	TRUE	integer	Reflects with regard to sedation management whether a sedation scale (SAS, RASS, MASS, Ramsay, etc) was used to adjust sedatives?	0=No;1=Yes;77=N/A
TILSedativesInterrupted	FALSE	TRUE	integer	Reflects with regard to sedation management whether infusions of sedatives (opioids) were interrupted during the day if the patient was receiving any.	0=No;1=Yes;77=N/A

Name	Static	Intervention	Format	Description	Possible Values
TotalTIL	FALSE	TRUE	integer	Calculated centrally - 24 hour TILS as the worst sum TILs for each day for the ICU timepoints (day 1-7, 10, 14, 21 and 28)	Not Applicable
Tracheostomy	FALSE	TRUE	integer	Describes absence or presence of a Tracheostomy.	0=No;1=Yes
Transfer	FALSE	FALSE			Not Applicable
TransReason	FALSE	FALSE	integer	WHY question: documents reason for transition of care	1=Mechanical ventilation;2=Frequent neurological observations;3=Haemodynamic invasive monitoring;4=Extracranial injuries;5=Neurological operation;6=Clinical deterioration;7=CT abnormalities;8=Clinical observation for TBI;9=No ICU bed available;10=Could be discharged home, but no adequate supervision;11=Improvement;12=Neurological deterioration;13=Systemic compilation;14=CT progression;15=Planned surgery;16=Condition stable;17=(acute) Treatment goals accomplished;18=Need to free a bed;19=Further improvement;20=Clinical rehab completed;21=Lack of improvement;22=Late neurological deterioration;23=Problems unrelated to trauma;24=Post operative care;25=Neurological complication;99=Other
TransTiming	FALSE	FALSE	text	Transitions of care may be premature or delayed because of logistic problems. This variable aims to capture such information.	APP=Appropriate;DEL=Delayed;PRE=Premature
UrineCath	FALSE	TRUE	integer	Describes absence or presence of an Urinary catheter.	0=No;1=Yes

ICU vitals and assessments

Name	Static	Intervention	Format	Description	Possible Values
DVAssmtConditions	FALSE	FALSE	integer	Reflects the assessment condition for the best GCS assessments as part of the daily vitals. Only applicable to ICU stratum.	0=No sedation or paralysis;1=Sedated;2=Paralyzed;3=Temporary stop of sedation/paralysis;4=Reversal of sedation/paralysis;5=Active reversal (pharmacologic) of sedation/paralysis;99=Other
DVAssmtConditionsWorst	FALSE	FALSE	integer	Reflects the assessment condition for the worst GCS assessments as part of the daily vitals. Only applicable to ICU stratum.	0=No sedation or paralysis;1=Sedated;2=Paralyzed;3=Temporary stop of sedation/paralysis;4=Reversal of sedation/paralysis;5=Active reversal (pharmacologic) of sedation/paralysis;99=Other
DVBestPupilSymmetry	FALSE	FALSE	integer	Reflects if the pupil are of equal size (symmetry) during the day as part of the Daily vitals.	1=Equal;2=Unequal R>L;3=Unequal L>R
DVBloodOxySatHigh	FALSE	FALSE	decimal	Reflects Highest oxygen saturation (in %) as determined from Arterial Blood Gas	Not Applicable
DVBloodOxySatLow	FALSE	FALSE	decimal	Reflects Lowest oxygen saturation (in %) as determined from Arterial Blood Gas	Not Applicable
DVChangeCauseWorst	FALSE	FALSE	integer	Reflects the cause of change for the worst GCS as part of the Daily Vitals.	1=Mainly intracranial;2=Mainly extracranial;3=Both to an equal extent;88=Unknown
DVChangeInOneDay	FALSE	FALSE	integer	Reflects presence or absence of change over past 24 hours for the Best GCS as part of the Daily Vitals.	0=No change;1=Improving;2=Episode of deterioration;3=Sustained deterioration;4=Fluctuating
DVChangeInOneDayWorst	FALSE	FALSE	integer	Reflects presence or absence of change over past 24 hours for the Worst GCS as part of the Daily Vitals.	0=No change;1=Improving;2=Episode of deterioration;3=Sustained deterioration;4=Fluctuating
DVDBP	FALSE	FALSE	integer	The diastolic blood pressure at the timepoint of the highest systolic blood pressure.	Not Applicable
DVDBPLow	FALSE	FALSE	integer	The diastolic blood pressure at the timepoint of the lowest systolic blood pressure.	Not Applicable
DVFiO2AtHighPaO2	FALSE	FALSE	decimal	FiO2 at time of sampling - Highest PaO2/FiO3 (in %)	Not Applicable
DVFiO2AtLowPaO2	FALSE	FALSE	decimal	FiO2 at time of sampling - Lowest PaO2/FiO3 (in %)	Not Applicable
DVFourScoreBrainstem	FALSE	FALSE	integer	Four Score for the Brainstem reflexes	0=Absent pupil, corneal;1=Pupil and corneal reflexes absent;2=Pupil or corneal reflexes absent;3=One pupil wide and fixed;4=Pupil and corneal reflexes present;88=Unknown
DVFourScoreEye	FALSE	FALSE	integer	Four Score for the Eye response	0=Eyelids remain closed with pain;1=Eyelids closed but opens to pain;2=Eyelids closed but opens to loud voice;3=Eyelids open but not tracking;4=Eyelids open or opened tracking or blinking to command;88=Unknown
DVFourScoreMotor	FALSE	FALSE	integer	Four Score for the Motor response	0=No response to pain or generalized myoclonus status epilepticus;1=Extensor posturing;2=Flexion response to pain;3=Localizing to pain;4=Thumbs up, fist;88=Unknown
DVFourScoreRespiration	FALSE	FALSE	integer	Four Score for the Respiration	0=Breathes at ventilator rate or apnea;1=Breathes above ventilator rate;2=Not intubated, irregular breathing pattern;3=Not intubated, Cheyne- Stokes breathing pattern;4=Not intubated, regular breathing pattern;88=Unknown
DVFourScoreTotal	FALSE	FALSE	integer	DVFourScoreEye + DVFourScoreMotor + DVFourScoreBraintstem +	Not Applicable

Name	Static	Intervention	Format	Description	Possible Values
				DVFourScoreRespiration. No score if any of the components =88 (unknown) or NULL	
DVGCSBest	FALSE	FALSE	integer	Reflects if the Worst GCS is the same as the best GCS as part of the Daily Vitals.	0=No;1=Yes
DVGCSBestChangeCause	FALSE	FALSE	integer	Cause of change for the Best GCS as part of the Daily Vitals.	1=Mainly intracranial;2=Mainly extracranial;3=Both to an equal extent;88=Unknown
DVGCSEyes	FALSE	FALSE	text	Best GCS eye opening as part of the Daily Vitals.	1=1-None;2=2-To pain;3=3-To speech;4=4-Spontaneously;O=Untestable (Other);S=Untestable (swollen);UNK=Unknown
DVGCSMotor	FALSE	FALSE	text	Best GCS motor score as part of the Daily Vitals.	1=1-None;2=2-Abnormal extension;3=3-Abnormal flexion;4=4-Normal flexion/withdrawal;5=5-Localizes to pain;6=6-Obeys command;O=Untestable (Other);P=Untestable (Deep sedation/paralyzed);UN=Unknown
DVGCSScore	FALSE	FALSE	text	Best GCS Score: DVGCSEyes + DVGCSMotor + DVGCSVerbal. If one or more of these is Untestable or unknown then = "No Sum"	3=3;4=4;5=5;6=6;7=7;8=8;9=9;10=10;11=11;12=12;13=13;14=14;15=15;No Sum=Untestable/Unknown
DVGCSVerbal	FALSE	FALSE	text	Best GCS verbal score as part of the daily vitals.	1=1-None;2=2- Incomprehensible sound;3=3-Inappropriate words;4=4-Confused;5=5-Oriented;O=Untestable (Other);T=Untestable (Tracheotomy/endotracheal tube);UN=Unknown
DVGCSWorstEyes	FALSE	FALSE	text	Worst GCS eye opening as part of the daily vitals.	1=1-None;2=2-To pain;3=3-To speech;4=4-Spontaneously;O=Untestable (Other);S=Untestable (swollen);UNK=Unknown
DVGCSWorstMotor	FALSE	FALSE	text	Worst GCS motor score as part of the daily vitals.	1=1-None;2=2-Abnormal extension;3=3-Abnormal flexion;4=4-Normal flexion/withdrawal;5=5-Localizes to pain;6=6-Obeys command;O=Untestable (Other);P=Untestable (Deep sedation/paralyzed);UN=Unknown
DVGCSWorstScore	FALSE	FALSE	text	Worst GCS: DVGCSWorstEyes + DVGCSWorstMotor + DVGCSWorstVerbal. If one or more of these is Untestable or unknown then = "No Sum"	3=3;4=4;5=5;6=6;7=7;8=8;9=9;10=10;11=11;12=12;13=13;14=14;15=15;No Sum=Untestable/Unknown
DVGCSWorstVerbal	FALSE	FALSE	text	Worst GCS Verbal score as part of the daily vitals	1=1-None;2=2- Incomprehensible sound;3=3-Inappropriate words;4=4-Confused;5=5-Oriented;O=Untestable (Other);T=Untestable (Tracheotomy/endotracheal tube);UN=Unknown
DVHighestPaCO2	FALSE	FALSE	decimal	Highest PaCO2 on a day, in mmHg.	Not Applicable
DVHighestPaO2	FALSE	FALSE	decimal	Highest PaO2 on a day, in mmHg	Not Applicable
DVHighestPaO2OverHighestFiO2	FALSE	FALSE	decimal	FiO2 at time of sampling - Highest PaO2/FiO4	Not Applicable
DVHighestpH	FALSE	FALSE	decimal	Highest pH as part of the Daily vitals Blood Gas Analysis	Not Applicable
DVHR	FALSE	FALSE	integer	The highest heart rate during the day.	Not Applicable
DVHRLow	FALSE	FALSE	integer	The lowest heart rate of a day.	Not Applicable
DVLowestPaCO2	FALSE	FALSE	decimal	The lowest value of PaCO2 measured on each day, in mmHg.	Not Applicable
DVLowestPaO2	FALSE	FALSE	decimal	Lowest PaO2 in mmHg as part of the Daily Vitals Blood Gas Analysis	Not Applicable
DVLowestPaO2OverLowestFiO2	FALSE	FALSE	decimal	FiO2 at time of sampling - Lowest PaO2/FiO4 as part of the Daily Vitals	Not Applicable
DVLowestpH	FALSE	FALSE	decimal	Lowest pH as part of the Daily Vitals Blood Gas Analysis	Not Applicable
DVPupilLftEyeMeasr	FALSE	FALSE	integer	Best Pupil Left eye size	Not Applicable
DVPupilReactivityLghtLftEyeReslt	FALSE	FALSE	integer	The best pupillary reactivity of the left eye during the day	1=+ (Brisk);2=+ (Sluggish);3=- (Negative)
DVPupilReactivityLghtRtEyeReslt	FALSE	FALSE	integer	Best pupillary reactivity of the right eye during the day	1=+ (Brisk);2=+ (Sluggish);3=- (Negative)
DVPupilRtEyeMeasr	FALSE	FALSE	integer	Best Pupil Right eye Size	Not Applicable
DVSBP	FALSE	FALSE	integer	The highest systolic blood pressure for each day where vitals are recorded.	Not Applicable
DVSBPLow	FALSE	FALSE	integer	The lowest systolic blood pressure for each day.	Not Applicable
DVSpO2	FALSE	FALSE	integer	Highest Oxygen saturation (pulse oximetry) in % for each day where vitals are recorded.	Not Applicable
DVSpO2Low	FALSE	FALSE	integer	Lowest Oxygen saturation (pulse oximetry) in % for each day where vitals are recorded.	Not Applicable

Name	Static	Intervention	Format	Description	Possible Values
DVTempHighC	FALSE	FALSE	decimal	The highest body temperature of the day in degrees Celsius. For the associated time point, see Vitals.DVTempHighDateTime. For location of where the temperature was measured, see Vitals.DVTempLocation.	Not Applicable
DVTempLocation	FALSE	FALSE	integer	Location of thermometer probe for temperature measurement.	1=External-axillary;2=External-skin;3=Core- rectal;4=Core- bladder;5=Core- oesophageal;6=Core- tympanic;7=Core- nasopharynx
DVTempLowC	FALSE	FALSE	decimal	The lowest temperature of a day in degrees Celsius. For the timepoint, see Vitals.DVTempLowDateTime. For location of where the temperature was measured, see Vitals.DVTempLocation.	Not Applicable
DVWorstPupilLftEyeMeasr	FALSE	FALSE	integer	Worst Pupils Left eye Size	Not Applicable
DVWorstPupilReactivityLghtLftEyeResult	FALSE	FALSE	integer	Worst Pupils Left eye Reactivity	1=+ (Brisk);2=+ (Sluggish);3=- (Negative)
DVWorstPupilReactivityLghtRghtEyeResult	FALSE	FALSE	integer	Worst Pupils Right eye Reactivity	1=+ (Brisk);2=+ (Sluggish);3=- (Negative)
DVWorstPupilRghtEyeMeasr	FALSE	FALSE	integer	Worst Pupils Right eye Size	Not Applicable
DVWorstPupilSymmetry	FALSE	FALSE	integer	Worst Pupil symmetry	1=Equal;2=Unequal R>L;3=Unequal L>R
HosComplEventHypocapnia	FALSE	FALSE	integer	Reflects Inadvertent hypocapnia. Second Insults reported in Vitals relate to the the hospital phase (both ward and ICU). Inadvertent hypocapnia is defined as a PaCO2 <=3.3 kPa (25 mmHg)	0=No;1=Single episode, short duration;2=Multiple episodes or prolonged duration;88=Unknown
HosComplEventHypotension	FALSE	FALSE	integer	Reflects Hypotensive Episodes. Second Insults reported in Vitals relate to the the hospital phase (both ward and ICU). Pre-hospital hypoxia is documented at: InjuryHx.EDComplEventHypotension. Definite hypotension is defined as a documented systolic BP < 90 mm Hg (adults)	0=No;1=Single episode, short duration;2=Multiple episodes or prolonged duration;88=Unknown
HosComplEventHypoxia	FALSE	FALSE	integer	Reflects Hypoxic Episodes. Second Insults reported in Vitals relate to the hospital phase (both ward and ICU). Pre-hospital hypoxia is documented at: InjuryHx.EDComplEventHypoxia. Definite hypoxia is defined as a documented PaO2 <8 kPa (60 mm Hg) and/or SaO2<90%	0=No;1=Single episode, short duration;2=Multiple episodes or prolonged duration;88=Unknown
HosComplEventSeizures	FALSE	FALSE	integer	Reflects absence or presence of seizures as Intracranial Second Insult. Second Insults reported in Vitals relate to the the hospital phase (both ward and ICU).	0=No;1=Single episode, short duration;2=Partial/Focal;3=Generalized;4=Status epilepticus;5=Silent seizure activity (only electrical, no clinical manifestation);88=Unknown
HospSecondInsultsNeuroWorse	FALSE	FALSE	integer	The importance of Neuroworsening was first described by Morris and Marshall. The occurrence of neuroworsening is related to poorer outcome in subjects with moderate to severe TBI. Neuroworsening is defined as 1) a decrease in GCS motor score of 2 or more points; 2) a new loss of pupillary reactivity or development of pupillary assymmetry >= 2mm; 3) deterioration in neurological or CT status sufficient to warrant immediate medical or surgical intervention	0=No;1=Yes;88=Unknown
HospSecondInsultsNeuroWorseYes1	FALSE	FALSE	integer	This variable provides a specification of the type of neuroworsening if it occurs.	1=Decrease in motor score >= 2 points; 2=Development of pupillary abnormalities; 3=Other neurological and/or CT deterioration
HospSecondInsultsNeuroWorseYes2	FALSE	FALSE	integer	This variable provides a specification of the type of neuroworsening if it occurs.	1=Decrease in motor score >= 2 points; 2=Development of pupillary abnormalities; 3=Other neurological and/or CT deterioration
HospSecondInsultsNeuroWorseYes3	FALSE	FALSE	integer	This variable provides a specification of the type of neuroworsening if it occurs.	1=Decrease in motor score >= 2 points; 2=Development of pupillary abnormalities; 3=Other neurological and/or CT deterioration
HourlyValueAccurate	FALSE	FALSE	integer	Are the measured values for ICP over this day considered to be accurate? An explanation can be found in HourlyValues.HourlyValueNotAccurateProblems. Associated with the date in HourlyValues.HVDate.	0=No;1=Yes;2=Doubtful
HourlyValueLevelABP	FALSE	FALSE	integer	Differences in level of zeroing the arterial blood pressure transducer may affect	1=Right atrium;2=Level of arterial catheter;99=Other

Name	Static	Intervention	Format	Description	Possible Values
				calculations of CPP and comparisons between centres.	
HourlyValueLevelABPOther	FALSE	FALSE	text	A free text description of the level of ABP transducer if HourlyValues.HourlyValueLevelABP is "other".	Not Applicable
HourlyValueNotAccurateProblems	FALSE	FALSE	text	Explanation in free text of the variable HourlyValues.HourlyValueAccurate. Associated with the date in HourlyValues.HVDate.	Not Applicable
HVDBP	FALSE	FALSE	integer	Bihourly diastolic blood pressure	Not Applicable
HVSBP	FALSE	FALSE	integer	Bihourly systolic blood pressure	Not Applicable

Surgery and neuromonitoring

Name	Static	Intervention	Format	Description	Possible Values
DecompressiveCran	TRUE	FALSE	integer	These variables specifically focus on decompressive craniectomy (DC). Considerable uncertainty exists on which patients may benefit from DC, as well as on the timing. Previous studies have shown that in approximately one third of cases the DC was too small in size.	0=No;1=Yes
DecompressiveCranLocation	TRUE	FALSE	integer	Documents type of decompressive craniectomy.	1=Bifrontal;2=Hemicraniectomy- left side;3=Hemicraniectomy- right side;4=Posterior fossa
DecompressiveCranReason	TRUE	FALSE	integer	WHY question: documents main reason for performing DC	1=Pre-emptive approach to treatment of (suspected) raised ICP (not last resort);2=Raised ICP, refractory to medical management (last resort);3=ICP not monitored, but CT evidence of raised ICP;4=Not directly planned, but decided on because of intra-operative brain swelling;5=Routinely performed with every ASDH or Contusion evacuation;6=Development of cerebral infarction
DecompressiveCranType	TRUE	FALSE	integer	The indications for the decompressive craniectomy can be documented here.	1=Isolated procedure;2=In association with ASDH removal;3=In association with contusion/ICH removal;4=In association with ASDH and contusion/ICH removal
DecompressiveSize	TRUE	FALSE	integer	In some sites, the size of DC was recorded by Investigators. No generable way of calculating was proposed.	Not Applicable
DVBrainTempCLow	FALSE	FALSE	decimal	Brain temperature at the timepoint of the lowest body temperature of the day, degrees Celsius.	Not Applicable
DVTempBrainC	FALSE	FALSE	decimal	Brain temperature at the timepoint of the highest body temperature of the day, degrees Celsius.	Not Applicable
HourlyValueICPDiscontinued	FALSE	TRUE	integer	Answer to the question: "Was active ICP treatment discontinued (due to poor prognosis)?"	0=No;1=Yes
HourlyValueLevelICP	FALSE	FALSE	integer	Only applicable in case ICP monitored; Differences in level of zeroing ICP may affect calculation of CPP and comparisons between centres.	1=Foramen of Monro;2=Same level as ABP;3=Meatus externus (ear)
HVCPP	FALSE	FALSE	integer	Bihourly cerebral perfusion pressure	Not Applicable
HVICP	FALSE	FALSE	integer	Bihourly intracranial pressure	Not Applicable
ICPMonitorStop	FALSE	TRUE	integer	If ICP monitoring has stopped or not. Time and date is found in Hospital.ICPRemTime and Hospital.ICPRemDate. Reason for stopping ICP monitoring is found in Hospital.ICPStopReason.	0=No;1=Yes
ICPMonitorStopReasonOther	FALSE	FALSE	text	Specifies the "other" reason if the reason for stopping ICP was not on the pre-defined list. Check also "Hospital.ICPStopReason"	Not Applicable
ICUCatheterICP	FALSE	TRUE	integer	Reflects if the ICP catheter has been revised in case of ICP monitoring	0=No;1=Yes
ICUProblemsICP	FALSE	FALSE	integer	In case of brain specific monitoring in the ICU, all details on the ICP monitoring were recorded. This variable reflects Problems in ICP monitoring.	0=No;1=Yes
ICUProblemsICPYes	FALSE	FALSE	integer	In case of brain specific monitoring in the ICU, all details on the ICP monitoring were	1=Accidental catheter removal;2=Catheter obstruction/failure;3=Suspicion of inaccurate measurement

Name	Static	Intervention	Format	Description	Possible Values
				recorded. This variable explains the Problems in ICP monitoring if applicable.	
ICURaisedICP	FALSE	FALSE	integer	In case of brain specific monitoring in the ICU, all details on the ICP monitoring were recorded. This variable reflects if there was Raised ICP (sustained).	0=No;1=Yes, controlled;2=Yes, refractory
ICUReasonForTypeICPMont	FALSE	FALSE	integer	In case of brain specific monitoring in the ICU, all details on the ICP monitoring were recorded. This variable reflects WHY Question: reason for choosing ventricular monitor	1=Routine in our department;2=Not routine, but enlarged ventricles;3=No parenchymal device available;99=Other
ICUReasonForTypeICPMontPare	FALSE	FALSE	integer	In case of brain specific monitoring in the ICU, all details on the ICP monitoring were recorded. This variable reflects WHY Question: reason for choosing parenchymal monitor.	1=Routine in our department;2=Not routine, but small ventricles;3=Mainly motivated by time of day;4=No OR available for placement ventr. catheter;5=Failed implantation ventricular catheter;99=Other
ICUReasonForTypeICUMontOther	FALSE	FALSE	text	In case of brain specific monitoring in the ICU, all details on the ICP monitoring were recorded. This variable reflects "Other Reason of choice for ventricular/ventricular+sensor monitoring"	Not Applicable
ICUReasonForTypeICUMontParOther	FALSE	FALSE	text	In case of brain specific monitoring in the ICU, all details on the ICP monitoring were recorded. This variable reflects 'Other Reason of choice for parenchymal sensor'.	Not Applicable
ICUReasonICP	FALSE	FALSE	integer	In case of brain specific monitoring in the ICU, all details on the ICP monitoring were recorded. This variable reflects WHY Question: documents reason for monitoring ICP in patient admitted to ICU	1=Guideline criteria;2=Radiological signs raised ICP;3=Clinical suspicion raised ICP;4=Anaesthesia or mechanical ventilation required for extracranial injuries;5=To inform surgical indication for mass lesion;99=Other
ICUReasonICPOther	FALSE	FALSE	text	In case of brain specific monitoring in the ICU, all details on the ICP monitoring were recorded. This variable is a free text field for description of why the patient has ICP monitoring if there is another reason than pre-specified in Hospital.ICUReasonICP.	Not Applicable
ShortTermSurvivalNoSurg	FALSE	FALSE	integer	Aims to capture information on the surgeon's expectations, eg if the surgeon considers a realistic expectation of benefit, or performs the surgery as a "last resort" in a likely hopeless case --> The short term survival chances of the patient if I DO NOT operate (1-100)	Not Applicable
ShortTermSurvivalYesSurg	FALSE	FALSE	integer	Aims to capture information on the surgeon's expectations, eg if the surgeon considers a realistic expectation of benefit, or performs the surgery as a "last resort" in a likely hopeless case --> The short term survival chances of the patient if I DO operate (1-100)	Not Applicable
SurgeryCranialDelay	FALSE	FALSE	integer	Reflects reason for delay of cranial surgery (if any).	1=Transferral from other hospital;2=Haemodynamic instability;3=No OR available;4=Surgeon delayed;5=No delay;99=Other
SurgeryCranialReason	FALSE	FALSE	integer	WHY Question: aims to document the reason for intracranial surgery	1=Emergency/Life saving;2=Clinical deterioration;3=Mass effect on CT;4=Radiological progression;5=suspicion of) raised ICP;6=Guideline adherence;7=To prevent deterioration
SurgeryDescCranial	FALSE	TRUE	integer	The cranial surgeries information was to be entered in the e-CRF in tables for which you could add as many rows as you wish. The tables consisted of - Surgery start date - Surgery start time - Surgery end date - Surgery end time - Cranial surgery code - Reason - Delay - Short time survival if you do not operate - Short time survival if you do operate For the ‚Äúcode‚Äù there was a choice of 23 values in a drop-down box. For each row you entered in the table, you could select only 1 code in the drop-down. But, if a patient had several surgical options that were applicable, you could enter several rows with each the same start and end date, but marking the different applicable surgical codes in each row separately.	1=Aneurysm (non trauma);2=Acute subdural hematoma;3=Contusion;4=Craniofacial surgery;5=CSF shunt;6=Chronic subdural hematoma;7=Decompressive craniectomy-hemicraniectomy;8=Depressed skull fracture;9=Epidural hematoma;10=Intracerebral hematoma;11=Infection;12=Optic nerve decompression;13=Posterior fossa surgery;14=Skull base fracture;15=Ventriculostomy for CSF drainage;16=Debridement minimal for penetrating injuries;17=Debridement extensive for penetrating injuries;18=Foreign body removal;19=Bone flap replacement;20=Cranioplasty;21=Other;71=Decompressive craniectomy - bifrontal;72=Decompressive craniectomy - removal previous bone flap
SurgeryDescExtraCranial	FALSE	TRUE	integer	The extra-cranial surgeries information was to be entered in the e-CRF in tables for	22=Maxillofacial;23=Extremity fracture lower limb (internal fixation);24=Extremity fracture lower limb (external

Name	Static	Intervention	Format	Description	Possible Values
				which you could add as many rows as you wish. The tables consisted of - Surgery start date - Surgery start time - Surgery end date - Surgery end time - Extracranial surgery code - Reason - Delay - Short time survival if you do not operate - Short time survival if you do operate For the ‚Äúcode‚Äù there was a choice of 18 values in a drop-down box. For each row you entered in the table, you could select only 1 code in the drop-down. But, if a patient had several surgical options that were applicable, you could enter several rows with each the same start and end date, but marking the different applicable surgical codes in each row separately.	fixation);25=Extremity fracture upper limb (internal fixation);26=Extremity fracture upper limb (external fixation);27=Fasciotomy;28=Laparotomy (abdomen);29=Pelvic fracture (internal fixation);30=Pelvic fracture (external fixation);31=Spinal stabilisation/cervical;32=Spinal stabilisation/thoracic;33=Spinal stabilisation/lumbar;34=Thoracotomy;35=Tracheostomy;36=Vascular (operative);37=Vascular (endovascular treatment);38=Wound closure/graft;39=Other
SurgeryExtraCranialDelay	FALSE	FALSE	integer	The extra-cranial surgeries information was to be entered in the e-CRF in tables for which you could add as many rows as you wish. The tables consisted of - Surgery start date - Surgery start time - Surgery end date - Surgery end time - Extracranial surgery code - Reason - Delay - Short time survival if you do not operate - Short time survival if you do operate	1=Transferral from other hospital;2=Haemodynamic instability;3=No OR available;4=Surgeon delayed;5=No Delay;99=Other
SurgeryExtraCranialReason	FALSE	FALSE	integer	The extra-cranial surgeries information was to be entered in the e-CRF in tables for which you could add as many rows as you wish. The tables consisted of - Surgery start date - Surgery start time - Surgery end date - Surgery end time - Extracranial surgery code - Reason - Delay - Short time survival if you do not operate - Short time survival if you do operate	1=Emergency/Lifesaving;2=Elective;3=Treatment of complication;4=Airway management;99=Other
SurgIntervenAppro	TRUE	FALSE	integer	WHY question: How strongly does the surgeon feels that this surgical intervention is appropriate in terms of the expected benefit to final clinical outcome?	0=0;1=1;2=2;3=3;4=4;5=5;6=6;7=7;8=8;9=9;10=10
TILICPSurgery	FALSE	TRUE	integer	Records for the Daily TIL whether there was an intracranial operation for progressive mass lesion, not scheduled on admission	0=No;1=Yes
TILICPSurgeryDecomCranectomy	FALSE	TRUE	integer	Records for the Daily TIL whether there was a Decompressive Craniectomy	0=No;1=Yes
TILPhysicianConcernsCPP	FALSE	FALSE	integer	Reflects daily the physician concern and satisfaction with regard to CPP. Scales from 1 (not concerned) to 10 (very concerned)	1=1;2=2;3=3;4=4;5=5;6=6;7=7;8=8;9=9;10=10
TILPhysicianConcernsICP	FALSE	FALSE	integer	Reflects daily the physician concern and satisfaction with regard to ICP. Scales from 1 (not concerned) to 10 (very concerned)	1=1;2=2;3=3;4=4;5=5;6=6;7=7;8=8;9=9;10=10
TILPhysicianSatICP	FALSE	FALSE	integer	Documents physician satisfaction with ICP control	1=Not at all;2=Slightly;3=Moderate;4=Quite;5=Very;77=N/A (no ICP monitoring)

Supplementary Note 2. Physician-based impression variables.

Name	Static	Format	Description	Possible Values
InjAIS	TRUE	integer	In the original AIS classification of injury severity, the grading is from 1 (minor) to 6 (unsurvivable). We added a score of 0 to designate absence of injuries. This is the AIS score for body regions as specified by AIS.InjBodyRegion.	0=None;1=Minor: no treatment needed;2=Moderate: requires only outpatient treatment;3=Serious: requires non-ICU hospital admission;4=Severe: requires ICU observation and/or basic treatment;5=Critical: requires intubation, mechanical ventilation or vasopressors for blood pressure support;6=Unsurvivable: not survivable
HVTILChangeReason	FALSE	integer	Bihourly reason for change in therapy intensity level	1=Intensified: Clinical deterioration;2=Intensified: Suspicion of increased of ICP (not measured);3=Intensified: Increased ICP (documented);4=Intensified: Clinical decision to target other mechanism;5=Intensified: Change of doctor (different shift);6=Decreasing: Clinical improvement;7=Decreasing: Adequate control over ICP;8=Decreasing: Upper treatment limit reached/past;9=Decreasing: Further treatment considered futile;10=Decreasing: Change of doctor (different shift)
TILPhysicianOverallSatisfaction	FALSE	integer	This variable aims to capture the overall satisfaction of the physician with the clinical course of this patient; "not at all satisfied" would indicate that the patient did much more poorly than expected; "very satisfied" would indicate that the patient did much better than expected. Physician satisfaction should be assessed on a daily basis,	0=Not at all;1=Slightly;2=Moderately;3=Quite;4=Very
TILPhysicianOverallSatisfactionSurvival	FALSE	integer	This variable aims to capture the opinion of the treating physician as to whether the short time survival change have chnged in comparision to the previous assessment	1=Much worse;2=A little worse;3=Unchanged;4=A little better;5=Much better
TILReasonForChange	FALSE	integer	Reflects the reason for change in TIL therapy over the day.	0=No change;1=Intensified: Clinical deterioration;2=Intensified:Suspicion of increased of ICP (not measured);3=Intensified:Increased ICP (documented);4=Intensified:Clinical decision to target other mechanism;5=Intensified:Change of doctor (different shift);6=Decreasing:Clinical improvement;7=Decreasing:Adequate control over ICP;8=Decreasing:Upper treatment limit reached/past;9=Decreasing:Further treatment considered futile;10=Decreasing:Change of doctor (different shift)
TotalTIL	FALSE	integer	Calculated centrally - 24 hour TILS as the worst sum TILs for each day for the ICU timepoints (day 1-7, 10, 14, 21 and 28)	Not Applicable
AbdomenPelvicContentsAIS	TRUE	integer	AIS score for the Abdomen/Pelvic Contents In the original AIS classification of injury severity, the grading is from 1 (minor) to 6 (unsurvivable). We added a score of 0 to designate absence of injuries.	0=None;1=Minor: no treatment needed;2=Moderate: requires only outpatient treatment;3=Serious: requires non-ICU hospital admission;4=Severe: requires ICU observation and/or basic treatment;5=Critical: requires intubation, mechanical ventilation or vasopressors for blood pressure support;6=Unsurvivable: not survivable
BaselineGOS6MoExpectedDeathRisk	TRUE	text	At ER discharge, physician estimate of six month outcome was recorded as a baseline risk assessment: "Given all current available information, what is, in your subjective opinion, the most likely 6-month outcome of this patient? To be based upon information on discharge ER or admission to hospital/ICU". This reflects the Risk of death in %	Not Applicable
BaselineGOS6MoExpectedOutcome	TRUE	text	At ER discharge, physician estimate of six month outcome was recorded as a baseline risk assessment: "Given all current available information, what is, in your subjective opinion, the most likely 6-month outcome of this patient? To be based upon information on discharge ER or admission to hospital/ICU". This reflects the Expected outcome (GOS)	D=D - Death;GR=GR - Good Recovery;MD=MD - Moderate Disability;SD=SD - Severe Disability;V=V - Vegetative State
BaselineGOS6MoUnfavourableOutcomeRisk	TRUE	text	At ER discharge, physician estimate of six month outcome was recorded as a baseline risk assessment: "Given all current available information, what is, in your subjective opinion, the most likely 6-month outcome of this patient? To be based upon information on discharge ER or admission to hospital/ICU". This reflects the Risk of unfavorable outcome (D, VS, SD) in %	Not Applicable
BaselinePhysEstOf6MoOutcomePhysicianQual	TRUE	integer	At ER discharge, physician estimate of six month outcome was recorded as a baseline risk assessment: "Given all current available information, what is, in your subjective opinion, the most likely 6-month outcome of this patient? To be based upon information on discharge ER or admission to hospital/ICU". This reflects the qualification of the physician who provided prognostic estimate on ER discharge/admission to hospital/ICU	1=Resident;2=Junior staff (< 5 years);3=Senior staff (>= 5 years);4=Head of department
BaselinePhysEstOf6MoOutcomePhysicianType	TRUE	integer	At ER discharge, physician estimate of six month outcome was recorded as a baseline risk assessment: "Given all current available information, what is, in your subjective opinion, the most likely 6-month outcome of this patient? To be based upon information on discharge ER or admission to	1=ER Physician;2=Intensive Care;3=Neurology;4=Neurosurgery;5=Traumatology;88=Unknown

			hospital/ICU". This reflects the type of the physician who provided prognostic estimate on ER discharge/admission to hospital/ICU	
BestOfAbdomenPelvicLumbarISS	TRUE	integer	AbdomenPelvicLumbar region (Highest AIS of the region)^2 compare AbdomenPelvicContentsAIS, LumbarSpineAIS. This score is taken forward for ISS calculation	Not Applicable
BestOfChestSpineISS	TRUE	integer	(highest AIS of the region)^2 Compare ThoraxChestAIS, ThoracicSpineAIS and select the highest for ISS calculation	Not Applicable
BestOfExternalISS	TRUE	integer	External region (ExternaAIS)^2 select the highest external AIS severity code for ISS calculation.	Not Applicable
BestOfExtremitiesISS	TRUE	integer	Extremities region (Highest AIS of the region)^2 compare UpperExtremitiesAIS, LowerExtremitiesAIS, PelvicGirdleAIS select the highest for ISS calculation	Not Applicable
BestOfFaceISS	TRUE	integer	Face region (FaceAIS)^2 select the highest facial injury for ISS calculation	Not Applicable
BestOfHeadBrainCervicalISS	TRUE	integer	HeadBrainCervical region (Highest AIS of the region)^2 Compare HeadNeckAIS, InjuryHx.BrainInjuryAIS, CervicalSpineAIS select the highest scoring injury in any of these 3 areas for ISS calculation	Not Applicable
BrainInjuryAIS	TRUE	integer	AIS score for the Brain Injury In the original AIS classification of injury severity, the grading is from 1 (minor) to 6 (unsurvivable). We added a score of 0 to designate absence of injuries.	0=None;1=Minor: no treatment needed;2=Moderate: requires only outpatient treatment;3=Serious: requires non-ICU hospital admission;4=Severe: requires ICU observation and/or basic treatment;5=Critical: requires intubation, mechanical ventilation or vasopressors for blood pressure support;6=Unsurvivable: not survivable
CervicalSpineAIS	TRUE	integer	AIS score for the Cervical Spine region. In the original AIS classification of injury severity, the grading is from 1 (minor) to 6 (unsurvivable). We added a score of 0 to designate absence of injuries.	0=None;1=Minor: no treatment needed;2=Moderate: requires only outpatient treatment;3=Serious: requires non-ICU hospital admission;4=Severe: requires ICU observation and/or basic treatment;5=Critical: requires intubation, mechanical ventilation or vasopressors for blood pressure support;6=Unsurvivable: not survivable
DispER	TRUE	integer	Destination of the patient at ER discharge.	1=Discharge home;2=Discharge other facility;3=Hospital admission--Ward;4=Hospital admission--Intermediate/high care unit;5=Hospital admission--ICU;6=Hospital admission--OR for immediate surgical procedure;7=Death;8=Hospital admission--Other (e.g. observation unit);88=Unknown
EmerSurgIntraCranSurviveNoSurg	TRUE	integer	"InjuryHx.EmerSurgIntraCranSurviveNoSurg" and "InjuryHx.EmerSurgIntraCranSurviveYesSurg" These 2 variables aim to capture information on the surgeon's expectations, eg if the surgeon considers a realistic expectation of benefit, or performs the surgery as a "last resort" in a likely hopeless case. 'The short term survival chances of the patients if I DO NOT operate will be (in %)'	Not Applicable
EmerSurgIntraCranSurviveYesSurg	TRUE	integer	"InjuryHx.EmerSurgIntraCranSurviveNoSurg" and "InjuryHx.EmerSurgIntraCranSurviveYesSurg" These 2 variables aim to capture information on the surgeon's expectations, eg if the surgeon considers a realistic expectation of benefit, or performs the surgery as a "last resort" in a likely hopeless case. 'The short term survival chances of the patients if I DO operate will be (in %)'	Not Applicable
ExternaAIS	TRUE	integer	AIS score for the External skin In the original AIS classification of injury severity, the grading is from 1 (minor) to 6 (unsurvivable). We added a score of 0 to designate absence of injuries.	0=None;1=Minor: no treatment needed;2=Moderate: requires only outpatient treatment;3=Serious: requires non-ICU hospital admission;4=Severe: requires ICU observation and/or basic treatment;5=Critical: requires intubation, mechanical ventilation or vasopressors for blood pressure support;6=Unsurvivable: not survivable
FaceAIS	TRUE	integer	AIS score for Face (incl.maxillofacial) In the original AIS classification of injury severity, the grading is from 1 (minor) to 6 (unsurvivable). We added a score of 0 to designate absence of injuries.	0=None;1=Minor: no treatment needed;2=Moderate: requires only outpatient treatment;3=Serious: requires non-ICU hospital admission;4=Severe: requires ICU observation and/or basic treatment;5=Critical: requires intubation, mechanical ventilation or vasopressors for blood pressure support;6=Unsurvivable: not survivable
HeadNeckAIS	TRUE	integer	AIS score for the Head Neck region In the original AIS classification of injury severity, the grading is from 1 (minor) to 6 (unsurvivable). We added a score of 0 to designate absence of injuries.	0=None;1=Minor: no treatment needed;2=Moderate: requires only outpatient treatment;3=Serious: requires non-ICU hospital admission;4=Severe: requires ICU observation and/or basic treatment;5=Critical: requires intubation, mechanical ventilation or vasopressors for blood pressure support;6=Unsurvivable: not survivable
LowerExtremitiesAIS	TRUE	integer	AIS score for the Lower extremities. In the original AIS classification of injury severity, the grading is from 1 (minor) to 6 (unsurvivable). We added a score of 0 to designate absence of injuries.	0=None;1=Minor: no treatment needed;2=Moderate: requires only outpatient treatment;3=Serious: requires non-ICU hospital admission;4=Severe: requires ICU observation and/or basic treatment;5=Critical: requires intubation, mechanical ventilation or vasopressors for blood pressure support;6=Unsurvivable: not survivable
LumbarSpineAIS	TRUE	integer	AIS score for the Lumbar Spine region. In the original AIS classification of injury severity, the grading is from 1 (minor)	0=None;1=Minor: no treatment needed;2=Moderate: requires only outpatient treatment;3=Serious: requires non-ICU

			to 6 (unsurvivable). We added a score of 0 to designate absence of injuries.	hospital admission;4=Severe: requires ICU observation and/or basic treatment;5=Critical: requires intubation, mechanical ventilation or vasopressors for blood pressure support;6=Unsurvivable: not survivable
PelvicGirdleAIS	TRUE	integer	AIS score for the Pelvic Girdle region. In the original AIS classification of injury severity, the grading is from 1 (minor) to 6 (unsurvivable). We added a score of 0 to designate absence of injuries.	0=None;1=Minor: no treatment needed;2=Moderate: requires only outpatient treatment;3=Serious: requires non-ICU hospital admission;4=Severe: requires ICU observation and/or basic treatment;5=Critical: requires intubation, mechanical ventilation or vasopressors for blood pressure support;6=Unsurvivable: not survivable
SurgIntervenAppro	TRUE	integer	WHY question: How strongly does the surgeon feels that this surgical intervention is appropriate in terms of the expected benefit to final clinical outcome?	0=0;1=1;2=2;3=3;4=4;5=5;6=6;7=7;8=8;9=9;10=10
ThoracicSpineAIS	TRUE	integer	AIS score for the Thoracic spine Region In the original AIS classification of injury severity, the grading is from 1 (minor) to 6 (unsurvivable). We added a score of 0 to designate absence of injuries.	0=None;1=Minor: no treatment needed;2=Moderate: requires only outpatient treatment;3=Serious: requires non-ICU hospital admission;4=Severe: requires ICU observation and/or basic treatment;5=Critical: requires intubation, mechanical ventilation or vasopressors for blood pressure support;6=Unsurvivable: not survivable
ThoraxChestAIS	TRUE	integer	AIS score for the Thorax Chest region. In the original AIS classification of injury severity, the grading is from 1 (minor) to 6 (unsurvivable). We added a score of 0 to designate absence of injuries.	0=None;1=Minor: no treatment needed;2=Moderate: requires only outpatient treatment;3=Serious: requires non-ICU hospital admission;4=Severe: requires ICU observation and/or basic treatment;5=Critical: requires intubation, mechanical ventilation or vasopressors for blood pressure support;6=Unsurvivable: not survivable
TotalISS	TRUE	integer	The Injury Severity Score is calculated as the sum of the squares of the the 3 body regions with the highest AIS score. The max score for the ISS = 75. If any body region AIS is assigned a score of "6", the ISS is automatically set to 75 (highest score). In the calculation of the ISS, only the 6 main body regions are taken into consideration.	Not Applicable
UpperExtremitiesAIS	TRUE	integer	AIS score for the Upper extremities. In the original AIS classification of injury severity, the grading is from 1 (minor) to 6 (unsurvivable). We added a score of 0 to designate absence of injuries.	0=None;1=Minor: no treatment needed;2=Moderate: requires only outpatient treatment;3=Serious: requires non-ICU hospital admission;4=Severe: requires ICU observation and/or basic treatment;5=Critical: requires intubation, mechanical ventilation or vasopressors for blood pressure support;6=Unsurvivable: not survivable
CRFCTReason	FALSE	text	This variable contains the main reason why a CT-scan, during hospital stay, was performed. One of following options: standard follow-up, post-operative control, clinical deterioration, (suspicion of) increasing ICP, lack of improvement, unknown, other (specified in CTMRI.CTReasonOther) The reason for making an early CT-scan/ER scan can be found in: CTMRI.CTERReason	CD=Clinical deterioration;ICUADM88=Unknown;ICUADM99=Other;IICP=(Suspicion of) Increasing ICP;LOP=Lack of improvement;POC=Post-operative control;SFU=Standard follow-up
CTNoOpMotiv	FALSE	integer	WHY question: documents reason for not having an indication for (intra)cranial surgery.	0=No surgical lesion;1=Lesion present, but Acceptable/good neurologic condition;2=Lesion present, but Guideline adherence;3=Lesion present, but Little/no mass effect;4=Lesion present, but Not hospital policy;5=Lesion present, but Extremely poor prognosis;6=Lesion present, but Brain Death;7=Lesion present, but Old age;8=Lesion present, but Wish family;88=Unknown;99=Lesion present, but Other
CTNoOpMotivOther	FALSE	text	Specification, only applicable if "CTMRI.CTNoOpMotiv" was "other"	Not Applicable
CTYesOpMotiv	FALSE	integer	WHY question: documents reason for having an indication for (intra)cranial surgery.	1=Emergency/life saving;2=Clinical deterioration;3=Mass effect on CT;4=Radiological progression;5=(Suspicion of) raised ICP;6=Guideline adherence;7=To prevent deterioration;8=Depressed skull fracture;99=Other
CTYesOpMotivOther	FALSE	text	Free text if "CTMRI.CTYesOpMotiv" was marked as 'Other'. Relates to the WHY question: documents reason for having an indication for (intra)cranial surgery.	Not Applicable
ERCTNoOpMotiv	TRUE	integer	In emergency room: WHY question: documents reason for not having an indication for (intra)cranial surgery.	0=No surgical lesion;1=Lesion present, but Acceptable/good neurologic condition;2=Lesion present, but Guideline adherence;3=Lesion present, but Little/no mass effect;4=Lesion present, but Not hospital policy;5=Lesion present, but Extremely poor prognosis;6=Lesion present, but Brain Death;7=Lesion present, but Old age;8=Lesion present, but Wish family;88=Unknown;99=Lesion present, but Other
ERCTNoOpMotivOther	TRUE	text	In emergency room: Specification, only applicable if "CTMRI.CTNoOpMotiv" was "other"	Not Applicable
ERCTYesOpMotiv	TRUE	integer	In emergency room: WHY question: documents reason for having an indication for (intra)cranial surgery.	1=Emergency/life saving;2=Clinical deterioration;3=Mass effect on CT;4=Radiological progression;5=(Suspicion of) raised ICP;6=Guideline adherence;7=To prevent deterioration;8=Depressed skull fracture;99=Other
ERCTYesOpMotivOther	TRUE	text	In emergency room: Free text if "CTMRI.CTYesOpMotiv" was marked as 'Other'. Relates to the WHY question:	Not Applicable

		documents reason for having an indication for (intra)cranial surgery.	
ShortTermSurvivalNoSurg	FALSE integer	Aims to capture information on the surgeon's expectations, eg if the surgeon considers a realistic expectation of benefit, or performs the surgery as a "last resort" in a likely hopeless case --> The short term survival chances of the patient if I DO NOT operate (1-100)	Not Applicable
ShortTermSurvivalYesSurg	FALSE integer	Aims to capture information on the surgeon's expectations, eg if the surgeon considers a realistic expectation of benefit, or performs the surgery as a "last resort" in a likely hopeless case --> The short term survival chances of the patient if I DO operate (1-100)	Not Applicable
SurgeryCranialReason	FALSE integer	WHY Question: aims to document the reason for intracranial surgery	1=Emergency/Life saving;2=Clinical deterioration;3=Mass effect on CT;4=Radiological progression;5=suspicion of) raised ICP;6=Guideline adherence;7=To prevent deterioration
SurgeryExtraCranialReason	FALSE integer	The extra-cranial surgeries information was to be entered in the e-CRF in tables for which you could add as many rows as you wish. The tables consisted of - Surgery start date - Surgery start time - Surgery end date - Surgery end time - Extracranial surgery code - Reason - Delay - Short time survival if you do not operate - Short time survival if you do operate	1=Emergency/Lifesaving;2=Elective;3=Treatment of complication;4=Airway management;99=Other
TransReason	FALSE integer	WHY question: documents reason for transition of care	1=Mechanical ventilation;2=Frequent neurological observations;3=Haemodynamic invasive monitoring;4=Extracranial injuries;5=Neurological operation;6=Clinical deterioration;7=CT abnormalities;8=Clinical observation for TBI;9=No ICU bed available;10=Could be discharged home, but no adequate supervision;11=Improvement;12=Neurological deterioration;13=Systemic compilation;14=CT progression;15=Planned surgery;16=Condition stable;17=(acute) Treatment goals accomplished;18=Need to free a bed;19=Further improvement;20=Clinical rehab completed;21=Lack of improvement;22=Late neurological deterioration;23=Problems unrelated to trauma;24=Post operative care;25=Neurological complication;99=Other

Supplementary Note 3. Manually excluded variables indicating death or withdrawal of life-sustaining treatment.

Name	Format	Description	Possible Values
BrainDeathDate	date	Reflects the date of Brain death in case of Withdrawal of life-sustaining measures	Not Applicable
BrainDeathTime	text	Reflects the Time of Brain death in case of Withdrawal of life-sustaining measures Check also "Hospital.BrainDeathDate" for the date.	Not Applicable
CTPatientLocation	text	This variable describes the in-hospital location of the patient when the CT-scan was performed and was not meant to describe the location of the CT-scanner. Three options: ER, Ward/Admission, ICU	ADMIS=Ward/Admission;ED=ER;ICU=ICU
DeadAge	integer	Reflects if the reason for Withdrawal of life-sustaining measures was age.	0=No;1=Yes;88=Unknown
DeadCoMorbidities	integer	Reflects if the reason for Withdrawal of life-sustaining measures was co-morbidities.	0=No;1=Yes;88=Unknown
DeadDeterminationOfBrainDeath	integer	Reflects if the reason for Withdrawal of life-sustaining measures was Determination of brain death (according to national law).	0=No;1=Yes;88=Unknown
DeadOrganDonation	integer	Reflects if Withdrawal of life-sustaining measures was followed by organ donation.	0=No;1=Yes;88=Unknown
DeadPatWill	integer	Reflects if the reason for Withdrawal of life-sustaining measures was Following living will of patient.	0=No;1=Yes;88=Unknown
DeadRequestRelatives	integer	Reflects if the reason for Withdrawal of life-sustaining measures was On request of relatives.	0=No;1=Yes;88=Unknown
DeadSeverityofTBI	integer	Reflects if the reason for Withdrawal of life-sustaining measures was Severity of TBI.	0=No;1=Yes;88=Unknown
DeathAutopsy	integer	Reflects if an autopsy was performed after the death of the patient.	0=No;1=Yes, forensic;2=Yes, clinical;88=Unknown
DeathCause	integer	Cause of death in or outside the hospital	1=Head injury/initial injury;2=Head injury/secondary intracranial damage;3=Systemic trauma;4=Medical complications;88=Unknown;99=Other
DeathCauseOther	text	"Other" cause of death in or outside the hospital (than predefined list).	Not Applicable
DeathDate	date	Date of death also recorded on hospital discharge and at followup: FollowUp.FUPrincipalDeathCause;Death may also have been recorded in the ER forms: Subject.DeathDate	Not Applicable
DeathERDeclaredBrainDeadFollowingNationalCriteria	integer	Reflects if patient was declared brain dead following national criteria. Only applicable if patient declared "dead" on the ER	0=No;1=Yes;88=Unknown
DeathERDOA	integer	Reflects if patient is declared dead on the ER --> Dead on arrival (DOA). Only applicable if patient declared "dead" on the ER.	0=No;1=Yes;88=Unknown
DeathERUnsuccResusForExtraCranInj	integer	Reflects unsuccessful resuscitation for extra cranial injuries if patient is declared dead on the ER. Only applicable if patient declared "dead" on the ER	0=No;1=Yes;88=Unknown
DeathERWithdrawalLifeSuppForSeverityOfTBI	integer	Reflects Withdrawal of life¬≠sustaining measures for severity of TBI if patient is declared dead on the ER. Only applicable if patient declared "dead" on the ER	0=No;1=Yes;88=Unknown
DeathTime	text	Time of death	Not Applicable
DischargeStatus	integer	Assessment by investigator Reflects if the patient was dead or alive at discharge.	0=Dead;1=Alive;88=Unknown
EOSReason	integer	Reason for end of study participation	1=Completion of study;2=Inability to obtain follow-up;3=Withdrawal from study (by patient or representative);4=Adverse event(s);5=Decision for DNR*:;6=Withdrawal of support;7=Death;99=Other
EOSReasonOtherTxt	text	"Other" reason for end of study participation than the predefined list.	Not Applicable
ICDCode1	text	Up to 16 fields available to enter diagnosis as recorded by hospital administration according to ICD codes; applicable to patients admitted/discharged from hospital. For patients discharged directly from the ER, ICD codes are documented in: InjuryHx.ERDestICDCodes1	Not Applicable
ICDCode2	text	Up to 16 fields available to enter diagnosis as recorded by hospital administration according to ICD codes; applicable to patients admitted/discharged from hospital. For patients discharged directly from the ER, ICD codes are documented in: InjuryHx.ERDestICDCodes1	Not Applicable
ICDCode3	text	Up to 16 fields available to enter diagnosis as recorded by hospital administration according to ICD codes; applicable to patients admitted/discharged from hospital. For patients discharged directly from the ER, ICD codes are documented in: InjuryHx.ERDestICDCodes1	Not Applicable
ICDCode4	text	Up to 16 fields available to enter diagnosis as recorded by hospital administration according to ICD codes; applicable to patients admitted/discharged from hospital.	Not Applicable

Name	Format	Description	Possible Values
		For patients discharged directly from the ER, ICD codes are docu mented in: InjuryHx.ERDestICDCodes1	
ICDCode5	text	Up to 16 fields available to enter diagnosis as recorded by hospital administration according to ICD codes; applicable to patients admi tted/discharged from hospital.<br>For patients discharged directly from the ER, ICD codes are docu mented in: InjuryHx.ERDestICDCodes1	Not Applicable
ICDCode6	text	Up to 16 fields available to enter diagnosis as recorded by hospital administration according to ICD codes; applicable to patients admi tted/discharged from hospital.<br>For patients discharged directly from the ER, ICD codes are docu mented in: InjuryHx.ERDestICDCodes1	Not Applicable
ICDCodeVersion	integer	This variable reflects if the ICD code version 9 or version 10 was u sed.<br>Up to 16 fields are available to enter diagnosis as recorded by hosp ital administration according to ICD codes.	9=ICD-9;10=ICD-10
ICPStopReason	integer	Reason for stopping ICP. Also check Hospital.ICPMonitorStop.	1=Clinically improved;2=ICP stable and < 20 mmHg;3= Monitor/catheter failure;4=Patient considered unsalvaga ble;5=Patient died;99=Other
ICPStopReason	integer	Reason for stopping ICP. Also check Hospital.ICPMonitorStop.	1=Clinically improved;2=ICP stable and < 20 mmHg;3= Monitor/catheter failure;4=Patient considered unsalvaga ble;5=Patient died;99=Other
ICUDischargeICDCode1	text	The intent here is to register ICD code as recorded in hospital admi nistrative files for patients directly discharged from the ICU. Up to 16 codes can be entered, ICD codes are further captured at ER disc harge and at hospital discharge.	Not Applicable
ICUDischargeICDCode2	text	The intent here is to register ICD code as recorded in hospital admi nistrative files for patients directly discharged from the ICU. Up to 16 codes can be entered, ICD codes are further captured at ER disc harge and at hospital discharge.	Not Applicable
ICUDischargeICDCode3	text	The intent here is to register ICD code as recorded in hospital admi nistrative files for patients directly discharged from the ICU. Up to 16 codes can be entered, ICD codes are further captured at ER disc harge and at hospital discharge.	Not Applicable
ICUDischargeICDCode4	text	The intent here is to register ICD code as recorded in hospital admi nistrative files for patients directly discharged from the ICU. Up to 16 codes can be entered, ICD codes are further captured at ER disc harge and at hospital discharge.	Not Applicable
ICUDischargeICDCode5	text	The intent here is to register ICD code as recorded in hospital admi nistrative files for patients directly discharged from the ICU. Up to 16 codes can be entered, ICD codes are further captured at ER disc harge and at hospital discharge.	Not Applicable
ICUDischargeICDCode6	text	The intent here is to register ICD code as recorded in hospital admi nistrative files for patients directly discharged from the ICU. Up to 16 codes can be entered, ICD codes are further captured at ER disc harge and at hospital discharge.	Not Applicable
ICUDischargeICDCodeVersion	integer	This variable reflects if the ICD code version 9 or version 10 was u sed.<br>Up to 16 fields are available to enter diagnosis as recorded by hosp ital administration according to ICD codes.	9=9;10=10
ICUDischargeStatus	integer	Reflects if patient was alive or dead on discharge from ICU	1=Alive;2=Dead;88=Unknown
ICUDischargeTo	integer	Reflects location to which the patient was discharged from ICU	1=General ward;2=Other ICU;3=Other hospital;4=Rehab unit;5=Home;6=Nursing home;7=Step down/high care u nit;88=Unknown;99=Other
ICUDischargeToOther	text	Specifies the "other" location to which the patient was discharged f rom ICU Check also "Hospital.ICUDischargeTo"	Not Applicable
ICUDisPatDeadAtICU	integer	Reflects if the patient was declared dead on the ICU.<br>Intended as an introductory question for the details on withdrawal of treatment, brain death and organ donation	0=No;1=Yes
ICUDisSupportWithdrawnDate	date	This variable documents date and time at which life prolonging the rapy was withdrawn<br>(together with "Hospital.ICUDisSupportWithdrawnTime")	Not Applicable
ICUDisSupportWithdrawnTime	text	This variable documents date and time at which life prolonging the rapy was withdrawn<br>(together with "Hospital.ICUDisSupportWithdrawnDate")	Not Applicable
ICUDisWithdrawalTreatmentDe cisionDate	date	Investigators were requested to record the details of Withdrawal of Treatment or Life support if applicable.	Not Applicable
ICUDisWithdrawalTreatmentDe cisionTime	text	Investigators were requested to record the details of Withdrawal of Treatment or Life support if applicable.	Not Applicable
ICUDisWithdrawlTreatmentDeci sion	integer	Investigators were requested to record the details of Withdrawal of Treatment or Life support if applicable.	1=Multi disciplinary;2=By a single physician;3=With rel atives
IntubationStopReason	integer	This variable describes the Stop Reason of Extubation in case of Ventilation Management (only for ICU patients).	1=Respiratory stable;2=Accidental;3=Withdrawal of care

Name	Format	Description	Possible Values
LengthOfStay	decimal	This variable reflects the length of stay of the patient at the study hospital. It has been derived using the information of the date and time of arrival at the study hospital and date and time of (study) hospital discharge.	Not Applicable
MonJugularSatStopReason	integer	Beside brain specific ICP monitoring in the ICU, details were recorded on other types of monitoring in the ICU. This variable reflects the reason for stopping if there was Jugular oximetry.	1=Monitor/catheter failure;2=Patient considered unsalvageable;3=Patient died;4=Clinically no longer required
MonLicoxStopReason	integer	Beside brain specific ICP monitoring in the ICU, details were recorded on other types of monitoring in the ICU. This variable reflects the reason for stopping if there was Brain tissue PO2 monitoring.	1=Monitor/catheter failure;2=Patient considered unsalvageable;3=Patient died;4=Clinically no longer required
MonLicoxStopReason	integer	Beside brain specific ICP monitoring in the ICU, details were recorded on other types of monitoring in the ICU. This variable reflects the reason for stopping if there was Brain tissue PO2 monitoring.	1=Monitor/catheter failure;2=Patient considered unsalvageable;3=Patient died;4=Clinically no longer required
MonMicrodialysisStopReason	integer	Beside brain specific ICP monitoring in the ICU, details were recorded on other types of monitoring in the ICU. This variable reflects the reason for stopping if there was Microdialysis.	1=Monitor/catheter failure;2=Patient considered unsalvageable;3=Patient died;4=Clinically no longer required
OrganDonationDate	date	Reflects the date of organ donation in case of Withdrawal of life-sustaining measures, if applicable.	Not Applicable
OrganDonationTime	text	Reflects the time of organ donation in case of Withdrawal of life-sustaining measures, if applicable.	Not Applicable
SupportWithdrawnDate	date	Investigators were requested to record the details of Withdrawal of Treatment or Life support if applicable.	Not Applicable
SupportWithdrawnTime	text	Investigators were requested to record the details of Withdrawal of Treatment or Life support if applicable.	Not Applicable
TimeSinceICUAdmisDeath	text	Investigators were requested to record the details of Withdrawal of Treatment or Life support if applicable. This reflects the time between admission in the ICU and death	Not Applicable
WithdrawalOption	integer	In case of complete withdrawal, all data have been deleted from the database	1=Complete Withdrawal (no further contact, destruction of all data and samples collected up to that point);2=No further study related activities, but consent to access of clinical notes and use of existing data
WithdrawalTreatmentDecision	integer	Intended only to be scored if a medical decision was made to withdraw active treatment because of anticipated poor prognosis. However, some investigators may have scored this when patients had recovered to an extent that active treatment was no longer necessary.	1=Multi disciplinary;2=By a single physician;3=With relatives
WithdrawalTreatmentDecisionDate	date	Investigators were requested to record the details of Withdrawal of Treatment or Life support if applicable. Intended only to be scored if a medical decision was made to withdraw active treatment because of anticipated poor prognosis. However, some investigators may have scored this when patients had recovered to an extent that active treatment was no longer necessary.	Not Applicable
WithdrawalTreatmentDecisionTime	text	Investigators were requested to record the details of Withdrawal of Treatment or Life support if applicable. Intended only to be scored if a medical decision was made to withdraw active treatment because of anticipated poor prognosis. However, some investigators may have scored this when patients had recovered to an extent that active treatment was no longer necessary.	Not Applicable
WithdrawSuppDateTime	datetime	Withdrawal of Support Date & Time if Withdrawal of life-sustaining support was the reason for end of study participation.	Not Applicable

Supplementary Note 4. The CENTER-TBI investigators and participants.

The co-lead investigators of CENTER-TBI are designated with an asterisk (*), and their contact email addresses are listed below.

Cecilia Åkerlund[1], Krisztina Amrein[2], Nada Andelic[3], Lasse Andreassen[4], Audny Anke[5], Anna Antoni[6], Gérard Audibert[7], Philippe Azouvi[8], Maria Luisa Azzolini[9], Ronald Bartels[10], Pál Barzó[11], Romuald Beauvais[12], Ronny Beer[13], Bo-Michael Bellander[14], Antonio Belli[15], Habib Benali[16], Maurizio Berardino[17], Luigi Beretta[9], Morten Blaabjerg[18], Peter Bragge[19], Alexandra Brazinova[20], Vibeke Brinck[21], Joanne Brooker[22], Camilla Brorsson[23], Andras Buki[24], Monika Bullinger[25], Manuel Cabeleira[26], Alessio Caccioppola[27], Emiliana Calappi[27], Maria Rosa Calvi[9], Peter Cameron[28], Guillermo Carbayo Lozano[29], Marco Carbonara[27], Simona Cavallo[17], Giorgio Chevallard[30], Arturo Chieregato[30], Giuseppe Citerio[31,32], Hans Clusmann[33], Mark Coburn[34], Jonathan Coles[35], Jamie D. Cooper[36], Marta Correia[37], Amra Čović [38], Nicola Curry[39], Endre Czeiter[24], Marek Czosnyka[26], Claire Dahyot-Fizelier[40], Paul Dark[41], Helen Dawes[42], Véronique De Keyser[43], Vincent Degos[16], Francesco Della Corte[44], Hugo den Boogert[10], Bart Depreitere[45], Đula Đilvesi[46], Abhishek Dixit[47], Emma Donoghue[22], Jens Dreier[48], Guy-Loup Dulière[49], Ari Ercole[47], Patrick Esser[42], Erzsébet Ezer[50], Martin Fabricius[51], Valery L. Feigin[52], Kelly Foks[53], Shirin Frisvold[54], Alex Furmanov[55], Pablo Gagliardo[56], Damien Galanaud[16], Dashiell Gantner[28], Guoyi Gao[57], Pradeep George[58], Alexandre Ghuysen[59], Lelde Giga[60], Ben Glocker[61], Jagoš Golubovic[46], Pedro A. Gomez[62], Johannes Gratz[63], Benjamin Gravesteijn[64], Francesca Grossi[44], Russell L. Gruen[65], Deepak Gupta[66], Juanita A. Haagsma[64], Iain Haitsma[67], Raimund Helbok[13], Eirik Helseth[68], Lindsay Horton[69], Jilske Huijben[64], Peter J. Hutchinson[70], Bram Jacobs[71], Stefan Jankowski[72], Mike Jarrett[21], Ji-yao Jiang[58], Faye Johnson[73], Kelly Jones[52], Mladen Karan[46], Angelos G. Kolias[70], Erwin Kompanje[74], Daniel Kondziella[51], Evgenios Kornaropoulos[47], Lars-Owe Koskinen[75], Noémi Kovács[76], Ana Kowark[77], Alfonso Lagares[62], Linda Lanyon[58], Steven Laureys[78], Fiona Lecky[79,80], Didier Ledoux[78], Rolf Lefering[81], Valerie Legrand[82], Aurelie Lejeune[83], Leon Levi[84], Roger Lightfoot[85], Hester Lingsma[64], Andrew I.R. Maas[43,*], Ana M. Castaño-León[62], Marc Maegele[86], Marek Majdan[20], Alex Manara[87], Geoffrey Manley[88], Costanza Martino[89], Hugues Maréchal[49], Julia Mattern[90], Catherine McMahon[91], Béla Melegh[92], David Menon[47,*], Tomas Menovsky[43], Ana Mikolic[64], Benoit Misset[78], Visakh Muraleedharan[58], Lynnette Murray[28], Ancuta Negru[93], David Nelson[1], Virginia Newcombe[47], Daan Nieboer[64], József Nyirádi[2], Otesile Olubukola[79], Matej Oresic[94], Fabrizio Ortolano[27], Aarno Palotie[95,96,97], Paul M. Parizel[98], Jean-François Payen[99], Natascha Perera[12], Vincent Perlbarg[16], Paolo Persona[100], Wilco Peul[101], Anna Piippo-Karjalainen[102], Matti Pirinen[95], Dana Pisica[64], Horia Ples[93], Suzanne Polinder[64], Inigo Pomposo[29], Jussi P. Posti[103], Louis Puybasset[104], Andreea Radoi[105], Arminas Ragauskas[106], Rahul Raj[102], Malinka Rambadagalla[107], Isabel Retel Helmrich[64], Jonathan Rhodes[108], Sylvia Richardson[109], Sophie Richter[47], Samuli Ripatti[95], Saulius Rocka[106], Cecilie Roe[110], Olav Roise[111,112], Jonathan Rosand[113], Jeffrey V. Rosenfeld[114], Christina Rosenlund[115], Guy Rosenthal[55], Rolf Rossaint[77], Sandra Rossi[100], Daniel Rueckert[61] Martin Rusnák[116], Juan Sahuquillo[105], Oliver Sakowitz[90,117], Renan Sanchez-Porras[117], Janos Sandor[118], Nadine Schäfer[81], Silke Schmidt[119], Herbert Schoechl[120], Guus Schoonman[121], Rico Frederik Schou[122], Elisabeth Schwendenwein[6], Charlie Sewalt[64], Ranjit D. Singh[101], Toril Skandsen[123,124,] Peter Smielewski[26], Abayomi Sorinola[125], Emmanuel Stamatakis[47], Simon Stanworth[39], Robert Stevens[126], William Stewart[127], Ewout W. Steyerberg[64,128], Nino Stocchetti[129], Nina Sundström[130], Riikka Takala[131], Viktória Tamás[125], Tomas Tamosuitis[132], Mark Steven Taylor[20], Braden Te Ao[52], Olli Tenovuo[103], Alice Theadom[52], Matt Thomas[87], Dick Tibboel[133], Marjolein Timmers[74], Christos Tolias[134], Tony Trapani[28], Cristina Maria Tudora[93], Andreas Unterberg[90], Peter Vajkoczy [135], Shirley Vallance[28], Egils Valeinis[60], Zoltán Vámos[50], Mathieu van der Jagt[136], Gregory Van der Steen[43], Joukje van der Naalt[71], Jeroen T.J.M. van Dijck[101], Inge A. M. van Erp[101], Thomas A. van Essen[101], Wim Van Hecke[137], Caroline van Heugten[138], Dominique Van Praag[139], Ernest van Veen[64], Thijs Vande Vyvere[137], Roel P. J. van Wijk[101], Alessia Vargiolu[32], Emmanuel Vega[83], Kimberley Velt[64], Jan Verheyden[137], Paul M. Vespa[140], Anne Vik[123,141], Rimantas Vilcinis[132], Victor Volovici[67], Nicole von Steinbüchel[38], Daphne Voormolen[64], Petar Vulekovic[46], Kevin K.W. Wang[142], Daniel Whitehouse[47], Eveline Wiegers[64], Guy Williams[47], Lindsay Wilson[69], Stefan Winzeck[47], Stefan Wolf[143], Zhihui Yang[113], Peter Ylén[144], Alexander Younsi[90], Frederick A. Zeiler[47,145], Veronika Zelinkova[20], Agate Ziverte[60,] Tommaso Zoerle[27]

[1]Department of Physiology and Pharmacology, Section of Perioperative Medicine and Intensive Care, Karolinska Institutet, Stockholm, Sweden
[2]János Szentágothai Research Centre, University of Pécs, Pécs, Hungary
[3]Division of Surgery and Clinical Neuroscience, Department of Physical Medicine and Rehabilitation, Oslo University Hospital and University of Oslo, Oslo, Norway
[4]Department of Neurosurgery, University Hospital Northern Norway, Tromso, Norway
[5]Department of Physical Medicine and Rehabilitation, University Hospital Northern Norway, Tromso, Norway
[6]Trauma Surgery, Medical University Vienna, Vienna, Austria
[7]Department of Anesthesiology & Intensive Care, University Hospital Nancy, Nancy, France
[8]Raymond Poincare hospital, Assistance Publique – Hopitaux de Paris, Paris, France
[9]Department of Anesthesiology & Intensive Care, S Raffaele University Hospital, Milan, Italy
[10]Department of Neurosurgery, Radboud University Medical Center, Nijmegen, The Netherlands
[11]Department of Neurosurgery, University of Szeged, Szeged, Hungary
[12]International Projects Management, ARTTIC, Munchen, Germany
[13]Department of Neurology, Neurological Intensive Care Unit, Medical University of Innsbruck, Innsbruck, Austria
[14]Department of Neurosurgery & Anesthesia & intensive care medicine, Karolinska University Hospital, Stockholm, Sweden
[15]NIHR Surgical Reconstruction and Microbiology Research Centre, Birmingham, UK
[16]Anesthesie-Réanimation, Assistance Publique – Hopitaux de Paris, Paris, France
[17]Department of Anesthesia & ICU, AOU Città della Salute e della Scienza di Torino - Orthopedic and Trauma Center, Torino, Italy

[18]Department of Neurology, Odense University Hospital, Odense, Denmark
[19]BehaviourWorks Australia, Monash Sustainability Institute, Monash University, Victoria, Australia
[20]Department of Public Health, Faculty of Health Sciences and Social Work, Trnava University, Trnava, Slovakia
[21]Quesgen Systems Inc., Burlingame, California, USA
[22]Australian & New Zealand Intensive Care Research Centre, Department of Epidemiology and Preventive Medicine, School of Public Health and Preventive Medicine, Monash University, Melbourne, Australia
[23]Department of Surgery and Perioperative Science, Umeå University, Umeå, Sweden
[24]Department of Neurosurgery, Medical School, University of Pécs, Hungary and Neurotrauma Research Group, János Szentágothai Research Centre, University of Pécs, Hungary
[25]Department of Medical Psychology, Universitätsklinikum Hamburg-Eppendorf, Hamburg, Germany
[26]Brain Physics Lab, Division of Neurosurgery, Dept of Clinical Neurosciences, University of Cambridge, Addenbrooke's Hospital, Cambridge, UK
[27]Neuro ICU, Fondazione IRCCS Cà Granda Ospedale Maggiore Policlinico, Milan, Italy
[28]ANZIC Research Centre, Monash University, Department of Epidemiology and Preventive Medicine, Melbourne, Victoria, Australia
[29]Department of Neurosurgery, Hospital of Cruces, Bilbao, Spain
[30]NeuroIntensive Care, Niguarda Hospital, Milan, Italy
[31]School of Medicine and Surgery, Università Milano Bicocca, Milano, Italy
[32]NeuroIntensive Care, ASST di Monza, Monza, Italy
[33]Department of Neurosurgery, Medical Faculty RWTH Aachen University, Aachen, Germany
[34]Department of Anesthesiology and Intensive Care Medicine, University Hospital Bonn, Bonn, Germany
[35]Department of Anesthesia & Neurointensive Care, Cambridge University Hospital NHS Foundation Trust, Cambridge, UK
[36]School of Public Health & PM, Monash University and The Alfred Hospital, Melbourne, Victoria, Australia
[37]Radiology/MRI department, MRC Cognition and Brain Sciences Unit, Cambridge, UK
[38]Institute of Medical Psychology and Medical Sociology, Universitätsmedizin Göttingen, Göttingen, Germany
[39]Oxford University Hospitals NHS Trust, Oxford, UK
[40]Intensive Care Unit, CHU Poitiers, Potiers, France
[41]University of Manchester NIHR Biomedical Research Centre, Critical Care Directorate, Salford Royal Hospital NHS Foundation Trust, Salford, UK
[42]Movement Science Group, Faculty of Health and Life Sciences, Oxford Brookes University, Oxford, UK
[43]Department of Neurosurgery, Antwerp University Hospital and University of Antwerp, Edegem, Belgium
[44]Department of Anesthesia & Intensive Care, Maggiore Della Carità Hospital, Novara, Italy
[45]Department of Neurosurgery, University Hospitals Leuven, Leuven, Belgium
[46]Department of Neurosurgery, Clinical centre of Vojvodina, Faculty of Medicine, University of Novi Sad, Novi Sad, Serbia
[47]Division of Anaesthesia, University of Cambridge, Addenbrooke's Hospital, Cambridge, UK
[48]Center for Stroke Research Berlin, Charité – Universitätsmedizin Berlin, corporate member of Freie Universität Berlin, Humboldt-Universität zu Berlin, and Berlin Institute of Health, Berlin, Germany
[49]Intensive Care Unit, CHR Citadelle, Liège, Belgium
[50]Department of Anaesthesiology and Intensive Therapy, University of Pécs, Pécs, Hungary
[51]Departments of Neurology, Clinical Neurophysiology and Neuroanesthesiology, Region Hovedstaden Rigshospitalet, Copenhagen, Denmark
[52]National Institute for Stroke and Applied Neurosciences, Faculty of Health and Environmental Studies, Auckland University of Technology, Auckland, New Zealand
[53]Department of Neurology, Erasmus MC, Rotterdam, the Netherlands
[54]Department of Anesthesiology and Intensive care, University Hospital Northern Norway, Tromso, Norway
[55]Department of Neurosurgery, Hadassah-hebrew University Medical center, Jerusalem, Israel
[56]Fundación Instituto Valenciano de Neurorrehabilitación (FIVAN), Valencia, Spain
[57]Department of Neurosurgery, Shanghai Renji hospital, Shanghai Jiaotong University/school of medicine, Shanghai, China
[58]Karolinska Institutet, INCF International Neuroinformatics Coordinating Facility, Stockholm, Sweden
[59]Emergency Department, CHU, Liège, Belgium
[60]Neurosurgery clinic, Pauls Stradins Clinical University Hospital, Riga, Latvia
[61]Department of Computing, Imperial College London, London, UK
[62]Department of Neurosurgery, Hospital Universitario 12 de Octubre, Madrid, Spain
[63]Department of Anesthesia, Critical Care and Pain Medicine, Medical University of Vienna, Austria
[64]Department of Public Health, Erasmus Medical Center-University Medical Center, Rotterdam, The Netherlands
[65]College of Health and Medicine, Australian National University, Canberra, Australia
[66]Department of Neurosurgery, Neurosciences Centre & JPN Apex trauma centre, All India Institute of Medical Sciences, New Delhi-110029, India
[67]Department of Neurosurgery, Erasmus MC, Rotterdam, the Netherlands
[68]Department of Neurosurgery, Oslo University Hospital, Oslo, Norway
[69]Division of Psychology, University of Stirling, Stirling, UK

[70]Division of Neurosurgery, Department of Clinical Neurosciences, Addenbrooke's Hospital & University of Cambridge, Cambridge, UK
[71]Department of Neurology, University of Groningen, University Medical Center Groningen, Groningen, Netherlands
[72]Neurointensive Care, Sheffield Teaching Hospitals NHS Foundation Trust, Sheffield, UK
[73]Salford Royal Hospital NHS Foundation Trust Acute Research Delivery Team, Salford, UK
[74]Department of Intensive Care and Department of Ethics and Philosophy of Medicine, Erasmus Medical Center, Rotterdam, The Netherlands
[75]Department of Clinical Neuroscience, Neurosurgery, Umeå University, Umeå, Sweden
[76]Hungarian Brain Research Program - Grant No. KTIA_13_Not ApplicableP-A-II/8, University of Pécs, Pécs, Hungary
[77]Department of Anaesthesiology, University Hospital of Aachen, Aachen, Germany
[78]Cyclotron Research Center, University of Liège, Liège, Belgium
[79]Centre for Urgent and Emergency Care Research (CURE), Health Services Research Section, School of Health and Related Research (ScHARR), University of Sheffield, Sheffield, UK
[80]Emergency Department, Salford Royal Hospital, Salford UK
[81]Institute of Research in Operative Medicine (IFOM), Witten/Herdecke University, Cologne, Germany
[82]VP Global Project Management CNS, ICON, Paris, France
[83]Department of Anesthesiology-Intensive Care, Lille University Hospital, Lille, France
[84]Department of Neurosurgery, Rambam Medical Center, Haifa, Israel
[85]Department of Anesthesiology & Intensive Care, University Hospitals Southhampton NHS Trust, Southhampton, UK
[86]Cologne-Merheim Medical Center (CMMC), Department of Traumatology, Orthopedic Surgery and Sportmedicine, Witten/Herdecke University, Cologne, Germany
[87]Intensive Care Unit, Southmead Hospital, Bristol, Bristol, UK
[88]Department of Neurological Surgery, University of California, San Francisco, California, USA
[89]Department of Anesthesia & Intensive Care,M. Bufalini Hospital, Cesena, Italy
[90]Department of Neurosurgery, University Hospital Heidelberg, Heidelberg, Germany
[91]Department of Neurosurgery, The Walton centre NHS Foundation Trust, Liverpool, UK
[92]Department of Medical Genetics, University of Pécs, Pécs, Hungary
[93]Department of Neurosurgery, Emergency County Hospital Timisoara, Timisoara, Romania
[94]School of Medical Sciences, Örebro University, Örebro, Sweden
[95]Institute for Molecular Medicine Finland, University of Helsinki, Helsinki, Finland
[96]Analytic and Translational Genetics Unit, Department of Medicine; Psychiatric & Neurodevelopmental Genetics Unit, Department of Psychiatry; Department of Neurology, Massachusetts General Hospital, Boston, MA, USA
[97]Program in Medical and Population Genetics; The Stanley Center for Psychiatric Research, The Broad Institute of MIT and Harvard, Cambridge, MA, USA
[98]Department of Radiology, University of Antwerp, Edegem, Belgium
[99]Department of Anesthesiology & Intensive Care, University Hospital of Grenoble, Grenoble, France
[100]Department of Anesthesia & Intensive Care, Azienda Ospedaliera Università di Padova, Padova, Italy
[101]Dept. of Neurosurgery, Leiden University Medical Center, Leiden, The Netherlands and Dept. of Neurosurgery, Medical Center Haaglanden, The Hague, The Netherlands
[102]Department of Neurosurgery, Helsinki University Central Hospital
[103]Division of Clinical Neurosciences, Department of Neurosurgery and Turku Brain Injury Centre, Turku University Hospital and University of Turku, Turku, Finland
[104]Department of Anesthesiology and Critical Care, Pitié -Salpêtrière Teaching Hospital, Assistance Publique, Hôpitaux de Paris and University Pierre et Marie Curie, Paris, France
[105]Neurotraumatology and Neurosurgery Research Unit (UNINN), Vall d'Hebron Research Institute, Barcelona, Spain
[106]Department of Neurosurgery, Kaunas University of technology and Vilnius University, Vilnius, Lithuania
[107]Department of Neurosurgery, Rezekne Hospital, Latvia
[108]Department of Anaesthesia, Critical Care & Pain Medicine NHS Lothian & University of Edinburg, Edinburgh, UK
[109]Director, MRC Biostatistics Unit, Cambridge Institute of Public Health, Cambridge, UK
[110]Department of Physical Medicine and Rehabilitation, Oslo University Hospital/University of Oslo, Oslo, Norway
[111]Division of Orthopedics, Oslo University Hospital, Oslo, Norway
[112]Institue of Clinical Medicine, Faculty of Medicine, University of Oslo, Oslo, Norway
[113]Broad Institute, Cambridge MA Harvard Medical School, Boston MA, Massachusetts General Hospital, Boston MA, USA
[114]National Trauma Research Institute, The Alfred Hospital, Monash University, Melbourne, Victoria, Australia
[115]Department of Neurosurgery, Odense University Hospital, Odense, Denmark
[116]International Neurotrauma Research Organisation, Vienna, Austria
[117]Klinik für Neurochirurgie, Klinikum Ludwigsburg, Ludwigsburg, Germany
[118]Division of Biostatistics and Epidemiology, Department of Preventive Medicine, University of Debrecen, Debrecen, Hungary
[119]Department Health and Prevention, University Greifswald, Greifswald, Germany
[120]Department of Anaesthesiology and Intensive Care, AUVA Trauma Hospital, Salzburg, Austria
[121]Department of Neurology, Elisabeth-TweeSteden Ziekenhuis, Tilburg, the Netherlands

[122]Department of Neuroanesthesia and Neurointensive Care, Odense University Hospital, Odense, Denmark
[123]Department of Neuromedicine and Movement Science, Norwegian University of Science and Technology, NTNU, Trondheim, Norway
[124]Department of Physical Medicine and Rehabilitation, St.Olavs Hospital, Trondheim University Hospital, Trondheim, Norway
[125]Department of Neurosurgery, University of Pécs, Pécs, Hungary
[126]Division of Neuroscience Critical Care, Johns Hopkins University School of Medicine, Baltimore, USA
[127]Department of Neuropathology, Queen Elizabeth University Hospital and University of Glasgow, Glasgow, UK
[128]Dept. of Department of Biomedical Data Sciences, Leiden University Medical Center, Leiden, The Netherlands
[129]Department of Pathophysiology and Transplantation, Milan University, and Neuroscience ICU, Fondazione IRCCS Cà Granda Ospedale Maggiore Policlinico, Milano, Italy
[130]Department of Radiation Sciences, Biomedical Engineering, Umeå University, Umeå, Sweden
[131]Perioperative Services, Intensive Care Medicine and Pain Management, Turku University Hospital and University of Turku, Turku, Finland
[132]Department of Neurosurgery, Kaunas University of Health Sciences, Kaunas, Lithuania
[133]Intensive Care and Department of Pediatric Surgery, Erasmus Medical Center, Sophia Children's Hospital, Rotterdam, The Netherlands
[134]Department of Neurosurgery, Kings college London, London, UK
[135]Neurologie, Neurochirurgie und Psychiatrie, Charité – Universitätsmedizin Berlin, Berlin, Germany
[136]Department of Intensive Care Adults, Erasmus MC– University Medical Center Rotterdam, Rotterdam, the Netherlands
[137]icoMetrix NV, Leuven, Belgium
[138]Movement Science Group, Faculty of Health and Life Sciences, Oxford Brookes University, Oxford, UK
[139]Psychology Department, Antwerp University Hospital, Edegem, Belgium
[140]Director of Neurocritical Care, University of California, Los Angeles, USA
[141]Department of Neurosurgery, St.Olavs Hospital, Trondheim University Hospital, Trondheim, Norway
[142]Department of Emergency Medicine, University of Florida, Gainesville, Florida, USA
[143]Department of Neurosurgery, Charité – Universitätsmedizin Berlin, corporate member of Freie Universität Berlin, Humboldt-Universität zu Berlin, and Berlin Institute of Health, Berlin, Germany
[144]VTT Technical Research Centre, Tampere, Finland
[145]Section of Neurosurgery, Department of Surgery, Rady Faculty of Health Sciences, University of Manitoba, Winnipeg, MB, Canada

*Co-lead investigators: andrew.maas@uza.be (AIRM) and dkm13@cam.ac.uk (DM)

SUPPLEMENTARY METHODS

Hyperparameter optimisation report

Summary

Combinations of the listed hyperparameters were tested on the validation sets of our repeated *k*-fold cross-validation (20 repeats, 5 folds) in successive model versions. A single combination of model hyperparameters is known as a configuration. Configurations which significantly (α=0.01) underperformed in calibration and discrimination on the validation set were dropped out after each repeat using the Bootstrap Bias Corrected with Dropping Cross-Validation (BBCD-CV) method[A3]. For greater detail regarding the role of each hyperparameter in model function, please see the model code in https://github.com/sbhattacharyay/dynamic_GOSE_model/blob/main/scripts/models/dynamic_APM.py.

Overview of tested hyperparameters

- Time window length: length, in hours, of the non-overlapping windows into which ICU stays were partitioned.
  - Tested values: 2, 8, 12, 24
  - Optimal value: 2
- Quantile bin count: number of quantile bins into which numerical variables were discretised for tokenisation.
  - Tested values: 3, 4, 5, 7, 10, 20
  - Optimal value: 20
- Embedding vector dimension: length of vectors learned for each token in the embedding layer.
  - Tested values: 16, 32, 64, 128
  - Optimal value: 128
- Recurrent neural network (RNN) architecture: type of RNN structure.
  - Tested values: long short-term memory (LSTM), gated recurrent unit (GRU)
  - Optimal value: GRU
- Output layer: Encoding of six-month Glasgow Outcome Scale – Extended (GOSE) in model. See the following reference for greater detail[A4].
  - Tested values: softmax (i.e., multinomial encoding), sigmoid (i.e., ordinal encoding)
  - Optimal value: softmax
- Inclusion of temporal tokens: whether to include tokens representing the time of day or time elapsed since ICU admission in patient time windows.
  - Tested values: only time of day tokens, only time from admission tokens, neither, both
  - Optimal value: neither
- Token presentation: how tokens are presented (i.e., transformed or not) to models. See the following reference for greater detail on how "dt-patient-matrix" patient windows are formed[A5].
  - Tested values: "patient-matrix" (i.e., no transformation of tokens), "dt-patient-matrix" (i.e., each patient window contains the difference of tokens from the previous window)
  - Optimal value: "patient-matrix"
- RNN hidden state dimension: dimension of the RNN hidden state.
  - Tested values: 16, 32, 64, 128, 256
  - Optimal value: 128
- Number of RNN layers: number of hidden layers in the RNN architecture.
  - Tested values: 1, 2
  - Optimal value: 1
- Window limit during training: limit to the number of time windows per training set patient considered during training.
  - Tested values: None, 12, 24, 36, 84
  - Optimal value: 84
- Dropout: proportion of embedding layer weights randomly set to zero during training to regularise against overfitting.
  - Tested values: 0, 0.2
  - Optimal value: 0.2
- Learning rate: initial learning rate of Adam stochastic gradient descent algorithm for parameter optimisation.
  - Tested values: 0.001, 0.0003
  - Optimal value: 0.001
- Batch size: number of training set patients used during each iteration of parameter updating during training.
  - Tested values: 1, 4, 8, 32, 64, 128
  - Optimal value: 1
- Maximum training epochs: maximum number of rounds updating model parameters across the full training set of patients.
  - Tested values: 4, 8, 20, 30
  - Optimal value: 30
- Early stopping patience: number of epochs of no improvement in validation set discrimination performance after which training is stopped.
  - Tested values: 10 (if maximum training epochs was 20 or 30)

    - Optimal value: 10

Tested hyperparameters per model version
In successive model versions, we tested different combinations of model hyperparameters, using top performing combinations of untested hyperparameters from previous versions.

Version 0-0: The purpose of this version was to initialise hyperparameter optimisation and test a wide range of configurations.
Tested configurations:
- Quantile bin count:
    - Tested values: 3, 4, 5, 7, 10, 20
    - Optimal value: 20
- Embedding vector dimension:
    - Tested values: 16, 32, 64
    - Optimal value: 64
- RNN architecture:
    - Tested values: LSTM, GRU
    - Optimal value: GRU
- Output layer:
    - Tested values: softmax (i.e., multinomial encoding), sigmoid (i.e., ordinal encoding)
    - Optimal value: softmax
- RNN hidden state dimension:
    - Tested values: 16, 32, 64, 128, 256
    - Optimal value: 128
- Number of RNN layers:
    - Tested values: 1, 2
    - Optimal value: 1
- Dropout: proportion of embedding layer weights randomly set to zero during training to regularise against overfitting.
    - Tested values: 0, 0.2
    - Optimal value: 0.2

Fixed configurations:
- Time window length: 2
- Inclusion of temporal tokens: neither
- Token presentation: "patient-matrix"
- Window limit during training: None
- Learning rate: 0.001
- Batch size: 32
- Maximum training epochs: 30
- Early stopping patience: 10

Version 0-1: The purpose of this version was to test the optimal configurations of the prior version with minor adjustments to the tokenisation algorithm.
Fixed configurations:
- Time window length: 2
- Quantile bin count: 20
- Embedding vector dimension: 128
- RNN architecture: GRU
- Output layer: softmax
- Inclusion of temporal tokens: neither
- Token presentation: "patient-matrix"
- RNN hidden state dimension: 128
- Number of RNN layers: 1
- Window limit during training: None
- Dropout: 0
- Learning rate: 0.001
- Batch size: 32
- Maximum training epochs: 30
- Early stopping patience: 10

Version 0-2: The purpose of this version was to test different optimisation parameters on the optimal configurations of the prior version for each output encoding type.
Tested configurations:

- Quantile bin count:
  - Tested values: 4, 20
  - Optimal value: 20
- Output layer:
  - Tested values: softmax (i.e., multinomial encoding), sigmoid (i.e., ordinal encoding)
  - Optimal value: softmax
- RNN hidden state dimension:
  - Tested values: 16, 128
  - Optimal value: 128
- Learning rate:
  - Tested values: 0.001, 0.0003
  - Optimal value: 0.001
- Batch size:
  - Tested values: 1, 8, 32, 64
  - Optimal value: 1

Fixed configurations:

- Time window length: 2
- Embedding vector dimension: 64
- RNN architecture: GRU
- Inclusion of temporal tokens: neither
- Token presentation: "patient-matrix"
- Number of RNN layers: 1
- Window limit during training: None
- Dropout: 0
- Maximum training epochs: 30
- Early stopping patience: 10

Version 0-3: The purpose of this version was to test different optimisation parameters on the optimal configurations of the prior version for each output encoding type.
Tested configurations:

- Quantile bin count:
  - Tested values: 4, 20
  - Optimal value: 20
- Output layer:
  - Tested values: softmax (i.e., multinomial encoding), sigmoid (i.e., ordinal encoding)
  - Optimal value: softmax
- RNN hidden state dimension:
  - Tested values: 16, 128
  - Optimal value: 128
- Maximum training epochs:
  - Tested values: 4, 8
  - Optimal value: 8

Fixed configurations:

- Time window length: 2
- Embedding vector dimension: 64
- RNN architecture: GRU
- Inclusion of temporal tokens: neither
- Token presentation: "patient-matrix"
- Number of RNN layers: 1
- Window limit during training: None
- Dropout: 0
- Learning rate: 0.001
- Batch size: 1
- Early stopping patience: 10

Version 1-0: The purpose of this version was to test the optimal configurations of the prior version with repeated cross-validation
Tested configurations:

- Quantile bin count:
    - Tested values: 4, 20
    - Optimal value: 20
- Output layer:
    - Tested values: softmax (i.e., multinomial encoding), sigmoid (i.e., ordinal encoding)
    - Optimal value: softmax
- RNN hidden state dimension:
    - Tested values: 16, 128
    - Optimal value: 128
- Maximum training epochs:
    - Tested values: 4, 8
    - Optimal value: 8

Fixed configurations:

- Time window length: 2
- Embedding vector dimension: 64
- RNN architecture: GRU
- Inclusion of temporal tokens: neither
- Token presentation: “patient-matrix”
- Number of RNN layers: 1
- Window limit during training: None
- Dropout: 0
- Learning rate: 0.001
- Batch size: 1
- Early stopping patience: 10

Version 2-0: The purpose of this version was to test the effect of modifying the time window length on model information and calibration.
Tested configurations:

- Time window length:
    - Tested values: 2, 8, 12, 24
    - Optimal value: 2

Fixed configurations:

- Quantile bin count: 20
- Embedding vector dimension: 64
- RNN architecture: GRU
- Output layer: softmax
- Inclusion of temporal tokens: neither
- Token presentation: “patient-matrix”
- RNN hidden state dimension: 128
- Number of RNN layers: 1
- Window limit during training: None
- Dropout: 0.2
- Learning rate: 0.001
- Batch size: 4
- Maximum training epochs: 20
- Early stopping patience: 10

Version 3-0:
Fixed configurations:

- Time window length: 2
- Quantile bin count: 20
- Embedding vector dimension: 64
- RNN architecture: GRU
- Output layer: softmax

- Inclusion of temporal tokens: neither
- Token presentation: “patient-matrix”
- RNN hidden state dimension: 128
- Number of RNN layers: 1
- Window limit during training: None
- Dropout: 0.2
- Learning rate: 0.001
- Batch size: 4
- Maximum training epochs: 20
- Early stopping patience: 10

Version 4-0: The purpose of this version was to test the effect of limiting the number of time windows per patient read from the training set during model training.
Tested configurations:
- Window limit during training: limit to the number of time windows per training set patient considered during training.
  - Tested values: None, 84
  - Optimal value: 84
- Batch size:
  - Tested values: 1, 4, 32, 128
  - Optimal value: 1

Fixed configurations:
- Time window length: 2
- Quantile bin count: 20
- Embedding vector dimension: 64
- RNN architecture: GRU
- Output layer: softmax
- Inclusion of temporal tokens: neither
- Token presentation: “patient-matrix”
- RNN hidden state dimension: 128
- Number of RNN layers: 1
- Dropout: 0.2
- Learning rate: 0.001
- Maximum training epochs: 30
- Early stopping patience: 10

Version 5-0: The purpose of this version was to test both the effect of limiting the number of time windows per patient read from the training set during model training and the pre-training transformation of patient windows to only focus on changes in clinical events.
Tested configurations:
- Token presentation:
  - Tested values: “patient-matrix” (i.e., no transformation of tokens), “dt-patient-matrix” (i.e., each patient window contains the difference of tokens from the previous window)
  - Optimal value: “patient-matrix”
- Window limit during training: limit to the number of time windows per training set patient considered during training.
  - Tested values: None, 12, 24, 36, 84
  - Optimal value: 84

Fixed configurations:
- Time window length: 2
- Quantile bin count: 20
- Embedding vector dimension: 64
- RNN architecture: GRU
- Output layer: softmax
- Inclusion of temporal tokens: neither
- RNN hidden state dimension: 128
- Number of RNN layers: 1
- Dropout: 0.2
- Learning rate: 0.001
- Batch size: 1

- Maximum training epochs: 30
- Early stopping patience: 10

Version 6-0: This version resulted in the best performing models, overall. We also tested calibration techniques on the validation set to improve calibration performance. These techniques included matrix scaling, vector scaling, and temperature scaling on validation set predictions[A6].

Tested configurations:

- Embedding vector dimension:
  - Tested values: 32, 64, 128
  - Optimal value: 128
- Inclusion of temporal tokens:
  - Tested values: only time of day tokens, only time from admission tokens, neither, both
  - Optimal value: neither
- RNN hidden state dimension:
  - Tested values: 32, 64, 128
  - Optimal value: 128
- Token presentation:
  - Tested values: "patient-matrix" (i.e., no transformation of tokens), "dt-patient-matrix" (i.e., each patient window contains the difference of tokens from the previous window)
  - Optimal value: "patient-matrix"
- Window limit during training: limit to the number of time windows per training set patient considered during training.
  - Tested values: None, 12, 24, 84
  - Optimal value: 84

Fixed configurations:

- Time window length: 2
- Quantile bin count: 20
- RNN architecture: GRU
- Output layer: softmax
- Number of RNN layers: 1
- Dropout: 0.2
- Learning rate: 0.001
- Batch size: 1
- Maximum training epochs: 30
- Early stopping patience: 10

Version 7-0: This purpose of this version was to test model performance after excluding explicit physician-based impressions (see Supplementary Note 2).

Tested configurations:

- RNN architecture:
  - Tested values: LSTM, GRU
  - Optimal value: LSTM
- Embedding vector dimension:
  - Tested values: 32, 64, 128
  - Optimal value: 128
- Inclusion of temporal tokens:
  - Tested values: only time of day tokens, only time from admission tokens, neither, both
  - Optimal value: neither
- RNN hidden state dimension:
  - Tested values: 32, 64, 128
  - Optimal value: 64
- Token presentation:
  - Tested values: "patient-matrix" (i.e., no transformation of tokens), "dt-patient-matrix" (i.e., each patient window contains the difference of tokens from the previous window)
  - Optimal value: "patient-matrix"
- Window limit during training: limit to the number of time windows per training set patient considered during training.
  - Tested values: None, 12, 24, 84
  - Optimal value: 84

Fixed configurations:

- Time window length: 2
- Quantile bin count: 20
- Output layer: softmax
- Number of RNN layers: 1
- Dropout: 0.2
- Learning rate: 0.001
- Batch size: 1
- Maximum training epochs: 30
- Early stopping patience: 10

Calculation of Somers' D_{xy}

The calculation of Somers' D_{xy} begins by converting the estimated probabilities of threshold-level six-month GOSE into individual GOSE probability scores ($\hat{p}_i$):

$$\hat{p}_i \triangleq \widehat{\Pr}(\text{GOSE} = \text{GOSE}_i) = \begin{cases} 1 - \widehat{\Pr}(\text{GOSE} > \text{GOSE}_i), \text{ for } i = 0 \\ \widehat{\Pr}(\text{GOSE} > \text{GOSE}_{i-1}) - \widehat{\Pr}(\text{GOSE} > \text{GOSE}_i), \text{ for } i \in [1,2,3,4,5] \\ \widehat{\Pr}(\text{GOSE} > \text{GOSE}_{i-1}), \text{ for } i = 6 \end{cases} \quad (1)$$

where $i \in [0,1,2,3,4,5,6]$ represents the index of possible GOSE ($\text{GOSE}_0 = 1$, $\text{GOSE}_1 = 2$ or 3, $\text{GOSE}_2 = 4$, $\text{GOSE}_3 = 5$, $\text{GOSE}_4 = 6$, $\text{GOSE}_5 = 7$, $\text{GOSE}_6 = 8$). Then, the expected six-month GOSE index is estimated by $\widehat{\mathbb{E}}[i] = \sum_{i=0}^{6} i \cdot \hat{p}_i$. A comparable pair is defined as a pair of patients with different six-month GOSE scores, and the total number of unique comparable pairs is denoted as N^{comp}. A concordant pair is a comparable pair in which the patient with the higher six-month GOSE also has a higher model-estimated $\widehat{\mathbb{E}}[i]$, and the total number of unique concordant pairs is denoted as N^{conc}. Since $N^{\text{conc}} = 0.5N^{\text{comp}}$ if there is no association between $\widehat{\mathbb{E}}[i]$ and six-month GOSE, Somers' D_{xy} (S) is defined as:

$$S \triangleq \frac{N^{\text{conc}} - \frac{1}{2}N^{\text{comp}}}{\frac{1}{2}N^{\text{comp}}} \quad (2).$$

SUPPLEMENTARY REFERENCES

A1. Harrell FE, Jr. Ordinal Logistic Regression. In: Harrell FE, Jr, ed. *Regression Modeling Strategies: With Applications to Linear Models, Logistic and Ordinal Regression, and Survival Analysis*. Springer 2015: 311–25. doi:10.1007/978-3-319-19425-7_13.

A2. Licht C. *New methods for generating significance levels from multiply-imputed data* [Dr rer pol thesis]. Bamberg: University of Bamberg; 2010.

A3. Tsamardinos I, Greasidou E, Borboudakis G. Bootstrapping the out-of-sample predictions for efficient and accurate cross-validation. *Mach Learning* 2018; **107**(12): 1895–922. doi:10.1007/s10994-018-5714-4.

A4. Bhattacharyay S, Milosevic I, Wilson L, et al. The leap to ordinal: Detailed functional prognosis after traumatic brain injury with a flexible modelling approach. *PLoS ONE* 2022; **17**(7): e0270973. doi:10.1371/journal.pone.0270973.

A5. Ho LV, Aczon M, Ledbetter D, Wetzel R. Interpreting a recurrent neural network's predictions of ICU mortality risk. *J Biomed Inform* 2021; **114**: 103672. doi:10.1016/j.jbi.2021.103672.

A6. Guo C, Pleiss G, Sun Y, Weinberger KQ. On calibration of modern neural networks. In: Precup D, The YW, eds. *Proceedings of the 34th International Conference on Machine Learning – Volume 70 (ICML '17)*. JMLR 2017: 1321–30.
