# Supplementary material for: Mining the contribution of intensive care clinical course to outcome after traumatic brain injury
Source: NPJ Digit Med. 2023 Aug 21;6:154. doi: 10.1038/s41746-023-00895-8 (PMC10442346; doi:10.1038/s41746-023-00895-8)
Supplement: Supplementary file 2 — Reporting Summary [file 41746_2023_895_MOESM2_ESM.pdf]

nature portfolio

Corresponding author(s): Shubhayu Bhattacharyay

Last updated by author(s): Jul 24, 2023

Reporting Summary

Nature Portfolio wishes to improve the reproducibility of the work that we publish. This form provides structure for consistency and transparency in reporting. For further information on Nature Portfolio policies, see our Editorial Policies and the Editorial Policy Checklist.

Statistics

For all statistical analyses, confirm that the following items are present in the figure legend, table legend, main text, or Methods section.

n/a	Confirmed	
☐	☒	The exact sample size (*n*) for each experimental group/condition, given as a discrete number and unit of measurement
☐	☒	A statement on whether measurements were taken from distinct samples or whether the same sample was measured repeatedly
☐	☒	The statistical test(s) used AND whether they are one- or two-sided<br>*Only common tests should be described solely by name; describe more complex techniques in the Methods section.*
☐	☒	A description of all covariates tested
☒	☐	A description of any assumptions or corrections, such as tests of normality and adjustment for multiple comparisons
☐	☒	A full description of the statistical parameters including central tendency (e.g. means) or other basic estimates (e.g. regression coefficient) AND variation (e.g. standard deviation) or associated estimates of uncertainty (e.g. confidence intervals)
☒	☐	For null hypothesis testing, the test statistic (e.g. *F*, *t*, *r*) with confidence intervals, effect sizes, degrees of freedom and *P* value noted<br>*Give P values as exact values whenever suitable.*
☒	☐	For Bayesian analysis, information on the choice of priors and Markov chain Monte Carlo settings
☒	☐	For hierarchical and complex designs, identification of the appropriate level for tests and full reporting of outcomes
☒	☐	Estimates of effect sizes (e.g. Cohen's *d*, Pearson's *r*), indicating how they were calculated

Our web collection on statistics for biologists contains articles on many of the points above.

Software and code

Policy information about availability of computer code

Data collection	Data for the CENTER-TBI study was collected through the QuesGen electronic case report form system (QuesGen Systems Inc, Burlingame, CA, USA) hosted on the International Neuroinformatics Coordinating Facility (INCF) platform (INCF, Stockholm, Sweden). All data were extracted from the CENTER-TBI core study (v3.0, ICU stratum) using Opal database software.
Data analysis	All code used for analysis in this project can be found at the following online repository: https://github.com/sbhattacharyay/dynamic_GOSE_model (doi:10.5281/zenodo.7668551). The primary software used was Python (v3.8.2).

For manuscripts utilizing custom algorithms or software that are central to the research but not yet described in published literature, software must be made available to editors and reviewers. We strongly encourage code deposition in a community repository (e.g. GitHub). See the Nature Portfolio guidelines for submitting code & software for further information.

Data

Policy information about availability of data

All manuscripts must include a data availability statement. This statement should provide the following information, where applicable:

- Accession codes, unique identifiers, or web links for publicly available datasets
- A description of any restrictions on data availability
- For clinical datasets or third party data, please ensure that the statement adheres to our policy

Individual participant data, including data dictionary, the study protocol, and analysis scripts are available online, conditional to approved study proposal, with no

end date. Interested investigators must submit a study proposal to the management committee at https://www.center-tbi.eu/data. Signed confirmation of a data access agreement is required, and all access must comply with regulatory restrictions imposed on the original study.

Research involving human participants, their data, or biological material

Policy information about studies with human participants or human data. See also policy information about sex, gender (identity/presentation), and sexual orientation and race, ethnicity and racism.

Reporting on sex and gender	Sex is reported in CENTER-TBI as a Male/Female binary based on a patient's medical chart. Gender was not assessed. The distribution of sex in our dataset is listed in Table 2 of the manuscript. Sex was included in the full-variable disease course modelling to determine whether sex was associated with outcome and, if so, decode the link to generate a hypothesis about potential sex-based disparities in TBI care and outcome. Many other demographic, socioeconomic status, and clinical variables (full list available here: https://www.center-tbi.eu/data/dictionary) were included to disentangle confounding factors. In our results (supplementary information, pp 9), sex was associated with outcome. This is not a confirmation of a direct, causal relationship between sex and outcome after TBI, and several prior studies have explored this link and possible confounding factors that are not encoded in our dataset: Gupte R, et al. Sex Differences in Traumatic Brain Injury: What We Know and What We Should Know. J Neurotrauma. 2019 Nov 15;36(22):3063-3091. doi: 10.1089/neu.2018.6171. Epub 2019 Jul 19. PMID: 30794028; PMCID: PMC6818488.
Reporting on race, ethnicity, or other socially relevant groupings	Race is reported in CENTER-TBI as one of the following broad categories: Asian (n=20), Black (n=21), White (n=1,386), Not allowed (i.e., patient did not consent to racial categorisation, n=3), and Unknown (n=31). No other similar relevant social grouping (e.g., ethnicity) was assessed. Race was included in the full-variable disease course modelling to determine whether race was associated with outcome and, if so, decode the link to generate a hypothesis about potential racial disparities in TBI care and outcome. Many other socioeconomic status variables (e.g., education level and employment status; full list available here: https://www.center-tbi.eu/crf/Tree/DemographicsandSocioeconomicStatus.pdf) were included to disentangle confounding factors. In our results (supplementary information, pp 9), none of the racial categories in CENTER-TBI had a significant association with outcome based on 95% confidence intervals: Asian (TimeSHAP: [-0.025 – 0.006]) , Black (TimeSHAP: [-0.003 – 0.003]), Not allowed (TimeSHAP: [-0.003 – 0.008]), Unknown (TimeSHAP: [-0.022 – 0.001]), and White (TimeSHAP: [-0.006 – 0.004]). This does not rule out a racial disparity in TBI care, and we encourage investigators to test our modelling strategy on populations more representative of non-White populations.
Population characteristics	See Table 2 in manuscript. For more detailed information on study population characteristics, please see interactive frequency tables at https://www.center-tbi.eu/data/dictionary and the tables in the following published article: Steyerberg EW, et al. Case-mix, care pathways, and outcomes in patients with traumatic brain injury in CENTER-TBI: a European prospective, multicentre, longitudinal, cohort study. Lancet Neurol. 2019 Oct;18(10):923-934. doi: 10.1016/S1474-4422(19)30232-7. PMID: 31526754.
Recruitment	Inclusion criteria were a clinical diagnosis of TBI, indication for CT scanning, presentation to study centre within 24 h of injury, and informed consent obtained according to local and national requirements. Participants were excluded if they had any severe pre-existing neurological disorder that could confound outcome assessments. Patients were differentiated by care pathway into three strata: (1) emergency room (ER) stratum (patients assessed in the ER and discharged), (2) admission stratum (admitted to hospital ward), and (3) intensive care unit (ICU) stratum (primary admission to the intensive care unit). The assignment to a stratum was done prospectively. More information on the recruitment strategy of CENTER-TBI (including discussion of potential sampling biases) has been published by: Steyerberg EW, et al. Case-mix, care pathways, and outcomes in patients with traumatic brain injury in CENTER-TBI: a European prospective, multicentre, longitudinal, cohort study. Lancet Neurol. 2019 Oct;18(10):923-934. doi: 10.1016/S1474-4422(19)30232-7. PMID: 31526754.
Ethics oversight	The CENTER-TBI study (EC grant 602150) has been conducted in accordance with all relevant laws of the EU if directly applicable or of direct effect and all relevant laws of the country where the Recruiting sites were located, including but not limited to, the relevant privacy and data protection laws and regulations (the “Privacy Law”), the relevant laws and regulations on the use of human materials, and all relevant guidance relating to clinical studies from time to time in force including, but not limited to, the ICH Harmonised Tripartite Guideline for Good Clinical Practice (CPMP/ICH/135/95) (“ICH GCP”) and the World Medical Association Declaration of Helsinki entitled “Ethical Principles for Medical Research Involving Human Subjects”. Informed Consent by the patients and/or the legal representative/next of kin was obtained, accordingly to the local legislations, for all patients recruited in the Core Dataset of CENTER-TBI and documented in the e-CRF.<br><br>Ethical approval was obtained for each recruiting sites. The list of sites, Ethical Committees, approval numbers and approval dates can be found at: https://www.center-tbi.eu/project/ethical-approval

Note that full information on the approval of the study protocol must also be provided in the manuscript.

Field-specific reporting

Please select the one below that is the best fit for your research. If you are not sure, read the appropriate sections before making your selection.

☒ Life sciences ☐ Behavioural & social sciences ☐ Ecological, evolutionary & environmental sciences

For a reference copy of the document with all sections, see nature.com/documents/nr-reporting-summary-flat.pdf

Life sciences study design

All studies must disclose on these points even when the disclosure is negative.

Sample size	The predetermination of CENTER-TBI study sample was explained in: Maas AIR, et al. Collaborative European NeuroTrauma Effectiveness Research in Traumatic Brain Injury (CENTER-TBI): a prospective longitudinal observational study. Neurosurgery. 2015 Jan;76(1):67-80. doi: 10.1227/NEU.0000000000000575. PMID: 25525693. The objective for our work was to maximise the sample size based on availability in the CENTER-TBI dataset. In the end, Of the 2,138 CENTER-TBI patients available for analysis in the ICU stratum of the core study, 1,550 met the additional inclusion criteria of this work (see inclusion criteria below). Suitability of sample size was assessed with model calibration. If sample sizes are too low, models are overfit and miscalibrated. Our results (Figures 1c and 1d) demonstrate suitable calibration and therefore suitable sample size for our analyses.
Data exclusions	During study recruitment, patients were excluded if they had any severe pre-existing neurological disorder that could confound outcome assessments. During our analysis, we additionally excluded patients who did not meet the pre-defined study criteria: (1) primary admission to the ICU for at least 24 hours, (2) at least 16 years old, and (3) availability of functional outcome assessment at six months post-injury. We required these criteria because: (1) our models require sufficient ICU stay duration (i.e., a day) for calibration, (2) paediatric TBI patients have different pathophysiology and clinical care pathways, and (3) our primary outcome was necessary for model interpretation and assessment.
Replication	The reproducibility and robustness of findings were assessed with repeated cross-validation and bootstrapping, as described in the Methods. We also had two investigators run model training and testing set output with fixed random seed values to confirm results. No other replication was performed since the objective of the study was not to produce a specific, pre-trained, generalisable model but rather to test the use of a modelling strategy (as mentioned in the Discussion).
Randomization	Randomisation and/or group allocation was not performed since the study was observational and randomisation was not relevant.
Blinding	Blinding was not performed since the study was observational and blinding was not relevant.

Reporting for specific materials, systems and methods

We require information from authors about some types of materials, experimental systems and methods used in many studies. Here, indicate whether each material, system or method listed is relevant to your study. If you are not sure if a list item applies to your research, read the appropriate section before selecting a response.

Materials & experimental systems

n/a	Involved in the study	
☒	☐	Antibodies
☒	☐	Eukaryotic cell lines
☒	☐	Palaeontology and archaeology
☒	☐	Animals and other organisms
☐	☒	Clinical data
☒	☐	Dual use research of concern
☒	☐	Plants

Methods

n/a	Involved in the study	
☒	☐	ChIP-seq
☒	☐	Flow cytometry
☒	☐	MRI-based neuroimaging

Clinical data

Policy information about clinical studies
All manuscripts should comply with the ICMJE guidelines for publication of clinical research and a completed CONSORT checklist must be included with all submissions.

Clinical trial registration	NCT02210221
Study protocol	The full study protocol of CENTER-TBI can be accessed here: https://clinicaltrials.gov/ProvidedDocs/21/NCT02210221/Prot_SAP_000.pdf
Data collection	The timeline of patient recruitment (19 Dec 2014 - 17 Dec 2017) and data collection (19 Dec 2014 - 31 Mar 2021) can be found here: https://www.center-tbi.eu/project/progression. The 65 participating centres and recruiting institutions can be found here: https://www.center-tbi.eu/project/institutes.
Outcomes	The primary outcome in our study was six-month functional outcome scored with the Glasgow Outcome Scale - Extended (GOSE) between five and eight months after injury. GOSE was scored via structured interview and patient/carer questionnaire, and all scores were reviewed by a central reviewer. The full list of CENTER-TBI outcomes are as follows:<br>Primary Outcome Measures :<br>Glasgow Outcome Scale - Extended (GOSE) at 6 Months [Time Frame: 6 months]<br>The Extended Glasgow Outcome Scale is a global scale for functional outcome that rates patient status into 8 categories, going from dead to good recovery.<br><br>Death

Vegetative sate
Lower severe disability
Upper severe disability
Lower moderate disability
Upper moderate disability - some disability but can potentially return to some form of employment
Lower good recovery - minor physical or mental defect
Upper good recovery - full recovery
The 6-month GOSE score is available in 3804 patients (84%).

SF-12v2 Health Survey (Short-Form Health Survey With 12 Questions) at 6 Months [Time Frame: 6 months]
The SF-12v2 Health Survey uses 12 questions to measure functional health and well-being from the patient's point of view. The SF-12v2 at 6 months is available in 2300 patients.

6 Month Quality of Life in Brain Injury (Qolibri-OS) <52 (Impaired) [Time Frame: 6 months]
The Quality of Life in Brain Injury (Qolibri-OS) is a 6 item overall scale that provides a profile of health-related quality of life in domains typicality affected by brain injury, such as physical function, cognition, emotional status, ability to perform daily activities, personal life and social relationship, and satisfaction with current situation and future prospects.

The QOLIBRI scores are reported on a 0-100 scale , where 0=worst possible quality of life and 100=best possible quality of life.

Secondary Outcome Measures :
6 Month Post-traumatic Stress Disorder (PTSD) Checklist for DSM-5 (PCL-5) <33 (Impaired) [Time Frame: 6 months]
The PCL-5 is a self-report rating scale intended to assess 20 DSM-5 symptoms of Posttraumatic Stress Disorder. The standard recall period for the PCL-5 is one month. For CENTER-TBI a recall period of one week was used at the 2-3 week assessment, and the standard one month recall period was used at other time points. The sum of scores can range from 0 to 80, where high scores indicate more pronounced PTSD symptoms.

6 Month Rivermead Post Concussion Questionnaire <16 (Impaired) [Time Frame: 6 months]
The Rivermead PCS Questionnaire (RPQ) was originally developed as a measure of severity of symptoms following MTBI. It consists of 16 post-concussion symptoms including headaches, dizziness, nausea/vomiting, noise sensitivity, sleep disturbance, fatigue, irritability, feeling depressed/tearful, feeling frustrated/ impatient, forgetfulness, poor concentration, taking longer to think, blurred vision, light sensitivity, double vision and restlessness. In the original version of the RPQ, participants are asked to rate the degree (on a scale of 0 to 4) to which a particular symptom has been absent or a mild, moderate or severe problem over the previous 7 days compared with premorbid levels. Total scores range from 0 to 64 with higher scores indicating more severe symptoms. Scores equal to or greater than 16 were considered indicative of persisting post-concussion symptoms.

6 Month Galveston Orientation and Amnesia Test (GOAT) < 75 (Impaired) [Time Frame: 6 months]
The GOAT is a standardised assessment used to determine whether a participant is in post-traumatic amnesia (PTA). PTA is an early phase of TBI recovery during which the person with injury shows markedly impaired memory, confusion, fluctuation in performance, disorientation, and other neurobehavioral signs and symptoms. GOAT questions assess orientation, memory for the first event that the participant can recall after the injury, and memory for the last event that the participant can recall from before the injury.

The GOAT's total score must be achieved by subtracting from 100 the total amount of error scores (Total score = 100 - total amount of error scores). Scores lower than 75 point to the fact that the victim is still experiencing amnesia.

6 Month Rey Auditory Verbal Learning Test (RAVLT) - Impaired [Time Frame: 6 months]
The Rey Auditory Verbal Learning Test (RAVLT) is a test of verbal memory that assesses the ability to acquire 15 words. Recall is assessed after each presentation of the list, after the recall of an interference list, and again following a 20-minute delay. The total score for recall of the principal list ranges from 0 to 75, with higher scores indicating better performance.

Impairment was defined as performance that was 1.33 SDs below the mean of a reference group of healthy peers.

6 Month Timed up and go Test - Impaired [Time Frame: 6 months]
In the timed up and go (TUG) test, subjects are asked to rise from a standard armchair, walk to a marker 3 m away, turn, walk back, and sit down again, for quantifying functional mobility. The presence of slowness, hesitancy, abnormal trunk or arm movements, staggering or stumbling is used to grade the patient from 1 (normal) to 5 (severely abnormal). Impaired mobility was defined as taking 14 seconds or longer to perform the TUG test.

6 Month JK Coma Recovery Scale - Revised <23 (Impaired) [Time Frame: 6 months]
The Coma Recovery Scale- Revised (CRS-R) is a standardized behavioral assessment instrument designed to measure neurobehavioral function in patients with disorders of consciousness (DOC). The CRS-R is comprised of six subscales addressing auditory, visual, motor, oromotor/verbal, communication and arousal functions. Subscale items are hierarchically-arranged, corresponding to brain stem, subcortical and cortically-mediated functions. Scores range between 0 (deep coma) and 23 (able to follow commands and to use objects purposefully). A total score less than 23 indicates impairment.

6 Month Trail Making Test (TMT) Part A - Impaired [Time Frame: 6 months]
The Trail Making Test (TMT) is a measure of attention, speed, and mental flexibility. Part A requires the individual to draw lines to connect 25 encircled numbers distributed on a page. Part B is similar except the person must alternate between numbers and letters and is more difficult and takes longer to complete. Both sections are timed and the score represents the amount of time required to complete the task, with shorter times indicating better performance. The maximum time allowed is 100 seconds.

Impairment cutoff: > 55.9 (based on the comparison group as a whole and expressed as raw score).

For all the cognitive tests impairment was defined as performance that was 1.33 SDs below the mean of a reference group of healthy peers. Raw scores indicating impaired performance could be > or < than the mean depending on the test (eg. on tests of accuracy the cut-off will be a score 1.33 SDs below the mean, while on a timed test the cut-off will be 1.33 SDs above the mean).

6 Month Trail Making Test (TMT) Part B - Impaired [Time Frame: 6 months]
The TMT is a measure of attention, speed, and mental flexibility. Part A requires the individual to draw lines to connect 25 encircled numbers distributed on a page. Part B is similar except the person must alternate between numbers and letters, is more difficult, takes longer to complete. Both sections are timed, the score represents the amount of time required to complete the task, with shorter times indicating better performance.

Impairment cutoff: > 143.7 (based on the comparison group as a whole and expressed as raw score).

For all the cognitive tests impairment was defined as performance that was 1.33 SDs below the mean of a reference group of healthy peers. Raw scores indicating impaired performance could be > or < than the mean depending on the test (eg. on tests of accuracy the cut-off will be a score 1.33 SDs below the mean, while on a timed test the cut-off will be 1.33 SDs above the mean).

6 Month Cambridge Neuropsychological Test Automated Battery (CANTAB) PAL (Paired Associate Learning Task) - Impaired [Time Frame: 6 months]
CANTAB is a computerized neuropsychological battery examining a range of domains including attention, memory and executive functioning. Using mainly nonverbal stimuli, the test is language- and culture-independent. For CANTAB PAL, the individual must remember 1 to 8 patterns displayed in different positions on the screen. The score is the number of incorrect responses adjusted if necessary for trials that have not been completed. Scores ranged from 0-194 with lower score indicating better performance.

Impairment cutoff: > 72.3 (based on the comparison group as a whole and expressed as raw score).

For all the cognitive tests impairment was defined as performance that was 1.33 SDs below the mean of a reference group of healthy peers. Raw scores indicating impaired performance could be > or < than the mean depending on the test (eg. on tests of accuracy the cut-off will be a score 1.33 SDs below the mean, while on a timed test the cut-off will be 1.33 SDs above the mean).

6 Month Cambridge Neuropsychological Test Automated Battery (CANTAB) RTI (Reaction Time Task) - Impaired [Time Frame: 6 months]
The CANTAB is a computerized neuropsychological battery examining a range of domains including attention, memory and executive functioning. Using mainly nonverbal stimuli, the test is language- and culture-independent. For CANTAB RTI, the individual must respond as quickly as possible to a circle presented at one of 5 positions. The outcome measure is the median time for correct responses (ms).

Impairment cutoff: > 470.9 (based on the comparison group as a whole and expressed as raw score).

For all the cognitive tests impairment was defined as performance that was 1.33 SDs below the mean of a reference group of healthy peers. Raw scores indicating impaired performance could be > or < than the mean depending on the test (eg. on tests of accuracy the cut-off will be a score 1.33 SDs below the mean, while on a timed test the cut-off will be 1.33 SDs above the mean).

6 Month Cambridge Neuropsychological Test Automated Battery (CANTAB) SWM (Spatial Working Memory Task) - Impaired [Time Frame: 6 months]
The CANTAB is a computerized neuropsychological battery examining a range of domains including attention, memory and executive functioning. Using mainly nonverbal stimuli, the test is language- and culture-independent. For the CANTAB SWM, the participant searches for tokens in boxes on the screen. The between errors measure is the number of times the participant queries a box that has already been searched. Errors ranged from 0 to 151, with lower numbers indicating better performance.

Impairment cutoff: > 52.0 (based on the comparison group as a whole and expressed as raw score).

For all the cognitive tests impairment was defined as performance that was 1.33 SDs below the mean of a reference group of healthy peers. Raw scores indicating impaired performance could be > or < than the mean depending on the test (eg. on tests of accuracy the cut-off will be a score 1.33 SDs below the mean, while on a timed test the cut-off will be 1.33 SDs above the mean).

6 Month Cambridge Neuropsychological Test Automated Battery (CANTAB) RVP (Rapid Visual Information Processing Task) - Impaired [Time Frame: 6 months]
The CANTAB is a computerized neuropsychological battery examining a range of domains including attention, memory and executive functioning. Using mainly nonverbal stimuli, the test is language- and culture-independent. For the CANTAB RVP, sequences of numbers must be detected as they appear on the screen. The measure of accuracy is A' (A prime) which is derived from hits and correct rejections. The maximum score is 1 and, a higher A' indicates better performance.

Impairment cutoff: < 0.82 (based on the comparison group as a whole and expressed as raw score).

For all the cognitive tests impairment was defined as performance that was 1.33 SDs below the mean of a reference group of healthy peers. Raw scores indicating impaired performance could be > or < than the mean depending on the test (eg. on tests of accuracy the cut-off will be a score 1.33 SDs below the mean, while on a timed test the cut-off will be 1.33 SDs above the mean).

6 Month Cambridge Neuropsychological Test Automated Battery (CANTAB) SOC (Stockings of Cambridge Task) - Impaired [Time Frame: 6 months]
The CANTAB is a computerized neuropsychological battery examining a range of domains including attention, memory and executive functioning. Using mainly nonverbal stimuli, the test is language- and culture-independent. For the CANTAB SOC, the individual moves circles to match a target in this task based on the Tower of Hanoi game. The score is the number of problems solved in the minimum number of moves, with a maximum of 12. Higher scores indicate better performance.

Impairment cutoff: < 5.7(based on the comparison group as a whole and expressed as raw score).

For all the cognitive tests impairment was defined as performance that was 1.33 SDs below the mean of a reference group of healthy peers. Raw scores indicating impaired performance could be > or < than the mean depending on the test (eg. on tests of accuracy the cut-off will be a score 1.33 SDs below the mean, while on a timed test the cut-off will be 1.33 SDs above the mean).

6 Month Cambridge Neuropsychological Test Automated Battery (CANTAB) AST (Attention Switching Task) - Impaired [Time Frame: 6 months]
The CANTAB is a computerized neuropsychological battery examining a range of domains including attention, memory and executive functioning. Using mainly nonverbal stimuli, the test is language- and culture-independent. For the CANTAB AST, participants respond either to the position of an arrow or the direction it is pointing, after being cued to the task on each trial. Total correct responses range from 0 to 160, where higher numbers indicate better performance.

Impairment cutoff: <135.5 (based on the comparison group as a whole and expressed as raw score).

For all the cognitive tests impairment was defined as performance that was 1.33 SDs below the mean of a reference group of healthy peers. Raw scores indicating impaired performance could be > or < than the mean depending on the test (eg. on tests of accuracy the cut-off will be a score 1.33 SDs below the mean, while on a timed test the cut-off will be 1.33 SDs above the mean).

Early (2-3 Weeks) MRI Imaging - Traumatic Intracranial Abnormalities [Time Frame: Between 2-3 weeks after enrolment]
A selected number of sites performed MRI follow up in a subset of patients that consented for imaging data collection. The CENTER-TBI MR protocols included a high-resolution 3D T1-weighted, a T2-weighted, a FLAIR, a DTI, a T2* sequence (gradient echo and/or SWI), and (optionally) a resting-state fMRI. Across all stratum the MRI sites performed a follow up MRI at 2-3 weeks after injury.

Traumatic Intracranial Abnormalities were assessed according to the TBI-Common Data Elements (CDEs). It indicates whether any of the 12 following imaging abnormalities are present (Mass lesion, Extra-axial Hematoma, Epidural Hematoma, Subdural Hematoma Acute, Subdural Hematoma Subacute Chronic, Subdural Collection Mixed Density, Contusion, TAI, traumatic Subarachnoid Hemorrhage, Intraventricular Hemorrhage, Midline Shift or Cisternal Compression.

Biospecimen Retention: Samples With DNA
whole blood samples
